# Beneficial rhizobacteria and virus infection modulate the soybean metabolome and influence the feeding preferences of the virus vector *Epilachna varivestis*

**DOI:** 10.1111/nph.71104

**Published:** 2026-03-24

**Authors:** Hannier Pulido, Kerry E. Mauck, Consuelo M. De Moraes, Mark C. Mescher

**Affiliations:** ^1^ Department of Environmental Systems Science Swiss Federal Institute of Technology (ETH Zürich) 8092 Zürich Switzerland; ^2^ Department of Entomology University of California, Riverside Riverside 92521 CA USA

**Keywords:** Bean pod mottle virus (BPMV), *Bradyrhizobium japonicum*, *Delftia acidovorans*, *Epilachna varivestis* (Mexican Bean Beetle), metabolomics, plant–microbe–insect interactions, soybean, transcriptomics

## Abstract

- Beneficial rhizobacteria and viral pathogens can both alter host plant phenotypes, yet little is known about how their simultaneous presence influences plant metabolism and species interactions. We investigated how two rhizobacteria, *Bradyrhizobium japonicum* and *Delftia acidovorans*, together with bean pod mottle virus (BPMV), shape soybean metabolism and interactions with the BPMV vector *Epilachna varivestis*.
- Using a multifactorial experimental design, we combined metabolomics, transcriptomics, and pathway analyses with behavioral assays to assess the impacts of rhizobacteria inoculation and BPMV infection on soybean physiology and *E. varivestis* feeding preferences.
- Beetle adults preferred feeding on rhizobia‐inoculated and virus‐infected plants, and larvae gained more weight on these hosts. Rhizobacteria inoculation and BPMV infection both increased primary metabolites (e.g. beta‐alanine, myo‐inositol, amino acids, and organic acids) while reducing secondary metabolites (e.g. kaempferol, rutin, and other flavonoids). Transcriptome analyses revealed shifts in defense‐ and metabolism‐related pathways, particularly under combined treatments.
- Our findings demonstrate that mutualistic and pathogenic symbionts reshape soybean metabolism in unique ways when they cocolonize the same host. These changes can alter symbiont fitness, as well as vector feeding behavior and performance in ways that enhance pathogen transmission.

## Introduction

Plant‐associated microbes can alter biochemical pathways within their hosts in ways that affect interactions among plants and other organisms. For example, mutualistic rhizobacteria associating with plant roots can modulate the production of primary and secondary plant metabolites, as well as volatile organic compounds (Schädler & Ballhorn, 2016). These changes typically alter plant phenotypes (i.e. physiological and metabolic traits that reflect microbial influences on host tissues) in ways that are beneficial to both the plant and its rhizobacteria by increasing plant growth, resistance against herbivores, or attractiveness to herbivore natural enemies (Pineda *et al*., 2013b; Pulido *et al*., 2019). Meanwhile, there is growing evidence that pathogenic microbes can similarly alter plant phenotypes through effects on metabolites, signaling pathways, and volatile emissions (Jiang *et al*., 2025). For pathogens that rely on arthropod vectors for transmission, such as plant viruses, these effects frequently enhance vector behaviors underlying pathogen acquisition or inoculation (Chesnais *et al*., 2025). The pathogen benefits from hijacking plant traits that act as cues for foraging vectors, while the plant experiences reductions in growth and fitness. In nature, plants frequently associate with both mutualistic and pathogenic symbionts simultaneously, with each of these players capable of influencing metabolic and defense pathways. Soils are rich with rhizobacteria that readily colonize plant roots, and virome studies reveal that plants are far more likely to be infected with a virus than not (Maclot *et al*., 2020). However, symbiont effects on plants are typically studied in isolation, leaving us with little information on the outcomes of simultaneous associations for symbiont fitness, plant phenotypes, and plant interactions with other species.

In this study, we investigate how engaging with multiple symbiotic partners shapes the metabolic pathways of soybean plants and their interactions with a pathogenic symbiont (the virus, bean pod mottle virus (BPMV, *Comovirus siliquae*)) and its herbivorous beetle vector, *Epilachna varivestis*. We aim to quantify and understand how and why co‐occurring symbiotic associations shape plant interactions with higher trophic levels through effects on insect behavior and virus epidemiological processes (e.g. transmission). These effects can be significant given that symbionts with different effects on plant health may target the same physiological and metabolic pathways in diverse ways, including those linked to nutrient status and plant defense (Pineda *et al*., 2013a). Outcomes may not be predictable from the individual effects of each player alone. For example, Van Dijk *et al*. (2022) found that the effects of soil microbial community composition on oak–aphid interactions were mediated by how soil microbes altered the severity of powdery mildew infections, which carried up to influence aphid populations. In the soybean system in which *E. varivestis* is both a key chewing herbivore and virus vector of BPMV, physiological changes that increase primary metabolites or suppress defensive compounds can influence beetle feeding behavior and performance. Beneficial rhizobacteria and viruses both have the potential to induce such changes through adaptations that enable symbiosis (Ledermann *et al*., 2021), but it is unclear how these effects manifest when symbionts colonize the same plant.

How plant viruses alter plant physiology in the context of mutualistic symbionts, such as rhizobacteria, is of considerable interest because plant viruses exploit plant resources systemically (rather than locally) and often cause reductions in plant fitness rather than enhancements. For arthropod‐borne viruses, the changes they induce as they infect and spread within plants must be balanced with their effects on plant interactions with virus vectors. Multiple lines of research show that virus effects on host‐plant phenotypes that influence insect vector behavior typically do so in ways that enhance virus transmission, suggesting selection against virus traits that inhibit or disrupt plant–vector interactions (Fereres & Moreno, 2009; Ingwell *et al*., 2012; Mauck *et al*., 2018; Ray & Casteel, 2022). For instance, infection of cucumber plants by cucurbit chlorotic yellows virus was reported to reduce defensive compounds, while increasing primary metabolites, such as amino acids, which led to enhanced feeding, and presumably virus acquisition, by the whitefly vector *Bemisia tabaci* (Zhang *et al*., 2022). Similarly, Luan *et al*. (2013) found that infection by tomato yellow leaf curl china virus triggered metabolite changes in tobacco plants, improving *B. tabaci* performance on this typically unfavorable host. Mauck *et al*. (2010) showed that cucumber mosaic virus infection increased aphid attraction to infected squash plants but promoted rapid dispersal of the vectors – conditions conducive to the transmission of this nonpersistently transmitted virus. Additionally, some studies have identified specific viral genes and gene variants responsible for transmission‐enhancing metabolic changes in host plants (Ray & Casteel, 2022).

While viruses may modify host phenotypes in ways that promote their own transmission at the expense of plant health, mutualistic soil‐dwelling rhizobacteria frequently alter host‐plant physiology in ways that enhance both bacterial and plant fitness (Berendsen *et al*., 2012; Zamioudis & Pieterse, 2012). These modifications extend beyond root tissue and can have a systematic influence on the entire plant, leading to changes in the levels of primary and secondary metabolites that influence multitrophic interactions among plants and other organisms (Pii *et al*., 2015; Grunseich *et al*., 2019). For example, Pulido *et al*. (2019) reported that cocolonization of soybean roots by a nitrogen‐fixing bacterium and a plant‐growth‐promoting rhizobacteria (PGPR) enhanced recruitment of parasitoid wasps to plants damaged by a specialist beetle herbivore. The current work builds upon that finding, further exploring the interactions between rhizobacteria, soybean plants, and insect herbivores, and elucidating additional facets of this multitrophic relationship.

New insight into the mechanisms underlying rhizobia effects on plant phenotypes has been enabled by large‐scale gene expression profiling approaches, and studies employing this approach have revealed the molecular interplay between *Bradyrhizobium japonicum* and soybean roots as the bacteria establish and proliferate within root nodules (Libault *et al*., 2009). For instance, *B. japonicum* colonization alters the expression of genes involved in canonical and antioxidant‐based plant defenses, as well as those involved in primary metabolism (Brechenmacher *et al*., 2008; Barros De Carvalho *et al*., 2013). Time‐course gene expression profiles further indicate that these effects are temporally dynamic and depend on the stage of the nodulation process (Libault *et al*., 2009). From a metabolomics perspective, *B. japonicum* nodulation has been shown to trigger rapid production of metabolites such as flavonoids, amino acids, and carbohydrates in soybean root hairs (Brechenmacher *et al*., 2010).

These studies demonstrate the power of transcriptomics and metabolomics in providing mechanistic insights into microbe‐induced effects on plant phenotypes. When employed in combination, these approaches are particularly valuable for uncovering plant trait changes relevant to multitrophic ecological interactions (Kang *et al*., 2018; Mhlongo *et al*., 2018; Castro‐Moretti *et al*., 2020). However, this integrated approach has been underutilized in microbe–plant interaction studies, especially in exploring cocolonization by multiple symbionts, despite the fact that such interactions are widespread in nature.

**Fig. 1:**
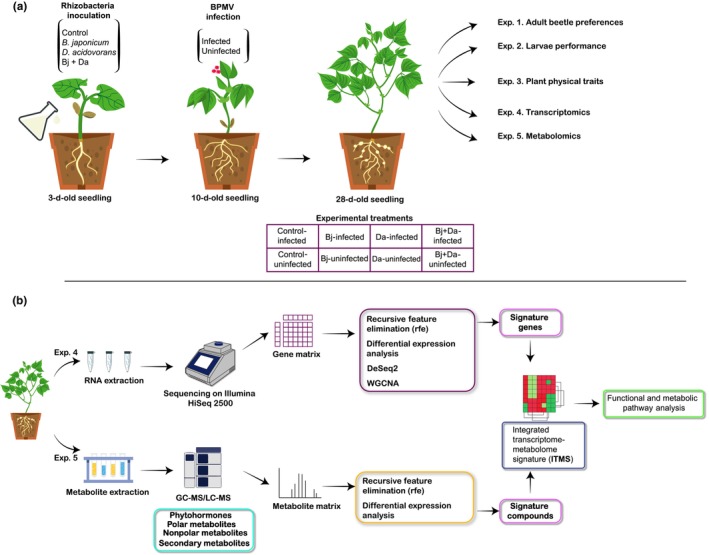
Experimental design overview. (a) Rhizobacteria inoculation and virus infection protocols were used to generate experimental treatment plants. This design was applied across various experiments detailed in the Materials and Methods section. (b) Integration of transcriptomics and metabolomics data. The Integrated Transcriptome‐Metabolome Signature was created by merging identified features. Pathway analysis and group comparisons were then performed to elucidate the impacted metabolic processes. BPMV, bean pod mottle virus; WGCNA, weighted correlation network analysis.

In this study, we address this research gap by examining changes in soybean plant physiology when in single and dual associations with two mutualistic microbes that differ in how they colonize plant roots (root‐nodulating *B. japonicum* and surface‐colonizing *Delftia acidovorans*) in the context of infection by BPMV. We use enrichment pathway analysis, combining metabolomics and transcriptomics, to identify signature genes and metabolites affected by these interactions (Fig. 1). We also quantify how interactive effects of symbionts on physiology carry up to alter plant growth, symbiont success, and feeding behavior of the BPMV vector (*E. varivestis*), ultimately enabling predictions about how rhizobacteria can influence virus acquisition. An important consideration in studies with nitrogen‐fixing rhizobia is the inherent link between symbiont mutualism and plant nutrition: Because exogenous nitrogen inhibits nodulation and biological nitrogen fixation (Jiang *et al*., 2020), experimental designs frequently compare inoculated plants grown without supplemental nitrogen to uninoculated plants receiving inorganic nitrogen (Kontopoulou *et al*., 2015; Allito *et al*., 2021; Ramula *et al*., 2023; Win *et al*., 2023). Although this approach unavoidably introduces some differences between rhizobial inoculation and nitrogen treatments, it generally reflects the relevant conditions found in natural settings, in which rhizobia symbiosis tends to occur in areas with low levels of soil nitrogen. We therefore adopted this design to maintain symbiosis, while recognizing its limitations for fully disentangling microbial vs nutrient effects. To link our molecular findings to ecological outcomes, we conducted behavioral assays to assess the feeding preferences and larval performance of the beetle vector *E. varivestis* in response to cocolonized plants. We hypothesized that cocolonization of beneficial rhizobacteria and BPMV induces distinct metabolic shifts in soybean, which in turn influence beetle feeding behavior, potentially affecting insect herbivory, the likelihood of virus transmission, and overall plant performance.

## Materials and Methods

### Rhizobacteria and BPMV culture conditions

We used a multifactorial design to investigate the effects of single, dual, and triple colonization by the PGPR *D. acidovorans* (den Dooren de Jong 1926) (Da), the nitrogen‐fixing root symbiont *B. japonicum* (Kirchner 1896) (Bj), and the virus pathogen BPMV (*C. siliquae*) on soybean plants (*Glycine max* (L.)) (Fig. 1; Supporting Information Table S1). This design allowed us to test various combinations of these treatments. The number of replicates varied depending on the specific experiments described later. Rhizobacteria cultures were isolated from a commercial product (BrettYoung) and kept for long‐term storage under sterile conditions at −80°C as 30% glycerol stocks. A BPMV strain that was collected in Ohio (US) was used to mechanically inoculate soybean plants in V1 (see Method S1, for details).

### Plant growth and experimental design

Soybean seeds (*G. max* cv Williams 82) were sterilized for 5 min in a 10% sodium hypochlorite solution and washed with copious amounts of ultrapure water. Seeds were germinated in a sterilized growing medium (Premier Pro‐mix without mycorrhiza, Griffin Supplies) autoclaved at 120°C for 40 min. Three‐day‐old seedlings were transplanted to individual 500‐ml pots containing the same growing medium and inoculated with 1 ml of the rhizobacterial suspension (adjusted to a cell density of 1 × 10^9^ CFU ml^−1^; see Method S1, for details). One week after rhizobacteria inoculation, seedlings were infected with BPMV or mock‐inoculated by rubbing leaves with a 0.1 M potassium phosphate buffer, following the multifactorial design shown in Fig. 1 and Table S1 (see Method S1, for inoculation details). Starting from the V1 stage, plants received 50 ml of a modified Hoagland's nutrient solution three times per week. Plants inoculated with Bj alone or in combination with Da received the same nutrient solution but without nitrogen, as nodulation is inhibited by the presence of nitrates in the soil (Ohyama *et al*., 2011; Carroll & Mathews, 2018). This strategy for supplementing nutrient solution to nonrhizobium inoculated plants has also been employed in our previous work (Pulido *et al*., 2019) and by other studies exploring similar systems (Barros De Carvalho *et al*., 2013; Kontopoulou *et al*., 2015; Martín‐Rodríguez *et al*., 2018; Allito *et al*., 2021; Ramula *et al*., 2023; Win *et al*., 2023). All plants were kept in an insect‐free growth chamber at 25°C (day), 23°C (night), a L16:D8 photoperiod and at 70% of relative humidity (see Method S2, for details). All measurements were taken at 3–4 wk of age (V4 stage), when nodules were fully developed and BPMV symptoms consistently expressed, providing a comparable physiological stage across treatments.

### Beetle colonies

Colonies of the Mexican bean beetle (*E. varivestis* Mulsant 1850 Coccinellidae), a significant herbivore of legumes and a known pest of soybeans, were initially provided by the New Jersey Department of Agriculture's Phillip Alampi Beneficial Insect Laboratory. *E. varivestis* was maintained on *Phaseolus vulgaris* plants in an incubator at 25°C and a L16:D8 photoperiod. One day before the bioassays, beetles were moved to the glasshouse in which the behavioral experiments took place to acclimate to the new conditions, and they were starved during this period.

### Adult beetle preferences and larval performance

We assessed larval performance using no‐choice assays across rhizobacteria and virus treatments. Ten newly hatched larvae were placed on each plant (*n* = 7) to ensure sufficient numbers throughout the assay, and larvae were allowed to feed until reaching the prepupal stage. When a plant was fully consumed, an additional plant of the same treatment and age was provided to maintain continuous access to food. After *c*. 2 wk, prepupae were removed, dried at 50°C for 2 d, and total dry weight was divided by the number of surviving larvae to obtain the average larval weight. Plants were enclosed in a transparent mesh net to prevent movement between plants. We analyzed average weight using ANOVA with rhizobacteria and virus infection as main factors.

We quantified adult feeding and foraging preferences using a dual‐choice assay in which two plants from different treatments were placed inside a fine‐mesh cage (Fig. S1; Video S1). During the test, five beetle adults were released within the tent and allowed to freely forage for 24 h. The combined activity (feeding or foraging behavior) of the five beetles within a tent was considered an individual replicate, as adult beetles do not tend to aggregate on individual host plants. Each choice test was repeated 12 times over the course of 8 d. After the test was completed, beetles were removed and the leaf damage was quantified using the Photoshop CC software. Differences in leaf damage across treatments were analyzed using ANOVA as described in the Statistical Analysis section. The following dual‐choice pairings were performed for uninfected and infected plants: (a). Bj vs Bj + Da, (b). Bj vs control, (c). Da vs Control, and (d). Bj + Da vs Control. Additionally, we tested the following mixed BPMV treatments: (e). Bj + Da‐BPMV vs control‐uninfected.

During the assay, we also recorded the beetle activity inside a tent using three DSLR cameras; the time‐lapse videos were analyzed using the Kinovea software, and beetle movement was converted into a plain text file with three spatial coordinates. We restricted the analyses to the space coordinates in which the plants were present. Each text file that represented the movement of five beetles inside a tent was considered a test replicate for a total of three replicates per paired‐choice test during the time of the experiment. This spatial analysis gives insight into foraging preferences of adult beetles and propensity of movements relevant for BPMV transmission. Beetle's foraging behavior was tested using exact binomial tests as detailed in the Statistical Analysis section.

### Plant physical traits

Before grinding the leaf tissue for metabolite extraction, samples were weighed, and total leaf biomass was recorded for each plant within a treatment. After harvesting the aboveground tissue, roots were thoroughly washed, and nodules were collected and placed separately inside paper envelopes, dried at 50°C for 48 h, and the total dry biomass of the nodules was measured for each plant (nodules are only present in plants inoculated with Bj, but control and Da plants were checked for possible cross‐contamination).

A TA‐XT2i Texture Analyzer (Texture Technologies Corp., Scarsdale, NY, USA) with a 3‐mm‐diameter spherical probe was used to measure the force (N) it takes to penetrate soybean leaf tissue using six replicates per treatment (rhizobacteria treatments crossed with BPMV‐infected plants, one leaf per plant was used). The test speed was 1 mm s^−1^. Leaf toughness is a physical trait that has been implicated as an important factor in direct defenses against herbivores (Marquis *et al*., 2002; Malishev & Sanson, 2015). In our model system, we tested whether beneficial rhizobacteria and virus infection played a role in promoting differences in leaf toughness and whether this trait might also explain the differences observed in feeding behavior.

Leaf toughness (Force/distance (g mm^−1^)), total shoot biomass, nodule biomass, and leaf toughness were analyzed separately in a two‐way ANOVA with rhizobacteria and virus infection as main factors. We used a regression analysis to assess the effect of nodulation on shoot biomass for each rhizobacteria and virus interaction.

### Phytohormone extraction and analysis

Plant tissue was harvested at 3–4 wk of age (V4 stage), weighed (100–150 mg), and flash‐frozen in liquid nitrogen (*n* = 10 total replications per treatment). For phytohormone extraction, we used a protocol modified from Schmelz *et al*. (2004). Briefly, frozen tissue was pulverized in a mill grinder (Spex Certiprep GenoGrinder 2000) under liquid nitrogen. The homogenized material received a mixture of 100 ng of internal, isotopic standards for *cis*‐OPDA, jasmonic acid (Tokyo Chemical Industry Co., Tokyo, Japan), salicylic acid (Campro Scientific, Berlin, Germany), abscisic acid, linoleic acid, and linolenic acid. Phytohormone extraction took place in an acidic buffer solution of water, propanol, and HCL. The organic phase was derivatized to methyl esters using trimethylsilyldiazomethane (Sigma‐Aldrich, St. Louis, MO, USA). The remaining solvent was evaporated, and vials were heated to 200°C while headspace was pulled across an adsorbent trap for 2 min (30 mg Super‐Q). Super‐Q traps were eluted with 150 μl dichloromethane, and the eluate was analyzed by GC‐MS (Agilent 6890) in single ion mode with isobutene chemical ionization using a HP‐1 column held at 40°C for 1 min then increased by 15°C min^−1^ to 300°C and held for 7 min. Final concentrations of free phytohormones were quantified in the MassHunter software relative to recovery of the internal standards. Amounts of phytohormones were corrected by the original fresh weight of the sample. Data were integrated with other metabolite information for multivariate analysis.

### Metabolite extraction and analysis

Immediately after harvesting the tissue for phytohormones, remnant leaf tissue per plant was collected in paper bags and frozen in liquid nitrogen (*n* = 10 total replications per treatment). All tissue was lyophilized for 72 h and ground to powder in a GenoGrinder. Once all the tissue per plant was homogenized, 10 ± 0.6 mg of dried tissue was weighed into a 4.0‐ml glass vial and processed with a multiphasic extraction protocol for polar, nonpolar, and secondary metabolites (Broeckling *et al*., 2005) (see Method S3, for details on sample and data processing).

We developed a Shiny app to provide an interactive and user‐friendly interface to visualize the effect of rhizobacteria and virus infection on soybean metabolites. The app allows users to select the metabolite of interest and compare its abundance between the two treatments. It is available at https://hannier.shinyapps.io/Metabolites_shinyApp/.

### RNA extraction

To assess the effect of rhizobacteria co‐inoculation and BPMV on soybean gene expression, we harvested plant leaf tissue from a separate set of untouched undamaged plants at the V4 stage. Tissue sections with weights of 100–200 mg were obtained, and the tissue was flash‐frozen in liquid nitrogen. The homogenized material was extracted for total RNA using a mirVana miRNA isolation kit (Ambion, Austin, TX, USA), with modifications suggested by Peña‐Llopis & Brugarolas (2013). We combined equal amounts of three individual total RNA samples with similar quality and concentration to obtain a pooled replicate sample for each treatment, resulting in a total of five biological pooled replicates per treatment (*n* = 40). We used a Nanodrop Spectrophotometer (NanoDrop Technologies, Wilmington, DE, USA) to determine RNA concentration and assessed RNA quality using the RNA Integrity Number (RIN) method, which is widely used for RNA quality control (Mueller *et al*., 2004), in an Agilent 2100 Bioanalyzer (Agilent Technologies, Santa Clara, CA, USA).

### RNA‐Seq library construction and sequencing

To prepare the barcoded libraries for the 40 RNA samples, 100 ng of total RNA was used with a TruSeq Stranded mRNA Library Kit, following the manufacturer's instructions. The concentration of each library was determined by quantitative polymerase chain reaction (qPCR), and an equimolar pool of the 40 barcoded libraries was created. Sequencing was performed on an Illumina HiSeq 2500 in Rapid Run mode, using 150‐nucleotide single‐read sequencing at the Pennsylvania State University core facility. We conducted three consecutive sequencing runs to achieve the desired number of reads per sample. Raw sequence reads are available from the National Center for Biotechnology Information (NCBI) read archive under the accession no. GSE244001.

### Mapping and processing of RNA‐seq reads

After base calling, adapter content and poor‐quality reads were removed from the samples using *Trimmomatic* (Bolger *et al*., 2014). The samples were then mapped to the reference genome of Glycine max v.2.0 using *tophat2*, utilizing genome and annotation files obtained from GenBank (ftp://ftp.ncbi.nlm.nih.gov/genomes/Glycine_max/Assembled_chromosomes/seq/). Coverage files and a count matrix were generated with *bedtools*. The rlog‐normalized feature counts matrix was then utilized for further statistical analysis, gene enrichment analysis, and pathway mapping.

### Transcriptomics and metabolomics data integration

To identify the metabolic processes affected by the interaction between beneficial rhizobacteria and BPMV infection on soybean, we integrated transcriptomic and metabolomic data using a functional and metabolic pathway analysis, as depicted in the flowchart (Fig. 1).

To simplify comparisons and account for similarities in gene expression and metabolite abundance, we grouped all uninfected rhizobacteria treatments (Bj, Bj + Da, and Da) into one category (‘all Bacteria‐uninfected’) and all BPMV‐infected rhizobacteria treatments into another (‘all Bacteria‐infected’). This allowed us to compare these groups with the control‐uninfected treatment and identify significant differences across analyses. See Methods S4, for details.

### Data analysis

All analyses were conducted in R v.4.1.0 (R Core Team, 2021). For larval performance, adult feeding assays, plant physical traits, shoot biomass, and nodule biomass, treatment effects were evaluated using two‐way ANOVA with rhizobacteria and virus infection as fixed factors. For the nodule–biomass relationship, simple linear regression models were fitted separately for each rhizobacteria × virus combination. Model assumptions were assessed using residual plots, Shapiro–Wilk tests for normality, and Levene's or Breusch–Pagan tests for homogeneity of variance. No major deviations were detected.

Analyses for transcriptomics and metabolomics datasets are detailed in Method S4.

## Results

### Effects on beetle performance

#### Both rhizobacteria inoculation and BPMV infection affect foraging and feeding preferences of adult beetles

**Fig. 2:**
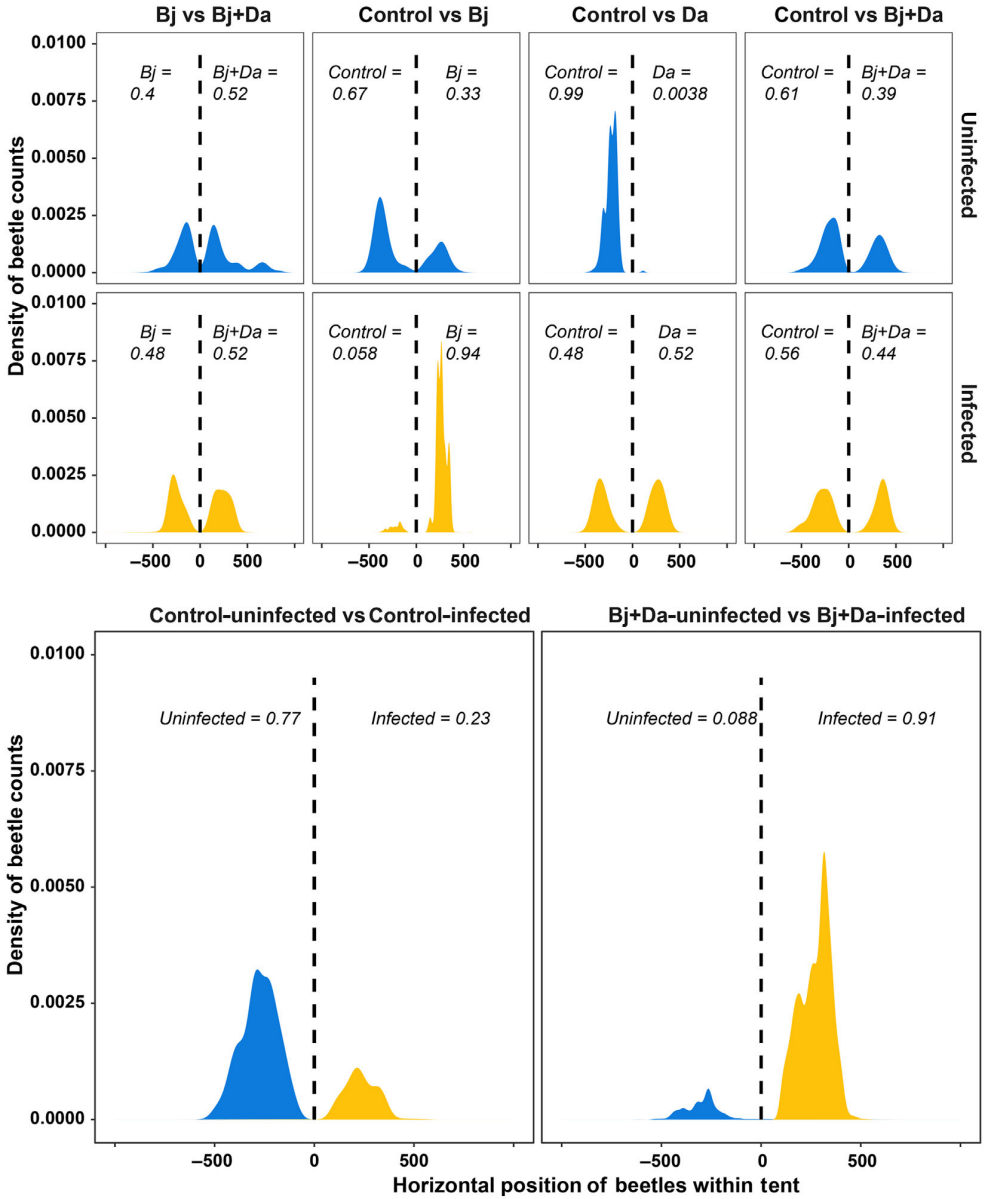
Adult foraging behavior in a dual‐choice test. Foraging behavior of beetles when rhizobacteria‐inoculated plants were either uninfected (upper) or infected with bean pod mottle virus (middle). The lower panel compares beetle foraging behavior between infected and uninfected plants for both Bj + Da‐inoculated plants and control plants. The *x*‐axis represents the horizontal position of the beetles within the tent, and the density plots represent the time beetles spent at each plant. The vertical dashed lines indicate the midpoint of the tent. Each choice test was repeated 12 times with five different beetles per trial. Group probabilities are shown within the plots, and the presence of beetles on each side of the tent was compared using an exact binomial test. Uninfected treatments are represented in blue, and infected treatments are shown in orange. Bj, *Bradyrhizobium japonicum*; Da, *Delftia acidovorans*.

Adult beetles exhibited clear patterns of preference that depended on both rhizobacterial treatment and BPMV infection status. In the dual‐choice foraging assay involving choices among uninfected plants with different rhizobacteria treatments, beetles spent more time on control plants over rhizobacteria‐inoculated plants. In trials involving choices between BPMV‐infected plants with different rhizobacteria treatments, the beetles preferred foraging on Bj‐ and Da‐inoculated plants (Fig. 2; Table S2; *P* < 0.001). Overall, these patterns indicate that rhizobacteria effects on beetle foraging differ between BPMV‐infected and uninfected plants.

**Fig. 3:**
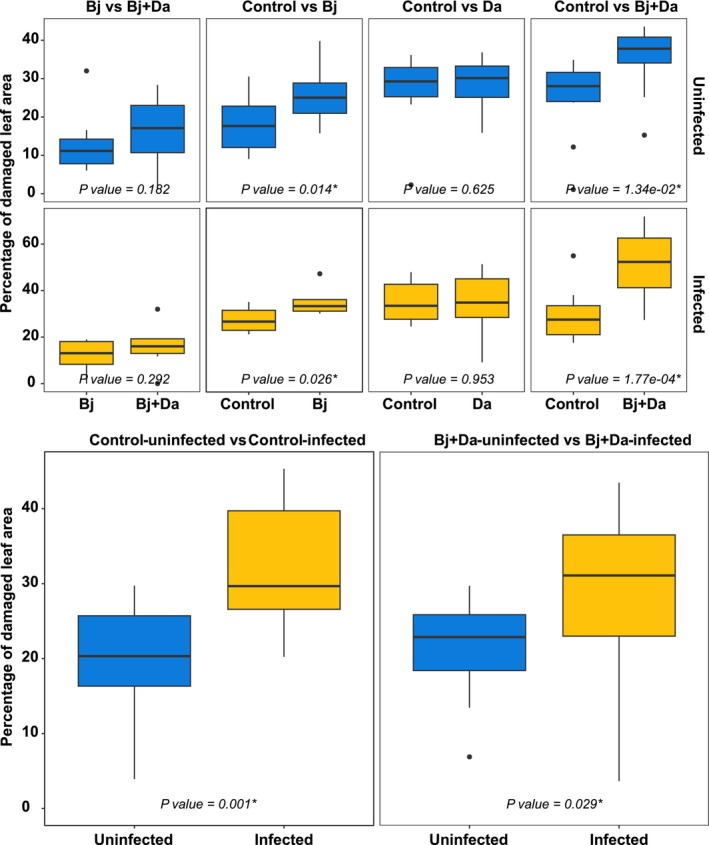
Adult feeding behavior in a dual‐choice test. Feeding behavior of beetles when rhizobacteria‐inoculated plants were either uninfected (upper) or infected with BPMV (middle). The lower panels compare beetle feeding behavior between infected and uninfected plants for both Bj + Da‐inoculated plants and control plants. The *y*‐axis represents the percentage of damaged leaf area. Uninfected treatments are represented in blue, while infected treatments are shown in orange. Bj, *Bradyrhizobium japonicum*; Da, *Delftia acidovorans*.

Beetles also preferred feeding on BPMV‐infected Bj + Da plants compared with their uninfected counterparts, but in the absence of rhizobacteria, they fed more on uninfected than infected plants (Fig. 2; *P* < 0.001). These responses further demonstrate that rhizobacteria influence adult feeding behavior in relation to BPMV infection status, with the presence of cocolonizing rhizobacteria leading to more consumption of virus‐infected tissue (Table S2). Rhizobacteria alone also modified beetle feeding patterns; across feeding assays, beetles consistently consumed more foliage from Bj‐inoculated plants than from controls, regardless of infection status (Fig. 3; Table S3; *P* < 0.001). When offered plants with identical rhizobacterial treatments, beetles preferred BPMV‐infected plants over uninfected plants (Fig. 3; Table S3; *P* < 0.001).

#### Both rhizobacteria inoculation and BPMV infection increase larval biomass

**Fig. 4:**
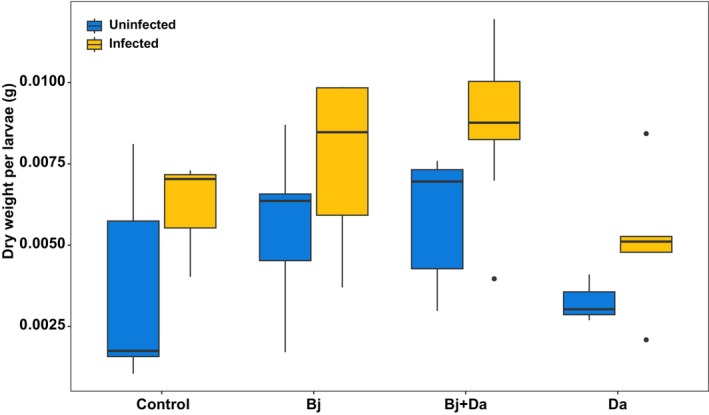
Larvae weight gain. Boxplots show the dry weight (g) gained per larva in a nonchoice assay. Recently hatched larvae were placed on each plant and weighed when they reached the prepupal stage. Ten larvae were placed on each plant (*n* = 7). Dry weight was analyzed using a two‐way ANOVA with rhizobacteria and virus infection as main factors. Uninfected treatments are represented in blue, while infected treatments are shown in orange. Bj, *Bradyrhizobium japonicum*; Da, *Delftia acidovorans*.

In our no‐choice assay, larvae gained more weight on BPMV‐infected plants and also performed better on certain rhizobacteria‐treated plants, although there was no significant interaction between BPMV infection and rhizobacteria (Fig. 4; Table S4). Post hoc comparisons showed that larvae feeding on Bj + Da plants gained more weight than those feeding on control or Da‐inoculated plants (Table S4).

### Effects on plant physical traits

#### BPMV infection influences nodulation and shoot biomass but not leaf toughness

**Fig. 5:**
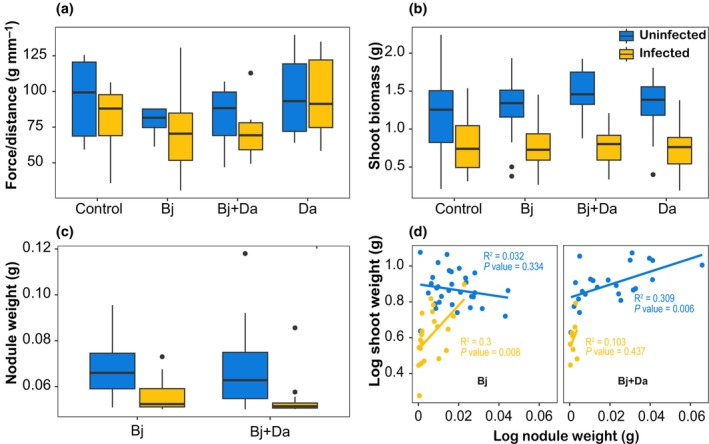
Plant physical traits. (a) Leaf toughness, measured as the force required to penetrate the leaf tissue (force/distance in g mm^−1^). (b) Shoot biomass for different rhizobacteria and virus treatments. (c) Effect of bean pod mottle virus infection on nodulation and *Delftia acidovorans* on nodulation. (d) Regression analysis between nodule weight and shoot biomass for Bj and Bj + Da treatments. Uninfected treatments are represented in blue, while infected treatments are shown in orange. Bj, *Bradyrhizobium japonicum*; Da, *Delftia acidovorans*. In boxplots (a–c), center lines indicate medians, boxes represent interquartile ranges, and points denote outliers.

We did not detect significant effects of rhizobacteria inoculation or BPMV infection on leaf toughness (Fig. 5a; Table S5; *P* = 0.293). Similarly, while BPMV‐infected plants had lower shoot biomass than uninfected plants, differences among rhizobacteria treatments were not significant (Fig. 5b; Table S6; *P* = 0.149). By contrast, nodule biomass was significantly reduced by BPMV infection in both rhizobacteria treatments (Bj and Bj + Da plants) (Fig. 5c; Table S7; *P* < 0.001). Regression analyses examining the relationship between nodule weight and shoot biomass revealed that two models showed a significant positive association: Bj‐infected (*F*_1,20_ = 8.55, *P* = 0.008, *R* ^2^ = 0.30) and Bj + Da‐uninfected (*F*_1,21_ = 9.39, *P* = 0.005, *R* ^2^ = 0.30). The remaining models did not show significant relationships: Bj‐uninfected (*F*_1,29_ = 0.96, *P* = 0.33, *R* ^2^ = 0.03) and Bj + Da‐infected (*F*_1,6_ = 0.69, *P* = 0.43, *R* ^2^ = 0.10) (Fig. 5d; Table S8). These results indicate that dual inoculation does not increase mean shoot biomass but rather modulates the efficiency with which nodulation translates into shoot growth.

### Effects on plant metabolomics

#### Both rhizobacteria inoculation and BPMV infection increase primary metabolite levels and reduce secondary metabolites

**Fig. 6:**
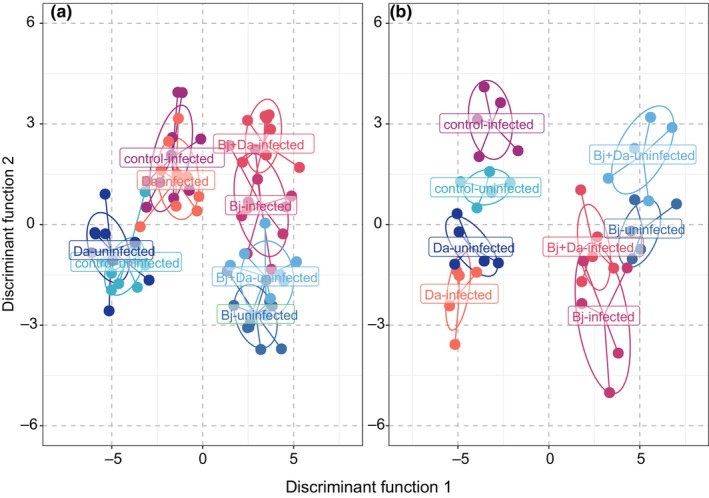
Group Separation Using Discriminant Analysis of Principal Components. (a) Clustering of samples based on metabolite abundance data, plotted along Discriminant Function 1 and Discriminant Function 2. Each point represents a sample, color‐coded by treatment. Ellipses indicate clusters, highlighting the separation between treatments. (b) Clustering of samples based on RNA‐Seq normalized counts data. Uninfected treatments are represented in shades of green, while infected treatments are shown in shades of red. Bj, *Bradyrhizobium japonicum*; Da, *Delftia acidovorans*.

**Fig. 7:**
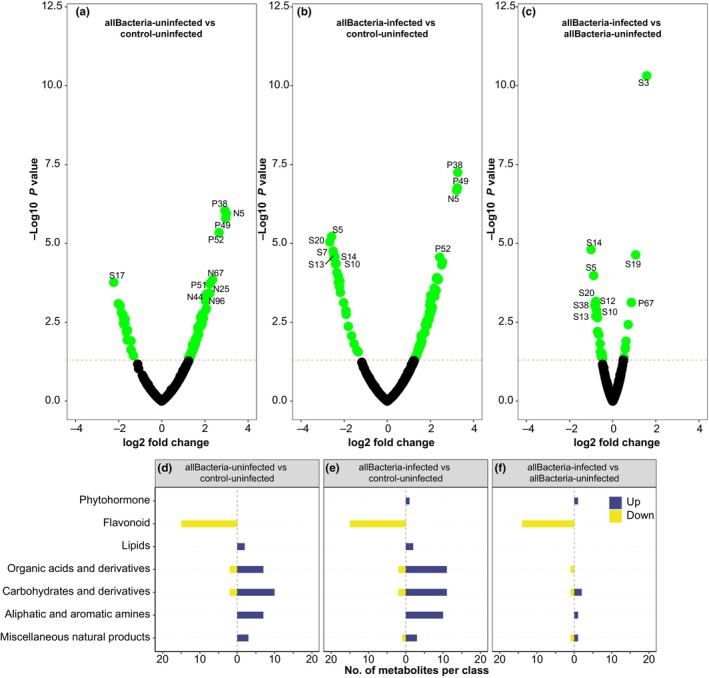
Modulation of metabolite profiles by rhizobacteria colonization and bean pod mottle virus (BPMV) infection in soybean plants. The upper panel shows differentially accumulated metabolites across three contrasts: (a) All bacteria‐uninfected treatments compared with control‐uninfected. (b) All bacteria, BPMV‐infected treatments compared to control‐uninfected. (c) All bacteria, BPMV‐infected treatments compared to all bacteria‐uninfected treatments. Significant metabolites (*P*‐value <0.05) are highlighted as green dots, while nonsignificant metabolites (*P*‐value >0.05) are shown as black dots. The lower panel displays the number of differentially accumulated metabolites for the three contrasts, categorized as follows: (1) organic acids and derivatives: carboxylic acid, fatty acid, organic acid, amide, peptide. (2) Carbohydrates and derivatives: carbohydrate, monosaccharide, sugar alcohol, glycoside. (3) Lipids: fat, fatty alcohol, lipid. (4) Aliphatic and aromatic amines: amine, amino acid, indole. (5) Miscellaneous natural products: acyloin, alcohol, aldehyde, glucosinolate, inorganic acid, pyranone, terpenoid, tetracycline. Compound IDs are listed in Supporting Information Table S9. all Bacteria = *Bradyrhizobium japonicum*, *B. japonicum + Delftia acidovorans*, and *D. acidovorans*.

Discriminant analysis of principal components (DAPC) analysis revealed clear separation between uninfected and BPMV‐infected samples, with Bj inoculation also strongly influencing group separation (Fig. 6a). Linear models identified 88 metabolites with significant changes due to rhizobacteria inoculation (allBacteria = combined Bj, Bj + Da, and Da treatments) in uninfected plants (65 accumulated and 23 depleted) (Fig. 7a). This number increased to 99 metabolites in plants that were both rhizobacteria‐inoculated and BPMV‐infected (75 accumulated and 24 depleted) (Fig. 7b). Specifically, in rhizobacteria‐inoculated BPMV‐infected plants, 10 metabolites (mainly carbohydrates and amino acids) were significantly accumulated, while 19 metabolites (mostly flavonoids) were significantly depleted (Fig. 7c).

Comparisons between individual rhizobacteria treatments in uninfected vs BPMV‐infected plants (Figs S2, S3; Dataset S1) and between mixed uninfected and BPMV‐infected treatments (Fig. S4; Dataset S1) revealed 71 metabolites significantly changed in Bj + Da‐inoculated uninfected plants (48 accumulated and 23 depleted) and 69 in Bj + Da‐infected plants (27 accumulated and 42 depleted). BPMV infection altered 13 metabolites in Bj + Da plants (9 accumulated and 4 depleted) and 22 metabolites in control plants (21 accumulated and 1 depleted). Notably, both BPMV infection and rhizobacteria inoculation reduced flavonoid production and increased organic acids, carbohydrates, and amino acids.

Furthermore, rhizobacteria inoculation of uninfected plants led to a more than fivefold increase in several metabolites, including beta‐alanine, glyceric acid, and pyruvic acid, compared with control plants. The presence of both rhizobacteria and BPMV further increased certain metabolites while reducing flavonoid levels (Table 1; Figs 7c, S4; Dataset S1).

**Table 1:** Metabolite changes upon rhizobacteria inoculation and bean pod mottle virus (BPMV) infection.

Metabolites	Effect of rhizobacteria inoculation (uninfected plants)	Effect of rhizobacteria inoculation + BPMV infection	Effect of BPMV infection (no rhizobacteria)
Beta‐alanine (P49)	↑ >5‐fold	↑	↑ >3‐fold
Serine (P41)	↑ 4‐fold	↑ >3.7‐fold	
Glyceric acid (P38)	↑ 6‐fold	↑ 3.5‐fold	↑ >3‐fold
Pyruvic acid (P52)	↑ >5‐fold	↑	↑ >3‐fold
Glyceryl‐glycoside (N68)	↑ 5.0‐fold	↑ 3.8‐fold	–
Myo‐inositol (N59)	–	↑	↑ >3‐fold
Luteolin (S20)	–	↓	↓
Eriocitrin (S5)	–	↓	↓
Formononetin 7‐O‐rutinoside (S7)	–	↓	–
Cyanidin 3‐glucogalactoside (S13)	–	↓	–
Kaempferol (S6)	–	↓	↓
Rutin (S10)	↓ –3.4‐fold	↓ –6.3‐fold	–
Pelargonidin 3‐galactoside‐5‐glucoside (S14)	–	↓	↓
Rubrofusarin (S9)	–	↓	–
Cyanidin 3‐rhamnoside 5‐glucoside (S12)	–	↓	↓
Tuberonic acid glucoside (S19)	–	↑ >2‐fold	–
L‐beta‐aspartyl‐L‐phenylalanine (S3)	–	↑ >2‐fold	↑ >3‐fold
Aspartic acid (P51)	–	–	↑ >3‐fold
4‐aminobutanoic acid (P58)	–	–	↑ >3‐fold
Galactaric acid (P145)	–	–	↑ >3‐fold
D‐allose (P127)	–	–	↑ >3‐fold
L‐rhamnose (N23)	–	–	↑ >3‐fold
Shikimic acid (P95)	–	–	↑ >3‐fold

The compound code used in the graphs is provided in parentheses after each compound name. Full numerical results, including fold changes, *P*‐values, and variability, are provided in Dataset S1. Compound IDs are listed in Supporting Information Table S9.

↑ represents an increase, ↓ represents a decrease, − represents no significant change.

#### BPMV infection decreases the levels of some rhizobacteria‐induced metabolites

BPMV infection of rhizobacteria‐inoculated plants led to a reduced concentration of some metabolites that were previously accumulated. Notably, metabolites such as glyceric acid, glyceryl‐glycoside, and serine showed decreased fold changes in BPMV‐infected plants compared with their uninfected rhizobacteria‐inoculated counterparts. Additionally, the depletion of flavonoid compounds was more pronounced following BPMV infection (Table 1).

Metabolite compounds were grouped based on their chemical properties. Our analysis confirmed that both BPMV infection and rhizobacteria inoculation caused significant decreases in flavonoid compounds, while increasing levels of organic acids, carbohydrates, and amino acids compared with control‐uninfected plants (Fig. 7d,e). Interestingly, BPMV infection of rhizobacteria‐inoculated plants also led to a reduction in the abundance of primary metabolites, including lipids, organic acids, carbohydrates, and amino acids (Fig. 7f).

### Effects on plant transcriptomes

#### BPMV and rhizobacteria treatments shift soybean gene expression patterns

We analyzed changes in soybean gene expression profiles induced by BPMV infection and rhizobacteria inoculation using RNA‐Seq followed by DAPC (see the Materials and Methods section). As shown in Fig. 6b, both rhizobacteria inoculation (Bj, Da, and Bj + Da) and BPMV infection strongly affected soybean gene expression.

To gain detailed insights, we identified signature genes – sets of genes whose expression changed coordinately under different conditions – based on differential expression (DESeq), importance (RFE), and gene‐module membership (weighted correlation network analysis, WGCNA). We performed multiple pairwise comparisons, assessing individual, and combined effects of rhizobacteria and virus infection (Fig. S5; Table S10; Datasets S1–, S4). We found a high number of signature genes upon inoculation with Bj and Bj + Da, and fewer with Da, indicating that the nitrogen‐fixing symbiont Bj has a stronger effect on soybean gene expression compared with the growth‐promoting Da. Additionally, fewer rhizobacteria‐induced signature genes were observed in control plants than in BPMV‐infected plants (Fig. S6; Dataset S2). For instance, 90 upregulated and 21 downregulated genes were identified in uninfected Bj + Da vs Bj plants, compared with 860 upregulated and 1530 downregulated genes in BPMV‐infected plants (Fig. S5; Dataset S2), highlighting the strong impact of BPMV on soybean gene regulation and microbial responses.

**Fig. 8:**
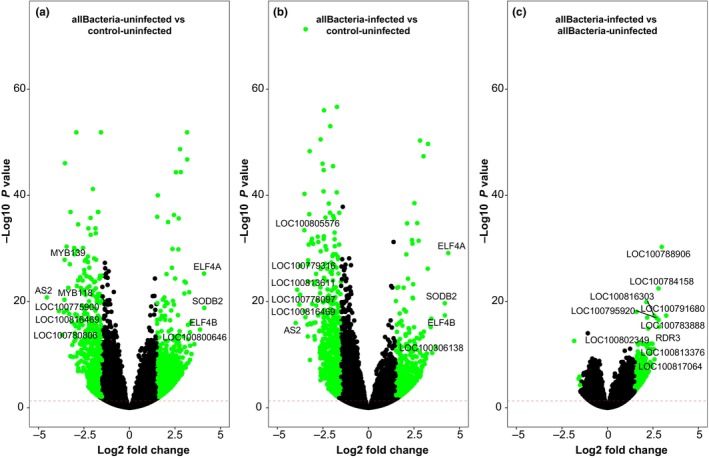
Volcano plots depicting gene expression profiles in soybean plants under rhizobacteria colonization and bean pod mottle virus (BPMV) infection. Signature genes identified through differential expression (DESeq) analysis, recursive feature elimination, and weighted correlation network analysis (WGCNA) are labeled. Significant genes (log_2_ fold change > 1.5 and adjusted *P*‐value <0.01) are highlighted in green, while nonsignificant genes are shown in black. The *y*‐axis represents −log_10_ *P*‐values, and the *x*‐axis shows log_2_ fold changes for each gene. (a) Gene expression in all uninfected rhizobacteria treatments compared to control‐uninfected. (b) Gene expression in all BPMV‐infected rhizobacteria treatments compared to control‐uninfected. (c) Gene expression in all BPMV‐infected rhizobacteria treatments compared to all uninfected rhizobacteria treatments. all Bacteria = *Bradyrhizobium japonicum*, *B. japonicum + Delftia acidovorans*, and *D. acidovorans*.

Fig. 8 displays the top 10 signature genes induced by rhizobacteria inoculation (All Bacteria), BPMV infection, or their combination. Among these, iron‐superoxide dismutase (SODB2), EARLY FLOWERING 4a (ELF4A), and ELF4B were upregulated in both uninfected and BPMV‐infected plants (Figs 7b, 8a). BPMV infection of rhizobacteria‐inoculated plants resulted in upregulation of all top 10 signature genes: LOC100791680 (NAC domain‐containing protein 73), LOC100788906 (AAA‐ATPase At3g50940), RDR3 (RNA‐dependent RNA polymerase), LOC100783888 (NAC domain‐containing protein 73), LOC100784158 (probable WRKY transcription factor 23‐like), LOC100795920 (NAC domain‐containing protein 73), LOC100817064 (pumilio homolog 2), LOC100816303 (TMV resistance protein N‐like), LOC100813376 (NAC domain‐containing protein 87), and LOC100802349 (ureide permease 1) (Fig. 8c). Figs S6–S8 and Dataset S2 present the number of signature genes (up‐ vs downregulated) in each treatment comparison, reinforcing the magnitude of transcriptomic differences we described.

#### Integration of signature genes and metabolites reveals changes in defense, energy, and nutrition pathways

**Fig. 9:**
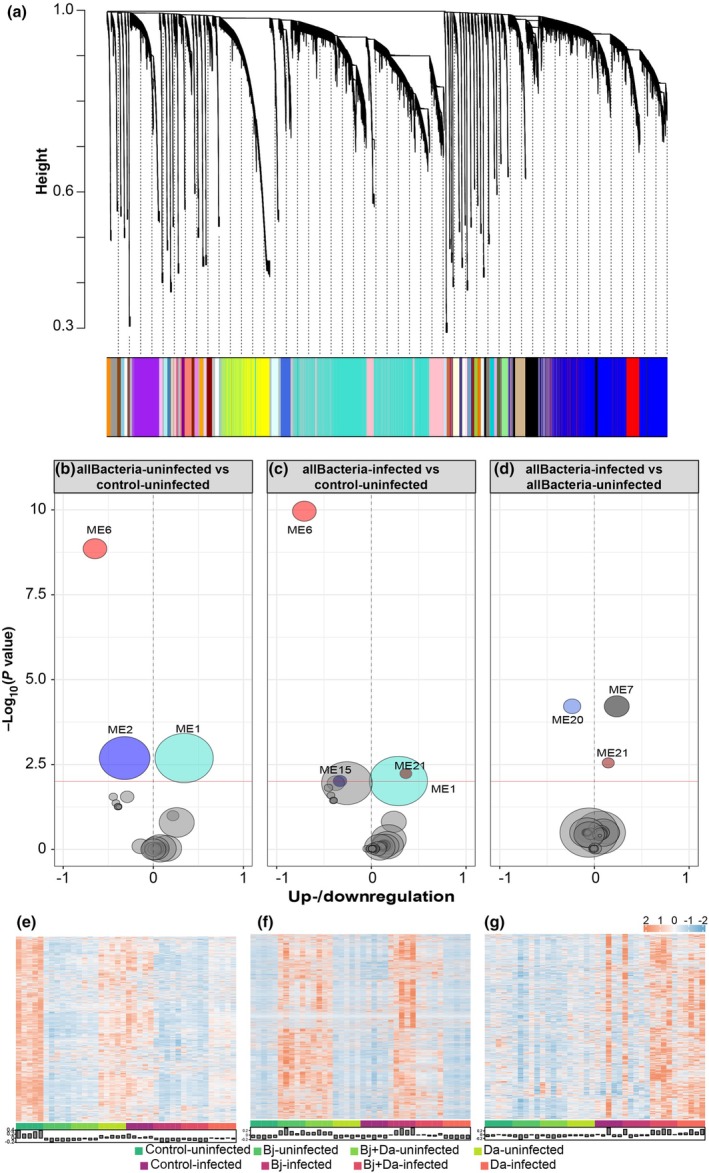
Weighted gene co‐expression network analysis (WGCNA) analysis of whole leaf transcriptomics on soybean plants after rhizobacteria inoculation and bean pod mottle virus (BPMV) infection. (a) Transcripts were grouped into 49 modules based on co‐expression patterns. The dendrogram shows the hierarchical relationships and dissimilarities between modules, with branch height indicating the degree of dissimilarity. Each module is assigned a unique color by WGCNA, displayed in the color strip at the bottom panel. Module sizes vary from 127 to 9735 genes, with size proportional to the number of genes. Middle panel: significant modules detected by WGCNA across three contrasts. (b) All uninfected rhizobacteria treatments vs control‐uninfected. (c) All BPMV‐infected rhizobacteria treatments vs control‐uninfected. (d) All BPMV‐infected rhizobacteria treatments vs all uninfected rhizobacteria treatments. Significant modules (adjusted *P*‐value <0.01) are shown in their respective WGCNA colors, while nonsignificant modules are depicted in gray. Module size is proportional to the number of genes assigned. Bottom panel: gene expression profiles for the most significant modules: (e) Module 6, (f) Module 1, (g) Module 7. Uninfected treatments are shaded in green, and BPMV‐infected samples in red. Bar plots represent module eigengenes, summarizing gene expression patterns for each sample in each module. all Bacteria = *Bradyrhizobium japonicum*, *B. japonicum + Delftia acidovorans*, and *D. acidovorans*.

Using the full transcriptomics dataset, we identified 48 co‐expression modules (MEs) containing between 127 and 9735 genes, totaling 41 540 genes (Fig. 9a; Dataset S3). Two modules, ME6 and ME1, were significantly regulated in response to rhizobacteria inoculation regardless of BPMV infection, with ME6 genes strongly downregulated and ME1 genes upregulated (Fig. 9b,c; Dataset S4). Conversely, two other modules, ME2 and ME15, were significantly downregulated in rhizobacteria‐inoculated plants either in the absence of BPMV (ME2) or in its presence (ME15) (Fig. 9b,c; Dataset S4). Additionally, ME21 was significantly upregulated by BPMV infection, independent of rhizobacteria inoculation (Figs 9c,d, 8e; Dataset S4), while modules ME20 and ME7 were regulated by BPMV infection only in the presence of rhizobacteria, with ME7 genes upregulated and ME20 genes downregulated (Fig. 9b,c; Dataset S4). The top six genes for each of these modules are listed in Table S11, and the expression profiles of key modules ME6, ME1, and ME7 are shown in Fig. 9e–g.

**Fig. 10:**
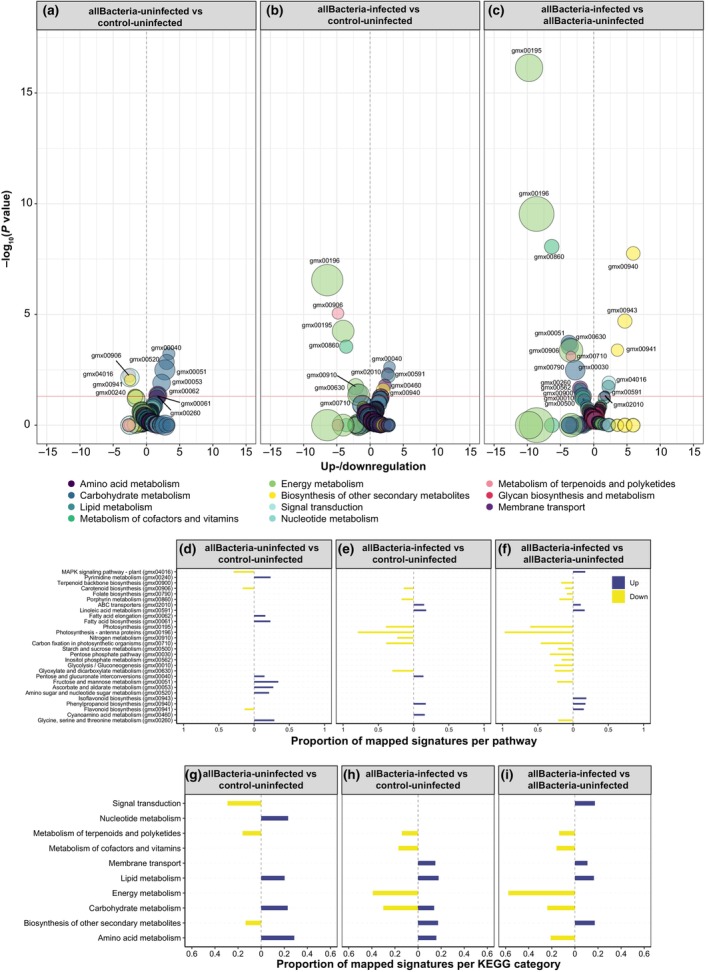
Gene‐set enrichment (GAGE) and KEGG pathway analysis of the Integrated Transcriptome‐Metabolome Signature. The top panel shows the distribution of KEGG pathways across three contrasts. (a) All uninfected rhizobacteria treatments vs control‐uninfected. (b) All BPMV‐infected rhizobacteria treatments vs control‐uninfected. (c) All BPMV‐infected rhizobacteria treatments vs all uninfected rhizobacteria treatments. Significantly enriched pathways identified through GAGE (*P*‐value <0.05) are labeled by their KEGG pathway ID. Colored bubbles indicate the KEGG category for each pathway, and bubble size represents the proportion of signature genes and metabolites mapped to that pathway. Pathways are color‐coded based on their KEGG category. The middle panel shows the proportion of signature genes and metabolites mapped to significant KEGG pathways for each contrast. The bottom panel illustrates the proportion of mapped signatures within KEGG categories for the same contrasts. all Bacteria = *Bradyrhizobium japonicum*, *B. japonicum + Delftia acidovorans*, and *D. acidovorans*.

To gain further insights, we performed a gene/metabolite enrichment analysis (GAGE) to identify significantly perturbed Kyoto encyclopedia of genes and genomes (KEGG) pathways in response to these treatments (Fig. 10; Dataset S5). For each pairwise treatment comparison, KEGG pathway maps were generated for all significantly upregulated and downregulated pathways; these are provided in Dataset S6. Rhizobacteria inoculation in uninfected plants upregulated pathways related to carbohydrate, lipid, nucleotide, and amino acid metabolism, while downregulating carotenoid biosynthesis (gmx00906), mitogen‐activated protein kinase (MAPK) signaling (gmx04016), and flavonoid biosynthesis (gmx00941) (Fig. 10a,d,g; Datasets S5–, S7). Interestingly, in rhizobacteria‐inoculated, BPMV‐infected plants, there was a reduction in the upregulation of carbohydrate metabolism pathways and a strong downregulation of pathways related to energy metabolism, nitrogen metabolism, cofactors, vitamins, and terpenoids and polyketides (Fig. 10b,e,h; Dataset S5). Further analysis revealed that BPMV infection significantly perturbed pathways associated with secondary metabolite biosynthesis, signal transduction, membrane transport, and energy, carbohydrate, and amino acid metabolism in rhizobacteria‐inoculated plants (Bj, Bj + Da, and Da). Notably, phenylpropanoid biosynthesis (gmx00940), isoflavonoid biosynthesis (gmx00943), flavonoid biosynthesis (gmx00941), and MAPK signaling (gmx04016) were the most significantly upregulated pathways, indicating their enrichment due to BPMV infection (Dataset S7C). By contrast, photosynthesis (gmx00195), photosynthesis antenna proteins (gmx00196), porphyrin metabolism (gmx00860), fructose and mannose metabolism (gmx00051), and glyoxylate and dicarboxylate metabolism (gmx00630) were the most significantly downregulated pathways, highlighting a strong impact on energy and carbohydrate metabolism (Fig. 10c,f,i; Datasets S5, S7C).

## Discussion

Mutualistic rhizobacteria and viral pathogens both modify plant phenotypes in ways that change interactions with herbivores (Pulido *et al*., 2019). To understand how cocolonization by these two symbiont types affects species interactions, symbiont fitness, and the likelihood of virus acquisition, we combined vector behavior and performance assays with metabolomic and transcriptomic analyses. We found that beetle vector foraging and feeding preferences for BPMV‐infected plants increased when infected plants were cocolonized with both types of rhizobacteria. Beetles also grew larger on plants with both BPMV infection and colonization by *B. japonicum* and *D. acidovorans*. These results indicate that beetle behaviors associated with virus acquisition and propagation to new hosts are enhanced when plants associate with certain rhizobacteria combinations. Across treatments, simultaneous colonization by *B. japonicum* and *D. acidovorans* consistently altered primary and secondary metabolism, increasing amino and fatty acids while depleting flavonoids in both virus‐free uninfected plants and BPMV‐infected plants. Together, these patterns suggest that microbially induced changes in plant quality may influence vector movement in ways relevant to virus transmission.

### Rhizobacteria and BPMV enhance beetle feeding and growth

Separate foraging and feeding experiments revealed divergence in beetle movement and tissue consumption patterns that have implications for BPMV transmission. In dual‐choice feeding assays, beetles fed more on BPMV‐infected plants regardless of rhizobacteria treatment, as in our previous work (Peñaflor *et al*., 2016). However, in foraging assays, beetles spent more time on uninfected plants in the absence of rhizobacteria (Fig. 2). This outcome is consistent with earlier findings that BPMV reduces volatile emissions in soybean (Pulido *et al*., 2019), which can decrease attractiveness to herbivores and stimulate movement toward uninfected plants. However, cocolonization with both rhizobacteria species shifted this pattern: Beetles fed the most on BPMV‐infected plants with Bj + Da colonization and also spent more time foraging on plants with all three symbionts (Figs 2, 3). Rhizobacteria may modulate this preference by restoring or enhancing volatile emissions even when plants are infected with BPMV (as seen in our prior work (Pulido *et al*., 2019)), ultimately increasing vector contacts. Changes in beetle preferences for BPMV‐infected plants in the presence of rhizobacteria may favor greater acquisition and retention of BPMV virions from infected tissue in the beetle foregut (Gergerich, 2001). If beetles ultimately disperse from these plants, this increased acquisition may also lead to greater virus spread (Madden *et al*., 2000).

While adult beetle preferences for BPMV‐infected plants were strongly influenced by cocolonizing rhizobacteria, larval responses to plants under no‐choice scenarios were more consistently driven by BPMV infection. Larvae feeding on BPMV‐infected plants gained more weight than those feeding on uninfected plants regardless of rhizobacteria treatment (Fig. 4). Prior work also found increased performance of *E. varivestis* on BPMV‐infected *P. vulgaris* (Musser *et al*., 2003). Faster larval growth may confer fitness benefits to the beetle by reducing the period when it is vulnerable to predation or parasitism while also promoting virus spread, as documented for other vector‐borne plant pathogens (Belliure *et al*., 2008). Although not significant, it is notable that larvae grew the largest in the presence of both BPMV infection and *B. japonicum* colonization (Fig. 4). This suggests that BPMV and *B. japonicum* effects on host traits underlying suitability for beetle vector growth are at least not antagonistic. Larval performance was also increased on *B. japonicum*‐inoculated plants in the absence of BPMV infection, likely reflecting enhanced nutritional quality and reduced defensive compounds (see the ‘*Bradyrhizobium japonicum* inoculation reallocates resources from defense toward symbiosis’ section), in line with other examples of rhizobacteria‐mediated increases in herbivore performance (Pineda *et al*., 2012; Dean *et al*., 2014).

We did not examine virus transmission in this study, but our results indicate these processes should be the target of future work. BPMV effects on vectors may be a product of viral adaptations for manipulating behavior in relation to infected hosts, as documented in other studies on plant viruses transmitted by diverse vector species (Ray & Casteel, 2022). Our study provides evidence that these putative adaptations induce the most transmission‐conducive phenotypes when plants associate with rhizobacteria, especially the co‐evolved *B. japonicum* symbiont. A similar outcome was also documented for an aphid‐transmitted virus infecting *Medicago sativa*, with the most transmission‐conducive phenotype only being observed when plants co‐associated with root‐nodulating *Sinorhizobium meliloti* (Nenadić *et al*., 2022). The modifications of virus‐induced phenotypes by root microbes observed in this study, and prior work may reflect the selective environment in which virus adaptations arise, in which the host physiology to be manipulated during infection is already shaped by co‐evolved bacterial symbiont and their effects on metabolites and defenses.

Rhizobacterial treatments containing *B. japonicum* and *D. acidovorans* tended to enhance BPMV effects on beetle behavior underlying virus acquisition, but BPMV infection negatively affected the *B. japonicum* symbiont. BPMV‐infected plants inoculated with *B. japonicum* had reduced nodule biomass. Nodule weight is correlated with the number of bacterial cells within nodules (Ratcliff *et al*., 2011), suggesting fitness costs for *B. japonicum* associating with BPMV‐infected plants. BPMV effects on nodule weight, and relationships between nodule weight and shoot biomass in infected plants were similar regardless of whether *D. acidovorans* was present with *B. japonicum*. However, in uninfected plants, *D. acidovorans* did alter the relationship between shoot biomass and nodule weight from a negative correlation (*B. japonicum* alone) to a positive correlation (Fig. 5). This indicates that in the absence of virus infection, co‐inoculation alters how nodulation scales with plant growth, possibly resulting in increased *B. japonicum* fitness when *D. acidovorans* is present. Testing this will require more precise quantification of bacterial cells in nodules under single and dual rhizobacteria treatments, as well as efforts to quantify *D. acidovorans* fitness.

### BPMV infection induces defense and flavonoid biosynthesis genes yet lowers flavonoid levels

Our metabolic profiles indicate that BPMV is the primary driver of beetle feeding preferences and larval performance. Infection significantly depleted key flavonoids, including eriocitrin, luteolin, and kaempferol (Fig. 7; Table S9), despite the upregulation of flavonoid biosynthesis and other defense‐related genes. These changes in flavonoid biosynthesis have not been previously reported in soybeans following BPMV infection, although the induction of defense pathways is well‐documented in response to pests and necrotrophic fungi (Dastmalchi *et al*., 2017). Because flavonoids and isoflavonoid phytoalexins can deter herbivores such as *E. varivestis* (Hart *et al*., 1983; Treutter, 2006), their depletion likely contributes to the increased beetle feeding observed on infected plants, aiding virus acquisition.

Despite the reduction in flavonoid levels, BPMV infection triggered the upregulation of pathways associated with phenylpropanoid, isoflavonoid, and flavonoid biosynthesis, along with other defense‐related secondary metabolites. Similar upregulation of these pathways in response to viral infection‐induced stress has been documented in previous studies (Jiang *et al*., 2021). The apparent contradiction – lower flavonoid levels despite increased expression of biosynthesis genes – could be due to the plant's defense response being counteracted by viral effectors through interference with post‐transcriptional regulation mechanisms (Heinlein, 2025).

The upregulation of phenylpropanoid biosynthesis and pentose and glucuronate interconversion pathways in BPMV‐infected plants suggests that the phenylpropanoid pathway may compensate for flavonoid depletion. This pathway is involved in synthesis of lignin, which provides structural support and defense against pathogens and herbivores. Elevated levels of L‐beta‐aspartyl‐L‐phenylalanine and phenylalanine ammonia‐lyase (PAL) in BPMV‐infected plants (Files S5.1 and S5.2) further support this hypothesis, suggesting that phenylalanine is being diverted into the phenylpropanoid pathway rather than the flavonoid pathway. The downregulation of purine metabolism in BPMV‐infected plants aligns with studies showing that viral infections can disrupt nucleotide metabolism in plants (Yue *et al*., 2018).

BPMV infection also altered primary metabolism. Infected plants accumulated sugars and organic acids, including D‐glucuronic acid, D‐fructose, and pyruvic acid, as well as amino acids such as glycine, aspartic acid, and 4‐aminobutanoic acid. These results agree with earlier observations of increased amino acid and carbohydrate concentrations in BPMV‐exposed soybean (Peñaflor *et al*., 2016), although they differ from findings of reduced amino acids in other studies (Smith *et al*., 2017). Many of these primary metabolites have roles in stress regulation (Rojas *et al*., 2014; Mur *et al*., 2017), suggesting that BPMV infection induces a broad reconfiguration of metabolic pathways that may enhance host quality for herbivores.

### *Bradyrhizobium japonicum* inoculation reallocates resources from defense toward symbiosis

In rhizobacteria‐inoculated plants, flavonoid depletion and downregulation of carotenoid biosynthesis, MAPK signaling, and PAL transcripts indicate reduced investment in chemical defenses. Suppression of carotenoid and phenylpropanoid pathways, which contribute to herbivore resistance (Jones *et al*., 2019; Chen *et al*., 2021), likely increases susceptibility to *E. varivestis*. These chemical changes are consistent with increased feeding on *B. japonicum*‐colonized plants (Fig. 3a).

Co‐inoculation with *B. japonicum* and *D. acidovorans* intensified shifts in primary metabolism, including increases in amino acids, carboxylic acids, and fatty acids, as well as upregulation of amino sugar and nucleotide sugar metabolism and pentose and glucuronate interconversions. These changes, associated with nodule development and nitrogen fixation (Brechenmacher *et al*., 2010), align with previous studies linking rhizobacterial colonization with activation of growth‐related pathways and reduced defense (Brechenmacher *et al*., 2008). Although recent work suggests that growth and defense are not always antagonistic (Afzal *et al*., 2019), our results indicate substantial resource reallocation during nodulation, reducing defense investment and increasing herbivory.

As noted previously, uninoculated plants received nitrate supplementation while rhizobia‐inoculated plants did not, which introduces some confounding effects and complicates interpretation of rhizobacterial effects. However, nitrogen supplementation was necessary to avoid severe nutrient stress in non‐nodulating controls, and providing nitrogen to rhizobacterial treatments would suppress nodulation, defeating the purpose of the study. Similar approaches have been employed in other studies (Kontopoulou *et al*., 2015; Allito *et al*., 2021; Ramula *et al*., 2023; Win *et al*., 2023). Moreover, although some of the responses we observed may reflect differences in nitrogen availability, several patterns point to effects of rhizobial association that go beyond nitrogen supply. Rhizobia‐inoculated plants and fertilized controls differed in both metabolic composition and beetle feeding responses, and these differences are not easily explained by nitrate supplementation alone. This suggests that physiological changes linked to rhizobial colonization, including nodule development and associated shifts in resource allocation, contributed to the plant and herbivore outcomes in our experiment. While nitrogen remains a factor to consider, the broader ecological picture remains robust: Rhizobacterial symbiosis reshapes soybean metabolism and modifies vector herbivore behavior.

### Conclusion

Our study demonstrates that soybean leaf metabolite production is regulated by the combined effects of beneficial rhizobacteria and BPMV infection. Both rhizobacteria inoculation and BPMV infection significantly altered beetle feeding preferences and performance. Beetles preferred feeding on BPMV‐infected plants over uninfected ones, and gained more weight on these plants, but beetle foraging preferences only shifted to favor BPMV‐infected plants when those plants were also inoculated with rhizobacteria. Feeding and larval performance outcomes correlated with higher levels of primary metabolites and lower levels of secondary metabolites in infected and rhizobacteria‐inoculated plants. These findings suggest that the combined effects of beneficial rhizobacteria and BPMV infection may inadvertently enhance virus acquisition and transmission by creating more nutritious and attractive host plants for the beetle vector. At the same time, at least one of the rhizobacterial symbionts (*B. japonicum*) may have reduced fitness due to its host being infected with BPMV.

The results highlight complex interactions among symbionts that affect plant metabolites and defense mechanisms. While BPMV infection exerts strong effects on feeding behavior of its vector, co‐occurring rhizobacteria modulate BPMV‐induced changes in plant traits mediating between‐plant movement of vectors, such as volatile cues (Pulido *et al*., 2019). Our study emphasizes the importance of considering co‐occurring microbes when evaluating virus‐induced host phenotypes in natural environments. The clear effects and mechanisms documented here are likely driving virus epidemiological processes in these environments and testing this will require future work performed at field scales over longer time frames. Our results are also useful for understanding how we might manipulate plant–microbe interactions in less ecologically complex agricultural settings. While beneficial microorganisms are increasingly being used in agriculture to enhance plant growth and defense against pests and diseases (Elnahal *et al*., 2022), our research suggests that the effects of microbial inoculants on plant metabolism can lead to unintended consequences, including changes in herbivore behavior and performance that lead to higher levels of damage and potentially increased virus transmission. As researchers and industry pursue microbe‐based product development, understanding these interactions will be critical for developing materials that effectively balance plant growth and defense.

## Competing interests

None declared.

## Author contributions

HP, KEM, MCM and CDM conceived and designed the study. HP conducted the experiments, curated data, performed statistical and bioinformatic analyses, and drafted the initial manuscript. All authors contributed to revisions and gave final approval for publication.

## Disclaimer

The New Phytologist Foundation remains neutral with regard to jurisdictional claims in maps and in any institutional affiliations.

## Supporting information


**Dataset S1** Limma results for metabolite differential abundance; ranked metabolites and statistics for all pairwise contrasts (Figs 7, S2–S4).

**Dataset S2** DESeq2 differential expression results for pairwise contrasts (Figs 8, S6–S8); includes gene identifiers, log_2_FC, *P*‐values, and adjusted *P*‐values.

**Dataset S3** WGCNA module membership table with gene IDs, symbols, Entrez IDs, module assignments, and hub gene positions.

**Dataset S4** Differential expression within WGCNA modules across three contrasts; includes logFC, t‐statistics, *P*‐values, and adjusted *P*‐values.

**Dataset S5** Pathway enrichment results from GAGE analyses; includes KEGG IDs, pathway names, regulation status, enrichment statistics, *P*‐values, q‐values, and set sizes.

**Dataset S6** KEGG pathway maps for all pairwise comparisons; folder names correspond to specific treatment contrasts listed in heading of Dataset S6 in the main .docx document.

**Dataset S7** Summary KEGG pathway diagrams for three major contrasts corresponding to Fig. 9.
**Fig. S1** Experimental setup illustrating adult beetle dual‐choice feeding and foraging assays.
**Fig. S2** Volcano plots showing rhizobacteria‐induced metabolite changes in uninfected soybean plants.
**Fig. S3** Volcano plots showing rhizobacteria‐induced metabolite changes in BPMV‐infected soybean plants.
**Fig. S4** Volcano plots showing BPMV‐induced metabolite changes under rhizobacteria and mixed treatment conditions.
**Fig. S5** Number of signature genes identified per pairwise treatment comparison.
**Fig. S6** Volcano plots of rhizobacteria‐induced signature gene profiles in uninfected soybean plants.
**Fig. S7** Volcano plots of rhizobacteria‐induced signature gene profiles in BPMV‐infected soybean plants.
**Fig. S8** Volcano plots summarizing soybean gene expression changes across rhizobacteria inoculation and BPMV infection treatments.

**Methods S1** Detailed protocols for rhizobacteria culture, BPMV inoculation.
**Methods S2** Description of plant growth conditions, and experimental design used in soybean assays.
**Methods S3** Description of metabolite extraction and analysis.
**Methods S4** Integrative transcriptome–metabolome analyses, feature selection procedures, enrichment analyses, and pathway mapping workflows.
**Table S1** Full factorial design combining rhizobacteria inoculation treatments and BPMV infection status.
**Table S2** Exact binomial test results for all pairwise dual‐choice beetle foraging contrasts.
**Table S3** ANOVA results for adult beetle feeding damage across rhizobacteria and virus treatments.
**Table S4** ANOVA and Tukey HSD *post‐hoc* comparisons for larval weight across treatments.
**Table S5** ANOVA summary for leaf toughness across rhizobacteria and virus treatments.
**Table S6** ANOVA summary for leaf biomass across rhizobacteria and virus treatments.
**Table S7** ANOVA summary for nodule biomass across virus treatments.
**Table S8** Regression analyses of nodule weight vs shoot biomass across virus treatments.
**Table S9** Identified metabolites with compound IDs and corresponding KEGG and HMDB annotations when available.
**Table S10** Behavioral contrasts and statistical approaches used for signature gene and metabolite selection.
**Table S11** Top six hub genes for each co‐expression module highlighted in the network analysis.

**Video S1** Representative dual‐choice assay demonstrating adult beetle foraging behavior under contrasting treatment conditions.
Please note: Wiley is not responsible for the content or functionality of any Supporting Information supplied by the authors. Any queries (other than missing material) should be directed to the *New Phytologist* Central Office.

## Data Availability

The complete results of the gene enrichment analysis, linear models (limma), differential gene expression analysis, and WGCNA are provided as Excel files in the supporting information. Additional supplementary methods, tables, and figures are available in the ETHZ repository https://doi.org/10.3929/ethz‐b‐000692860. The source code and data used for the analyses in this paper can be accessed in the following repository: https://gitlab.ethz.ch/hannierp/beetles_metabolomics.git. Raw sequence reads are available from the NCBI read archive under the accession no. GSE244001.
