# Supplementary figures and images for: Beneficial rhizobacteria and virus infection modulate the soybean metabolome and influence the feeding preferences of the virus vector Epilachna varivestis (part 1 of 3)
Source: New Phytol. 2026 Mar 24;250(4):2599–618. doi: 10.1111/nph.71104 (PMC13103440; doi:10.1111/nph.71104)

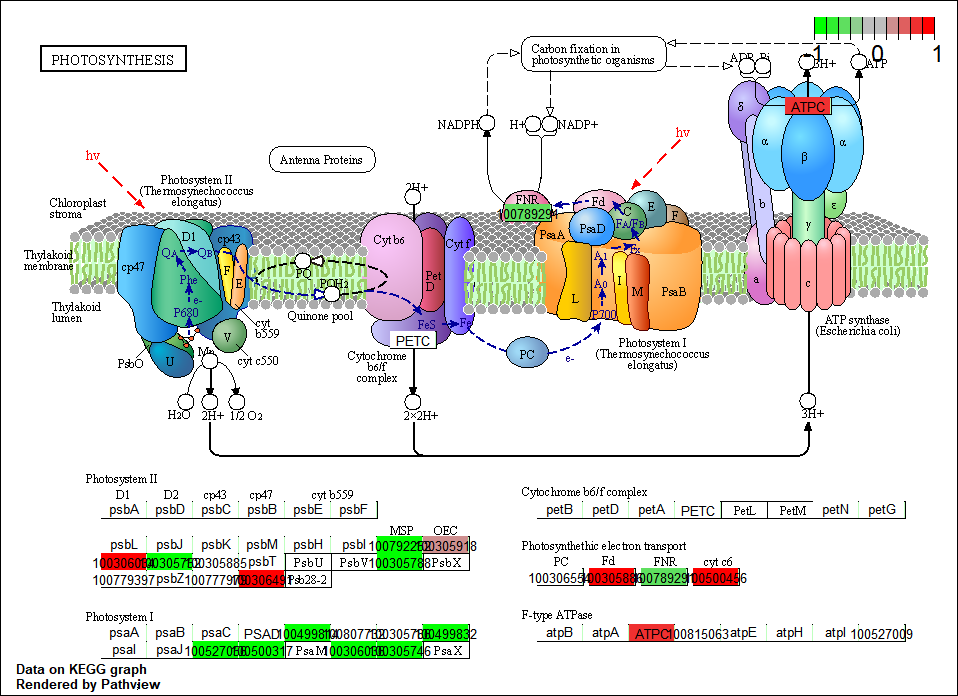

Supplement: Supplementary file 6 — Dataset S6 KEGG pathway maps for all pairwise comparisons; folder names correspond to specific treatment contrasts listed in heading of Dataset S6 in the main .docx document. [file NPH-250-2599-s003.zip › a.BPMV - Bj vs control, BPMV/down/gmx00195.pathview.png]

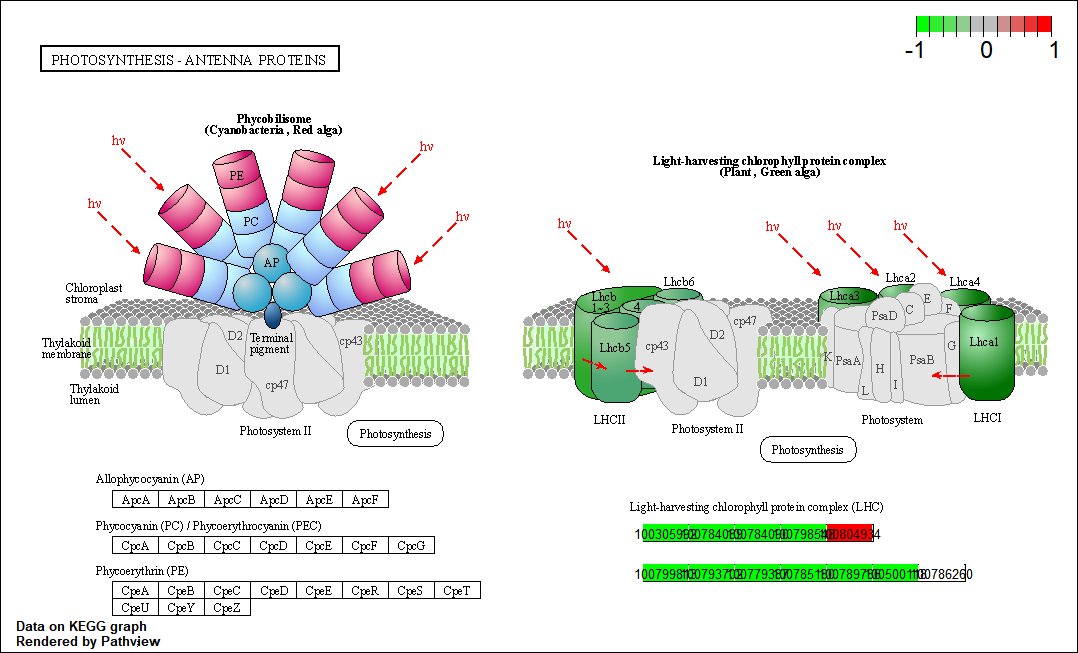

Supplement: Supplementary file 6 — Dataset S6 KEGG pathway maps for all pairwise comparisons; folder names correspond to specific treatment contrasts listed in heading of Dataset S6 in the main .docx document. [file NPH-250-2599-s003.zip › a.BPMV - Bj vs control, BPMV/down/gmx00196.pathview.png]

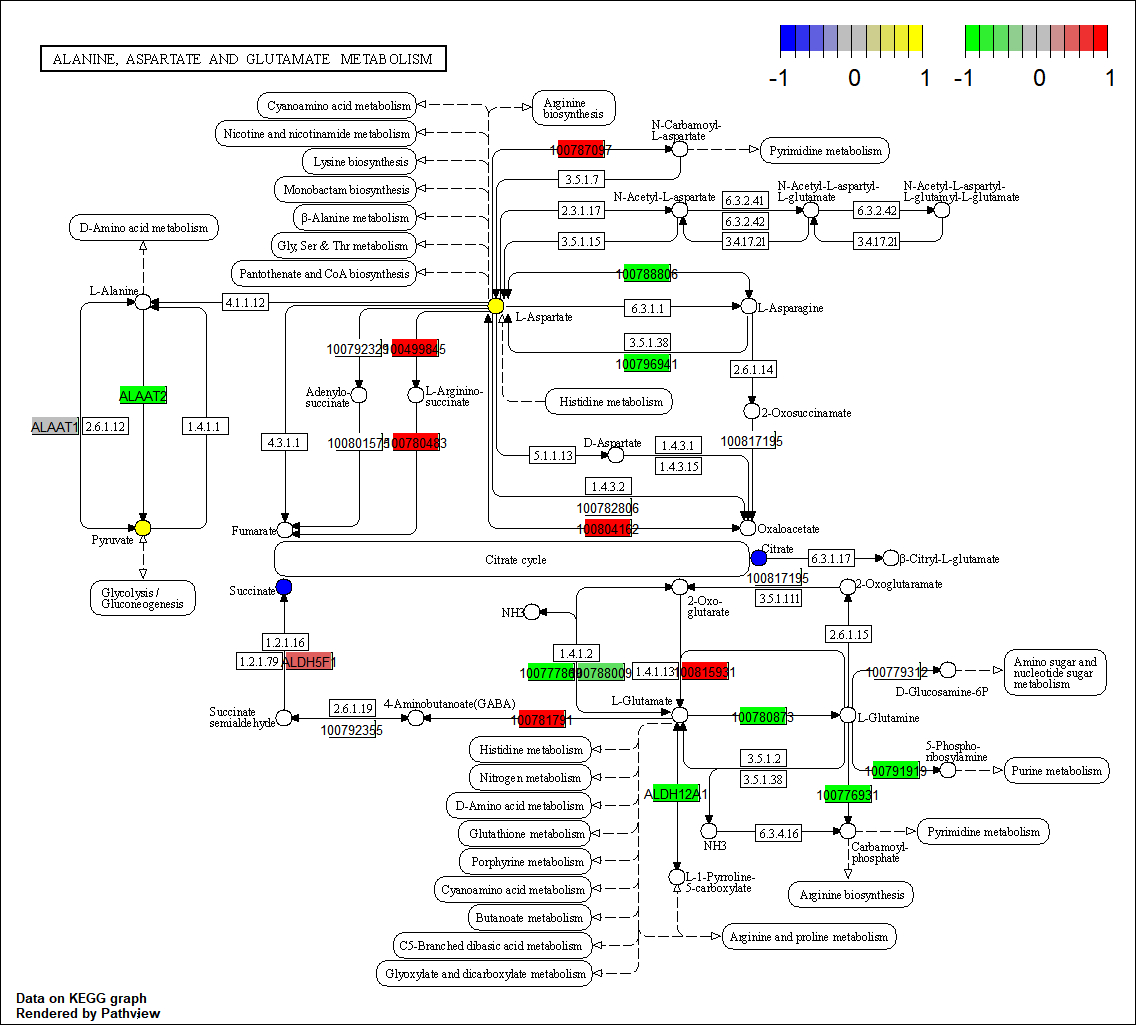

Supplement: Supplementary file 6 — Dataset S6 KEGG pathway maps for all pairwise comparisons; folder names correspond to specific treatment contrasts listed in heading of Dataset S6 in the main .docx document. [file NPH-250-2599-s003.zip › a.BPMV - Bj vs control, BPMV/down/gmx00250.pathview.png]

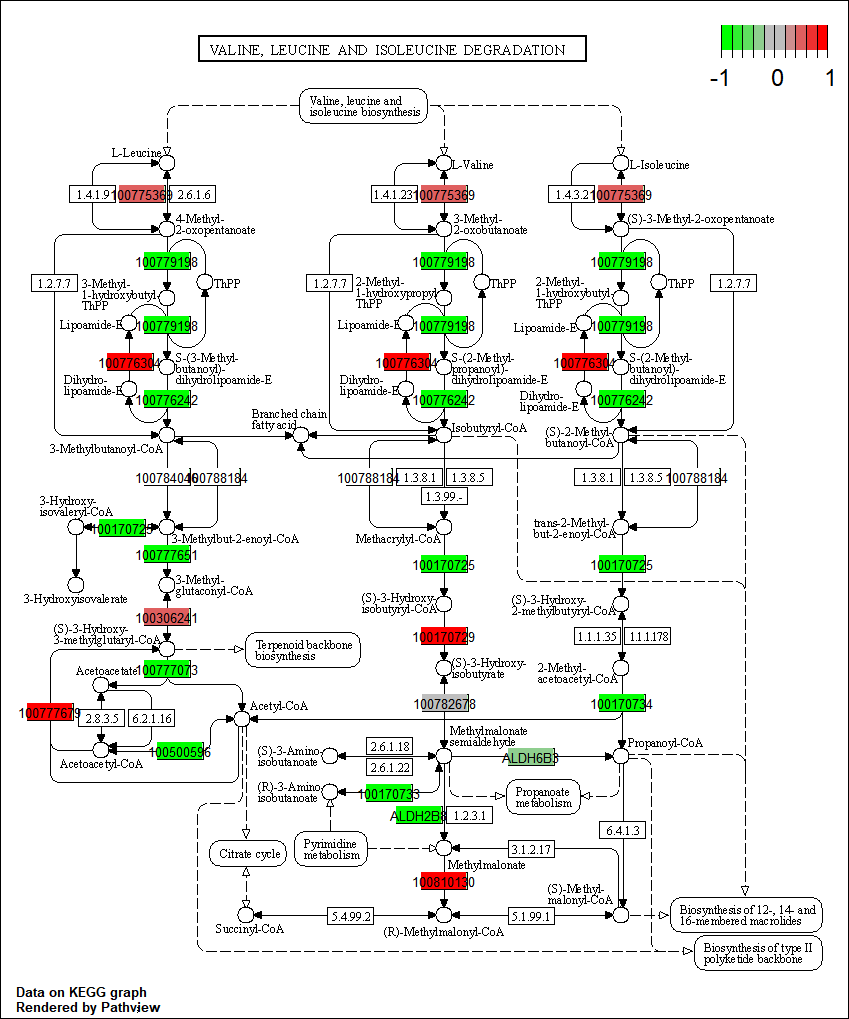

Supplement: Supplementary file 6 — Dataset S6 KEGG pathway maps for all pairwise comparisons; folder names correspond to specific treatment contrasts listed in heading of Dataset S6 in the main .docx document. [file NPH-250-2599-s003.zip › a.BPMV - Bj vs control, BPMV/down/gmx00280.pathview.png]

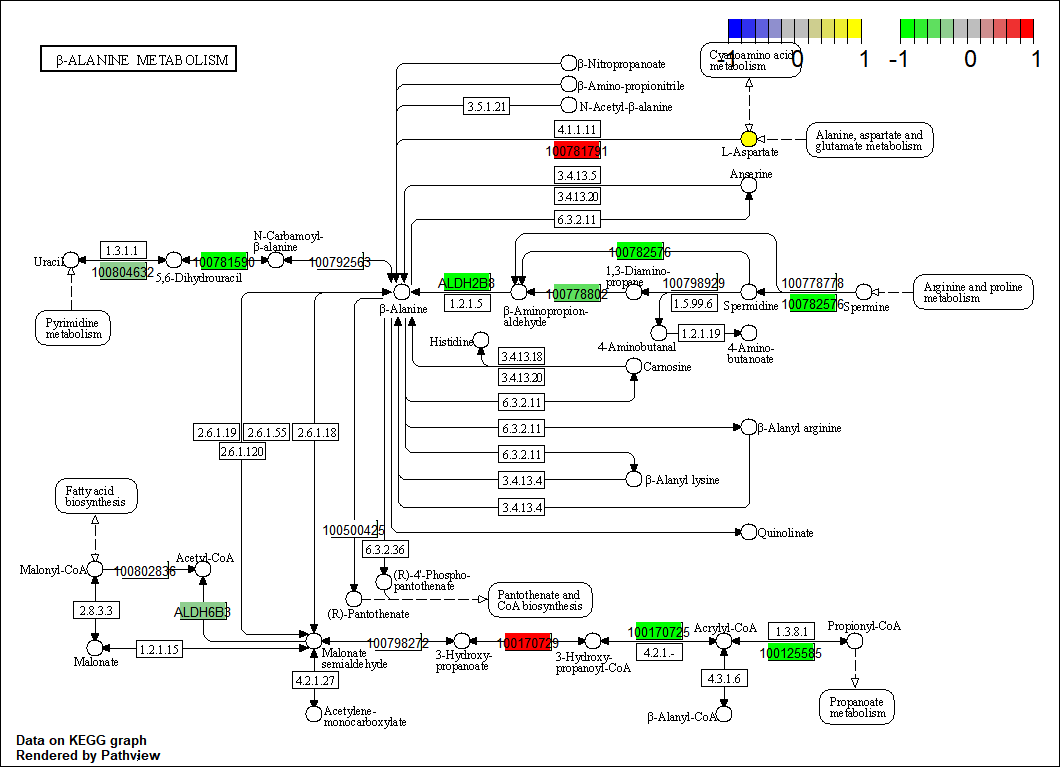

Supplement: Supplementary file 6 — Dataset S6 KEGG pathway maps for all pairwise comparisons; folder names correspond to specific treatment contrasts listed in heading of Dataset S6 in the main .docx document. [file NPH-250-2599-s003.zip › a.BPMV - Bj vs control, BPMV/down/gmx00410.pathview.png]

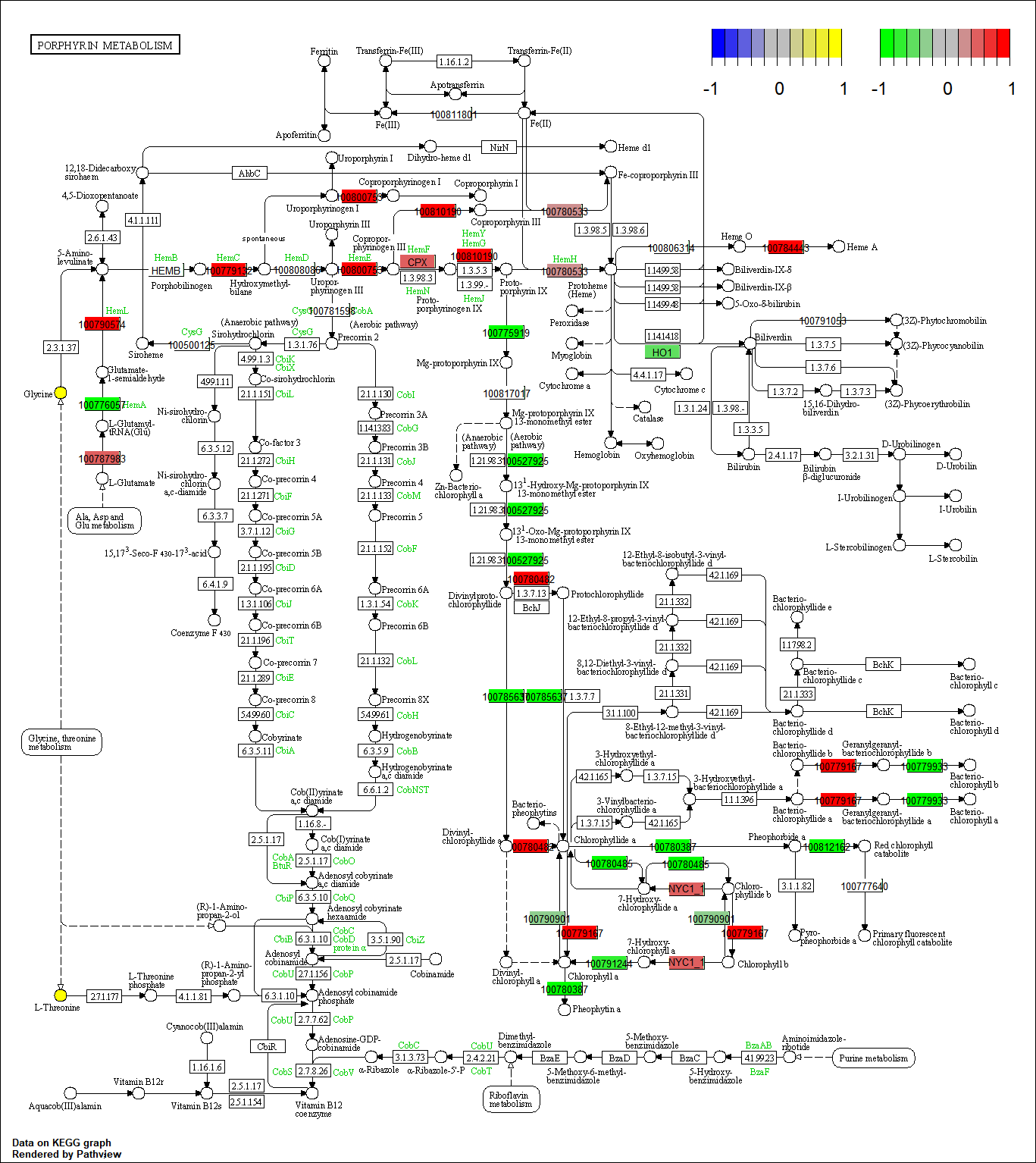

Supplement: Supplementary file 6 — Dataset S6 KEGG pathway maps for all pairwise comparisons; folder names correspond to specific treatment contrasts listed in heading of Dataset S6 in the main .docx document. [file NPH-250-2599-s003.zip › a.BPMV - Bj vs control, BPMV/down/gmx00860.pathview.png]

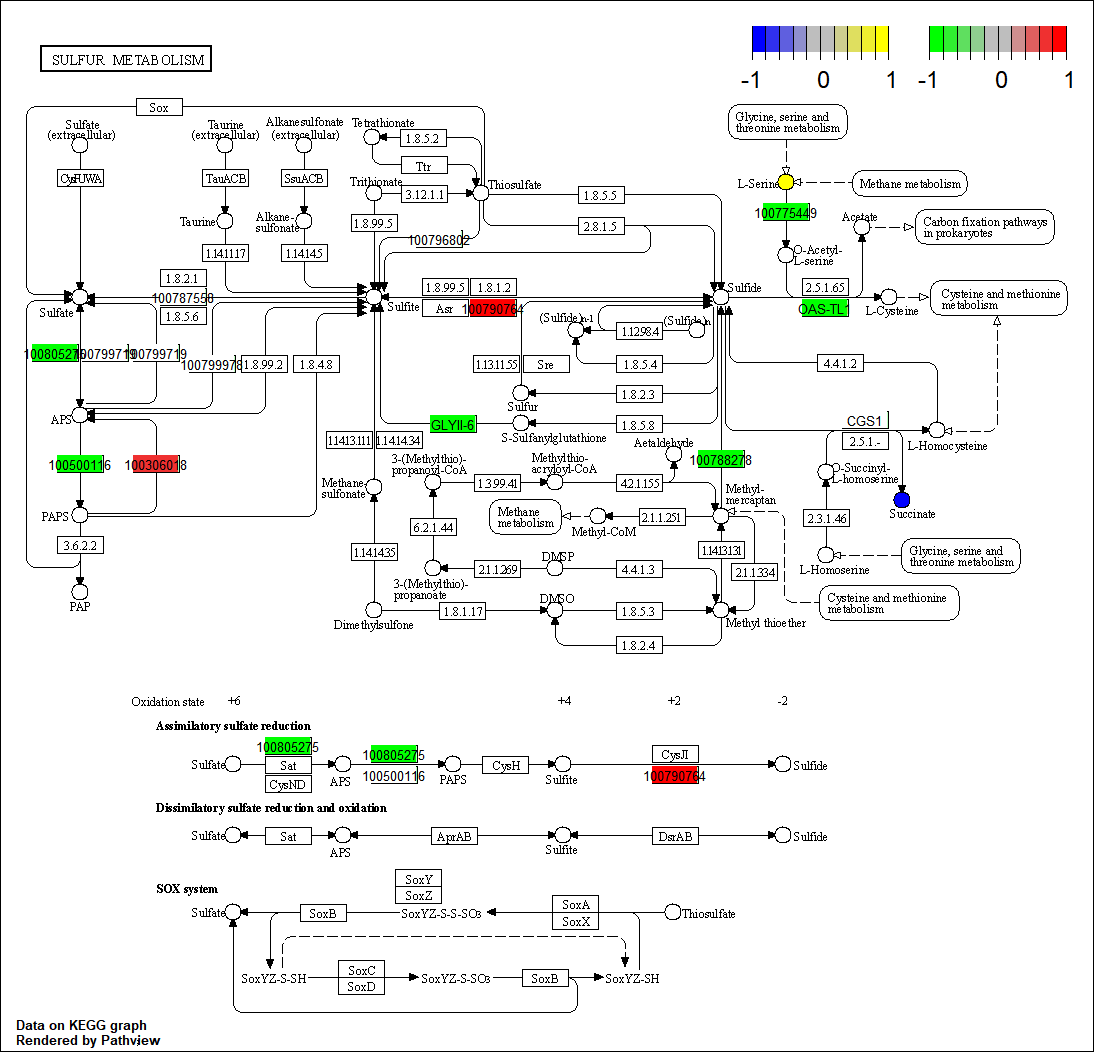

Supplement: Supplementary file 6 — Dataset S6 KEGG pathway maps for all pairwise comparisons; folder names correspond to specific treatment contrasts listed in heading of Dataset S6 in the main .docx document. [file NPH-250-2599-s003.zip › a.BPMV - Bj vs control, BPMV/down/gmx00920.pathview.png]

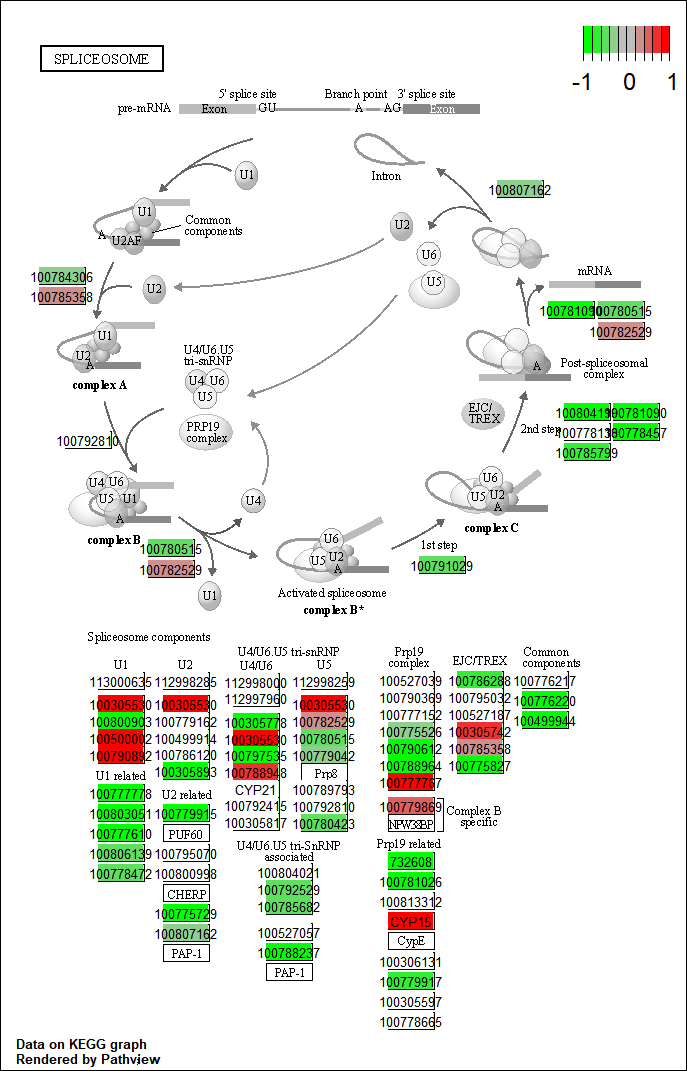

Supplement: Supplementary file 6 — Dataset S6 KEGG pathway maps for all pairwise comparisons; folder names correspond to specific treatment contrasts listed in heading of Dataset S6 in the main .docx document. [file NPH-250-2599-s003.zip › a.BPMV - Bj vs control, BPMV/down/gmx03040.pathview.png]

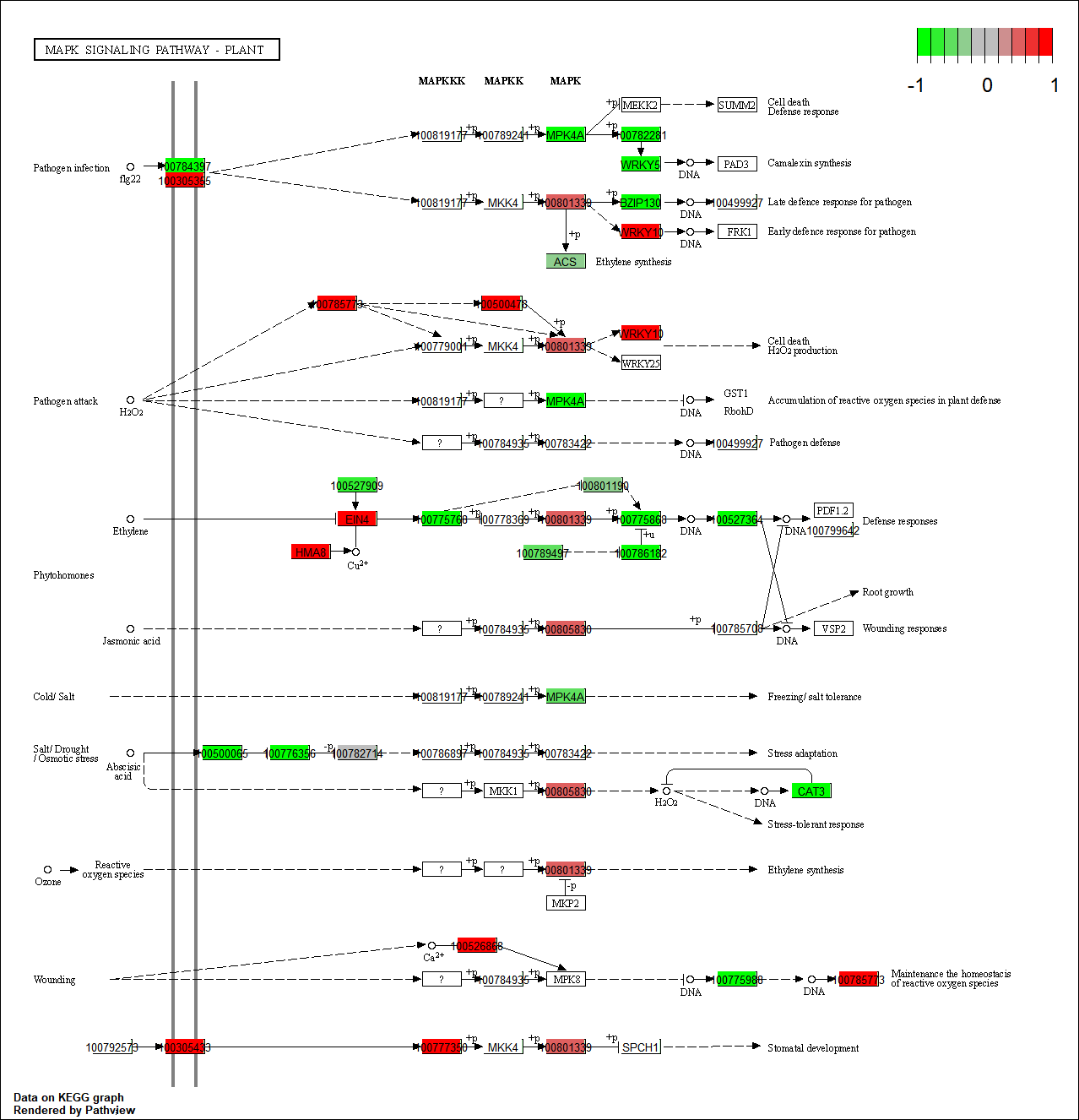

Supplement: Supplementary file 6 — Dataset S6 KEGG pathway maps for all pairwise comparisons; folder names correspond to specific treatment contrasts listed in heading of Dataset S6 in the main .docx document. [file NPH-250-2599-s003.zip › a.BPMV - Bj vs control, BPMV/down/gmx04016.pathview.png]

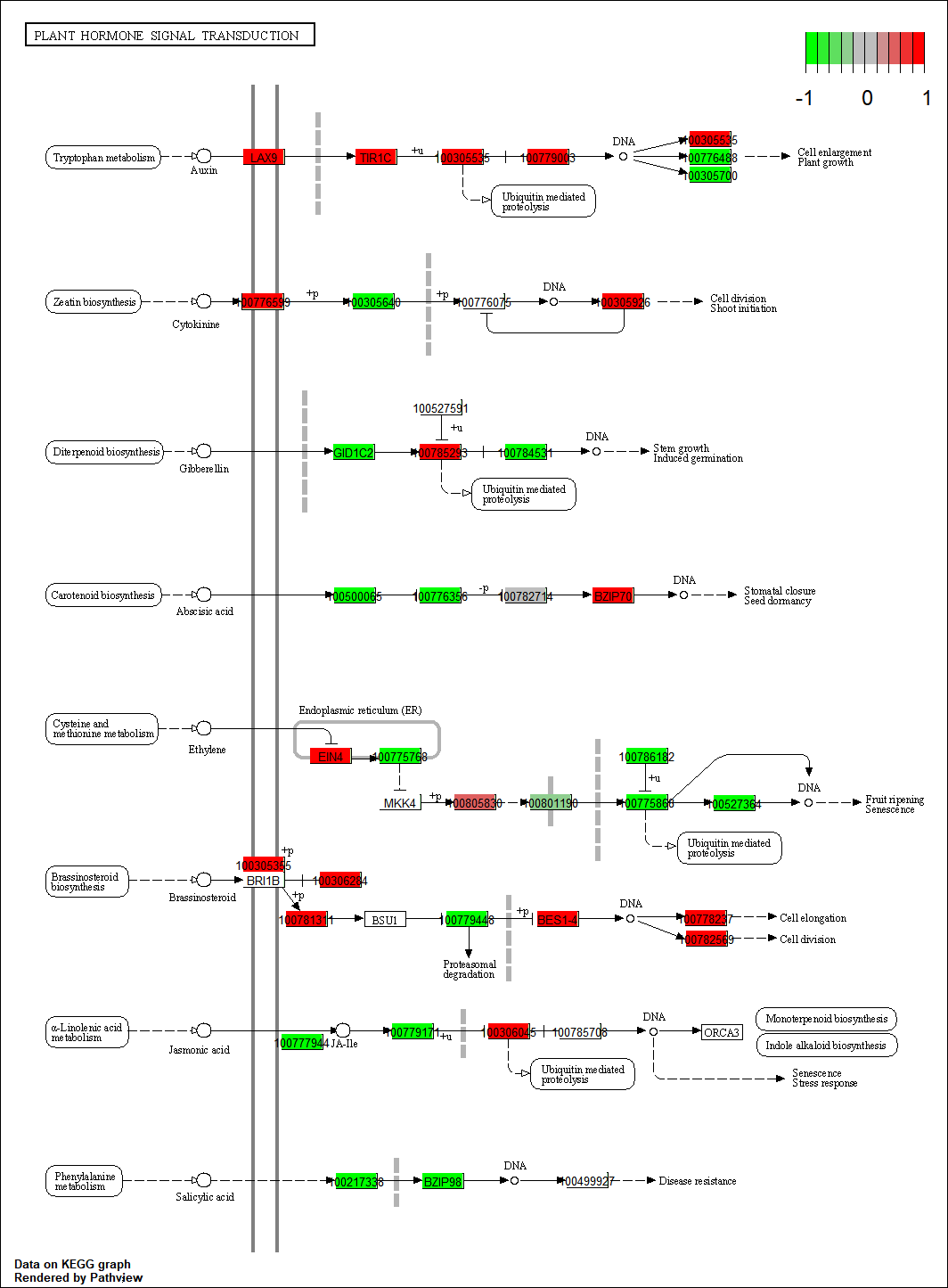

Supplement: Supplementary file 6 — Dataset S6 KEGG pathway maps for all pairwise comparisons; folder names correspond to specific treatment contrasts listed in heading of Dataset S6 in the main .docx document. [file NPH-250-2599-s003.zip › a.BPMV - Bj vs control, BPMV/down/gmx04075.pathview.png]

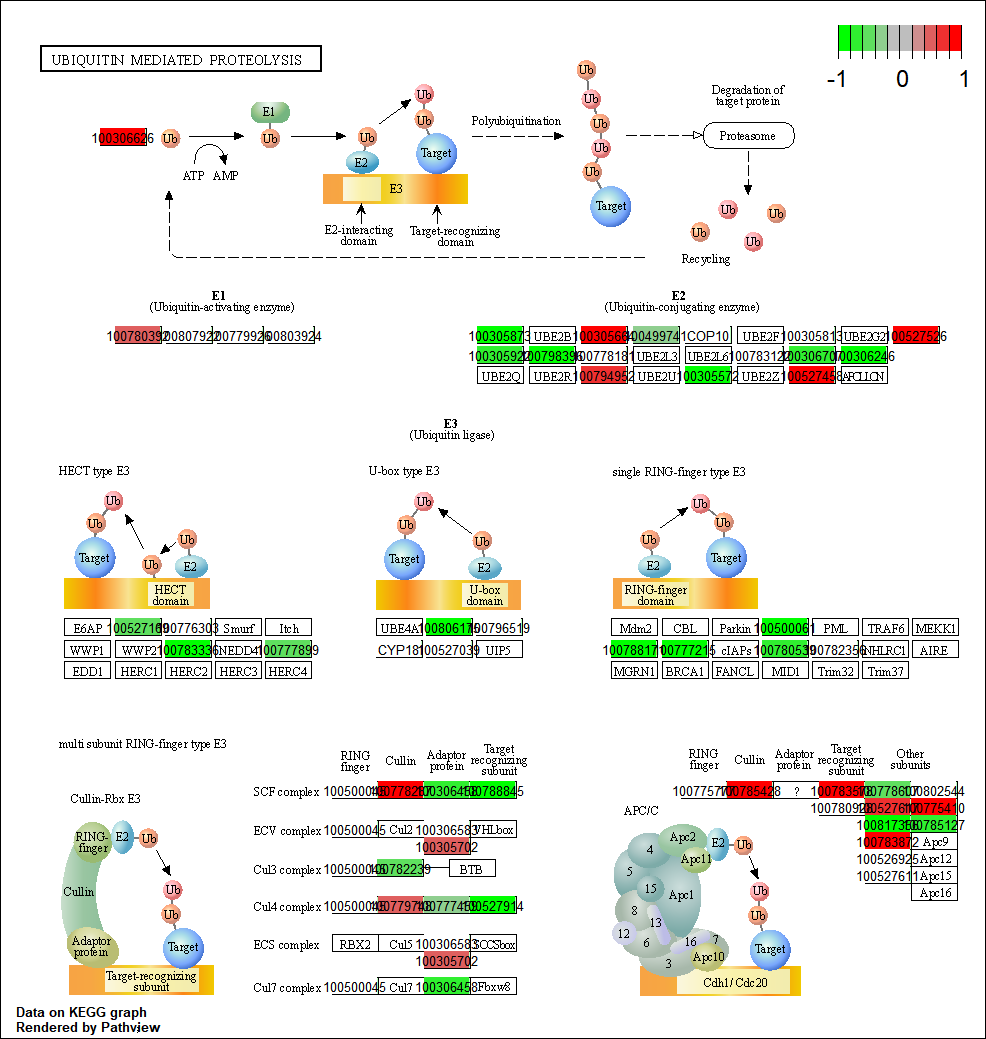

Supplement: Supplementary file 6 — Dataset S6 KEGG pathway maps for all pairwise comparisons; folder names correspond to specific treatment contrasts listed in heading of Dataset S6 in the main .docx document. [file NPH-250-2599-s003.zip › a.BPMV - Bj vs control, BPMV/down/gmx04120.pathview.png]

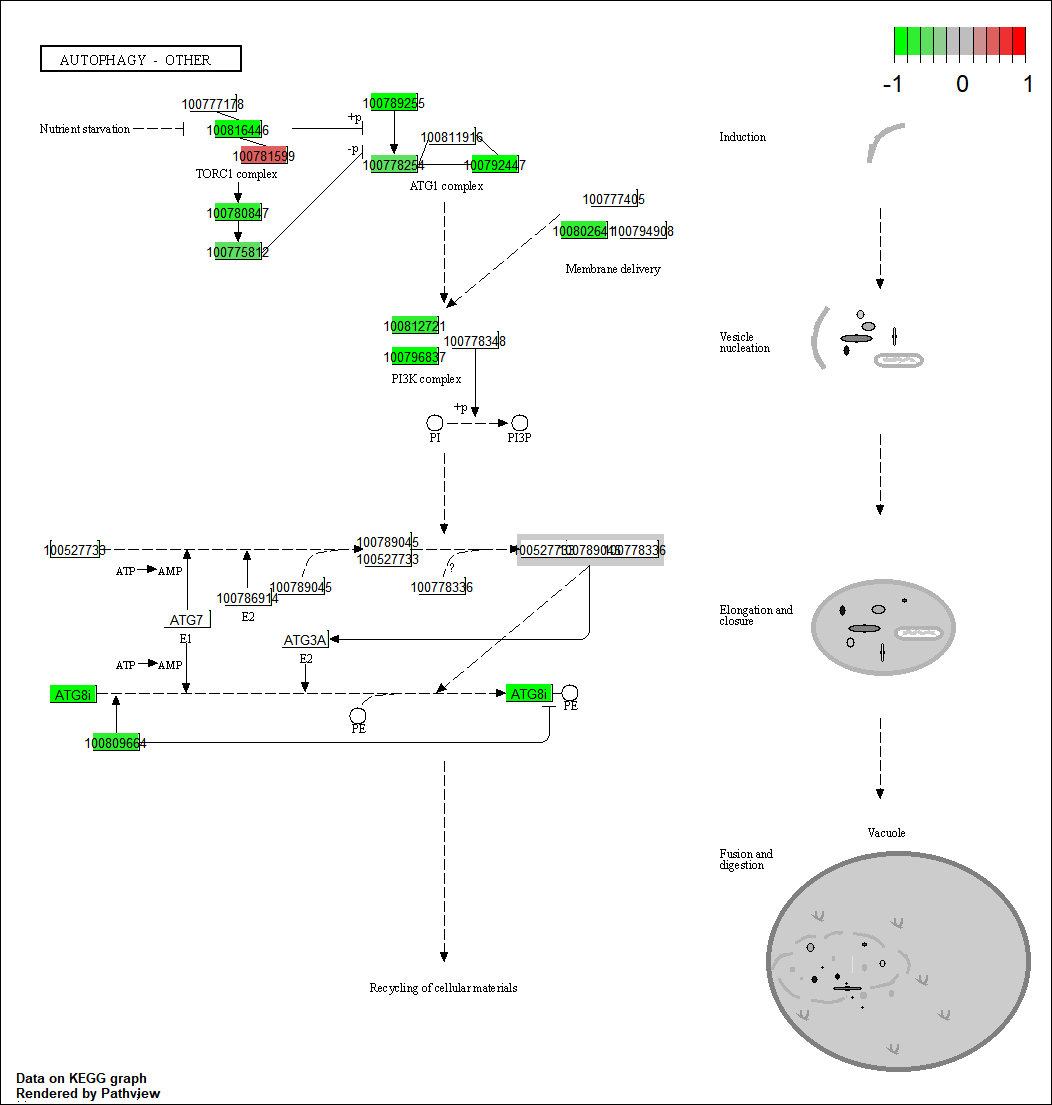

Supplement: Supplementary file 6 — Dataset S6 KEGG pathway maps for all pairwise comparisons; folder names correspond to specific treatment contrasts listed in heading of Dataset S6 in the main .docx document. [file NPH-250-2599-s003.zip › a.BPMV - Bj vs control, BPMV/down/gmx04136.pathview.png]

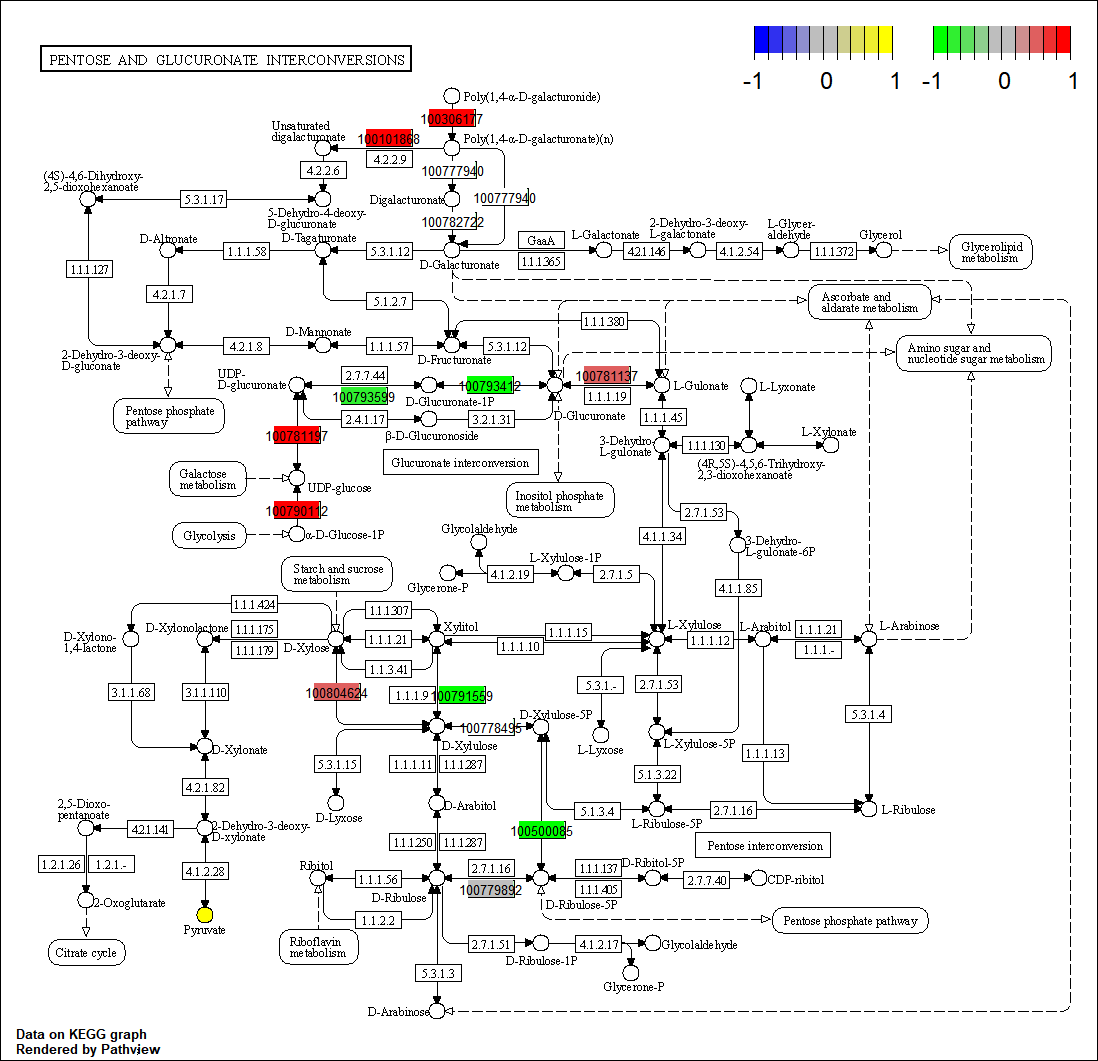

Supplement: Supplementary file 6 — Dataset S6 KEGG pathway maps for all pairwise comparisons; folder names correspond to specific treatment contrasts listed in heading of Dataset S6 in the main .docx document. [file NPH-250-2599-s003.zip › a.BPMV - Bj vs control, BPMV/up/gmx00040.pathview.png]

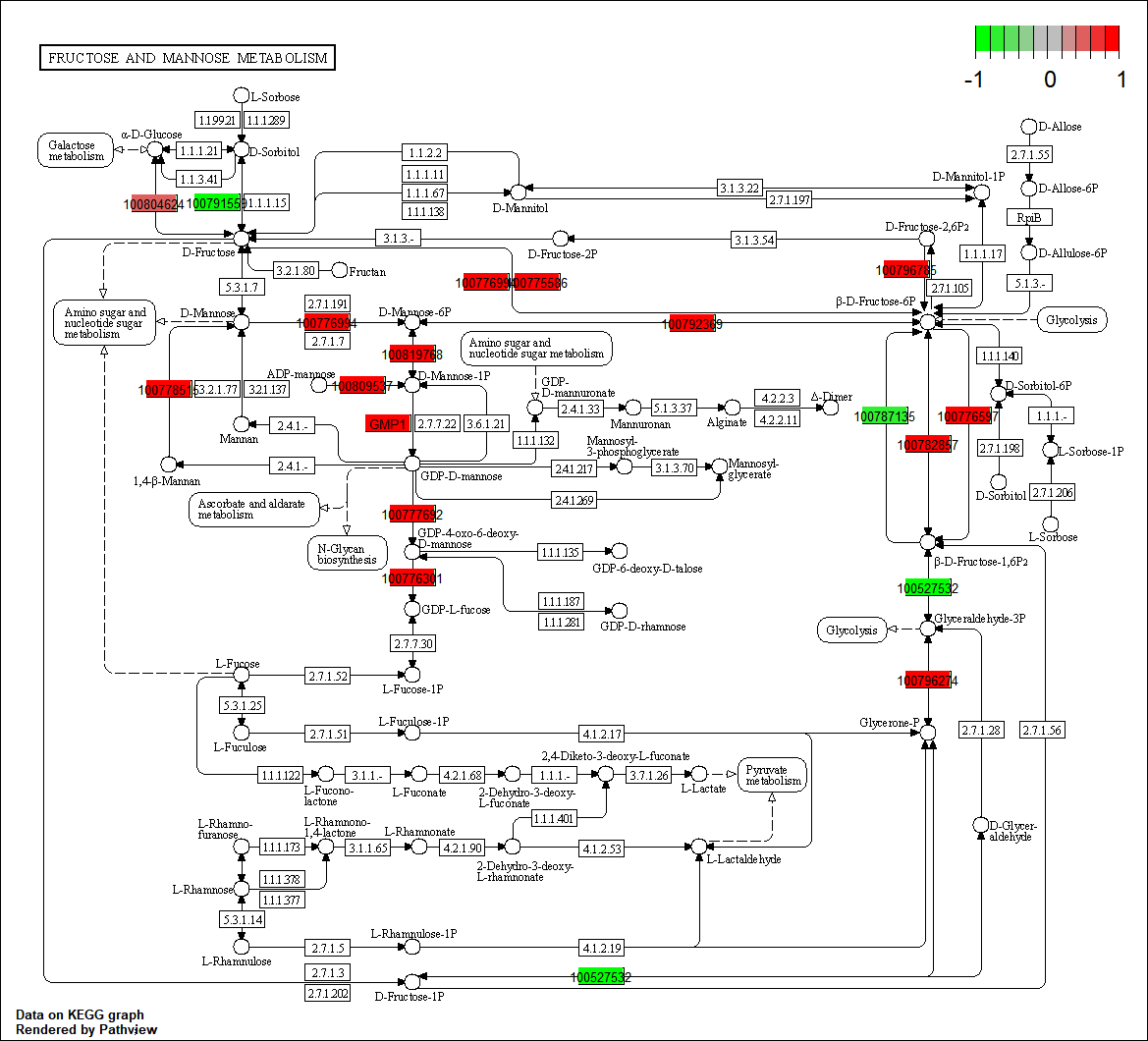

Supplement: Supplementary file 6 — Dataset S6 KEGG pathway maps for all pairwise comparisons; folder names correspond to specific treatment contrasts listed in heading of Dataset S6 in the main .docx document. [file NPH-250-2599-s003.zip › a.BPMV - Bj vs control, BPMV/up/gmx00051.pathview.png]

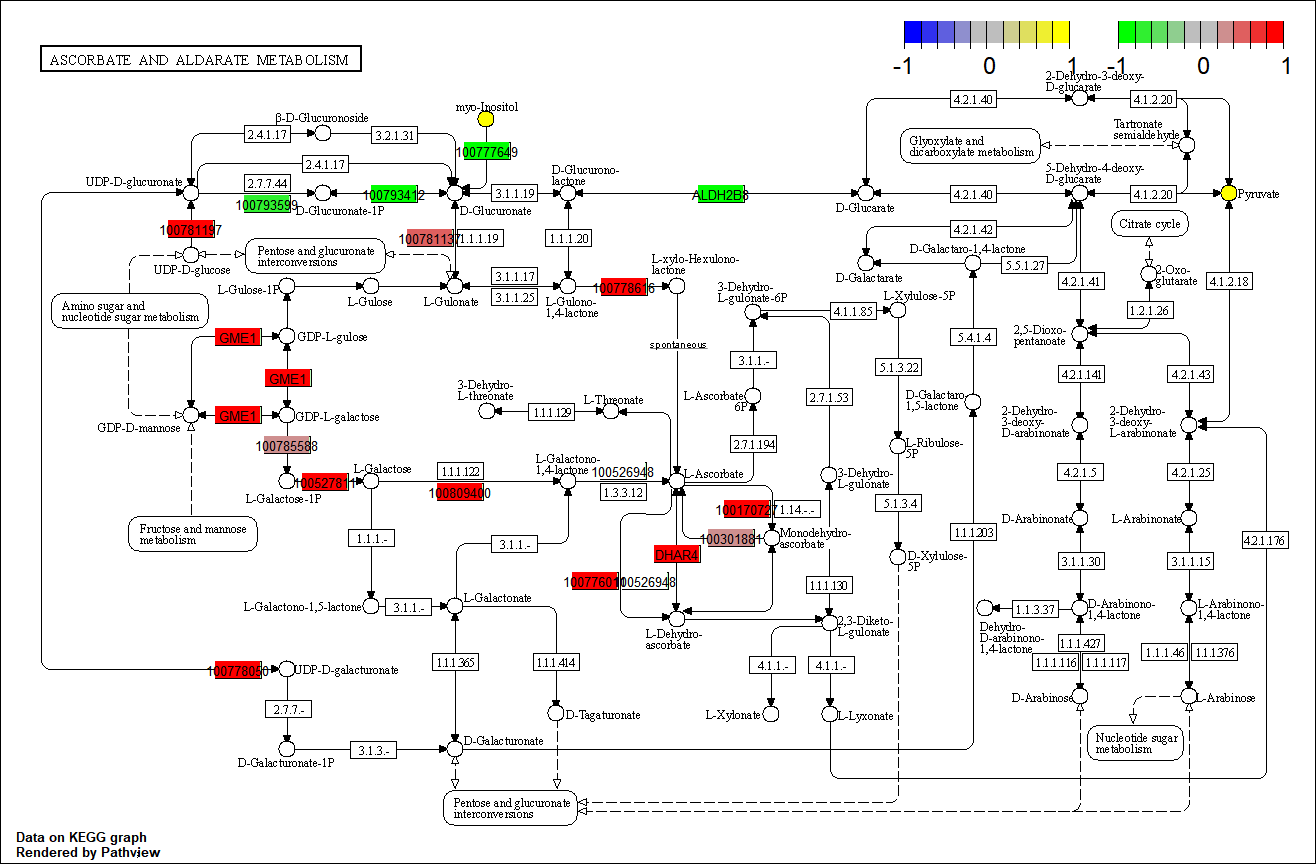

Supplement: Supplementary file 6 — Dataset S6 KEGG pathway maps for all pairwise comparisons; folder names correspond to specific treatment contrasts listed in heading of Dataset S6 in the main .docx document. [file NPH-250-2599-s003.zip › a.BPMV - Bj vs control, BPMV/up/gmx00053.pathview.png]

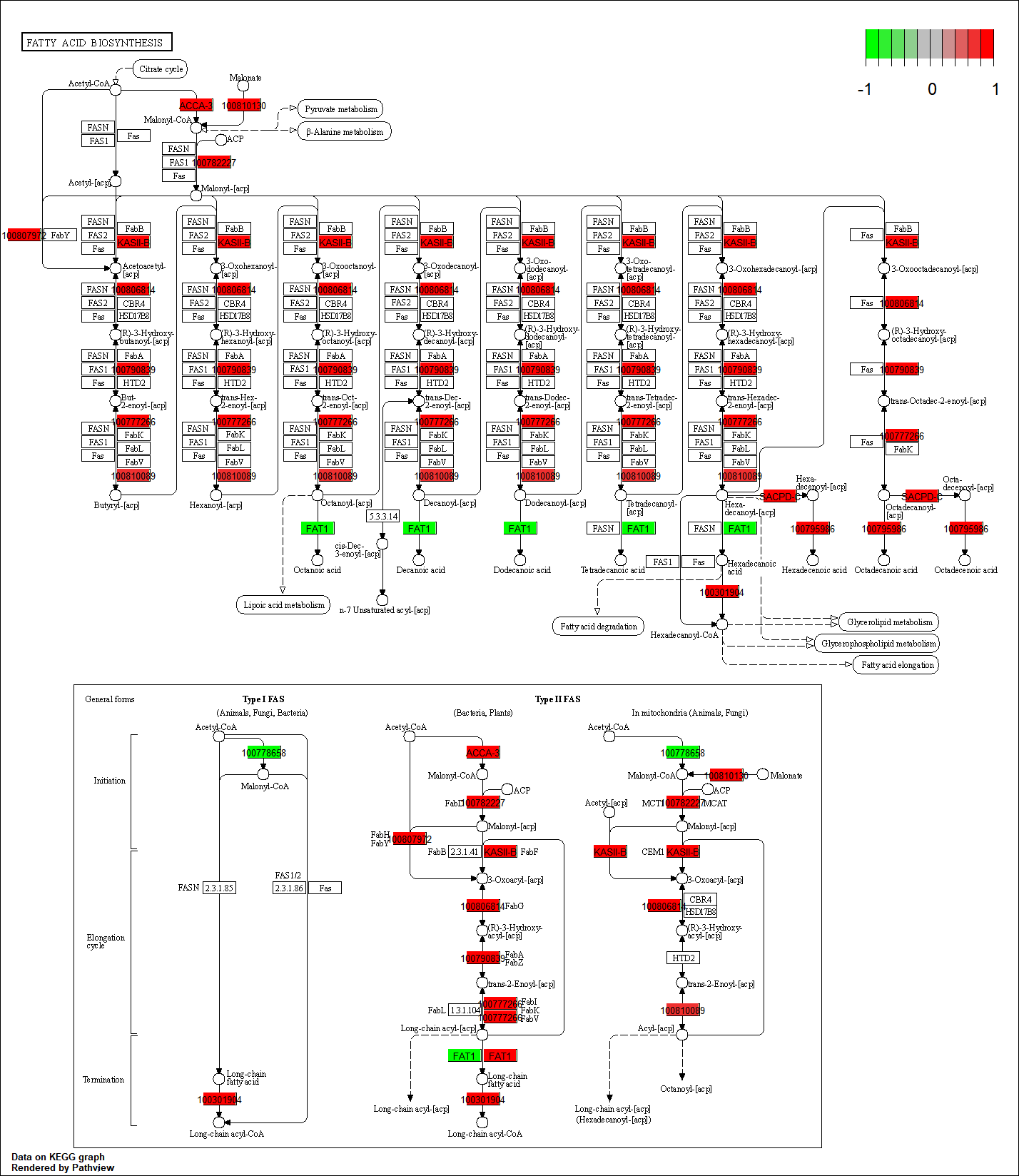

Supplement: Supplementary file 6 — Dataset S6 KEGG pathway maps for all pairwise comparisons; folder names correspond to specific treatment contrasts listed in heading of Dataset S6 in the main .docx document. [file NPH-250-2599-s003.zip › a.BPMV - Bj vs control, BPMV/up/gmx00061.pathview.png]

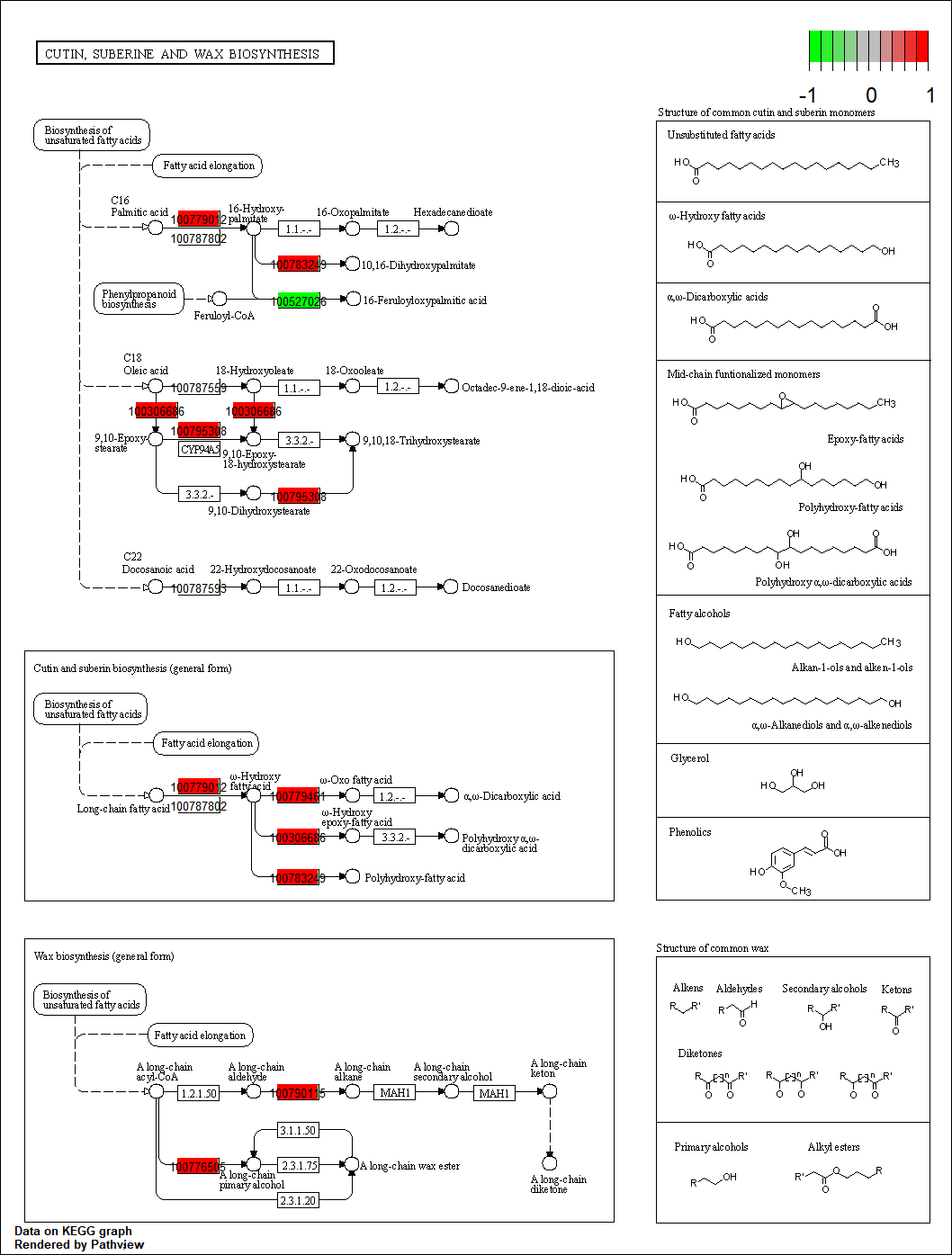

Supplement: Supplementary file 6 — Dataset S6 KEGG pathway maps for all pairwise comparisons; folder names correspond to specific treatment contrasts listed in heading of Dataset S6 in the main .docx document. [file NPH-250-2599-s003.zip › a.BPMV - Bj vs control, BPMV/up/gmx00073.pathview.png]

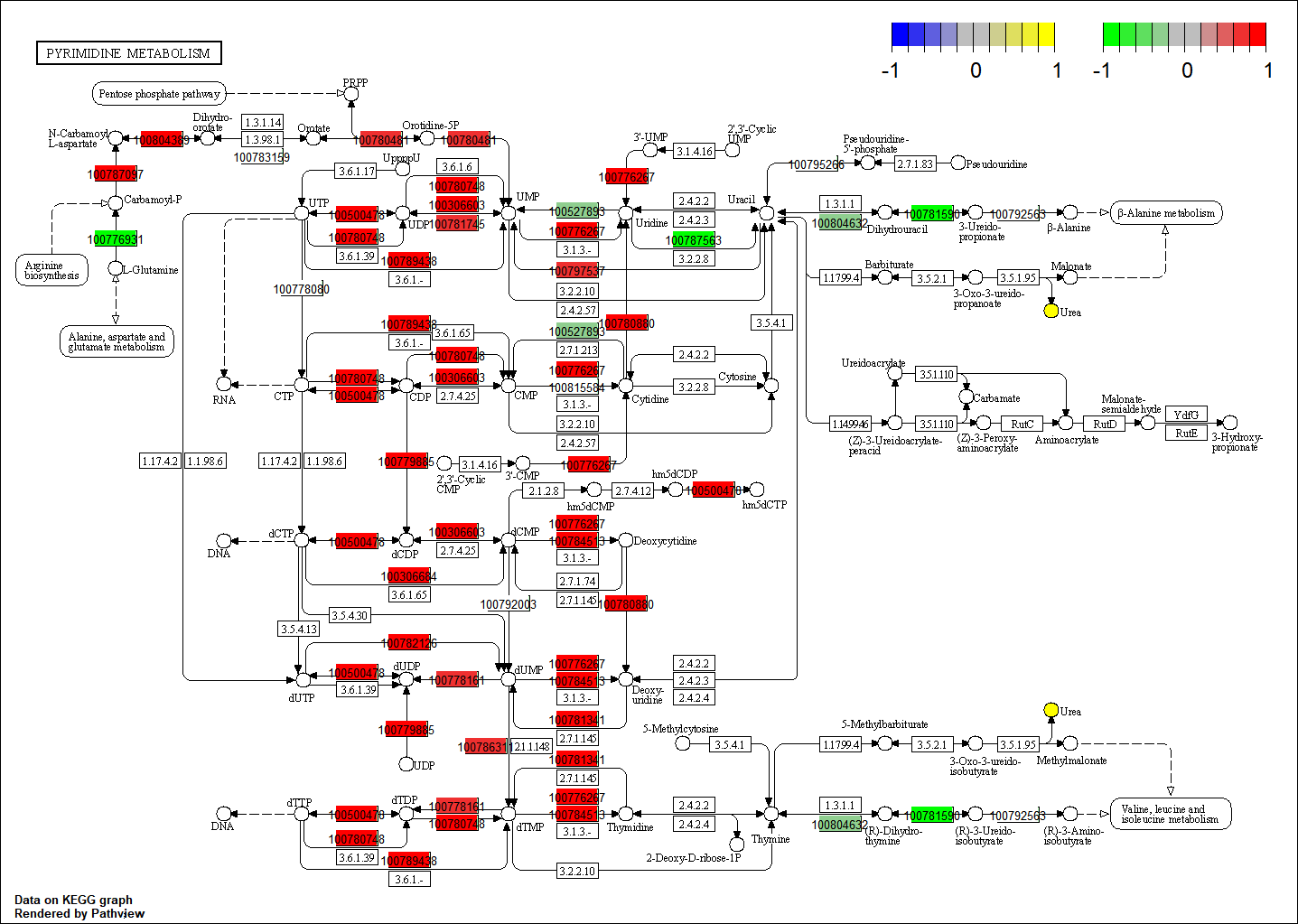

Supplement: Supplementary file 6 — Dataset S6 KEGG pathway maps for all pairwise comparisons; folder names correspond to specific treatment contrasts listed in heading of Dataset S6 in the main .docx document. [file NPH-250-2599-s003.zip › a.BPMV - Bj vs control, BPMV/up/gmx00240.pathview.png]

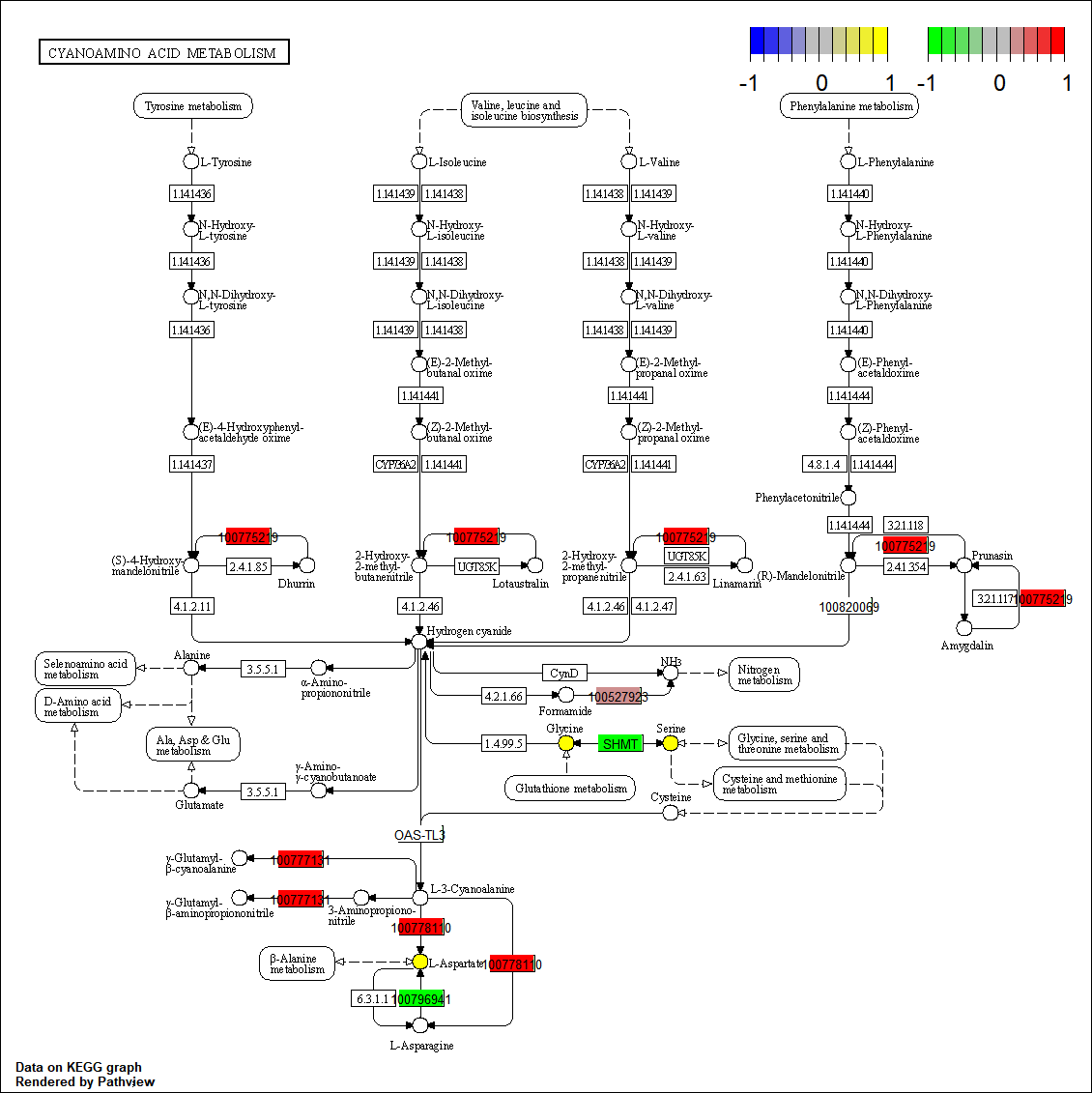

Supplement: Supplementary file 6 — Dataset S6 KEGG pathway maps for all pairwise comparisons; folder names correspond to specific treatment contrasts listed in heading of Dataset S6 in the main .docx document. [file NPH-250-2599-s003.zip › a.BPMV - Bj vs control, BPMV/up/gmx00460.pathview.png]

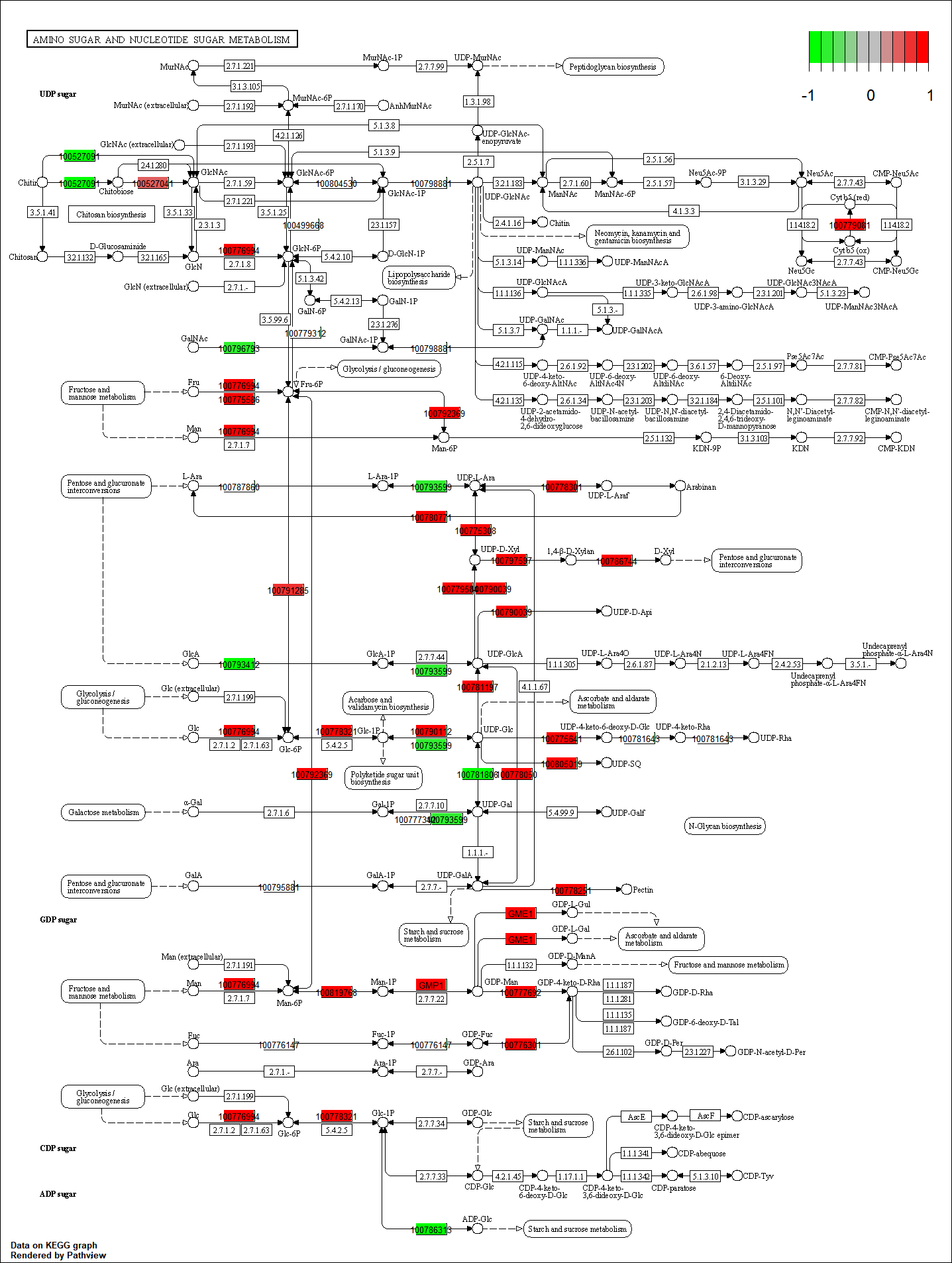

Supplement: Supplementary file 6 — Dataset S6 KEGG pathway maps for all pairwise comparisons; folder names correspond to specific treatment contrasts listed in heading of Dataset S6 in the main .docx document. [file NPH-250-2599-s003.zip › a.BPMV - Bj vs control, BPMV/up/gmx00520.pathview.png]

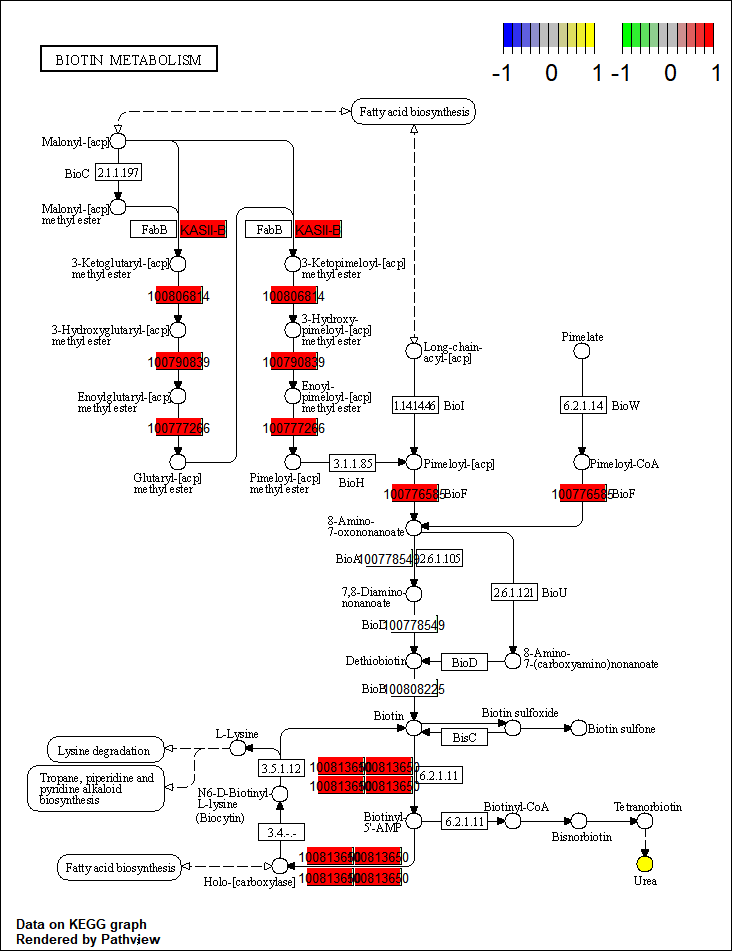

Supplement: Supplementary file 6 — Dataset S6 KEGG pathway maps for all pairwise comparisons; folder names correspond to specific treatment contrasts listed in heading of Dataset S6 in the main .docx document. [file NPH-250-2599-s003.zip › a.BPMV - Bj vs control, BPMV/up/gmx00780.pathview.png]

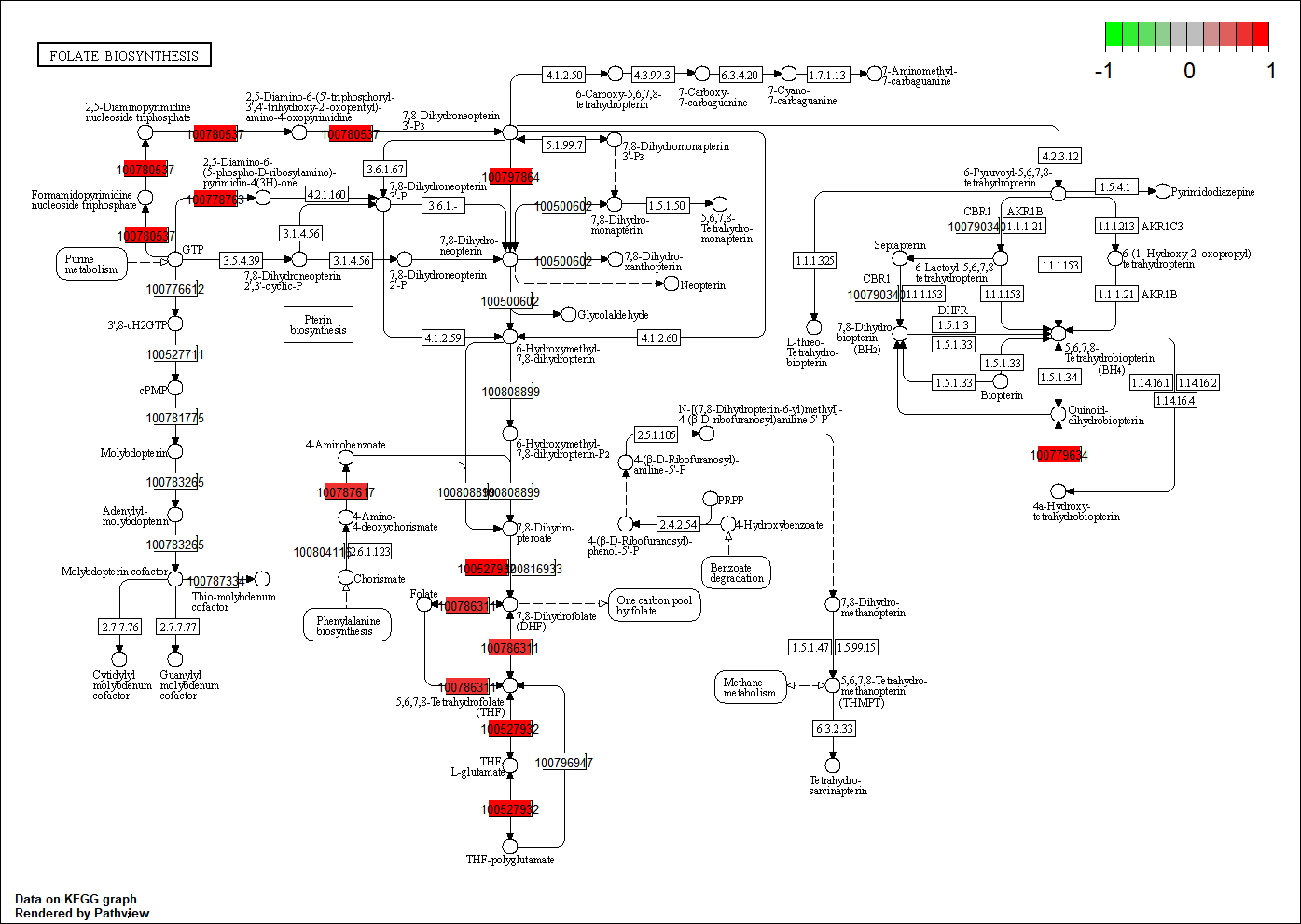

Supplement: Supplementary file 6 — Dataset S6 KEGG pathway maps for all pairwise comparisons; folder names correspond to specific treatment contrasts listed in heading of Dataset S6 in the main .docx document. [file NPH-250-2599-s003.zip › a.BPMV - Bj vs control, BPMV/up/gmx00790.pathview.png]

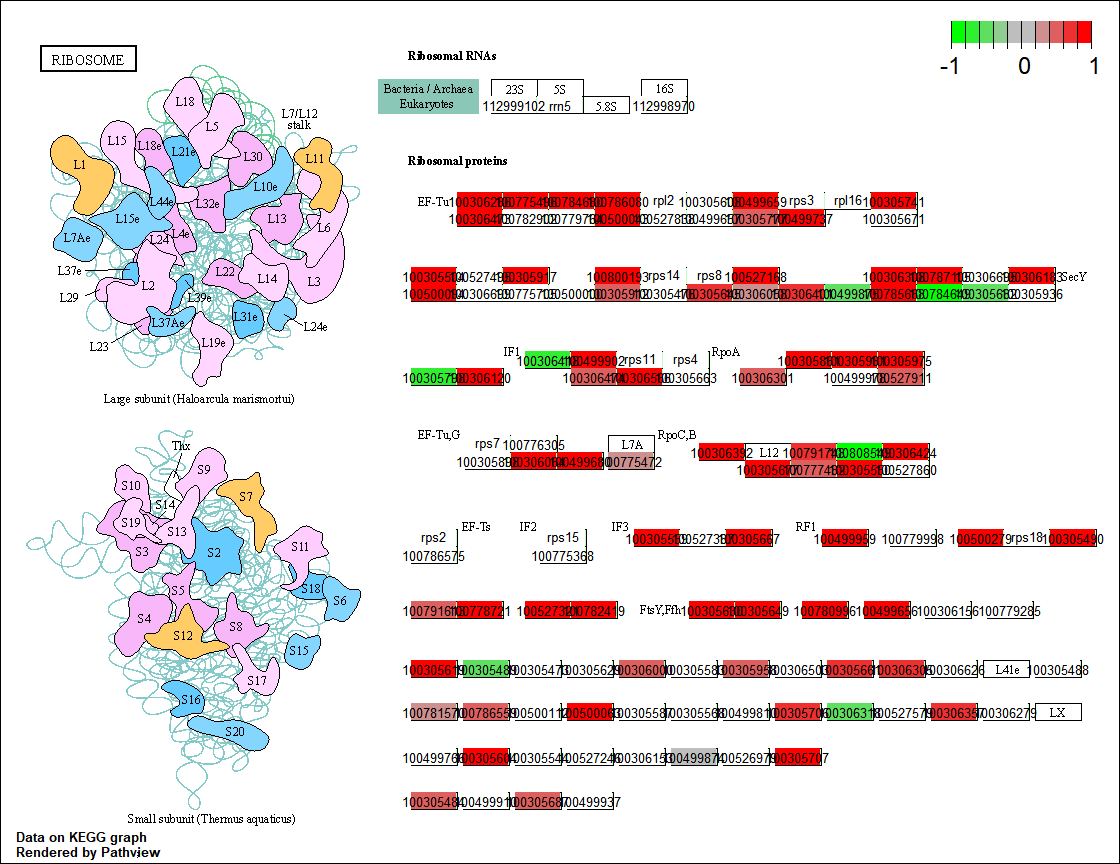

Supplement: Supplementary file 6 — Dataset S6 KEGG pathway maps for all pairwise comparisons; folder names correspond to specific treatment contrasts listed in heading of Dataset S6 in the main .docx document. [file NPH-250-2599-s003.zip › a.BPMV - Bj vs control, BPMV/up/gmx03010.pathview.png]

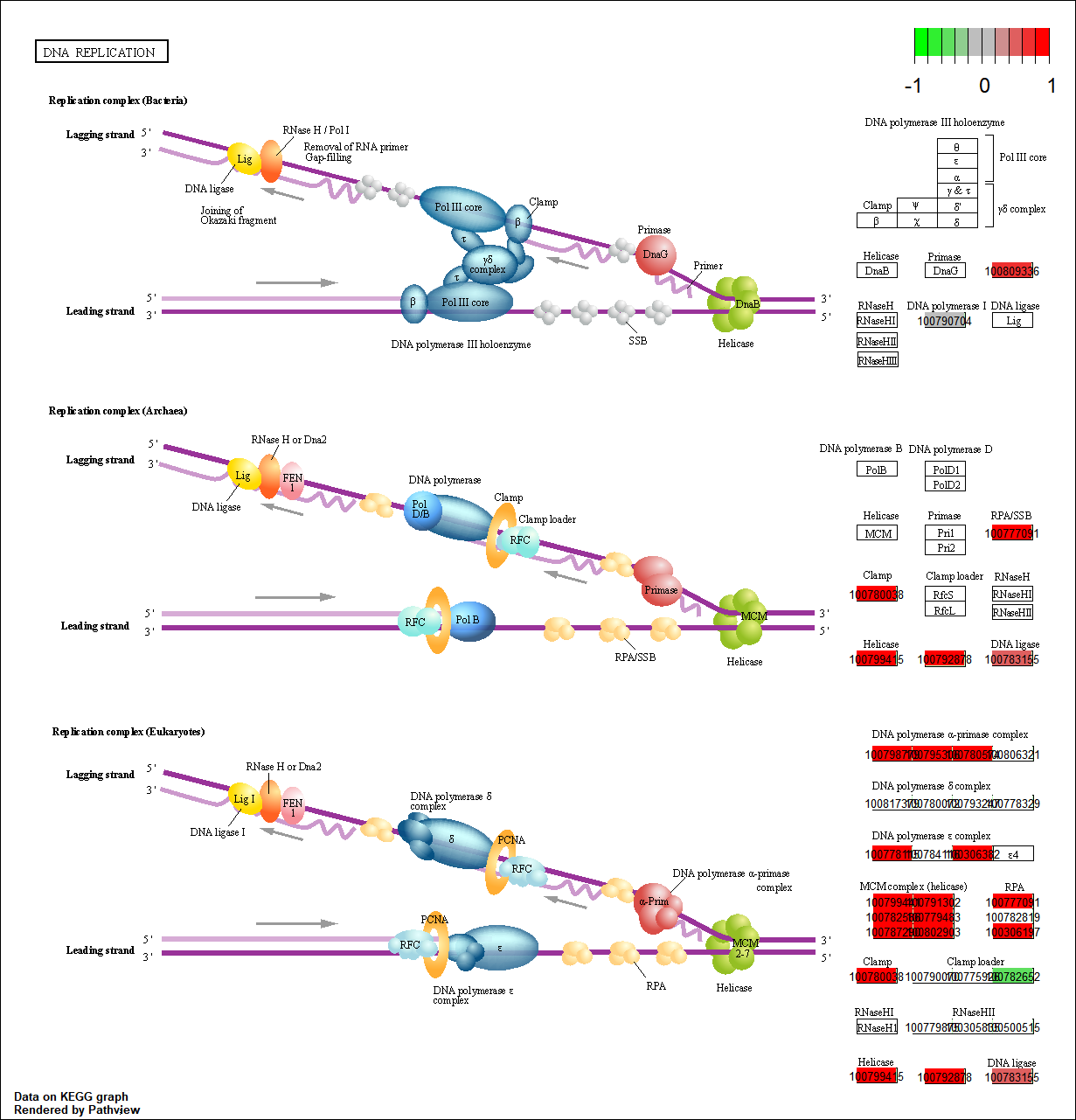

Supplement: Supplementary file 6 — Dataset S6 KEGG pathway maps for all pairwise comparisons; folder names correspond to specific treatment contrasts listed in heading of Dataset S6 in the main .docx document. [file NPH-250-2599-s003.zip › a.BPMV - Bj vs control, BPMV/up/gmx03030.pathview.png]

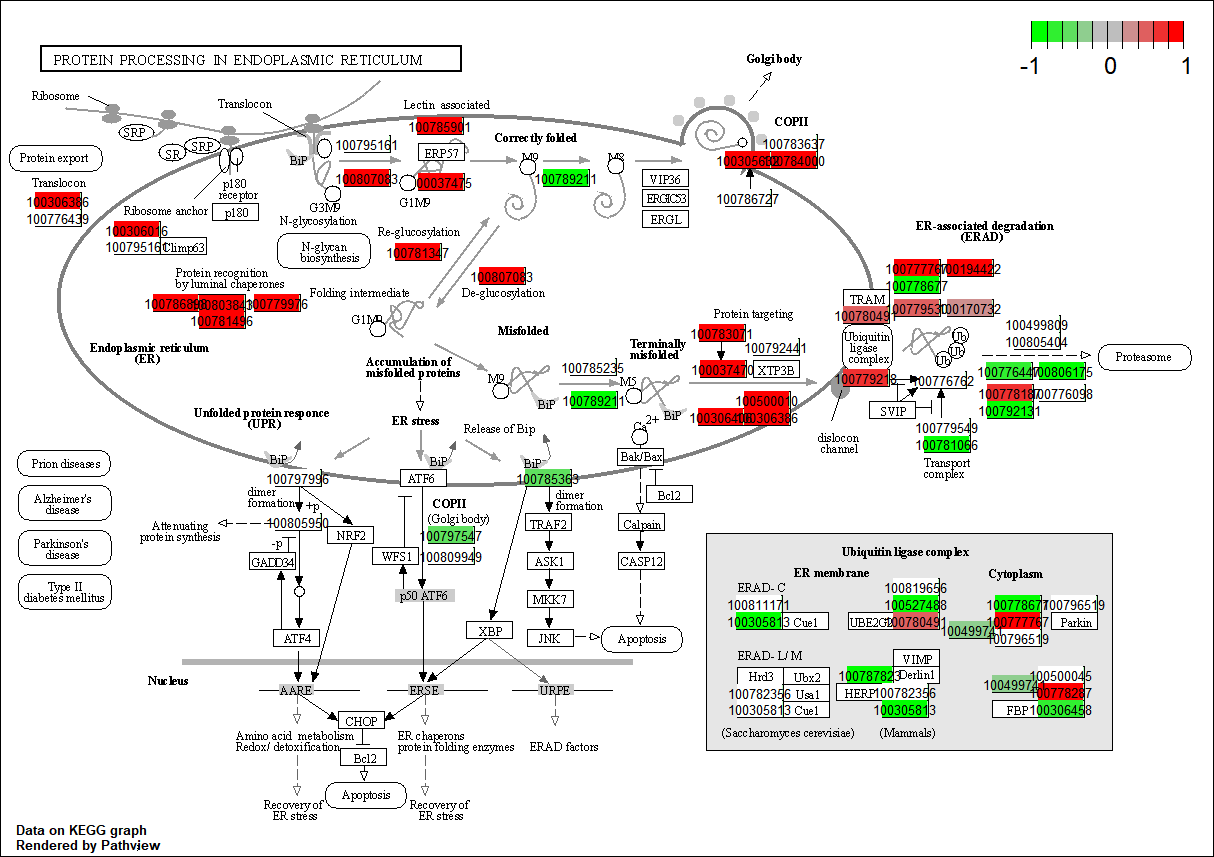

Supplement: Supplementary file 6 — Dataset S6 KEGG pathway maps for all pairwise comparisons; folder names correspond to specific treatment contrasts listed in heading of Dataset S6 in the main .docx document. [file NPH-250-2599-s003.zip › a.BPMV - Bj vs control, BPMV/up/gmx04141.pathview.png]

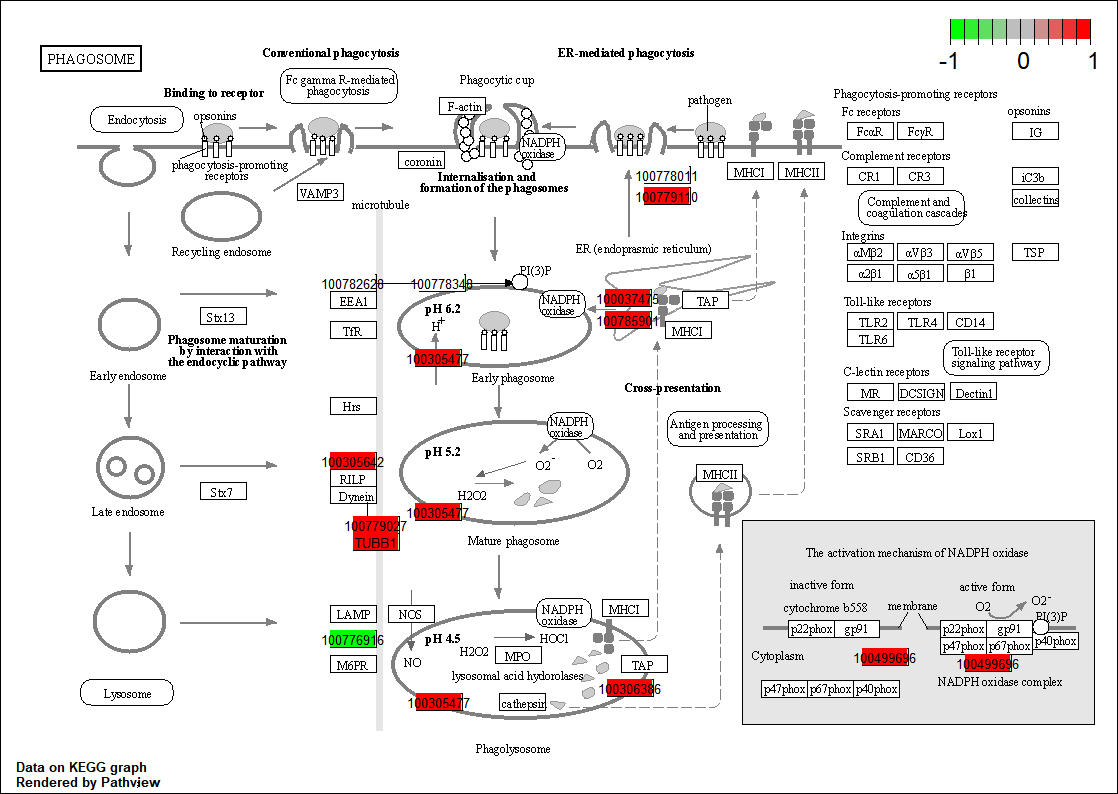

Supplement: Supplementary file 6 — Dataset S6 KEGG pathway maps for all pairwise comparisons; folder names correspond to specific treatment contrasts listed in heading of Dataset S6 in the main .docx document. [file NPH-250-2599-s003.zip › a.BPMV - Bj vs control, BPMV/up/gmx04145.pathview.png]

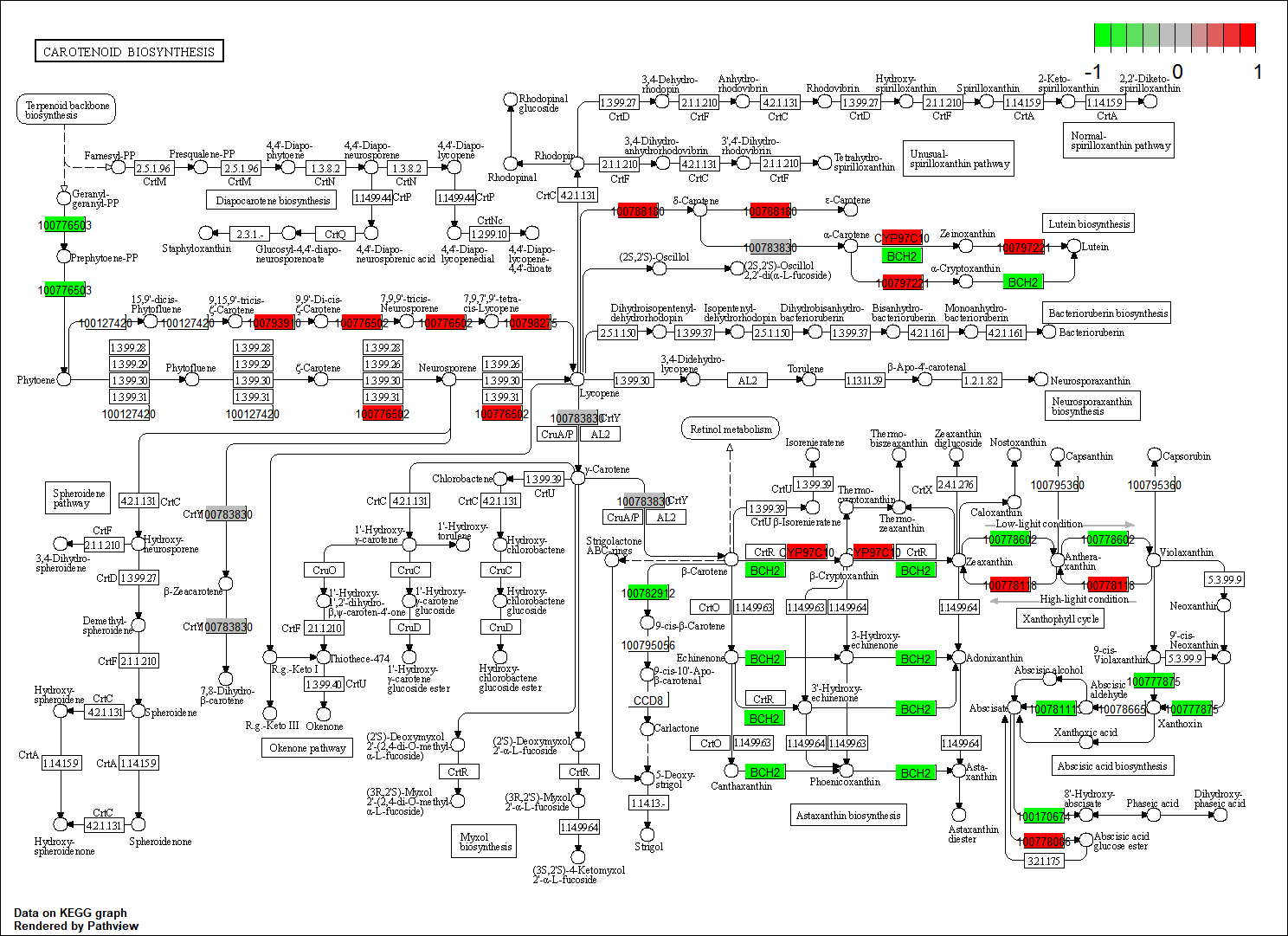

Supplement: Supplementary file 6 — Dataset S6 KEGG pathway maps for all pairwise comparisons; folder names correspond to specific treatment contrasts listed in heading of Dataset S6 in the main .docx document. [file NPH-250-2599-s003.zip › a.uninfected - Bj vs control, uninfected/down/gmx00906.pathview.png]

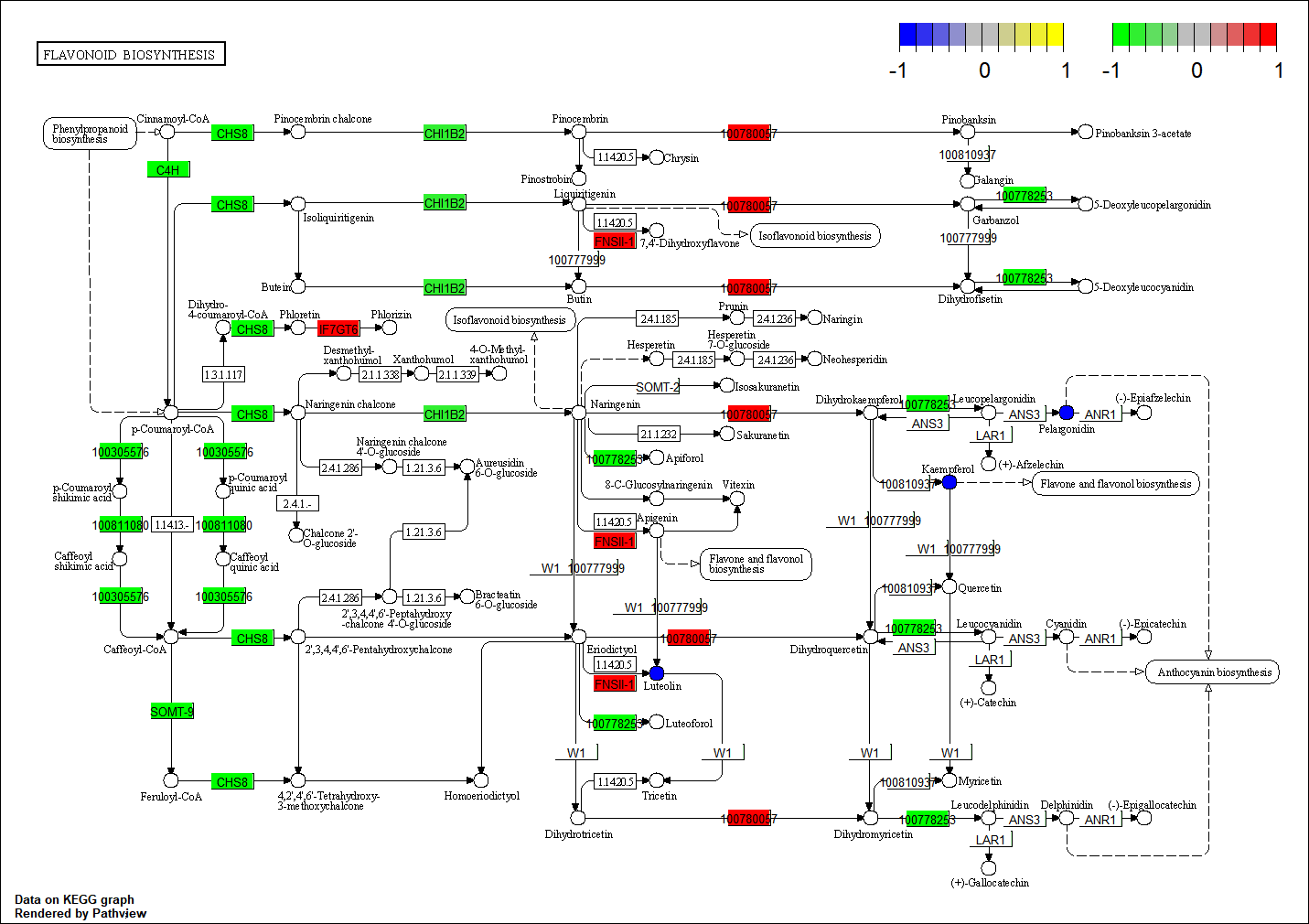

Supplement: Supplementary file 6 — Dataset S6 KEGG pathway maps for all pairwise comparisons; folder names correspond to specific treatment contrasts listed in heading of Dataset S6 in the main .docx document. [file NPH-250-2599-s003.zip › a.uninfected - Bj vs control, uninfected/down/gmx00941.pathview.png]

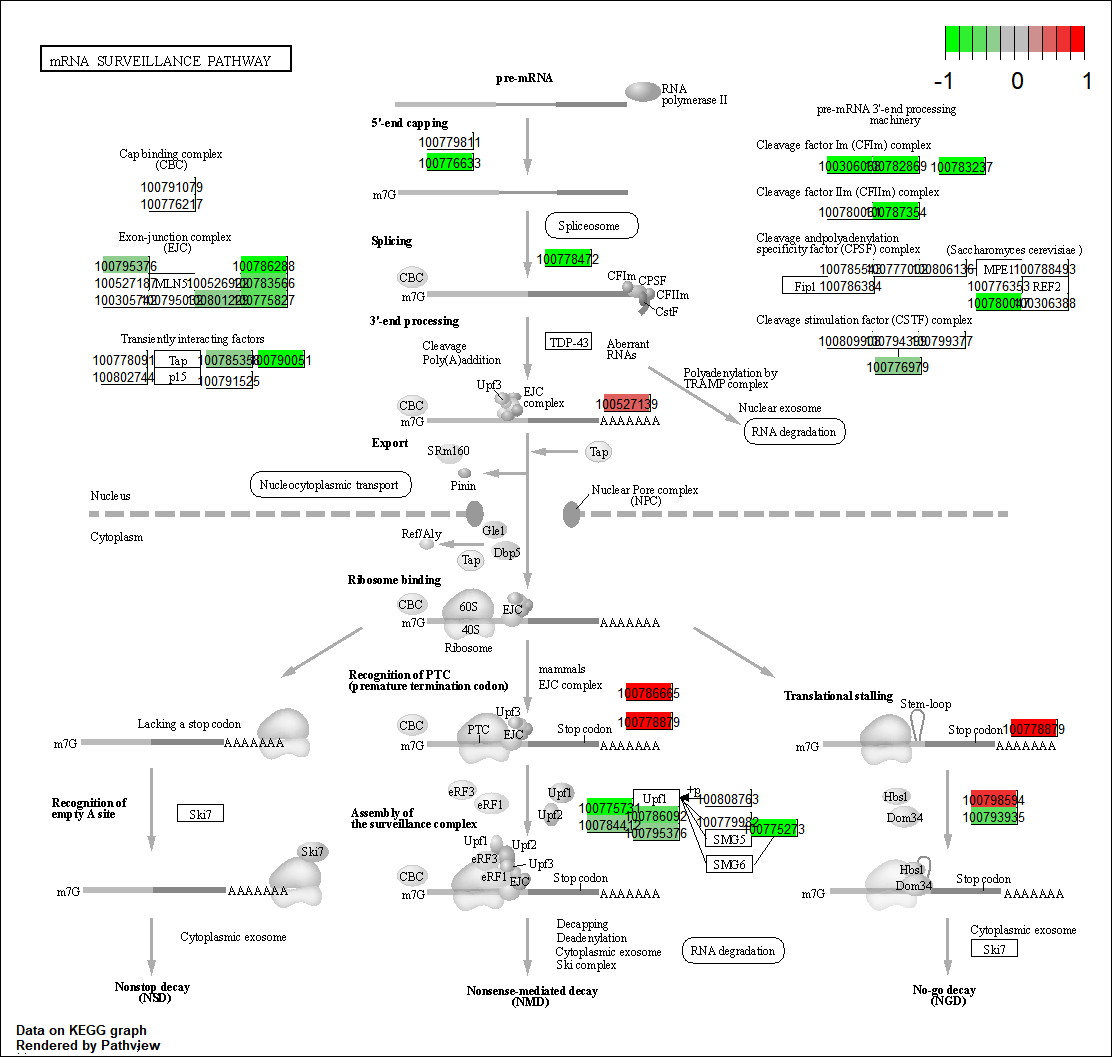

Supplement: Supplementary file 6 — Dataset S6 KEGG pathway maps for all pairwise comparisons; folder names correspond to specific treatment contrasts listed in heading of Dataset S6 in the main .docx document. [file NPH-250-2599-s003.zip › a.uninfected - Bj vs control, uninfected/down/gmx03015.pathview.png]

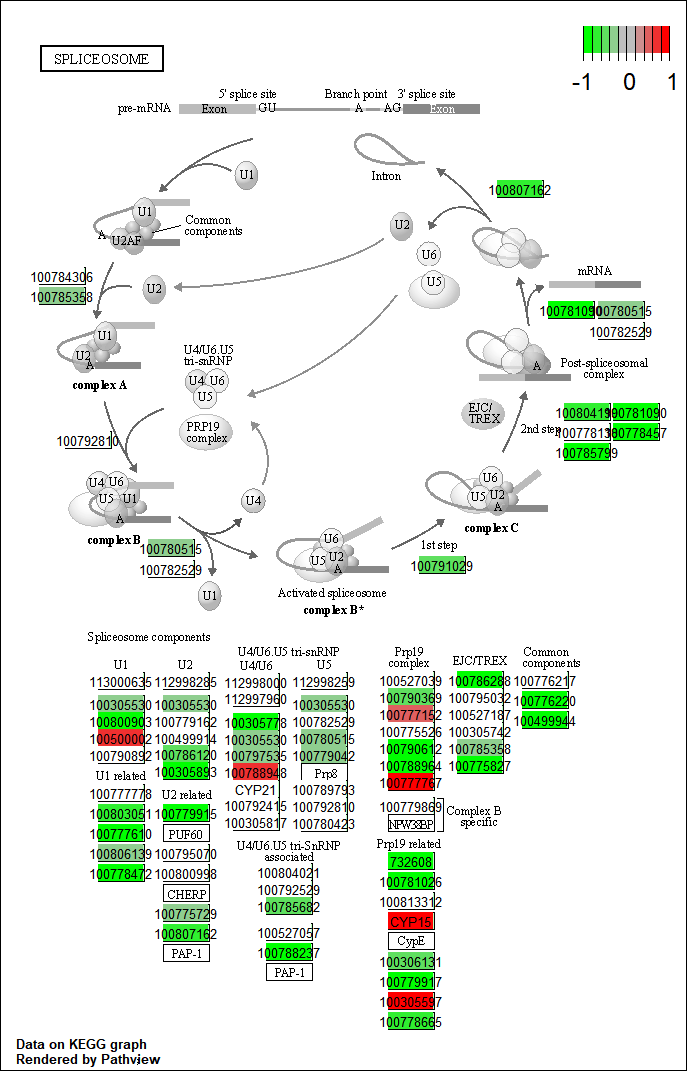

Supplement: Supplementary file 6 — Dataset S6 KEGG pathway maps for all pairwise comparisons; folder names correspond to specific treatment contrasts listed in heading of Dataset S6 in the main .docx document. [file NPH-250-2599-s003.zip › a.uninfected - Bj vs control, uninfected/down/gmx03040.pathview.png]

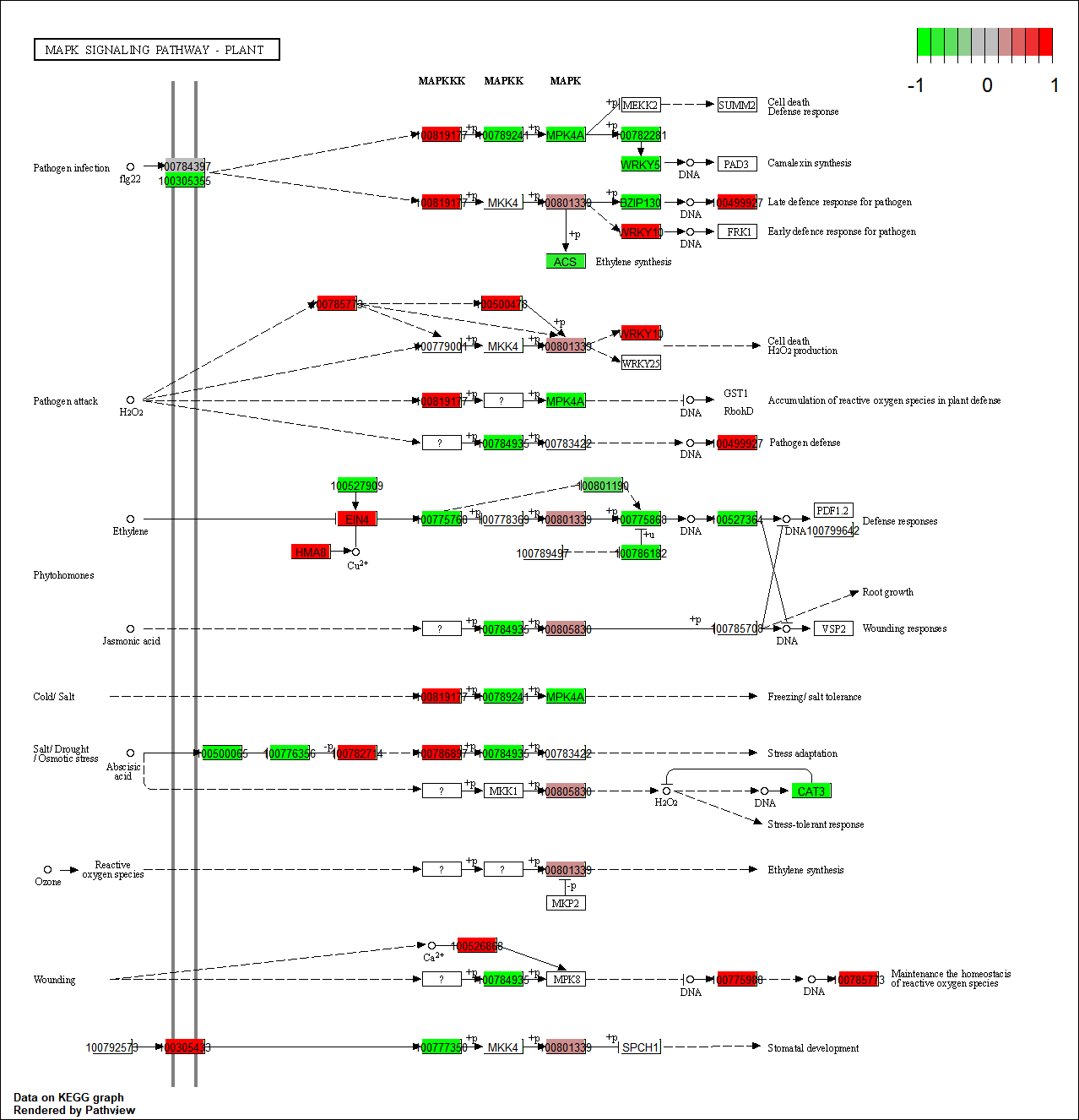

Supplement: Supplementary file 6 — Dataset S6 KEGG pathway maps for all pairwise comparisons; folder names correspond to specific treatment contrasts listed in heading of Dataset S6 in the main .docx document. [file NPH-250-2599-s003.zip › a.uninfected - Bj vs control, uninfected/down/gmx04016.pathview.png]

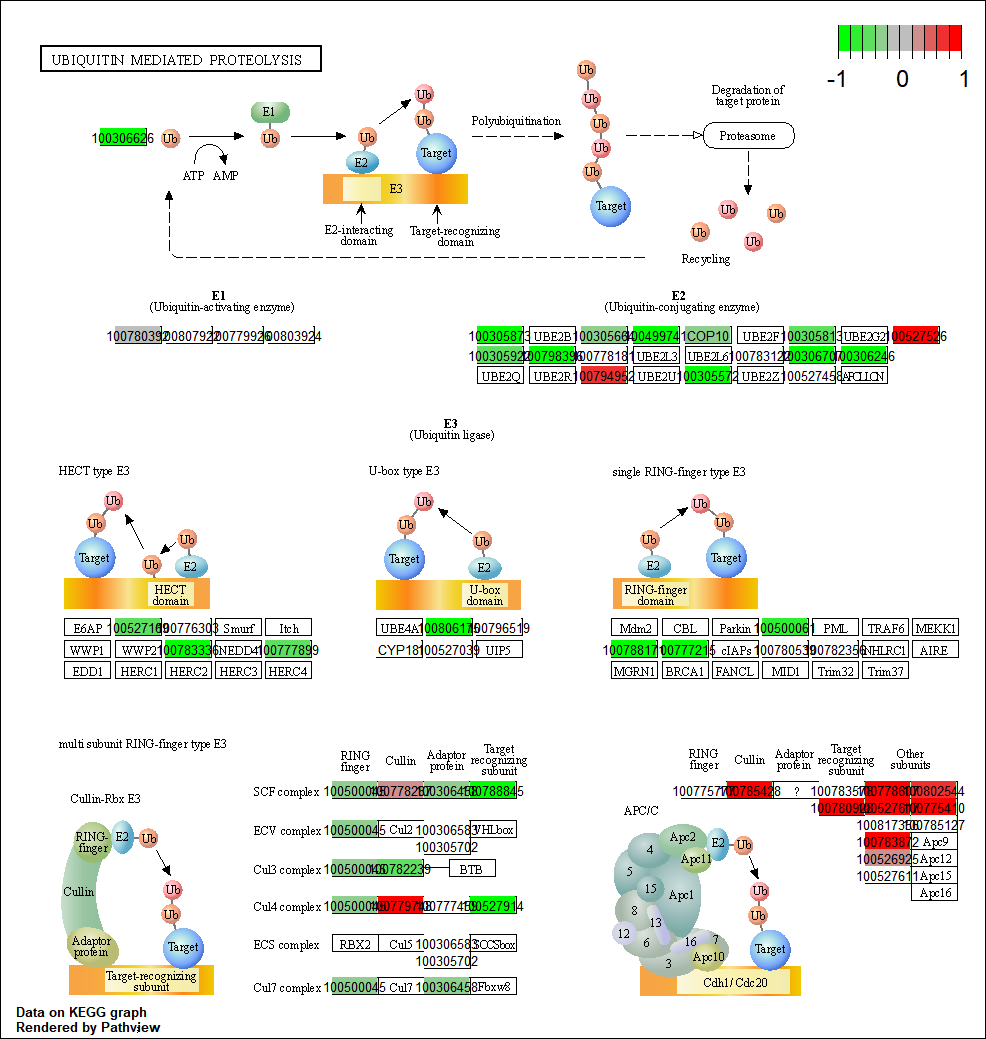

Supplement: Supplementary file 6 — Dataset S6 KEGG pathway maps for all pairwise comparisons; folder names correspond to specific treatment contrasts listed in heading of Dataset S6 in the main .docx document. [file NPH-250-2599-s003.zip › a.uninfected - Bj vs control, uninfected/down/gmx04120.pathview.png]

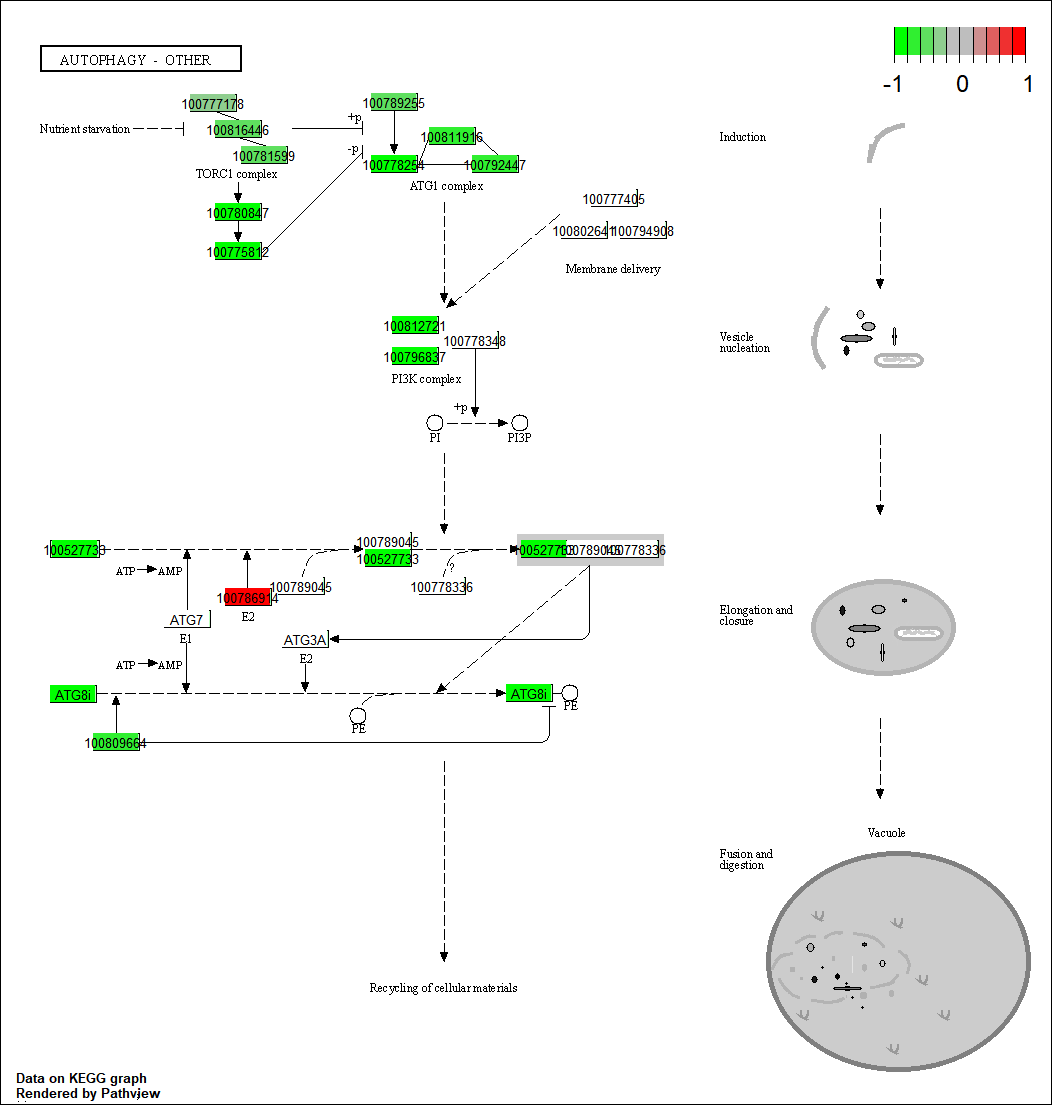

Supplement: Supplementary file 6 — Dataset S6 KEGG pathway maps for all pairwise comparisons; folder names correspond to specific treatment contrasts listed in heading of Dataset S6 in the main .docx document. [file NPH-250-2599-s003.zip › a.uninfected - Bj vs control, uninfected/down/gmx04136.pathview.png]

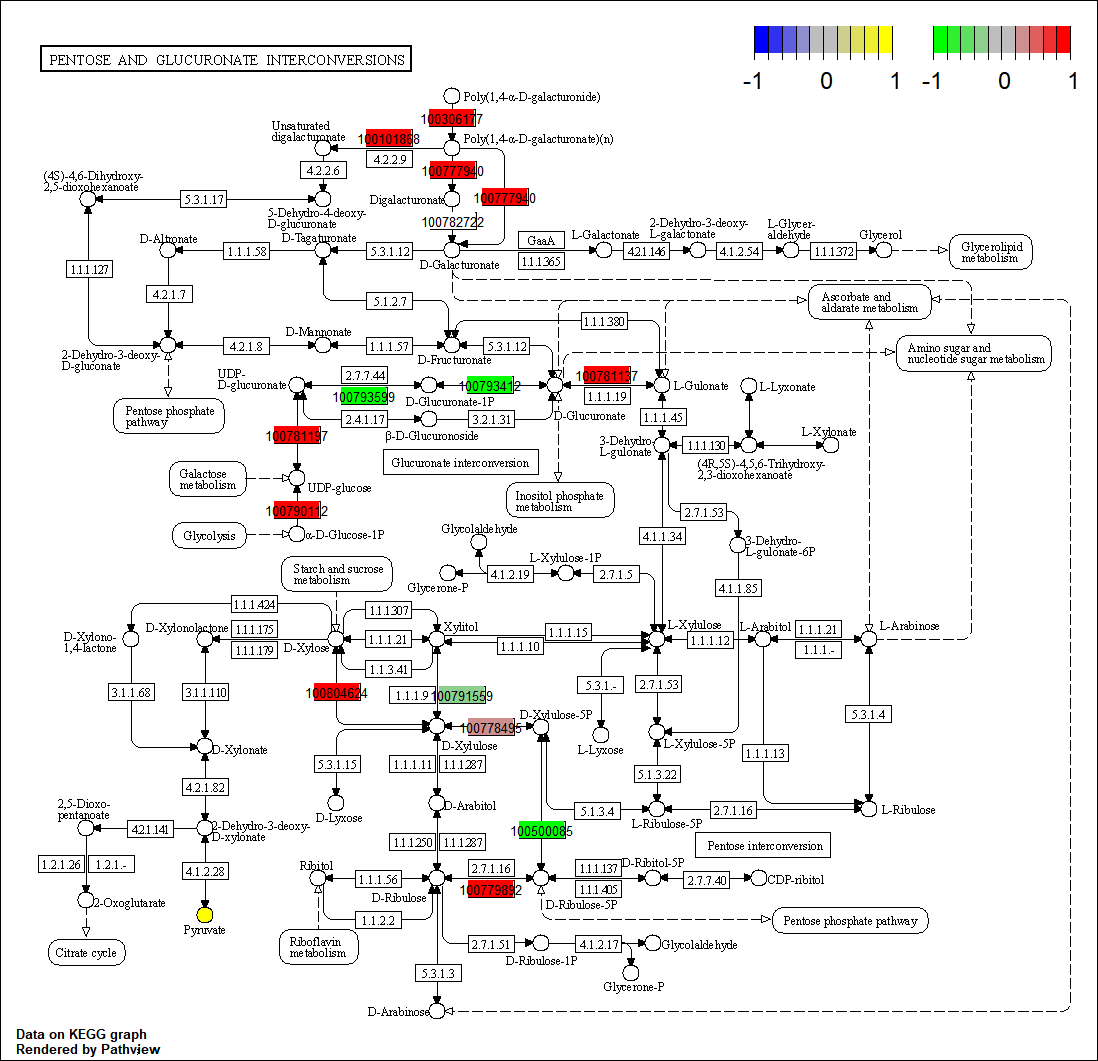

Supplement: Supplementary file 6 — Dataset S6 KEGG pathway maps for all pairwise comparisons; folder names correspond to specific treatment contrasts listed in heading of Dataset S6 in the main .docx document. [file NPH-250-2599-s003.zip › a.uninfected - Bj vs control, uninfected/up/gmx00040.pathview.png]

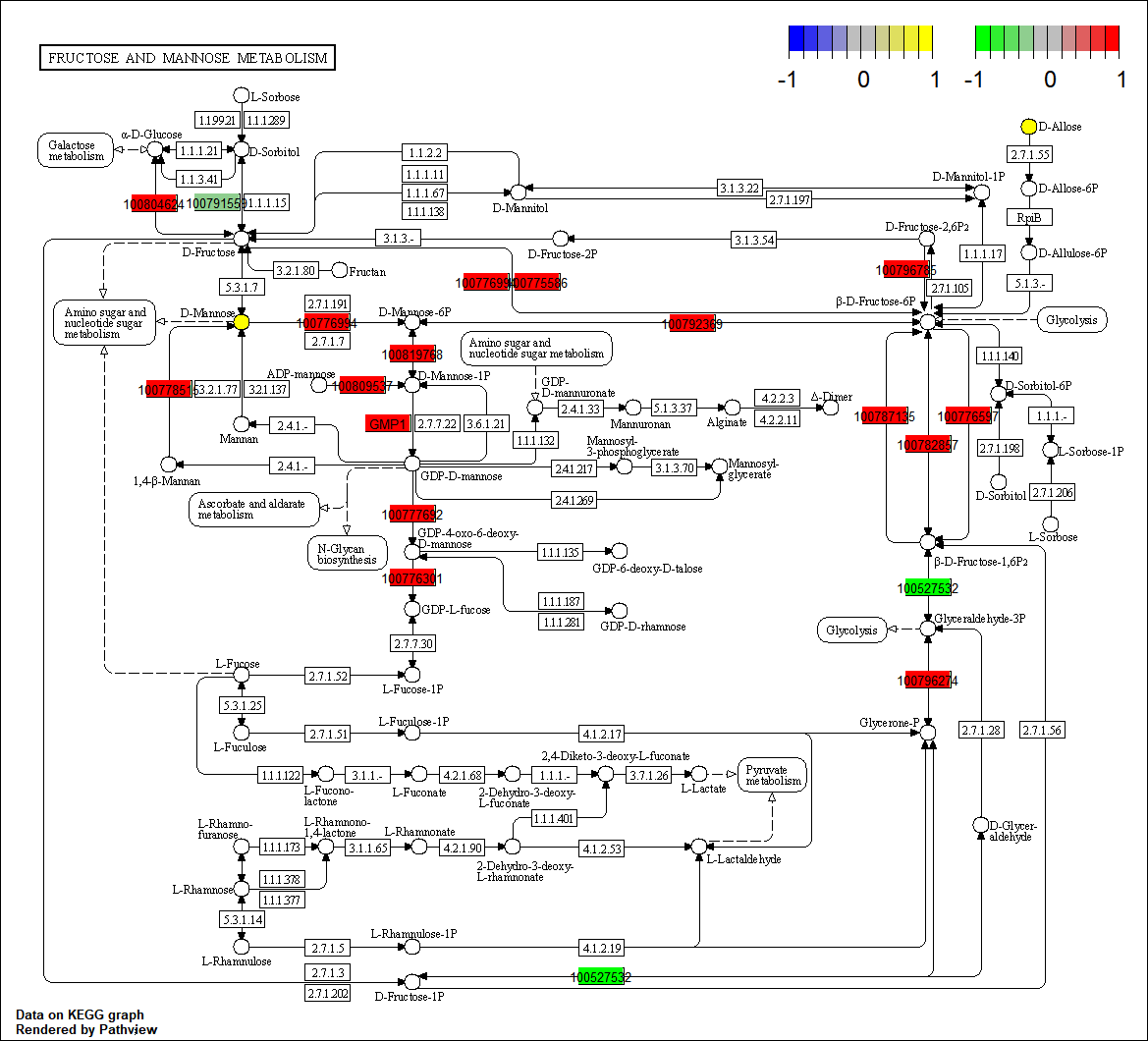

Supplement: Supplementary file 6 — Dataset S6 KEGG pathway maps for all pairwise comparisons; folder names correspond to specific treatment contrasts listed in heading of Dataset S6 in the main .docx document. [file NPH-250-2599-s003.zip › a.uninfected - Bj vs control, uninfected/up/gmx00051.pathview.png]

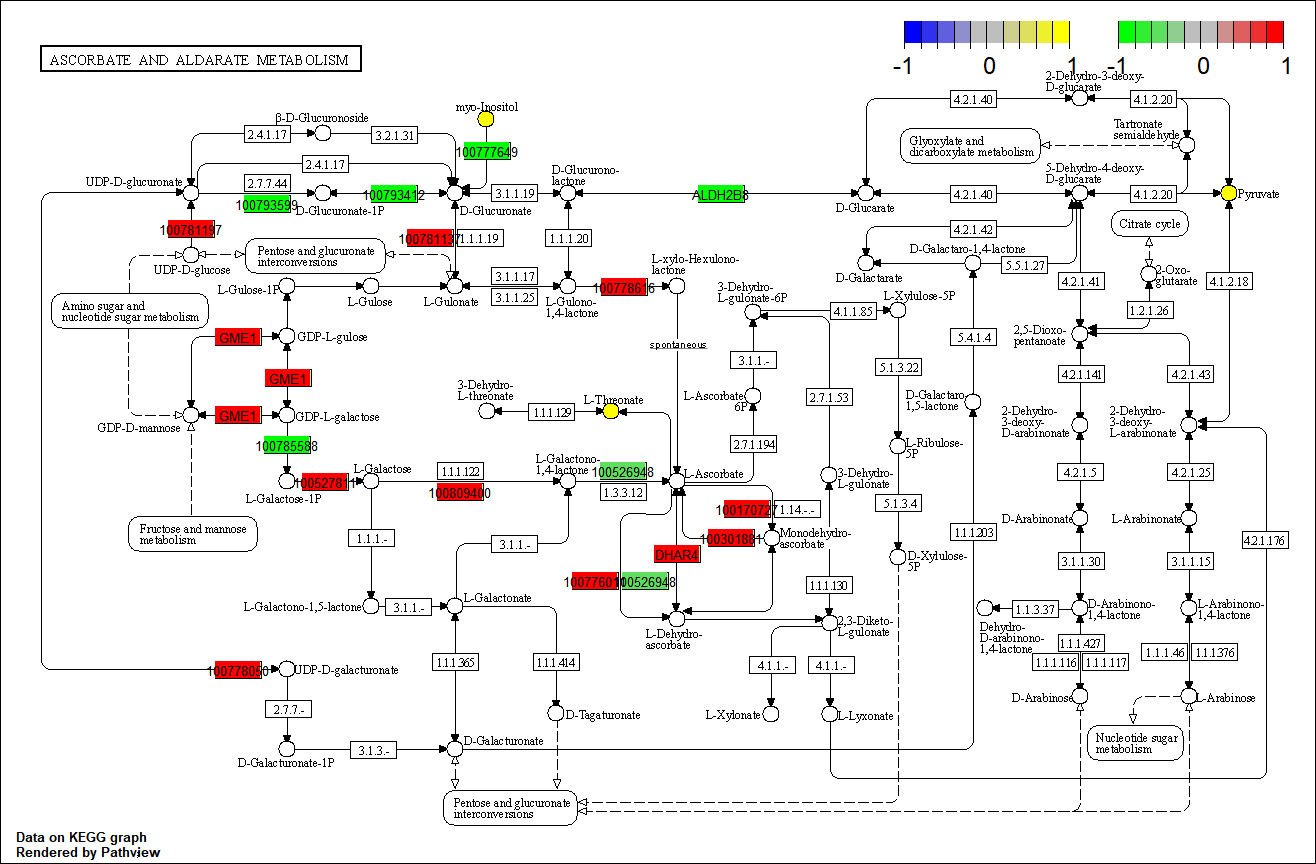

Supplement: Supplementary file 6 — Dataset S6 KEGG pathway maps for all pairwise comparisons; folder names correspond to specific treatment contrasts listed in heading of Dataset S6 in the main .docx document. [file NPH-250-2599-s003.zip › a.uninfected - Bj vs control, uninfected/up/gmx00053.pathview.png]

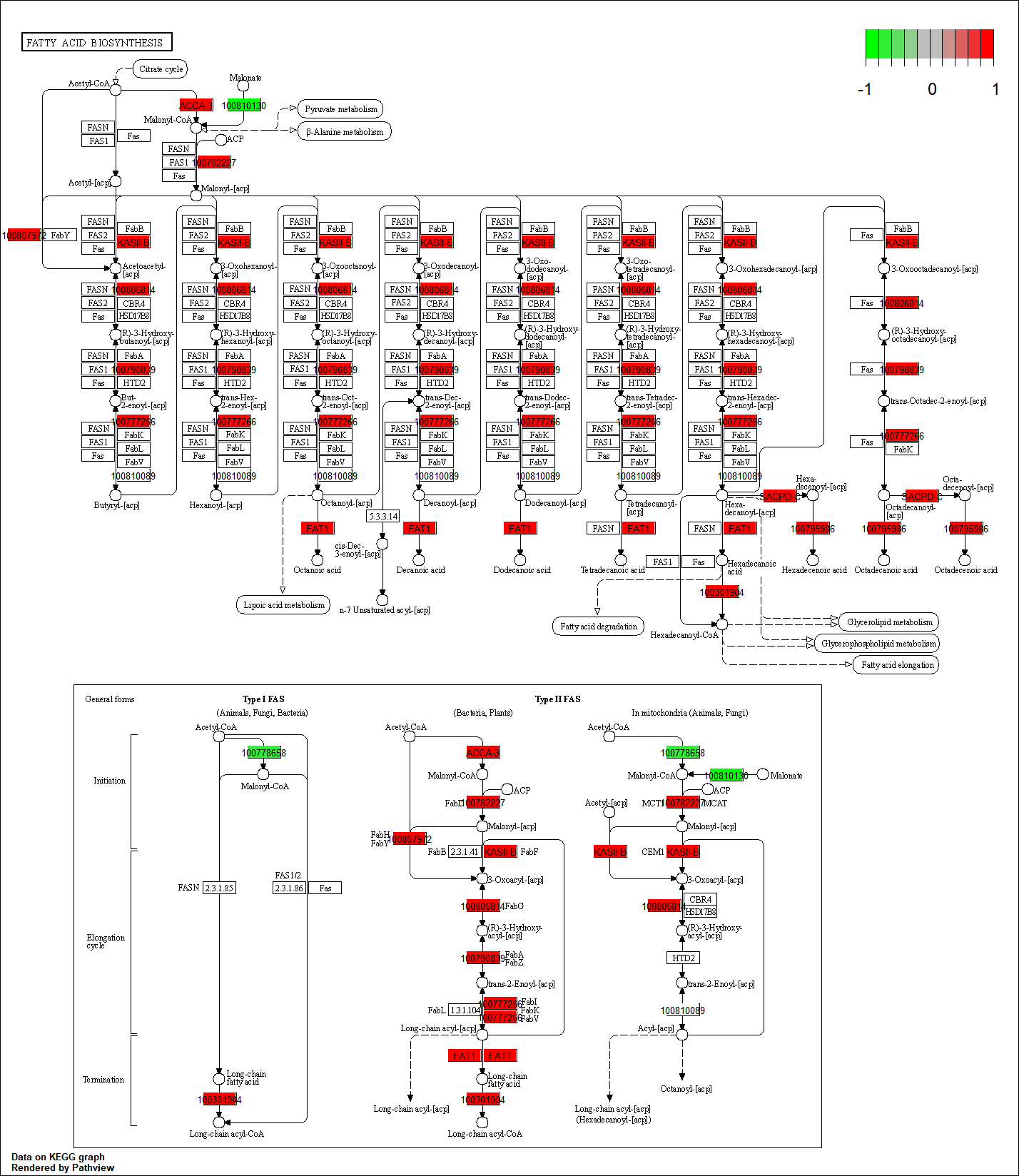

Supplement: Supplementary file 6 — Dataset S6 KEGG pathway maps for all pairwise comparisons; folder names correspond to specific treatment contrasts listed in heading of Dataset S6 in the main .docx document. [file NPH-250-2599-s003.zip › a.uninfected - Bj vs control, uninfected/up/gmx00061.pathview.png]

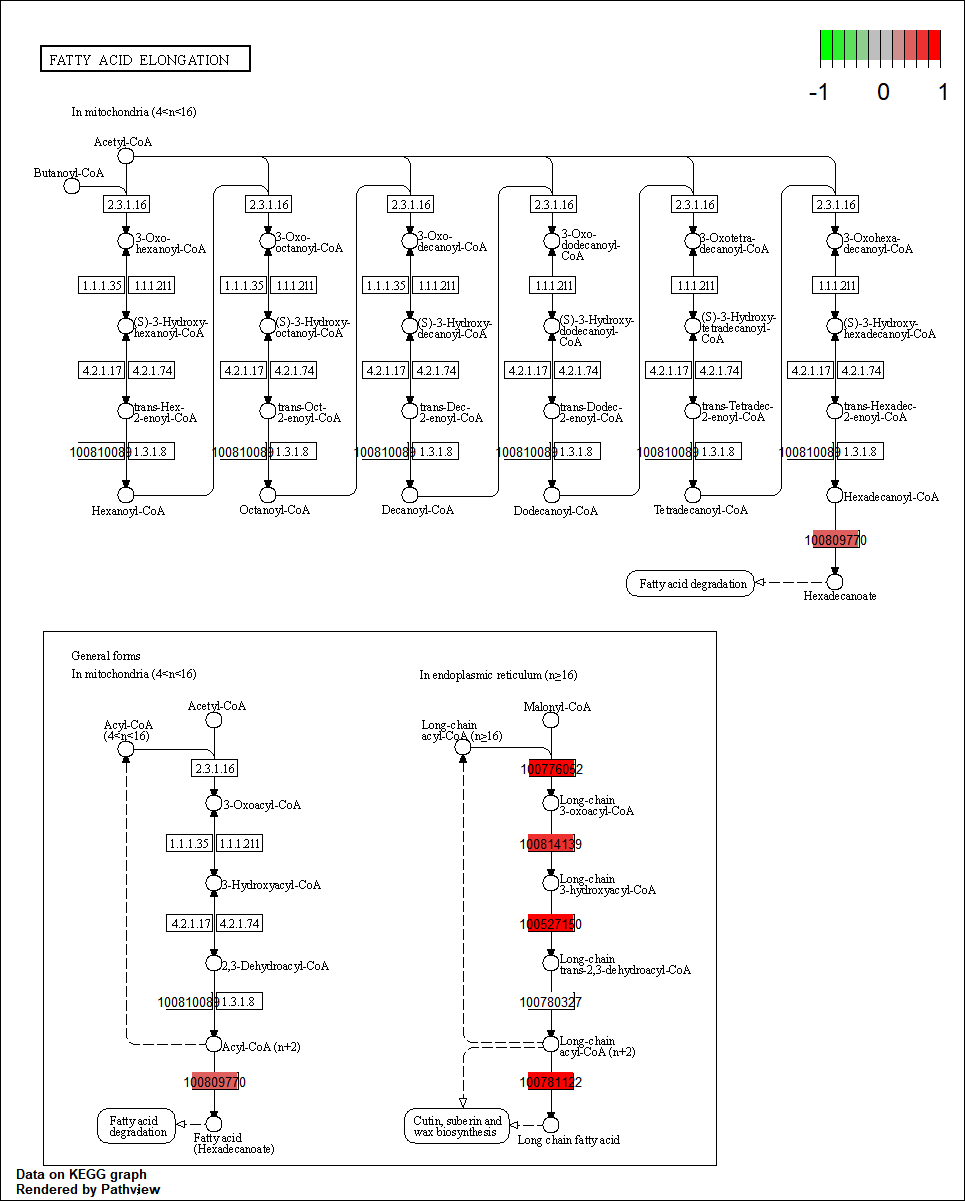

Supplement: Supplementary file 6 — Dataset S6 KEGG pathway maps for all pairwise comparisons; folder names correspond to specific treatment contrasts listed in heading of Dataset S6 in the main .docx document. [file NPH-250-2599-s003.zip › a.uninfected - Bj vs control, uninfected/up/gmx00062.pathview.png]

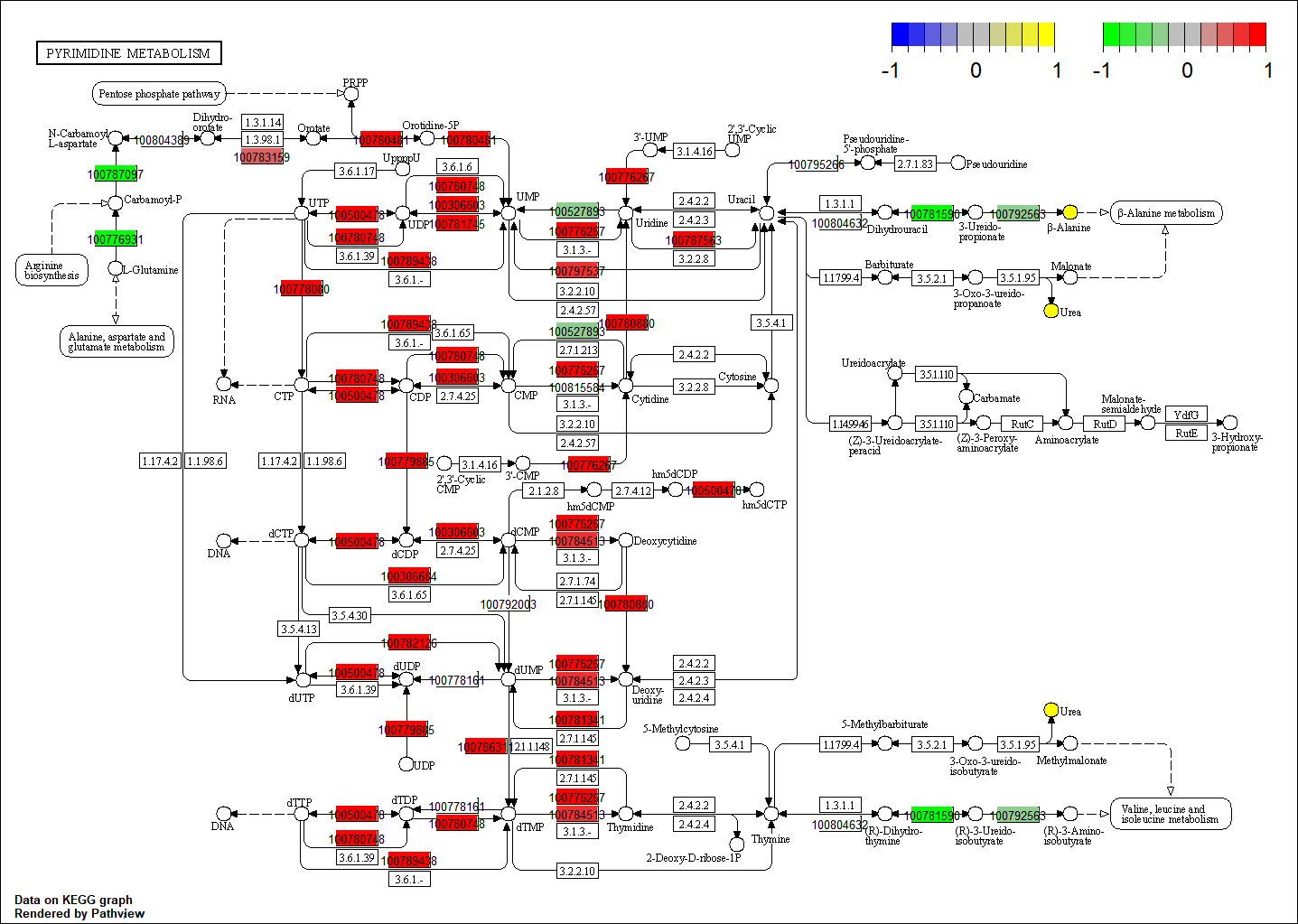

Supplement: Supplementary file 6 — Dataset S6 KEGG pathway maps for all pairwise comparisons; folder names correspond to specific treatment contrasts listed in heading of Dataset S6 in the main .docx document. [file NPH-250-2599-s003.zip › a.uninfected - Bj vs control, uninfected/up/gmx00240.pathview.png]

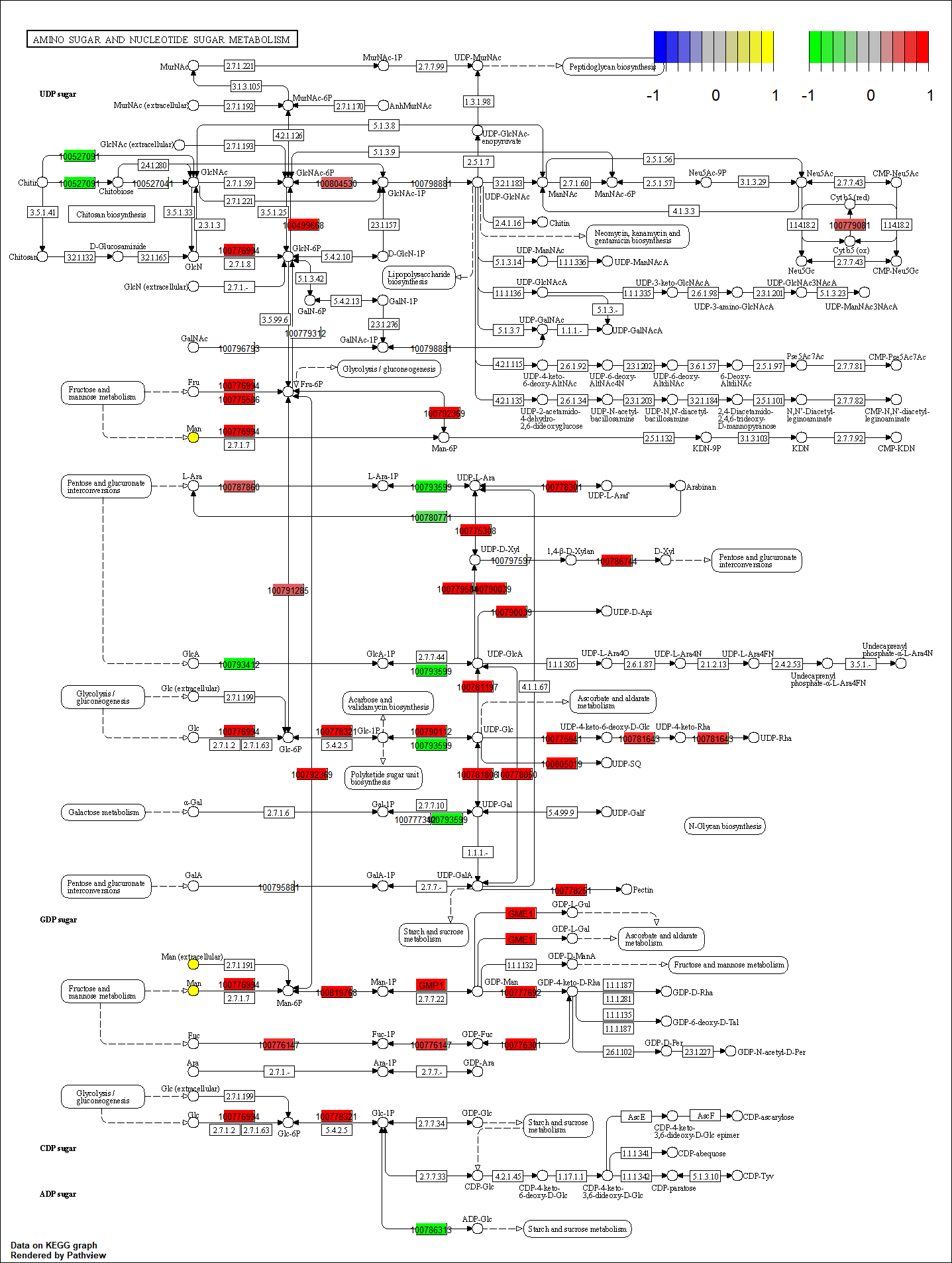

Supplement: Supplementary file 6 — Dataset S6 KEGG pathway maps for all pairwise comparisons; folder names correspond to specific treatment contrasts listed in heading of Dataset S6 in the main .docx document. [file NPH-250-2599-s003.zip › a.uninfected - Bj vs control, uninfected/up/gmx00520.pathview.png]

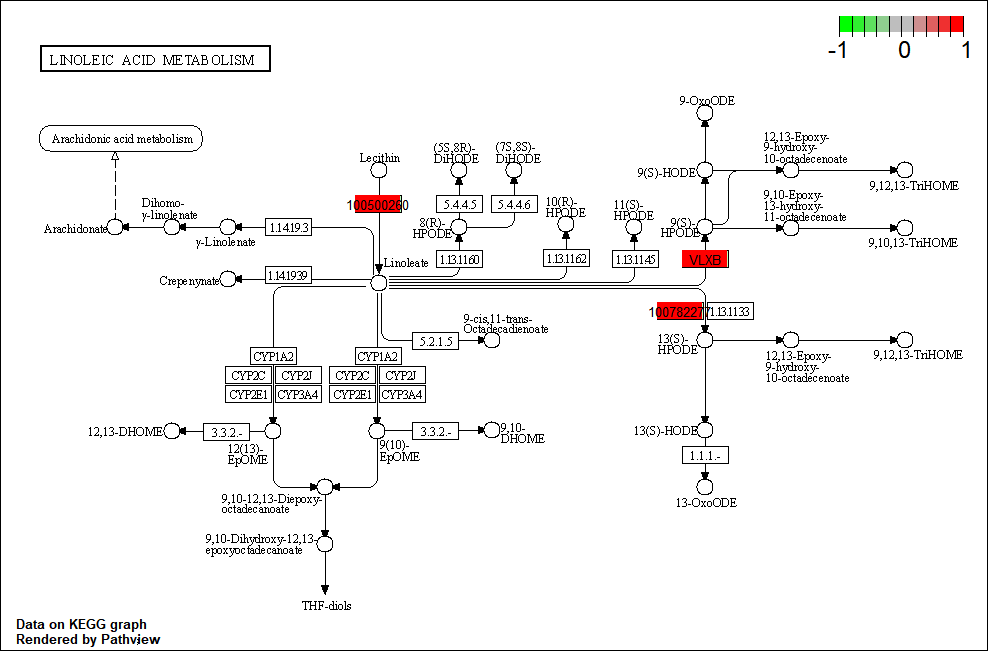

Supplement: Supplementary file 6 — Dataset S6 KEGG pathway maps for all pairwise comparisons; folder names correspond to specific treatment contrasts listed in heading of Dataset S6 in the main .docx document. [file NPH-250-2599-s003.zip › a.uninfected - Bj vs control, uninfected/up/gmx00591.pathview.png]

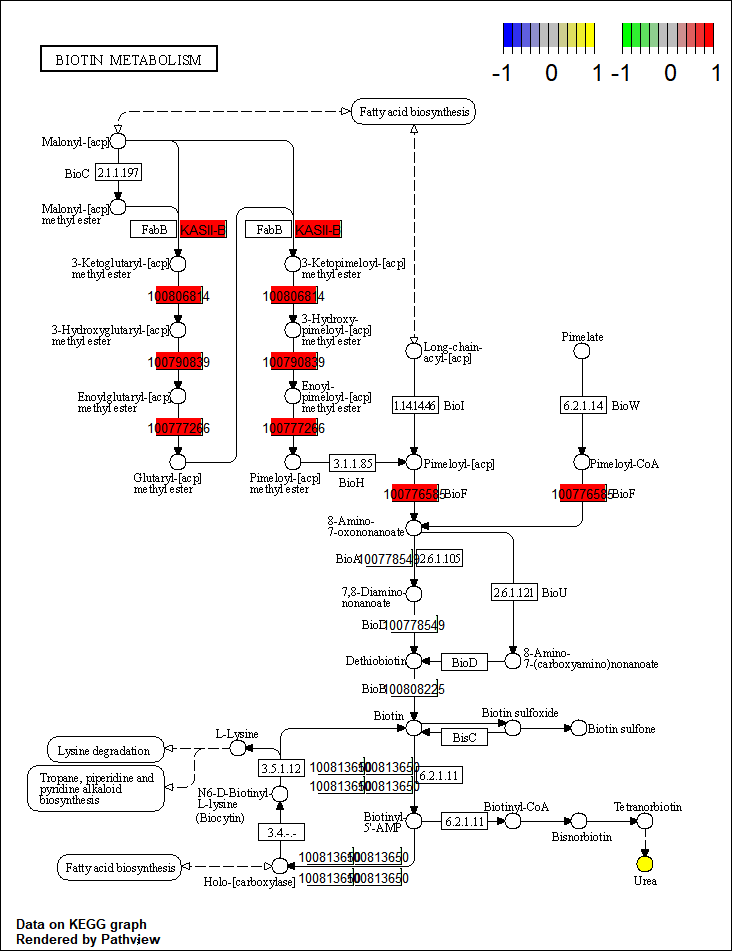

Supplement: Supplementary file 6 — Dataset S6 KEGG pathway maps for all pairwise comparisons; folder names correspond to specific treatment contrasts listed in heading of Dataset S6 in the main .docx document. [file NPH-250-2599-s003.zip › a.uninfected - Bj vs control, uninfected/up/gmx00780.pathview.png]

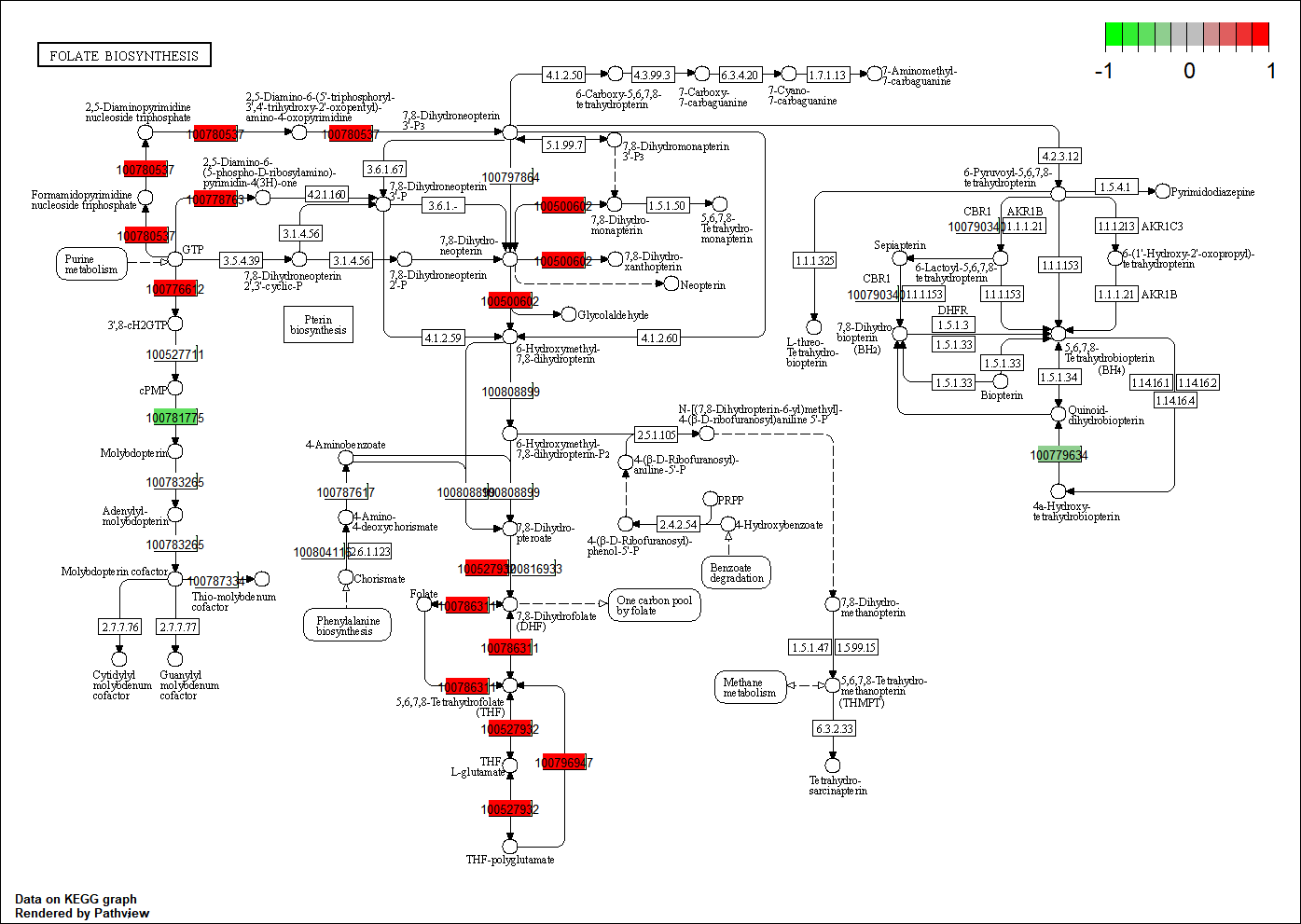

Supplement: Supplementary file 6 — Dataset S6 KEGG pathway maps for all pairwise comparisons; folder names correspond to specific treatment contrasts listed in heading of Dataset S6 in the main .docx document. [file NPH-250-2599-s003.zip › a.uninfected - Bj vs control, uninfected/up/gmx00790.pathview.png]

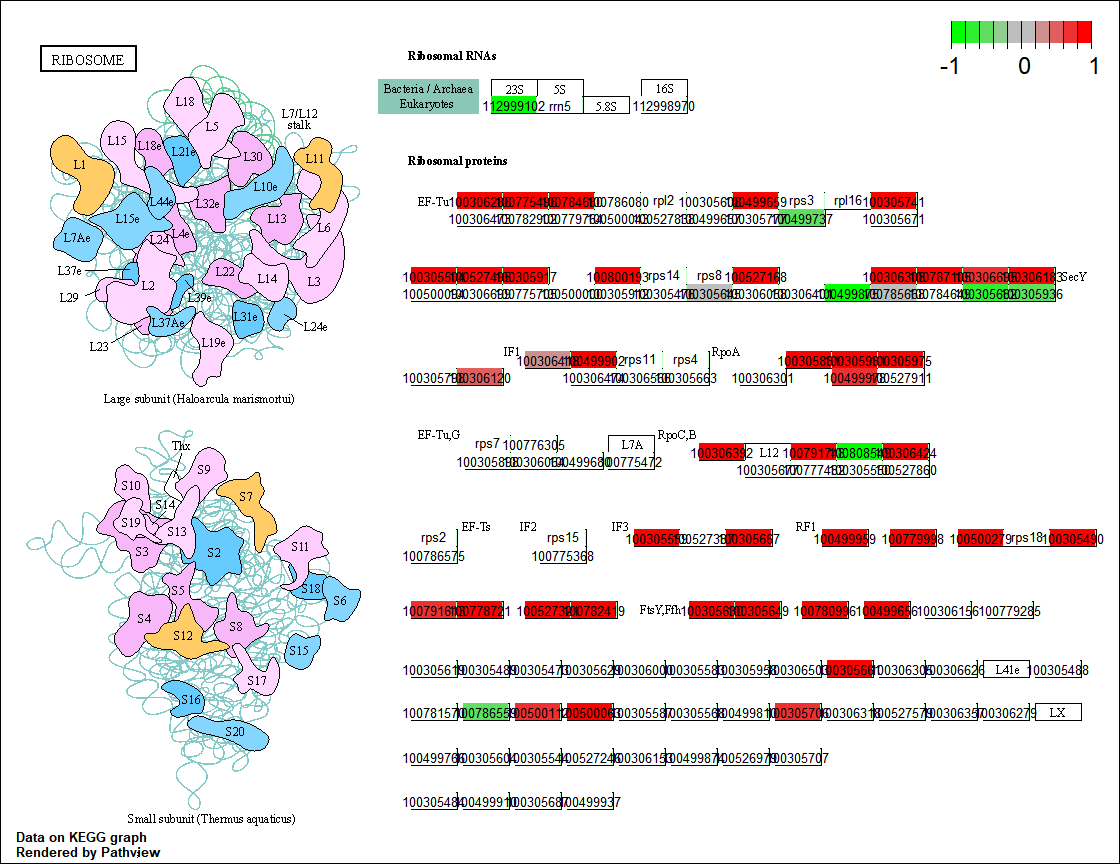

Supplement: Supplementary file 6 — Dataset S6 KEGG pathway maps for all pairwise comparisons; folder names correspond to specific treatment contrasts listed in heading of Dataset S6 in the main .docx document. [file NPH-250-2599-s003.zip › a.uninfected - Bj vs control, uninfected/up/gmx03010.pathview.png]

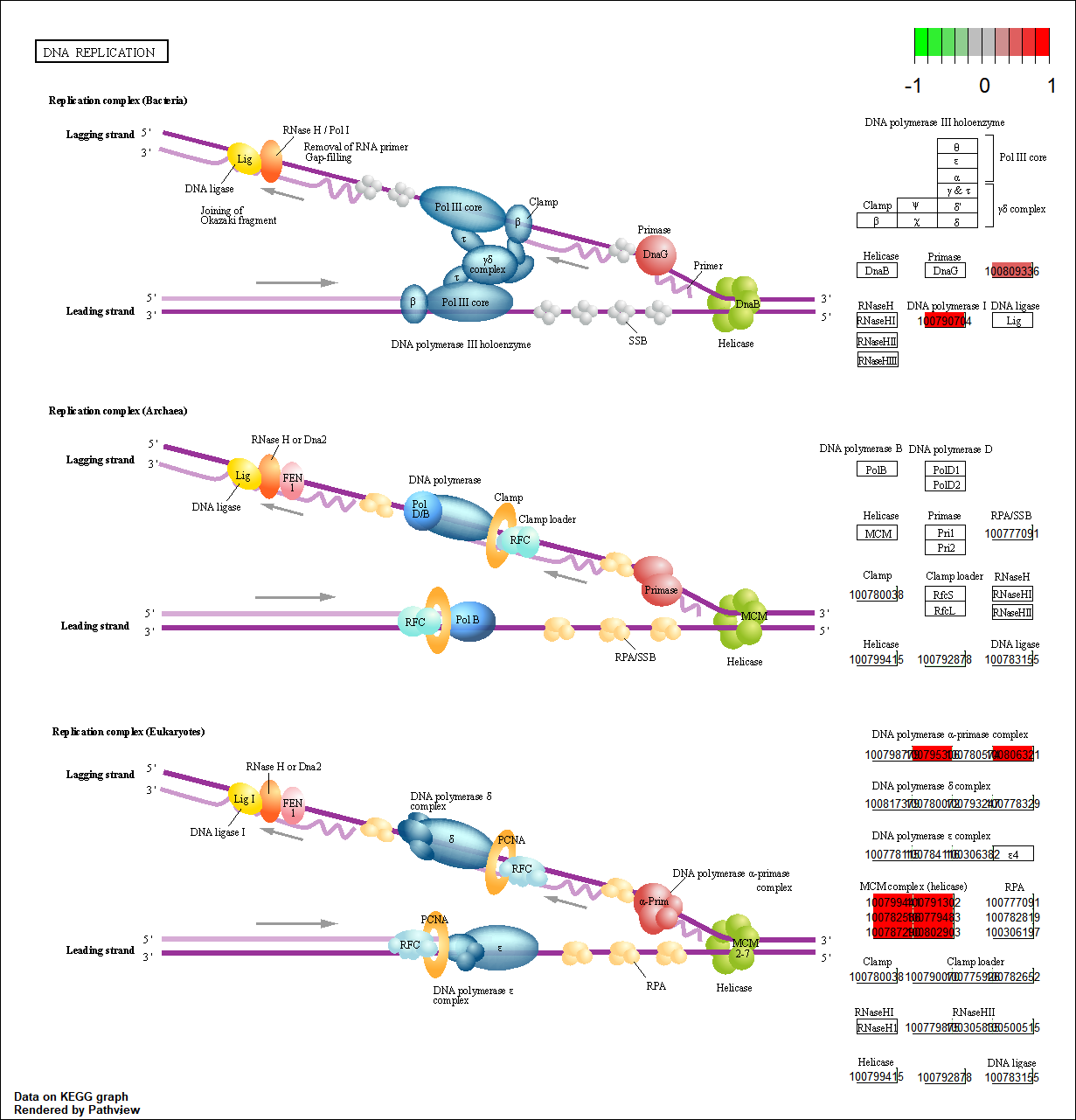

Supplement: Supplementary file 6 — Dataset S6 KEGG pathway maps for all pairwise comparisons; folder names correspond to specific treatment contrasts listed in heading of Dataset S6 in the main .docx document. [file NPH-250-2599-s003.zip › a.uninfected - Bj vs control, uninfected/up/gmx03030.pathview.png]

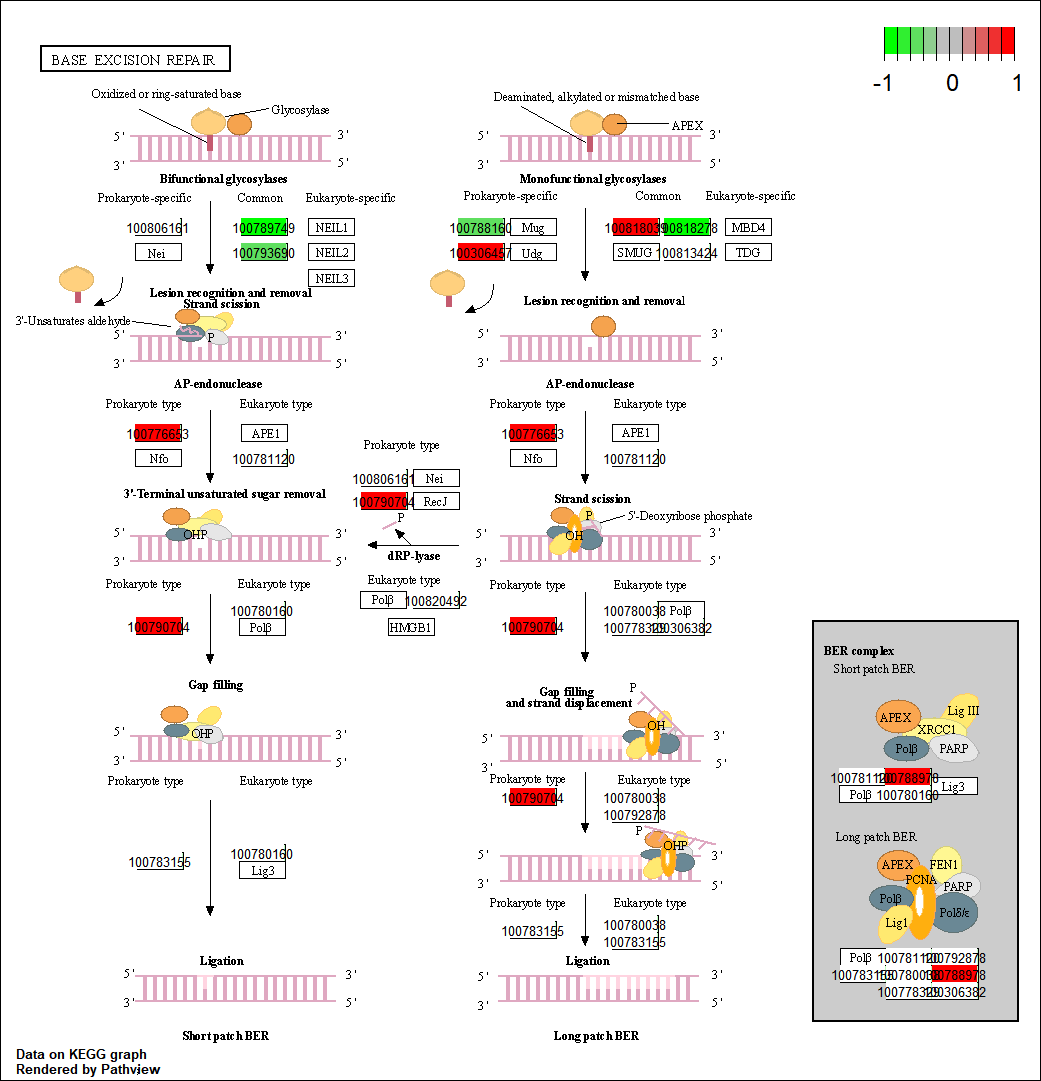

Supplement: Supplementary file 6 — Dataset S6 KEGG pathway maps for all pairwise comparisons; folder names correspond to specific treatment contrasts listed in heading of Dataset S6 in the main .docx document. [file NPH-250-2599-s003.zip › a.uninfected - Bj vs control, uninfected/up/gmx03410.pathview.png]

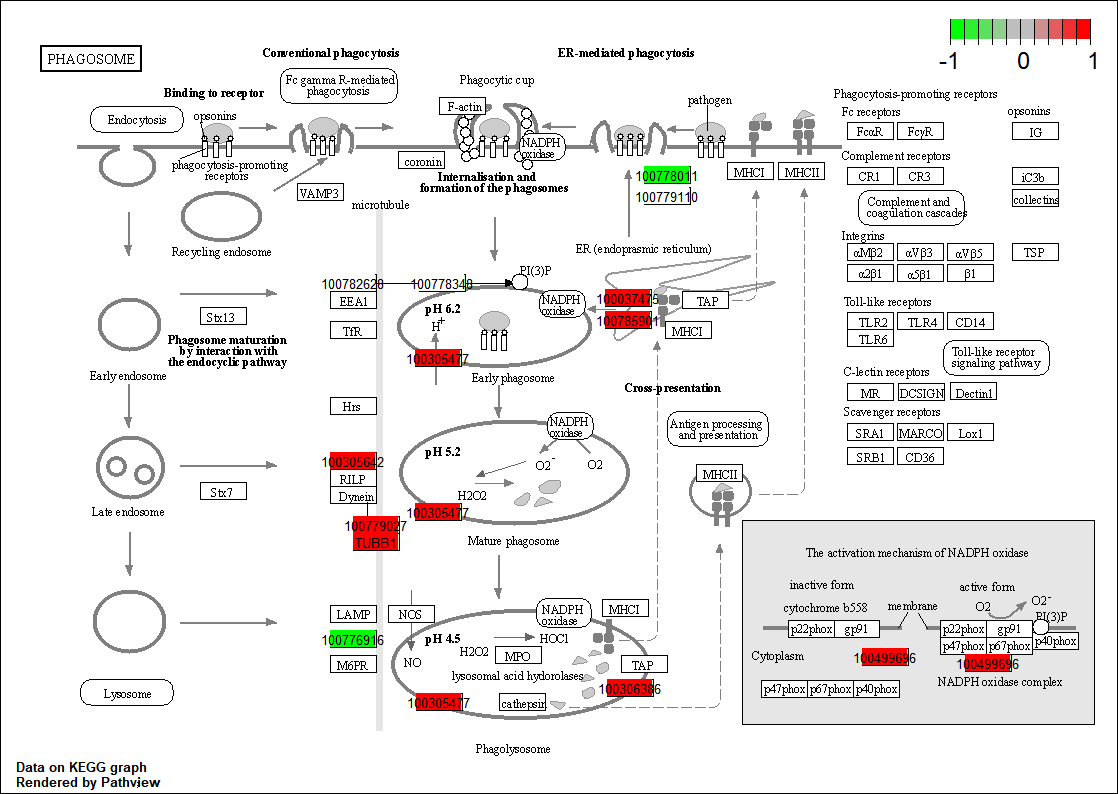

Supplement: Supplementary file 6 — Dataset S6 KEGG pathway maps for all pairwise comparisons; folder names correspond to specific treatment contrasts listed in heading of Dataset S6 in the main .docx document. [file NPH-250-2599-s003.zip › a.uninfected - Bj vs control, uninfected/up/gmx04145.pathview.png]

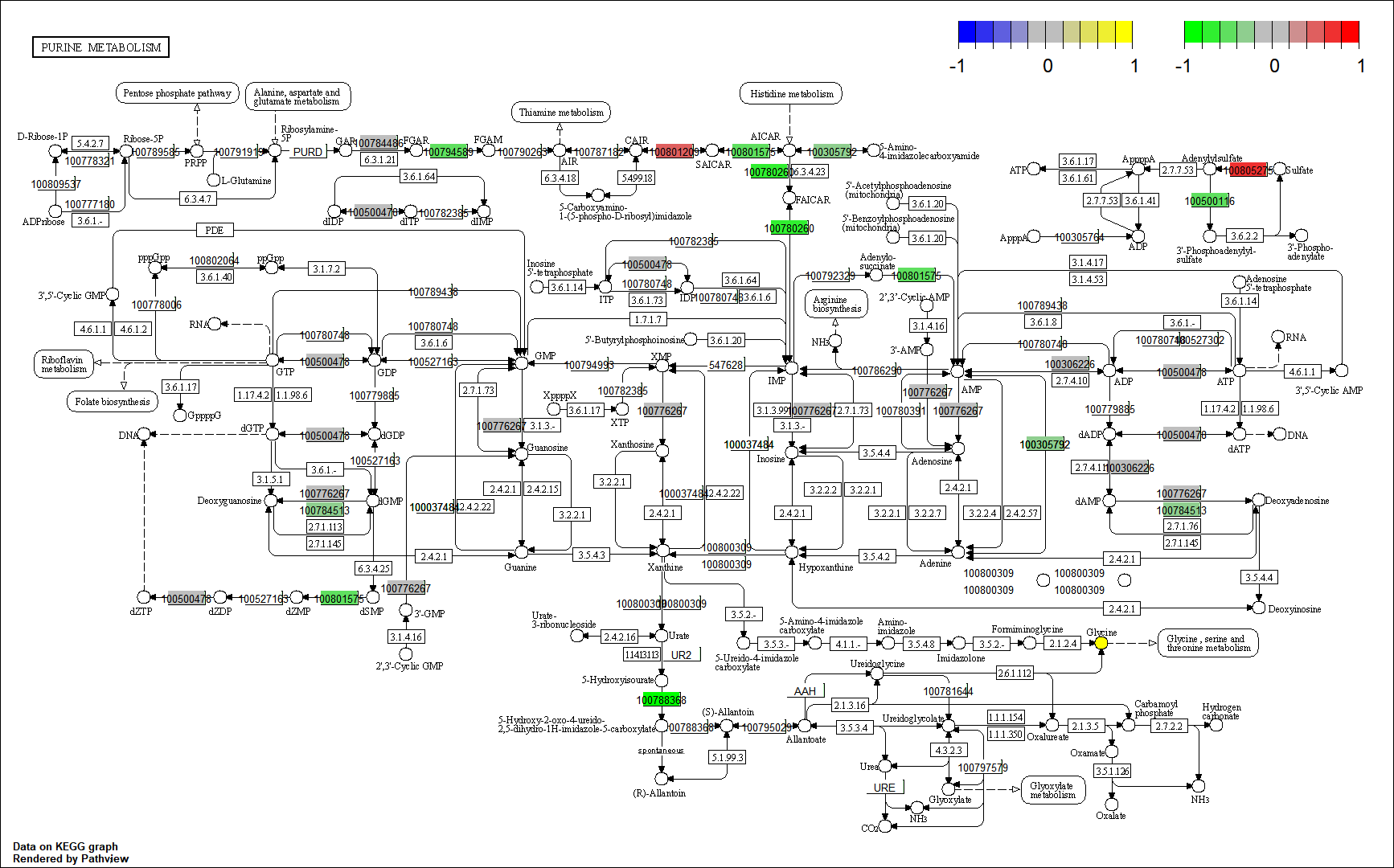

Supplement: Supplementary file 6 — Dataset S6 KEGG pathway maps for all pairwise comparisons; folder names correspond to specific treatment contrasts listed in heading of Dataset S6 in the main .docx document. [file NPH-250-2599-s003.zip › a.mixed - control BPMV vs control uninfected/down/gmx00230.pathview.png]

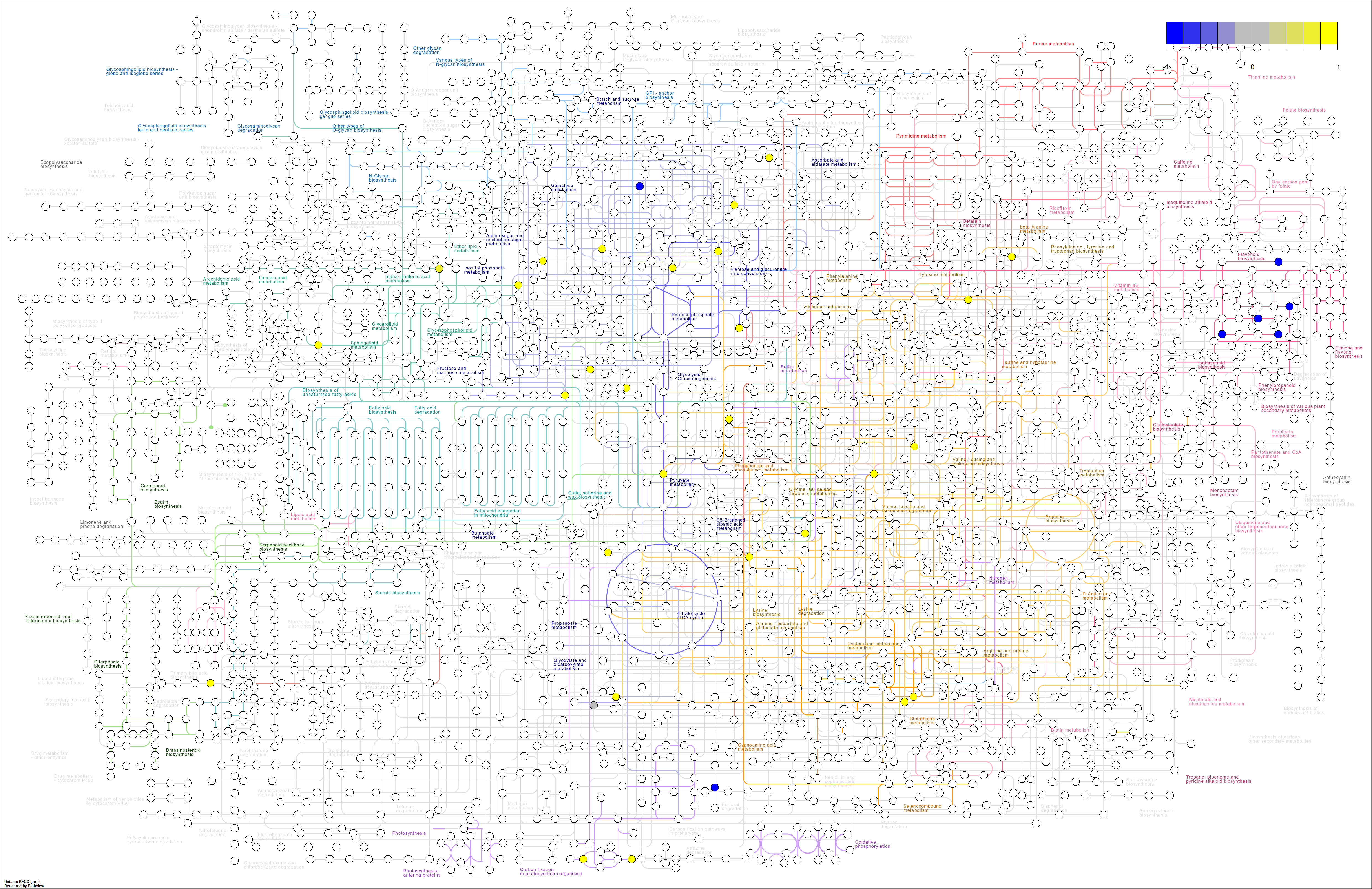

Supplement: Supplementary file 6 — Dataset S6 KEGG pathway maps for all pairwise comparisons; folder names correspond to specific treatment contrasts listed in heading of Dataset S6 in the main .docx document. [file NPH-250-2599-s003.zip › a.mixed - control BPMV vs control uninfected/down/gmx01100.pathview.png]

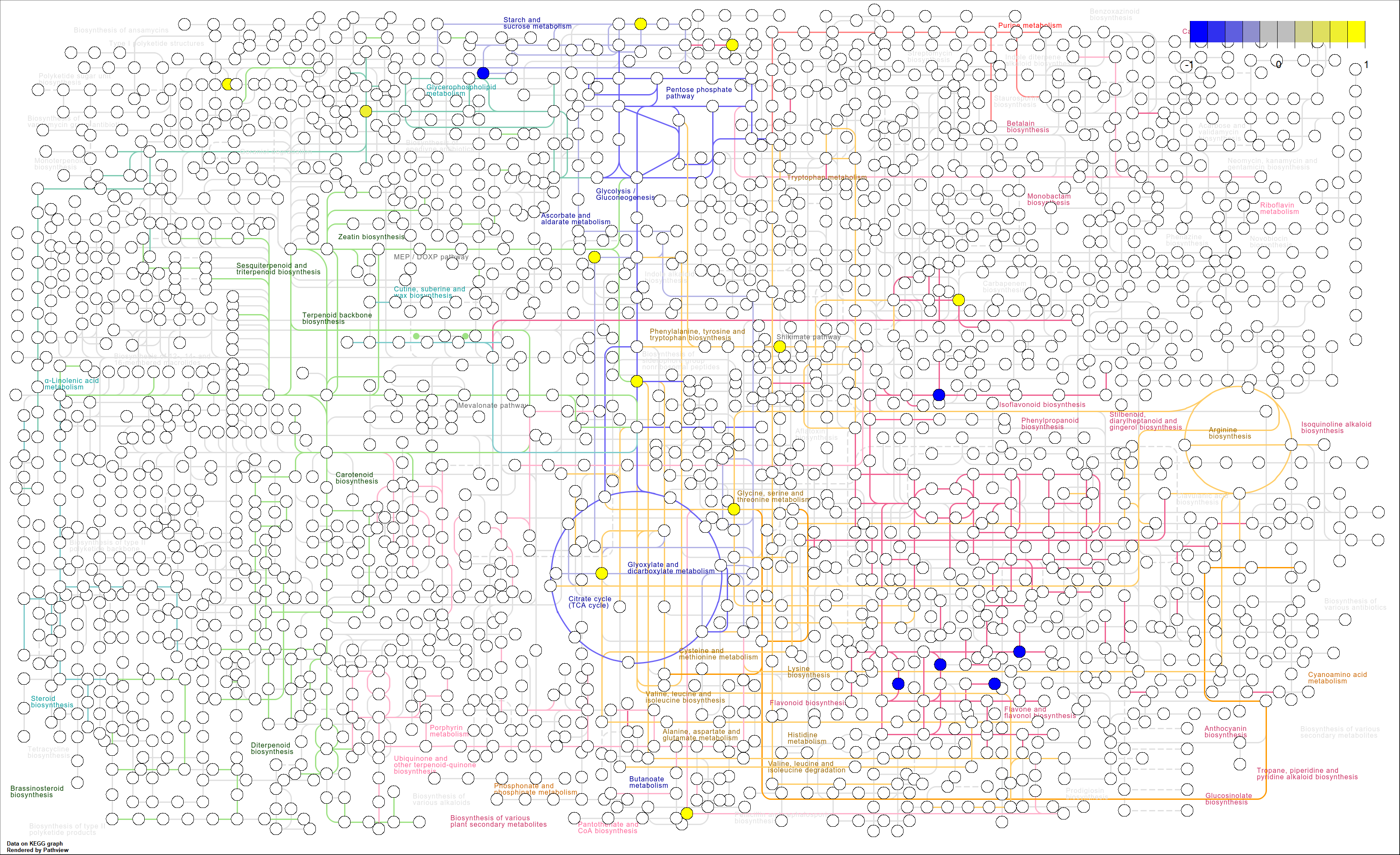

Supplement: Supplementary file 6 — Dataset S6 KEGG pathway maps for all pairwise comparisons; folder names correspond to specific treatment contrasts listed in heading of Dataset S6 in the main .docx document. [file NPH-250-2599-s003.zip › a.mixed - control BPMV vs control uninfected/down/gmx01110.pathview.png]

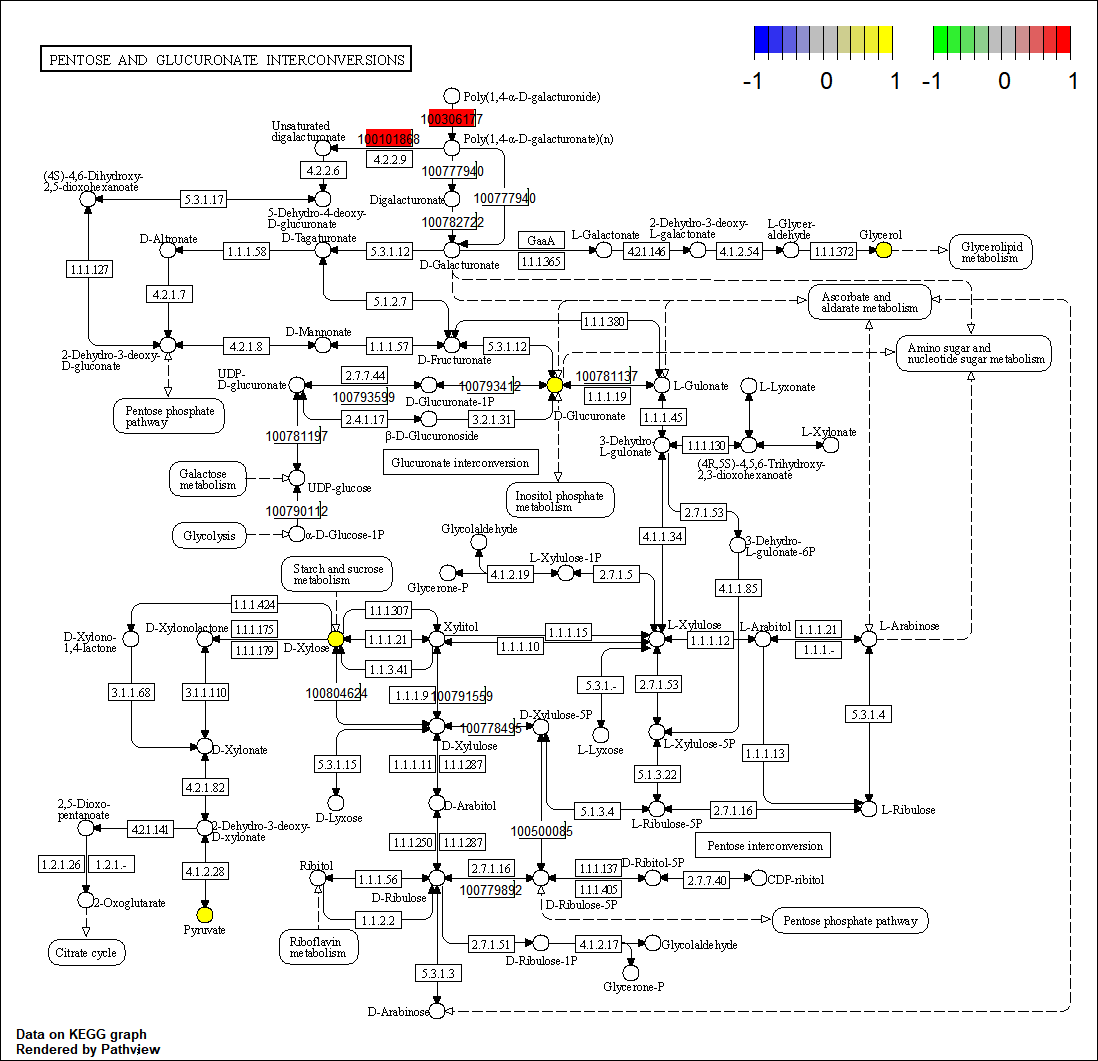

Supplement: Supplementary file 6 — Dataset S6 KEGG pathway maps for all pairwise comparisons; folder names correspond to specific treatment contrasts listed in heading of Dataset S6 in the main .docx document. [file NPH-250-2599-s003.zip › a.mixed - control BPMV vs control uninfected/up/gmx00040.pathview.png]

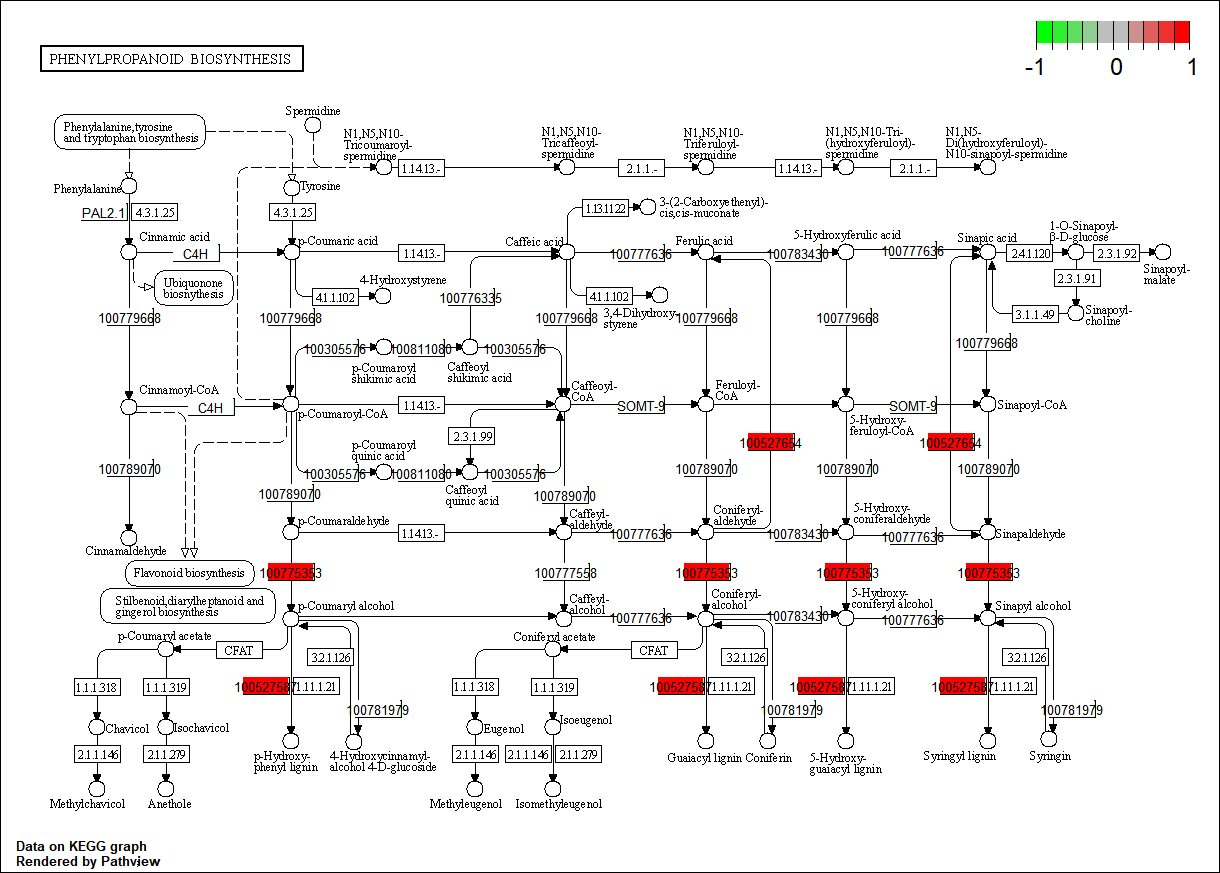

Supplement: Supplementary file 6 — Dataset S6 KEGG pathway maps for all pairwise comparisons; folder names correspond to specific treatment contrasts listed in heading of Dataset S6 in the main .docx document. [file NPH-250-2599-s003.zip › a.mixed - control BPMV vs control uninfected/up/gmx00940.pathview.png]

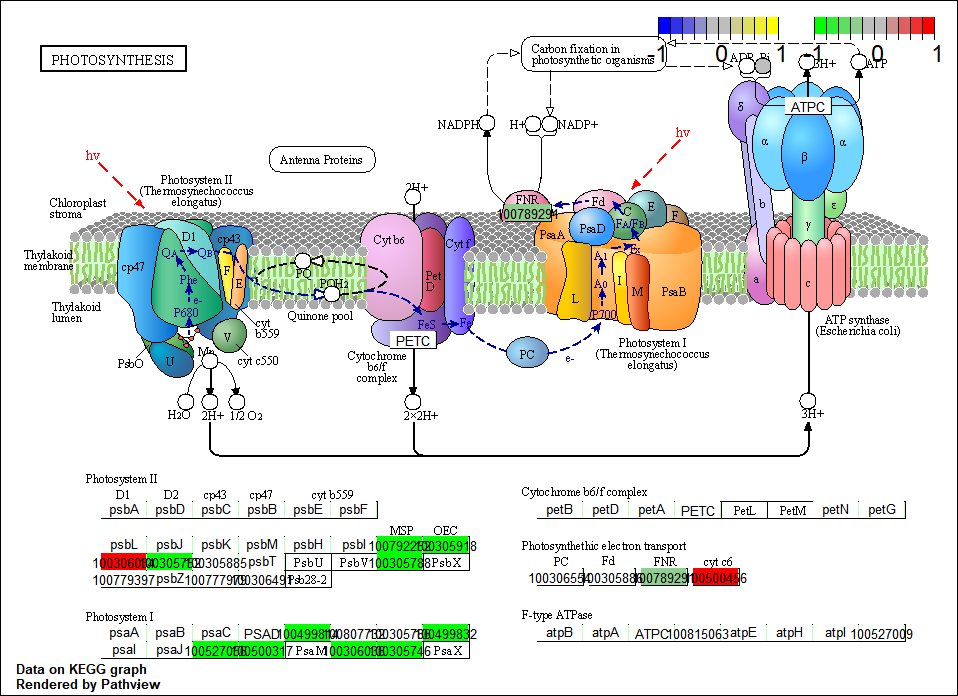

Supplement: Supplementary file 6 — Dataset S6 KEGG pathway maps for all pairwise comparisons; folder names correspond to specific treatment contrasts listed in heading of Dataset S6 in the main .docx document. [file NPH-250-2599-s003.zip › b.BPMV - Bj+Da vs control, BPMV/down/gmx00195.pathview.png]

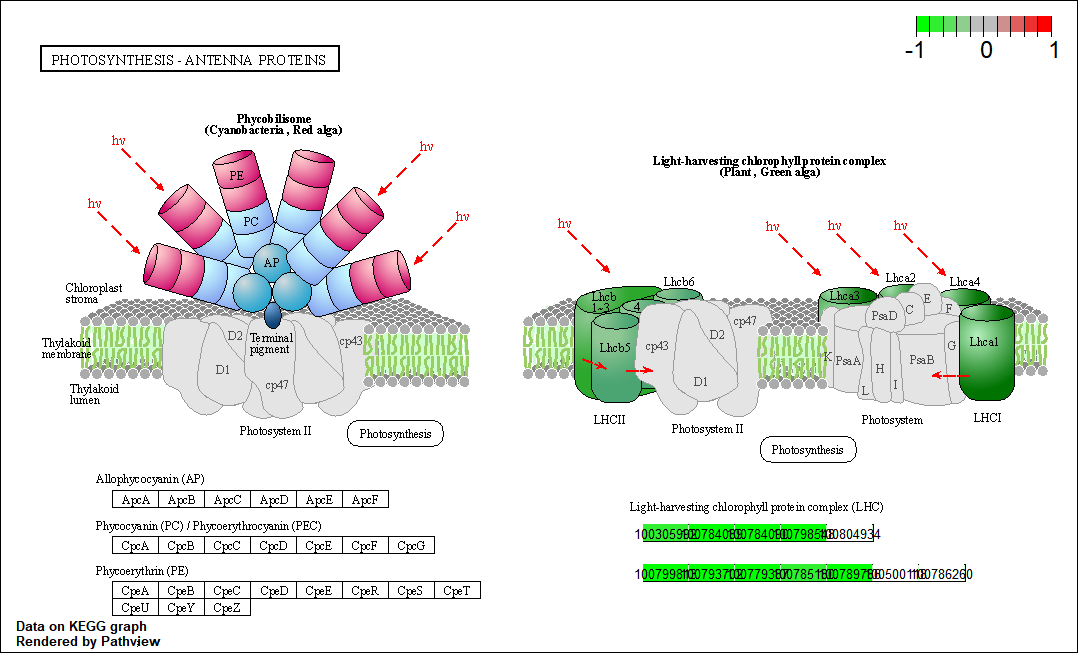

Supplement: Supplementary file 6 — Dataset S6 KEGG pathway maps for all pairwise comparisons; folder names correspond to specific treatment contrasts listed in heading of Dataset S6 in the main .docx document. [file NPH-250-2599-s003.zip › b.BPMV - Bj+Da vs control, BPMV/down/gmx00196.pathview.png]

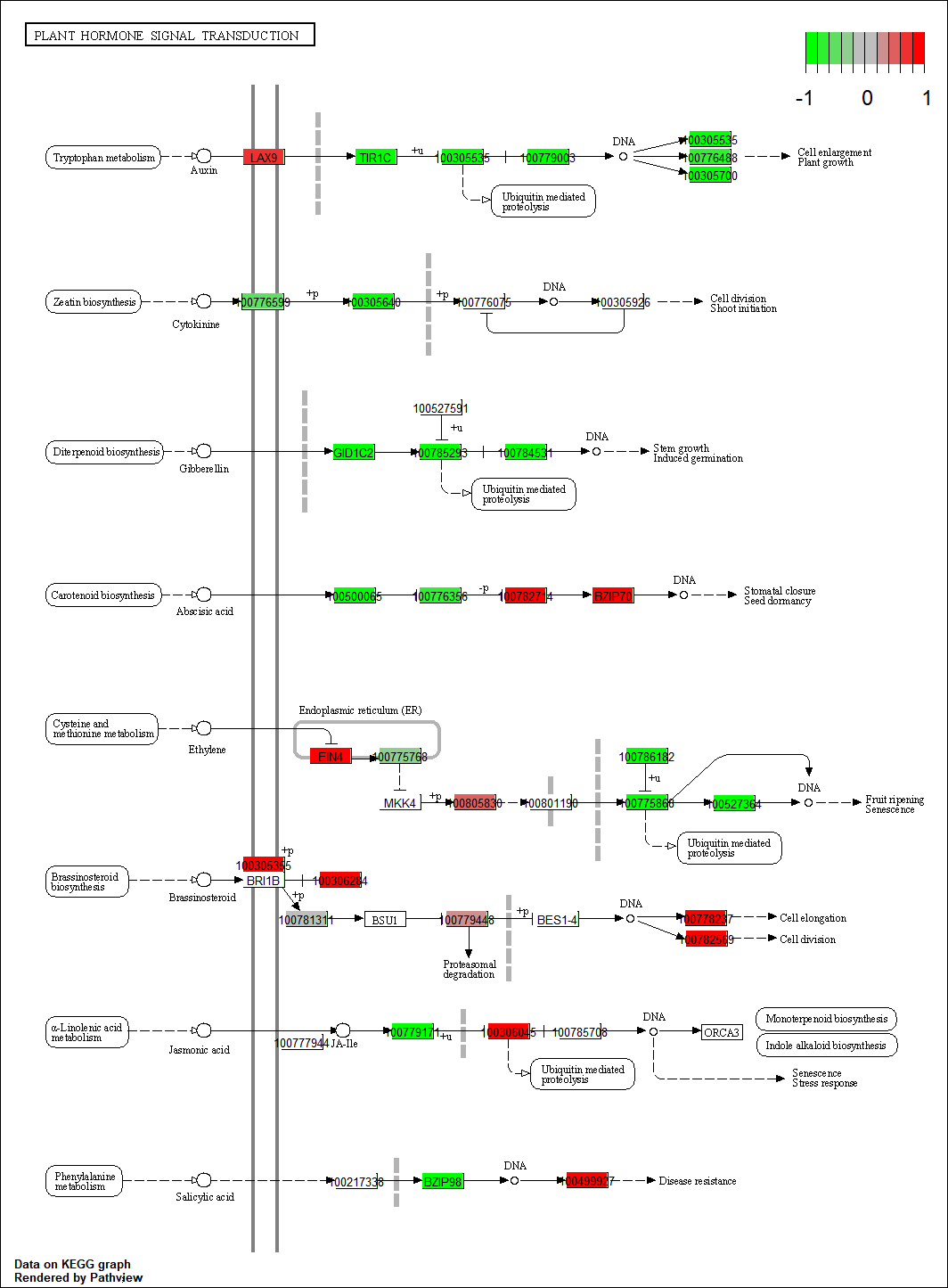

Supplement: Supplementary file 6 — Dataset S6 KEGG pathway maps for all pairwise comparisons; folder names correspond to specific treatment contrasts listed in heading of Dataset S6 in the main .docx document. [file NPH-250-2599-s003.zip › b.BPMV - Bj+Da vs control, BPMV/down/gmx04075.pathview.png]

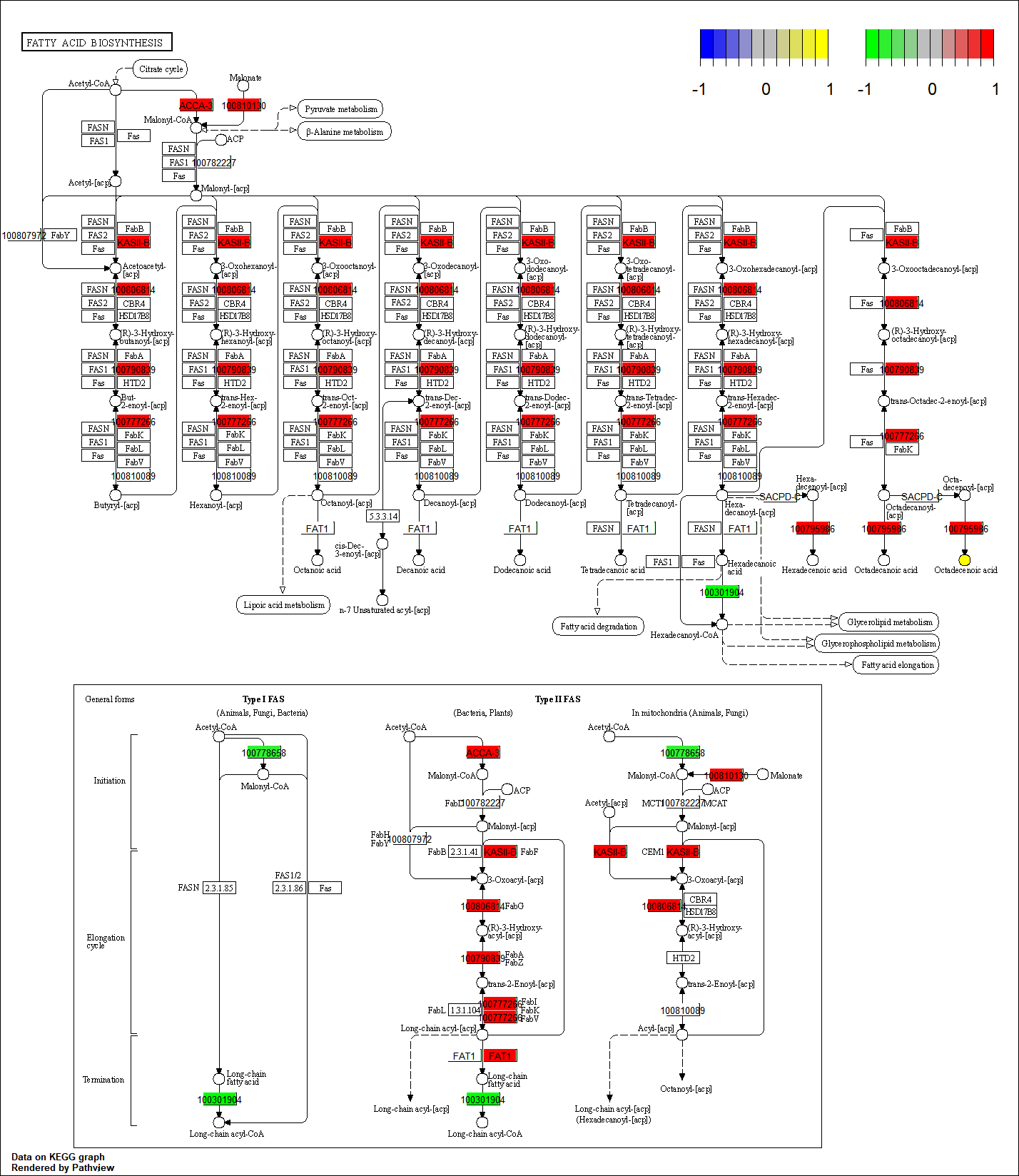

Supplement: Supplementary file 6 — Dataset S6 KEGG pathway maps for all pairwise comparisons; folder names correspond to specific treatment contrasts listed in heading of Dataset S6 in the main .docx document. [file NPH-250-2599-s003.zip › b.BPMV - Bj+Da vs control, BPMV/up/gmx00061.pathview.png]

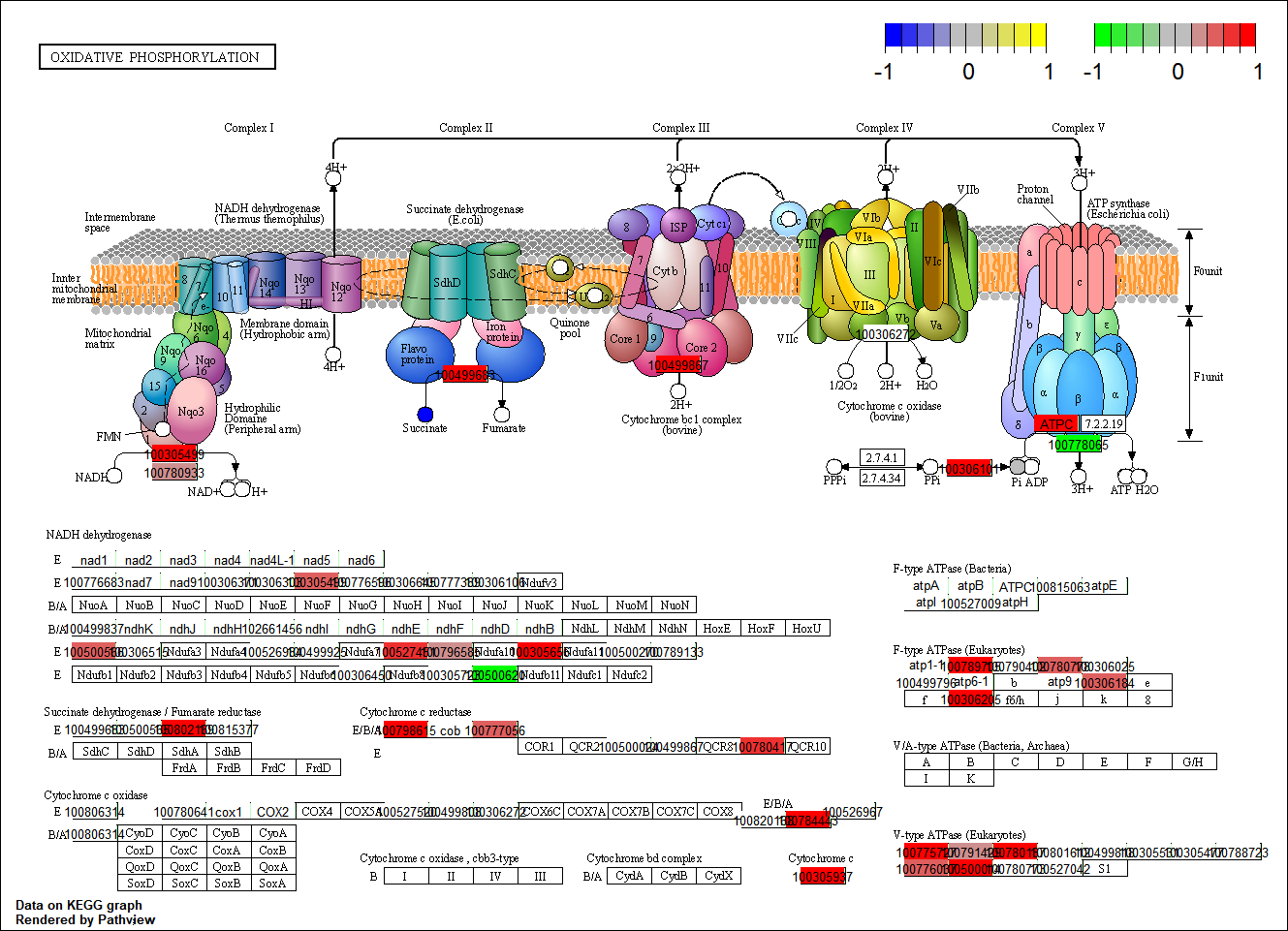

Supplement: Supplementary file 6 — Dataset S6 KEGG pathway maps for all pairwise comparisons; folder names correspond to specific treatment contrasts listed in heading of Dataset S6 in the main .docx document. [file NPH-250-2599-s003.zip › b.BPMV - Bj+Da vs control, BPMV/up/gmx00190.pathview.png]

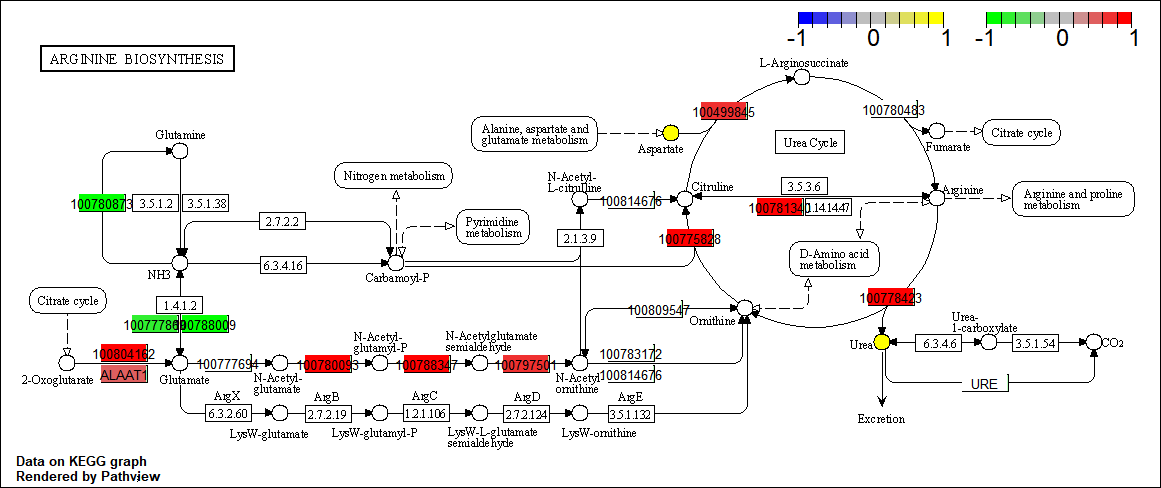

Supplement: Supplementary file 6 — Dataset S6 KEGG pathway maps for all pairwise comparisons; folder names correspond to specific treatment contrasts listed in heading of Dataset S6 in the main .docx document. [file NPH-250-2599-s003.zip › b.BPMV - Bj+Da vs control, BPMV/up/gmx00220.pathview.png]

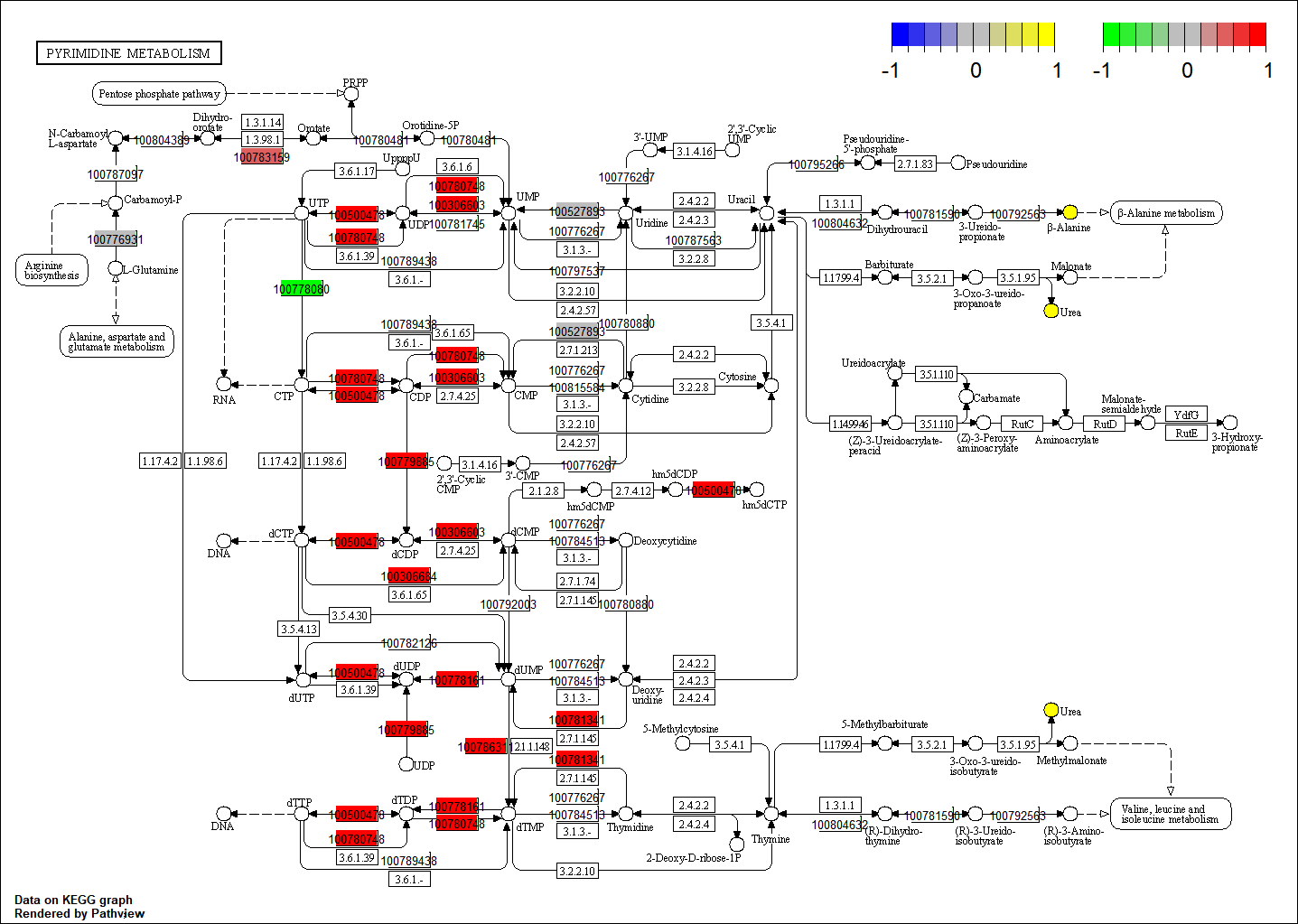

Supplement: Supplementary file 6 — Dataset S6 KEGG pathway maps for all pairwise comparisons; folder names correspond to specific treatment contrasts listed in heading of Dataset S6 in the main .docx document. [file NPH-250-2599-s003.zip › b.BPMV - Bj+Da vs control, BPMV/up/gmx00240.pathview.png]

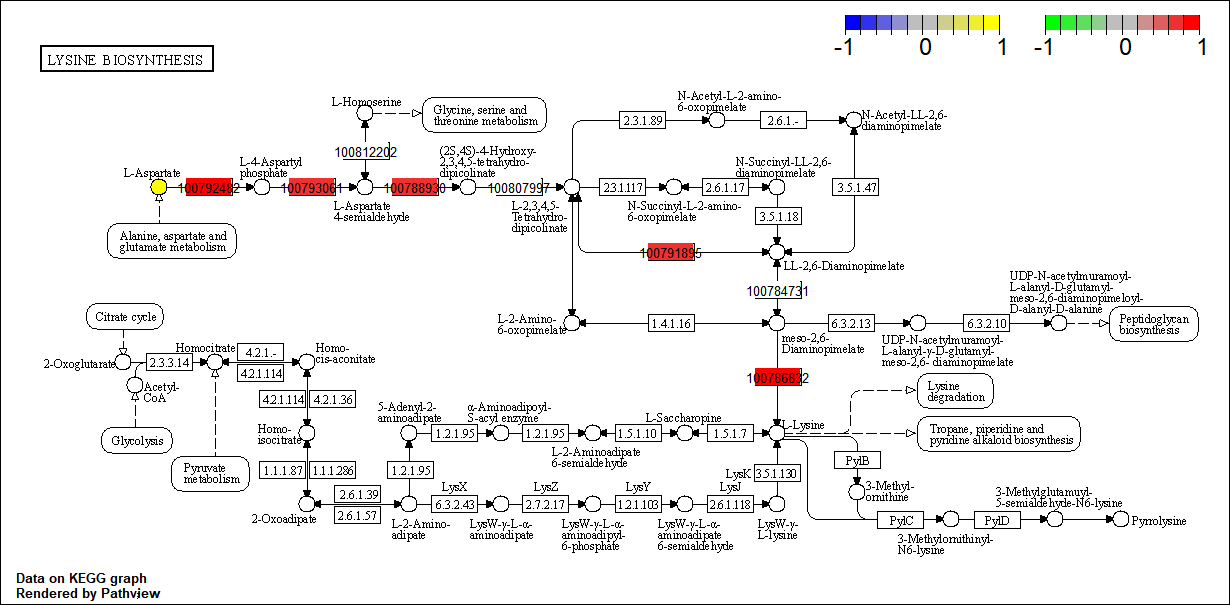

Supplement: Supplementary file 6 — Dataset S6 KEGG pathway maps for all pairwise comparisons; folder names correspond to specific treatment contrasts listed in heading of Dataset S6 in the main .docx document. [file NPH-250-2599-s003.zip › b.BPMV - Bj+Da vs control, BPMV/up/gmx00300.pathview.png]

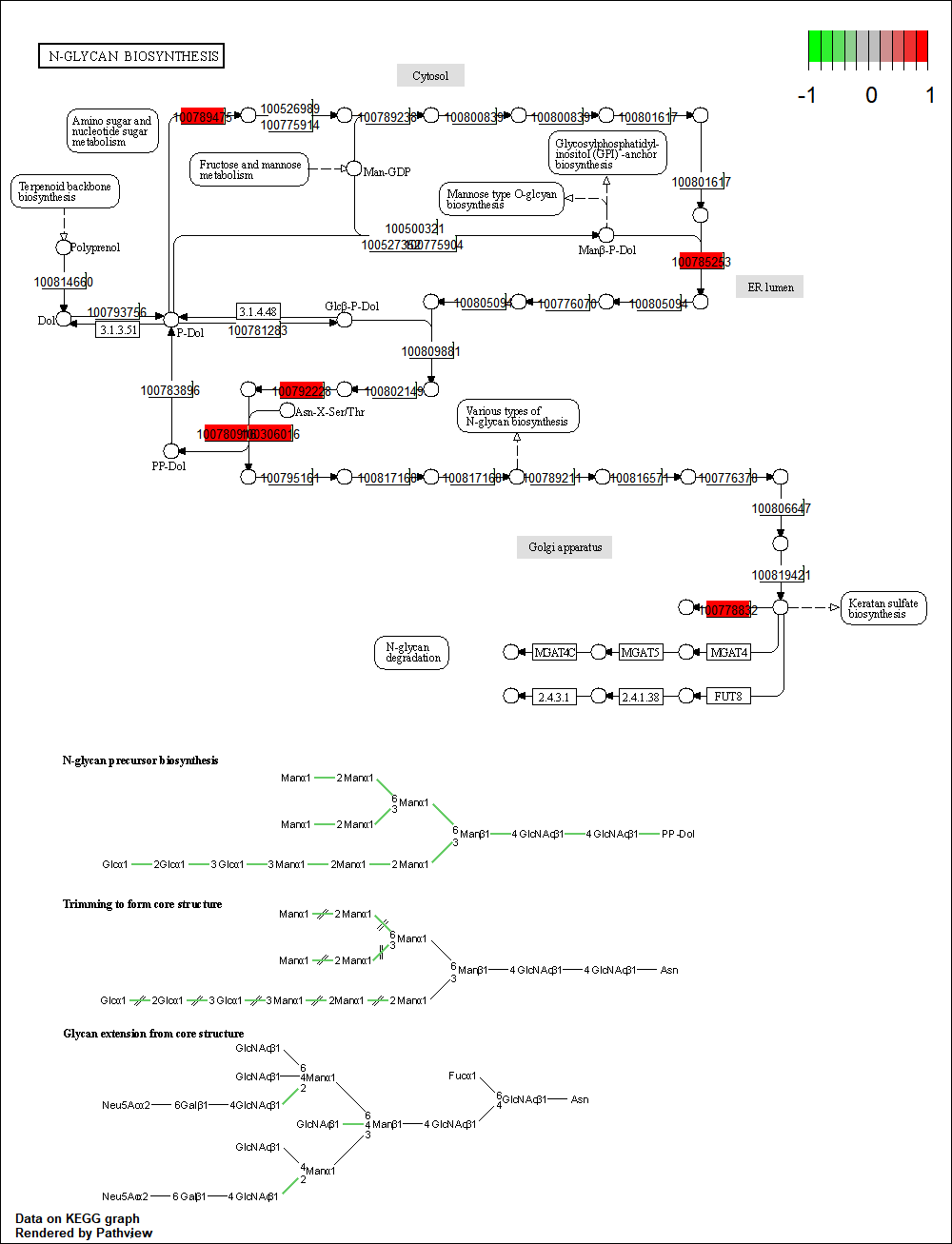

Supplement: Supplementary file 6 — Dataset S6 KEGG pathway maps for all pairwise comparisons; folder names correspond to specific treatment contrasts listed in heading of Dataset S6 in the main .docx document. [file NPH-250-2599-s003.zip › b.BPMV - Bj+Da vs control, BPMV/up/gmx00510.pathview.png]

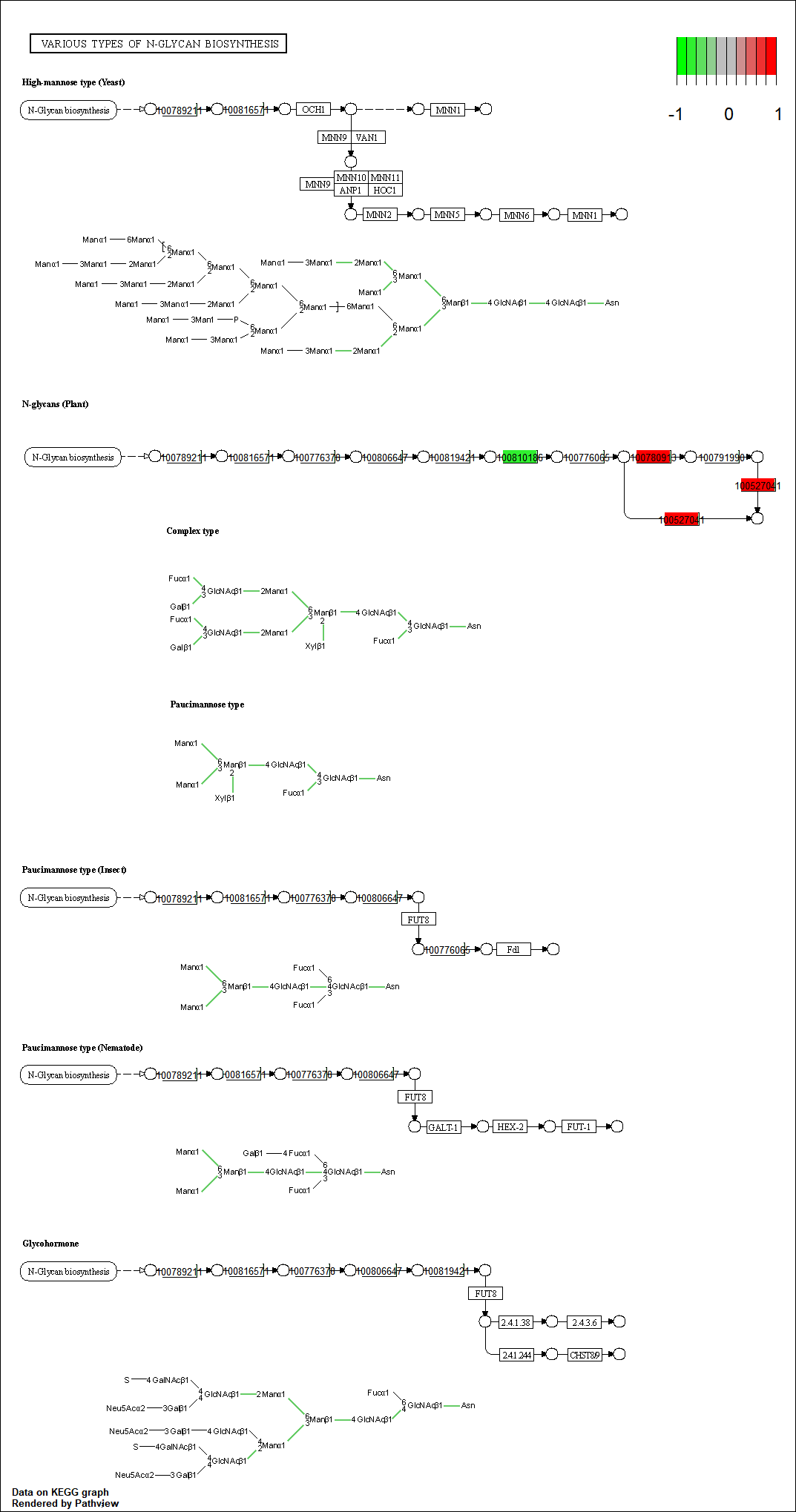

Supplement: Supplementary file 6 — Dataset S6 KEGG pathway maps for all pairwise comparisons; folder names correspond to specific treatment contrasts listed in heading of Dataset S6 in the main .docx document. [file NPH-250-2599-s003.zip › b.BPMV - Bj+Da vs control, BPMV/up/gmx00513.pathview.png]

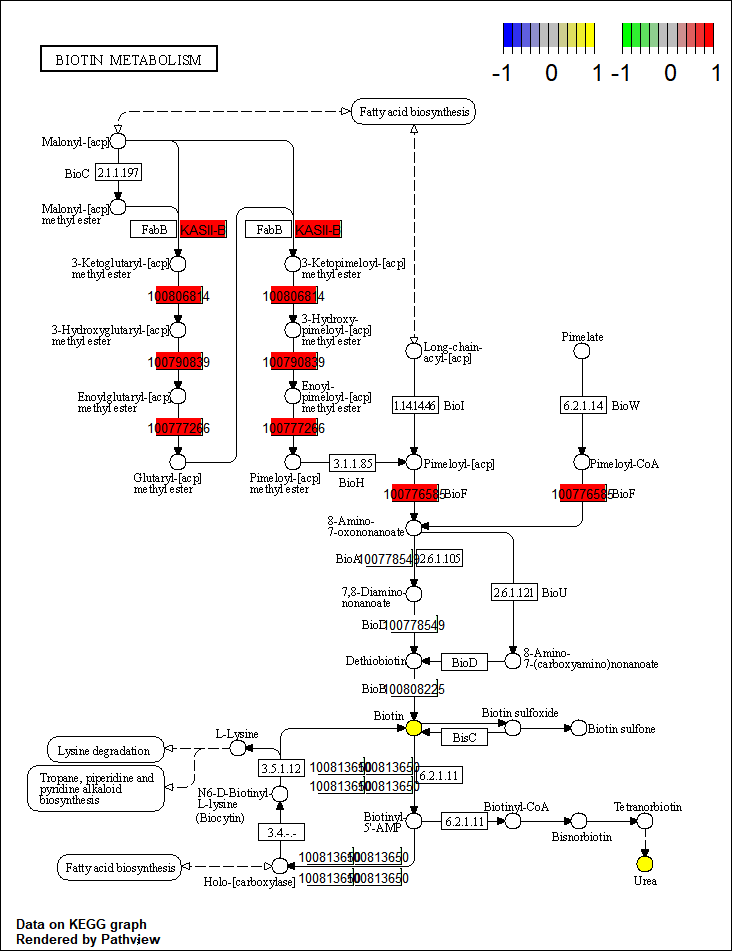

Supplement: Supplementary file 6 — Dataset S6 KEGG pathway maps for all pairwise comparisons; folder names correspond to specific treatment contrasts listed in heading of Dataset S6 in the main .docx document. [file NPH-250-2599-s003.zip › b.BPMV - Bj+Da vs control, BPMV/up/gmx00780.pathview.png]

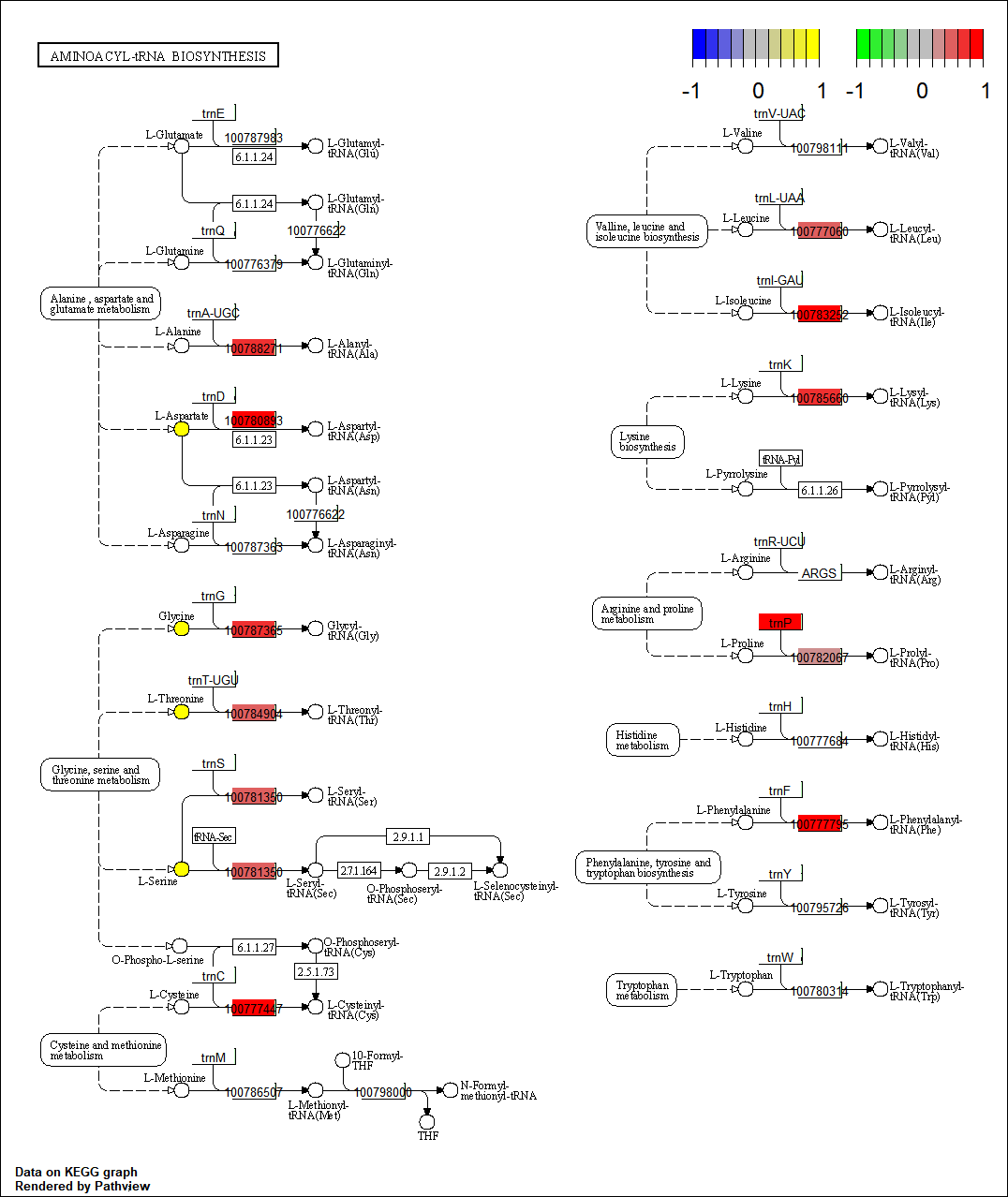

Supplement: Supplementary file 6 — Dataset S6 KEGG pathway maps for all pairwise comparisons; folder names correspond to specific treatment contrasts listed in heading of Dataset S6 in the main .docx document. [file NPH-250-2599-s003.zip › b.BPMV - Bj+Da vs control, BPMV/up/gmx00970.pathview.png]

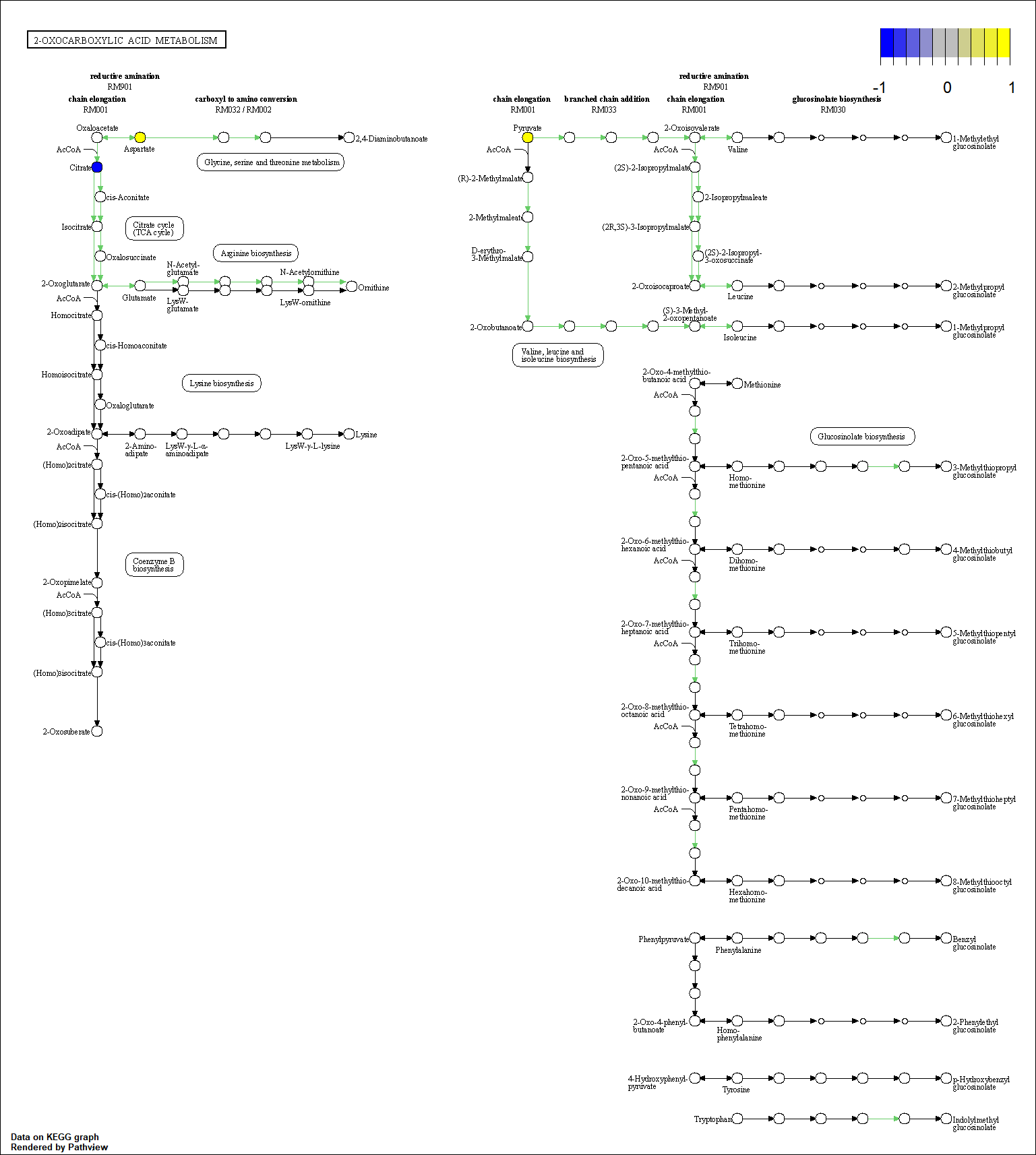

Supplement: Supplementary file 6 — Dataset S6 KEGG pathway maps for all pairwise comparisons; folder names correspond to specific treatment contrasts listed in heading of Dataset S6 in the main .docx document. [file NPH-250-2599-s003.zip › b.BPMV - Bj+Da vs control, BPMV/up/gmx01210.pathview.png]

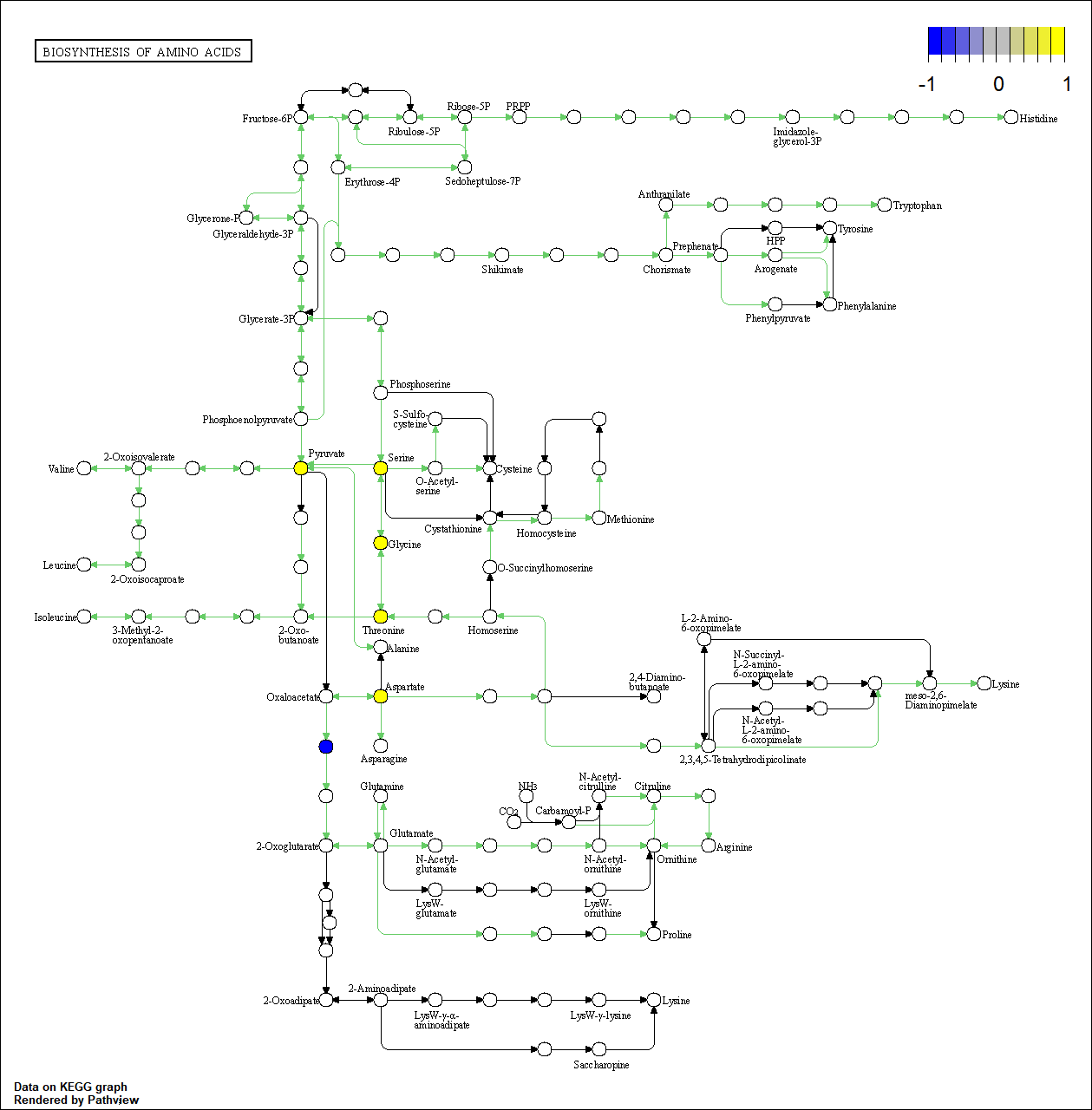

Supplement: Supplementary file 6 — Dataset S6 KEGG pathway maps for all pairwise comparisons; folder names correspond to specific treatment contrasts listed in heading of Dataset S6 in the main .docx document. [file NPH-250-2599-s003.zip › b.BPMV - Bj+Da vs control, BPMV/up/gmx01230.pathview.png]

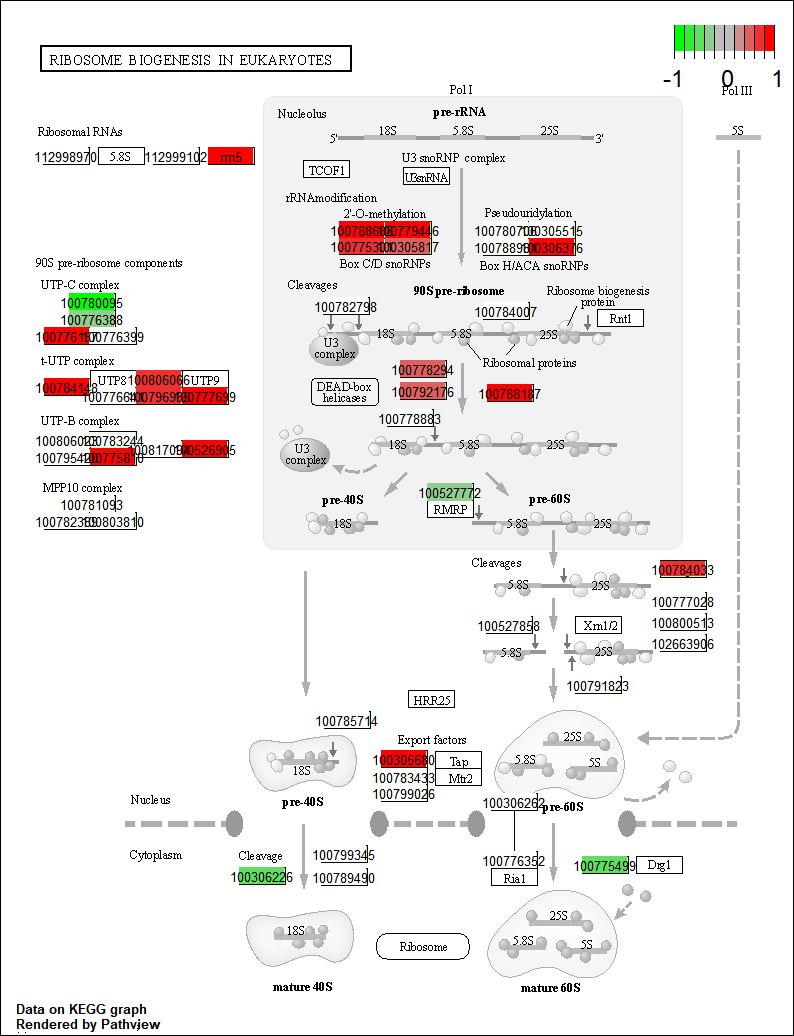

Supplement: Supplementary file 6 — Dataset S6 KEGG pathway maps for all pairwise comparisons; folder names correspond to specific treatment contrasts listed in heading of Dataset S6 in the main .docx document. [file NPH-250-2599-s003.zip › b.BPMV - Bj+Da vs control, BPMV/up/gmx03008.pathview.png]

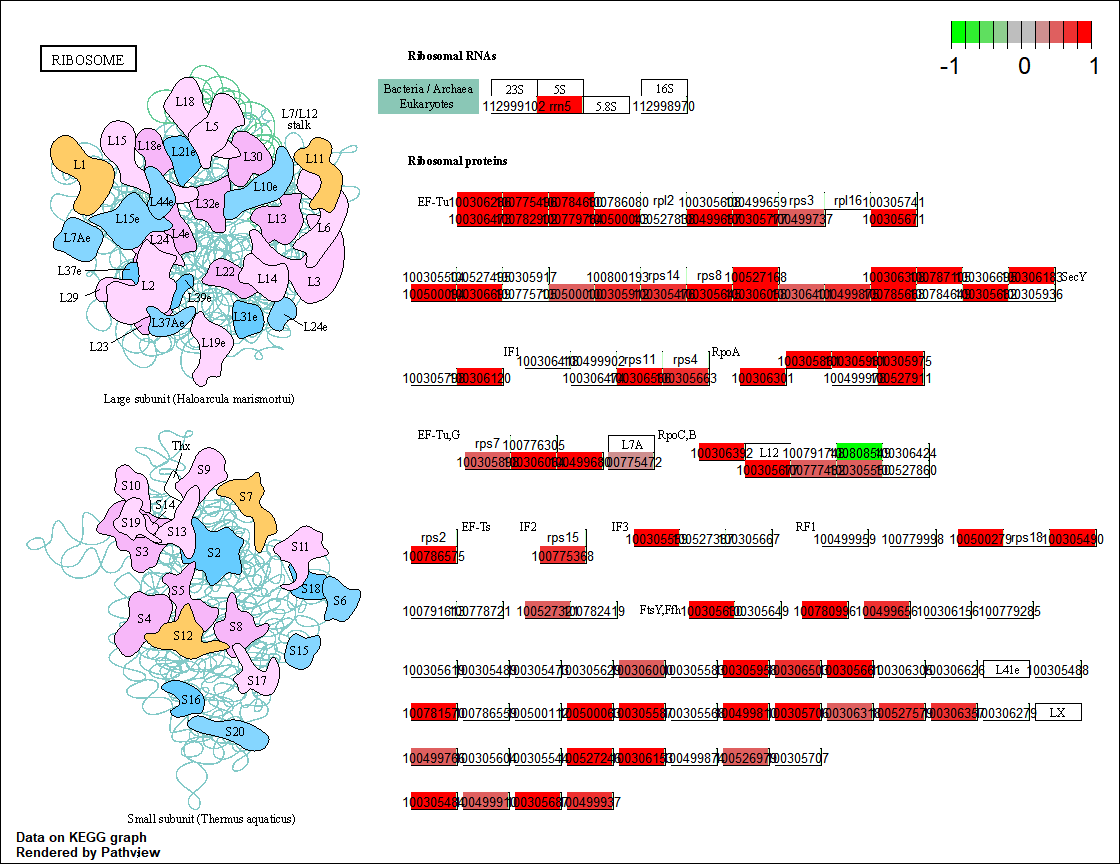

Supplement: Supplementary file 6 — Dataset S6 KEGG pathway maps for all pairwise comparisons; folder names correspond to specific treatment contrasts listed in heading of Dataset S6 in the main .docx document. [file NPH-250-2599-s003.zip › b.BPMV - Bj+Da vs control, BPMV/up/gmx03010.pathview.png]

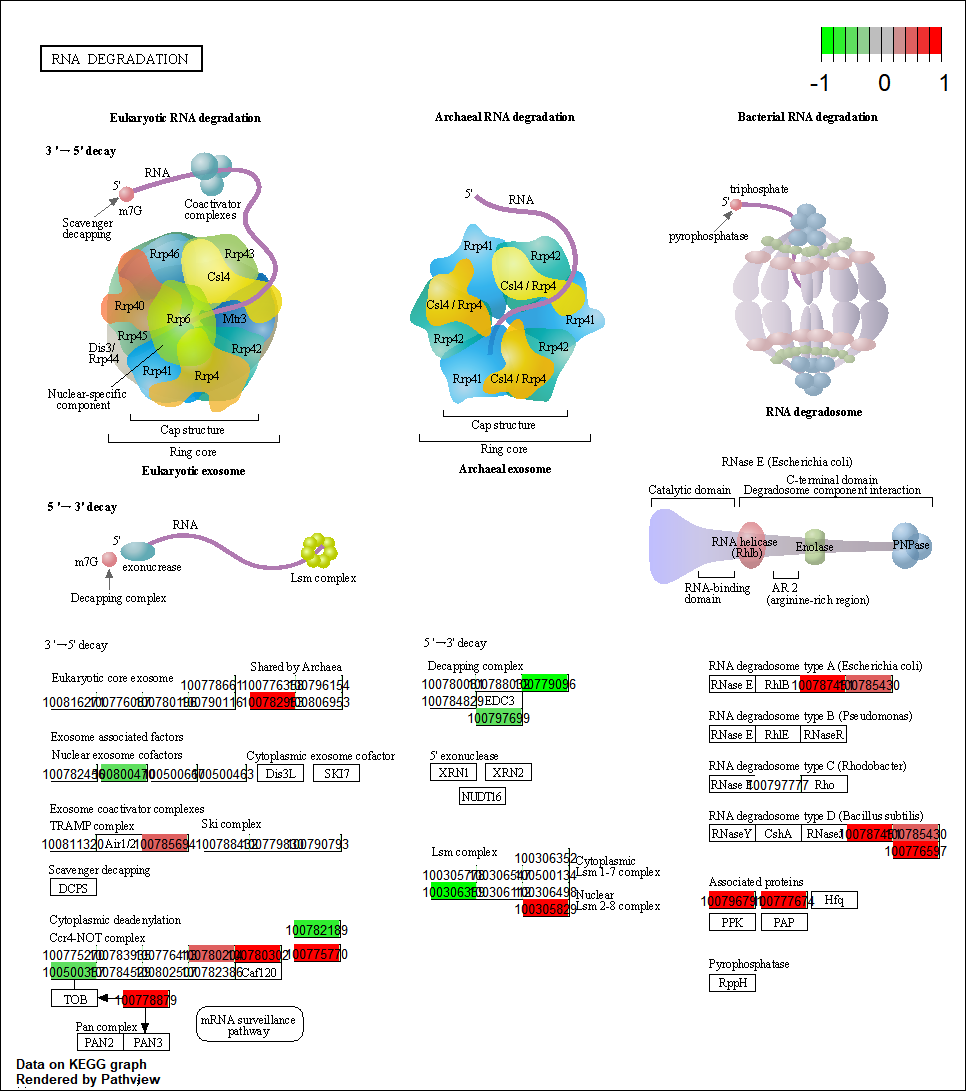

Supplement: Supplementary file 6 — Dataset S6 KEGG pathway maps for all pairwise comparisons; folder names correspond to specific treatment contrasts listed in heading of Dataset S6 in the main .docx document. [file NPH-250-2599-s003.zip › b.BPMV - Bj+Da vs control, BPMV/up/gmx03018.pathview.png]

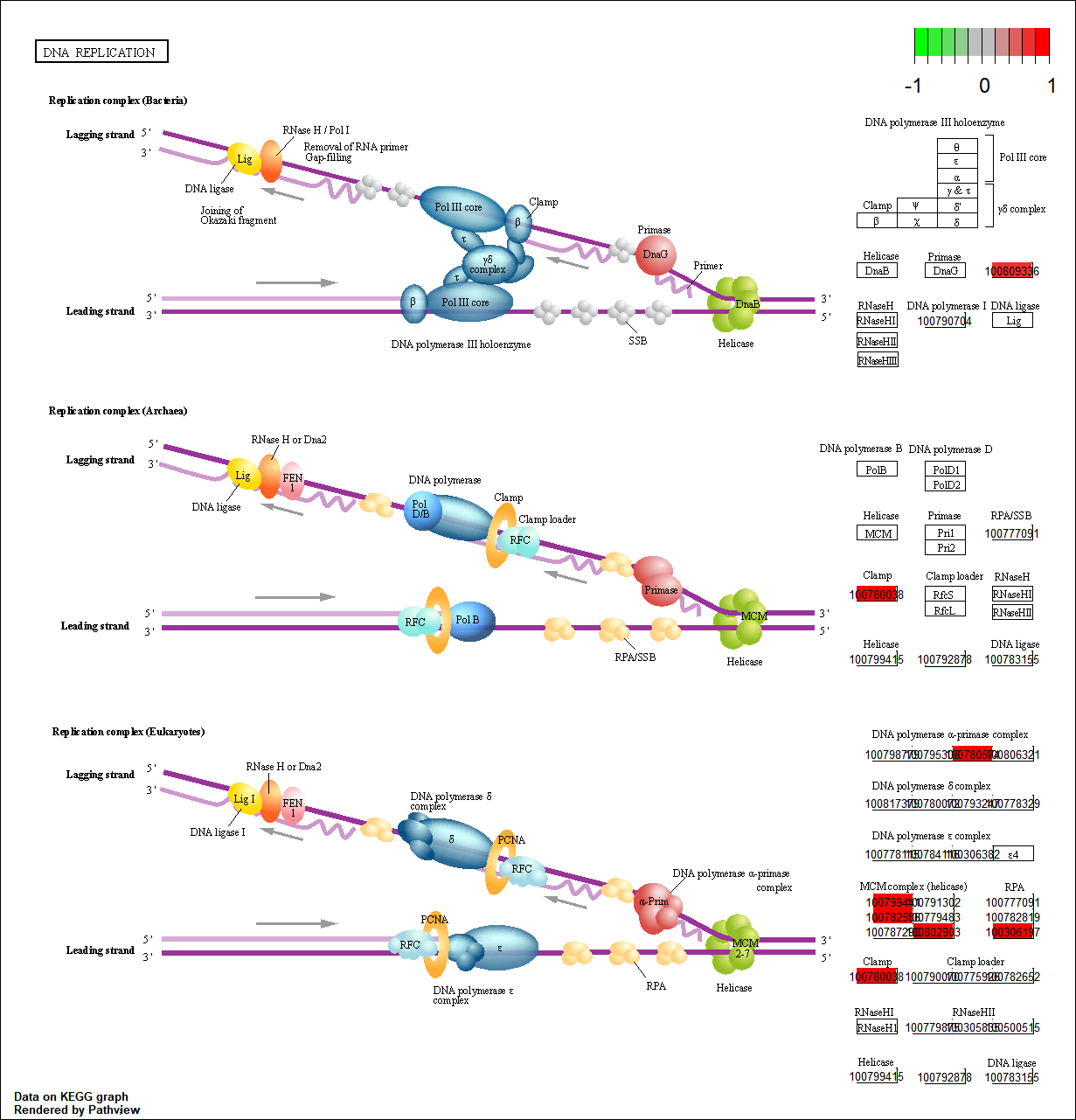

Supplement: Supplementary file 6 — Dataset S6 KEGG pathway maps for all pairwise comparisons; folder names correspond to specific treatment contrasts listed in heading of Dataset S6 in the main .docx document. [file NPH-250-2599-s003.zip › b.BPMV - Bj+Da vs control, BPMV/up/gmx03030.pathview.png]

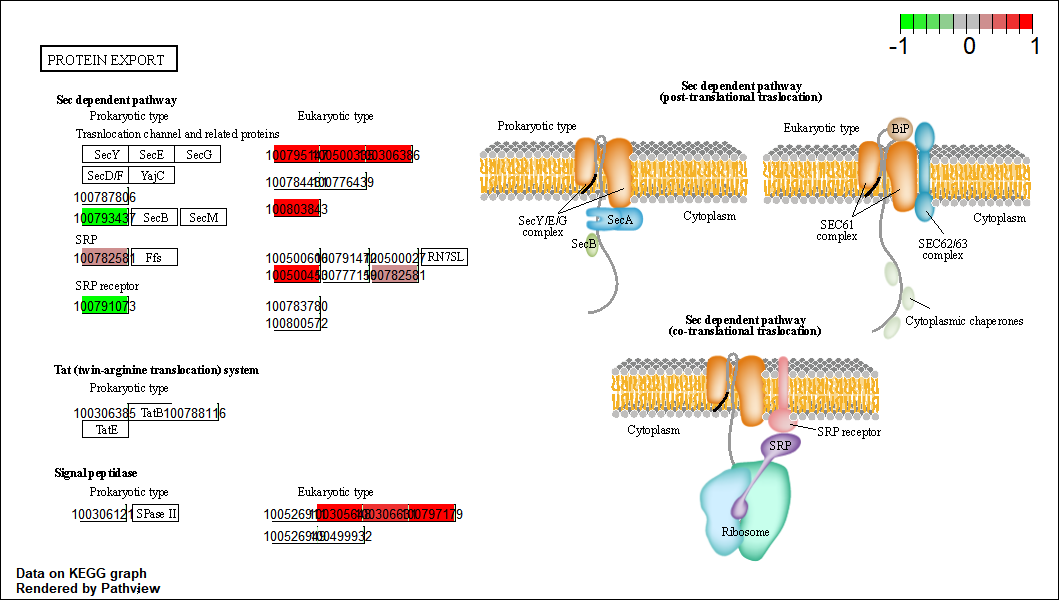

Supplement: Supplementary file 6 — Dataset S6 KEGG pathway maps for all pairwise comparisons; folder names correspond to specific treatment contrasts listed in heading of Dataset S6 in the main .docx document. [file NPH-250-2599-s003.zip › b.BPMV - Bj+Da vs control, BPMV/up/gmx03060.pathview.png]

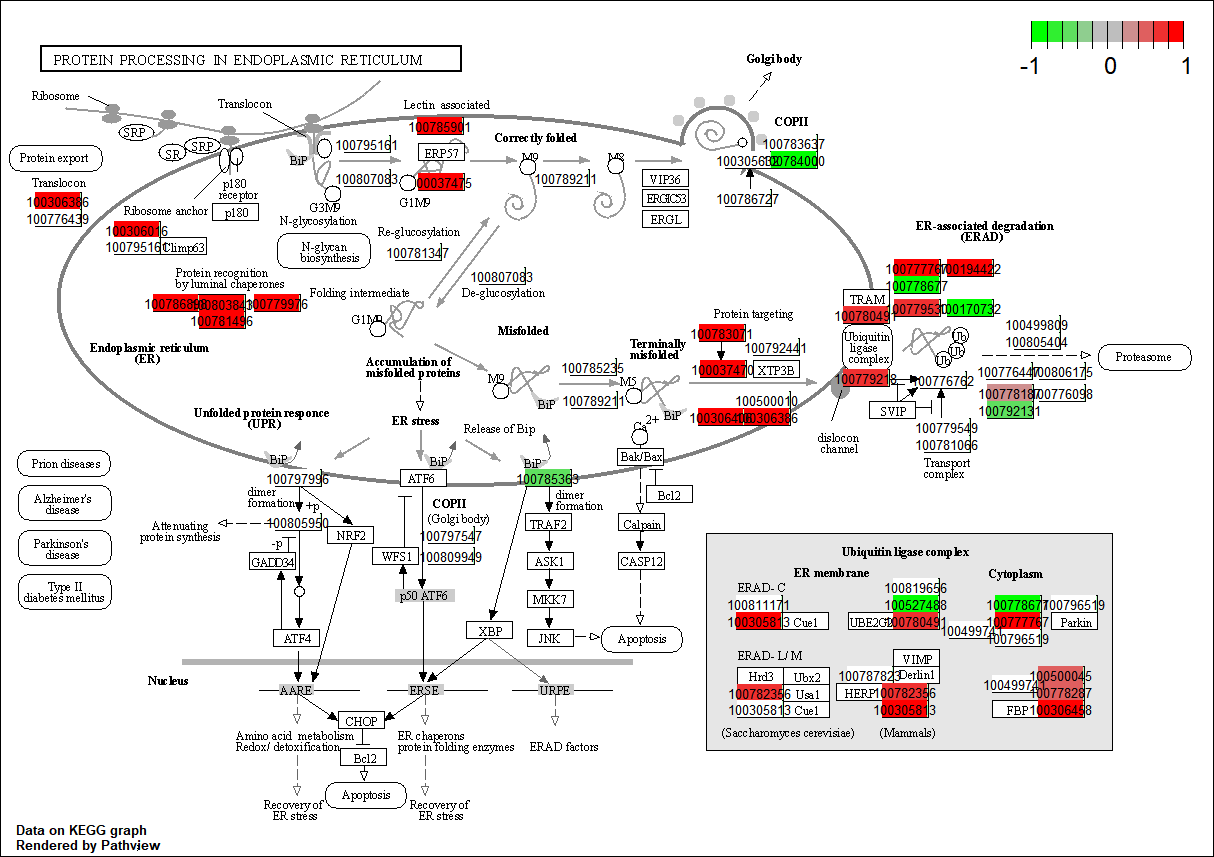

Supplement: Supplementary file 6 — Dataset S6 KEGG pathway maps for all pairwise comparisons; folder names correspond to specific treatment contrasts listed in heading of Dataset S6 in the main .docx document. [file NPH-250-2599-s003.zip › b.BPMV - Bj+Da vs control, BPMV/up/gmx04141.pathview.png]

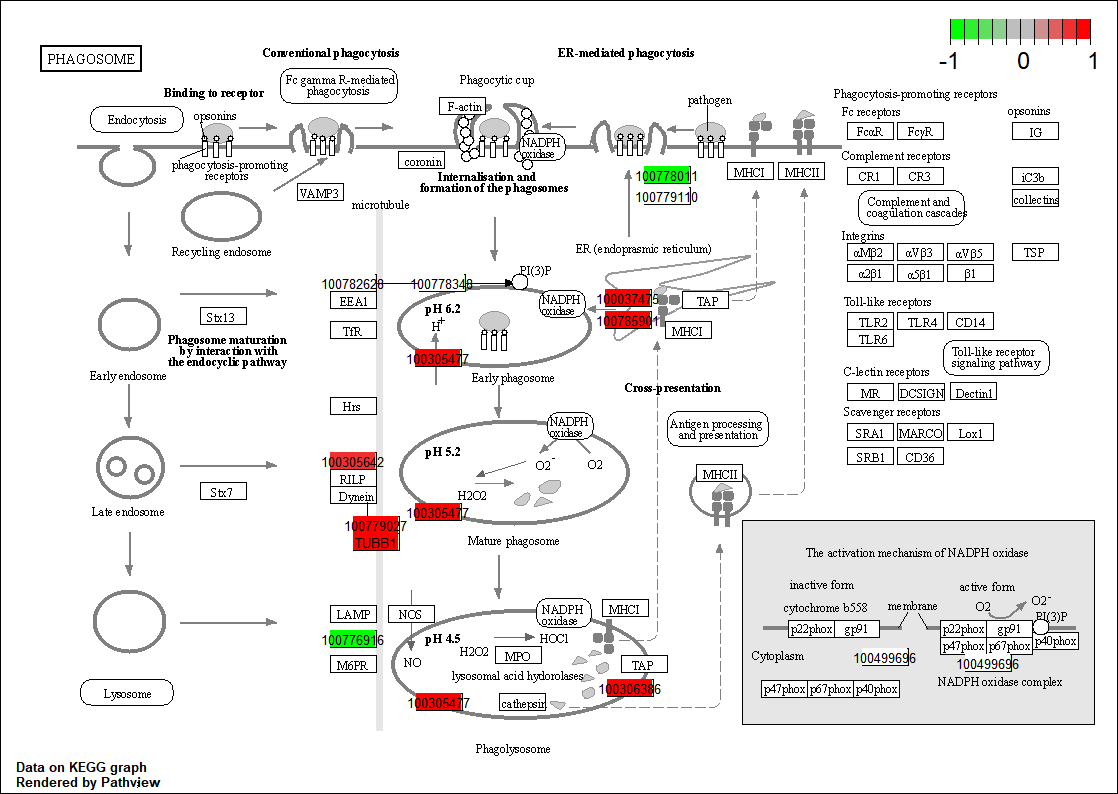

Supplement: Supplementary file 6 — Dataset S6 KEGG pathway maps for all pairwise comparisons; folder names correspond to specific treatment contrasts listed in heading of Dataset S6 in the main .docx document. [file NPH-250-2599-s003.zip › b.BPMV - Bj+Da vs control, BPMV/up/gmx04145.pathview.png]

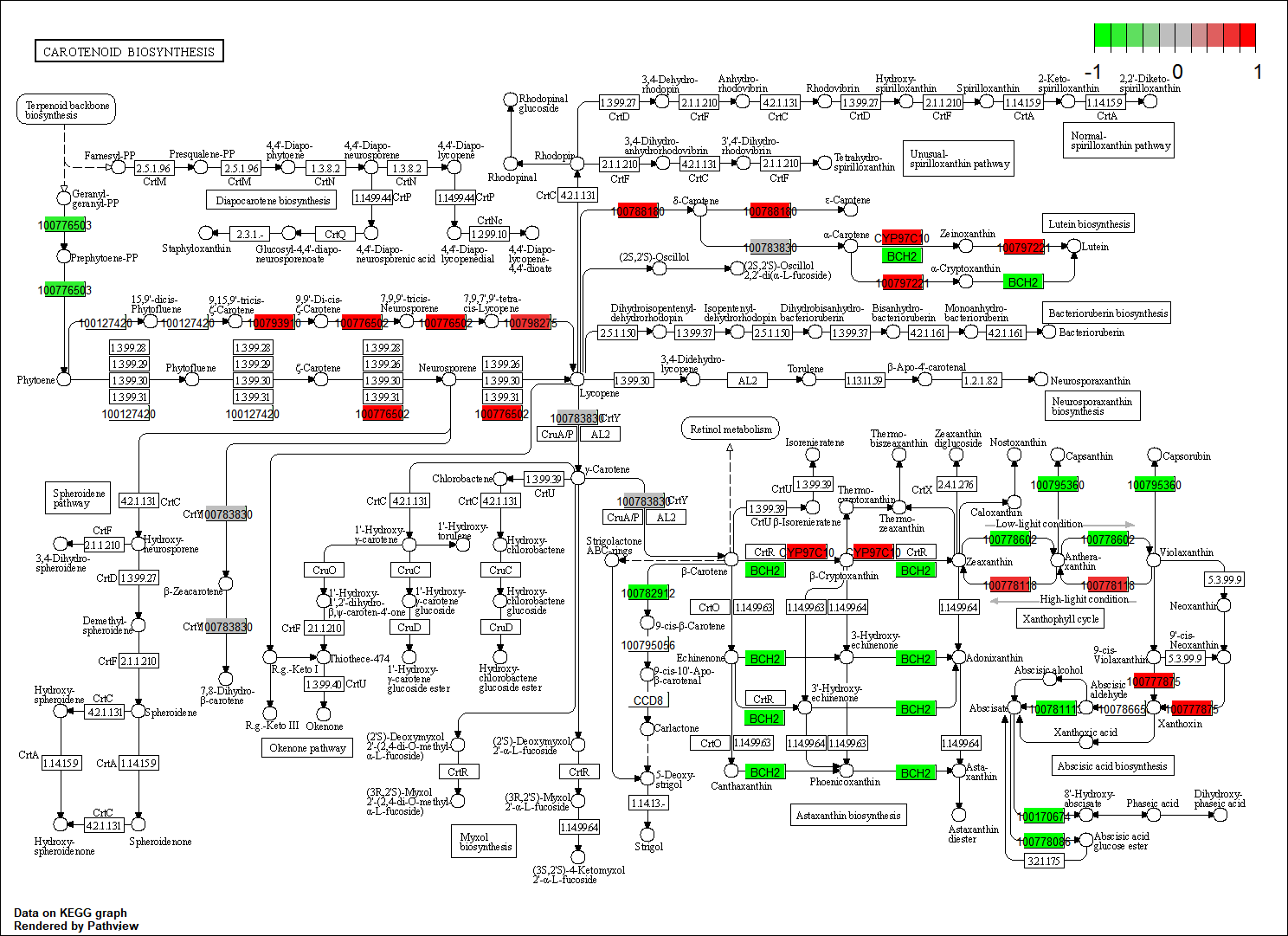

Supplement: Supplementary file 6 — Dataset S6 KEGG pathway maps for all pairwise comparisons; folder names correspond to specific treatment contrasts listed in heading of Dataset S6 in the main .docx document. [file NPH-250-2599-s003.zip › b.uninfected - Bj+Da vs control, uninfected/down/gmx00906.pathview.png]

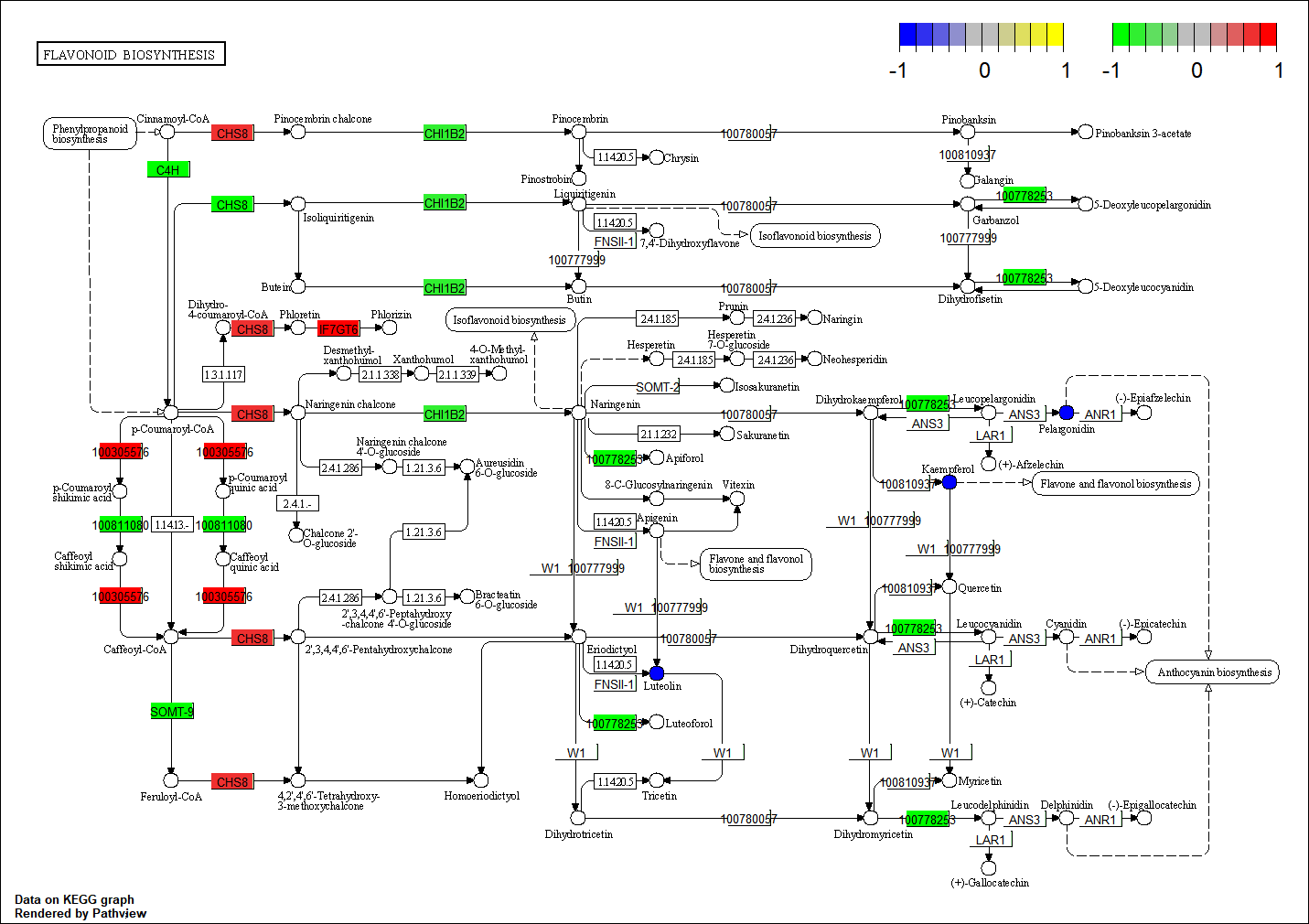

Supplement: Supplementary file 6 — Dataset S6 KEGG pathway maps for all pairwise comparisons; folder names correspond to specific treatment contrasts listed in heading of Dataset S6 in the main .docx document. [file NPH-250-2599-s003.zip › b.uninfected - Bj+Da vs control, uninfected/down/gmx00941.pathview.png]

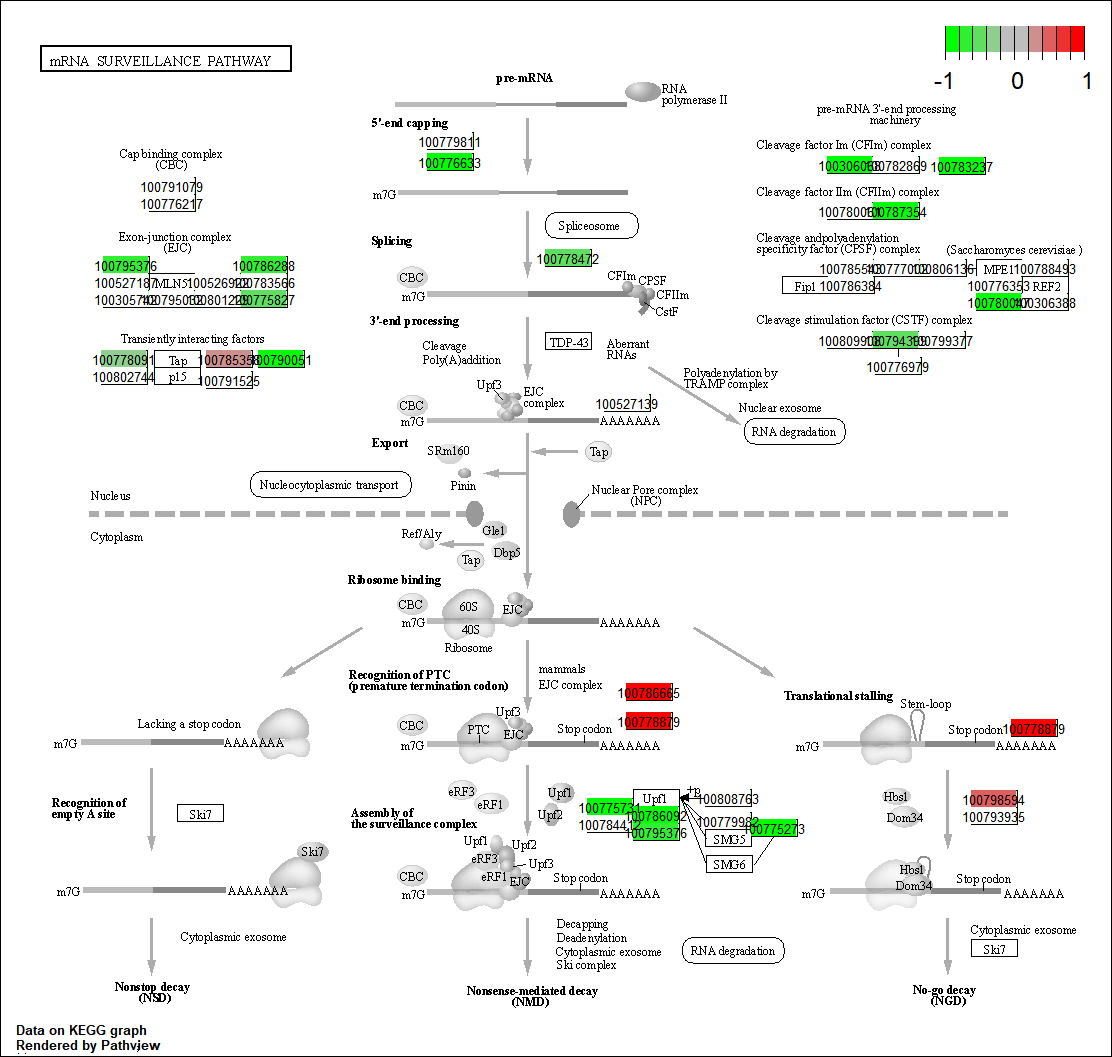

Supplement: Supplementary file 6 — Dataset S6 KEGG pathway maps for all pairwise comparisons; folder names correspond to specific treatment contrasts listed in heading of Dataset S6 in the main .docx document. [file NPH-250-2599-s003.zip › b.uninfected - Bj+Da vs control, uninfected/down/gmx03015.pathview.png]

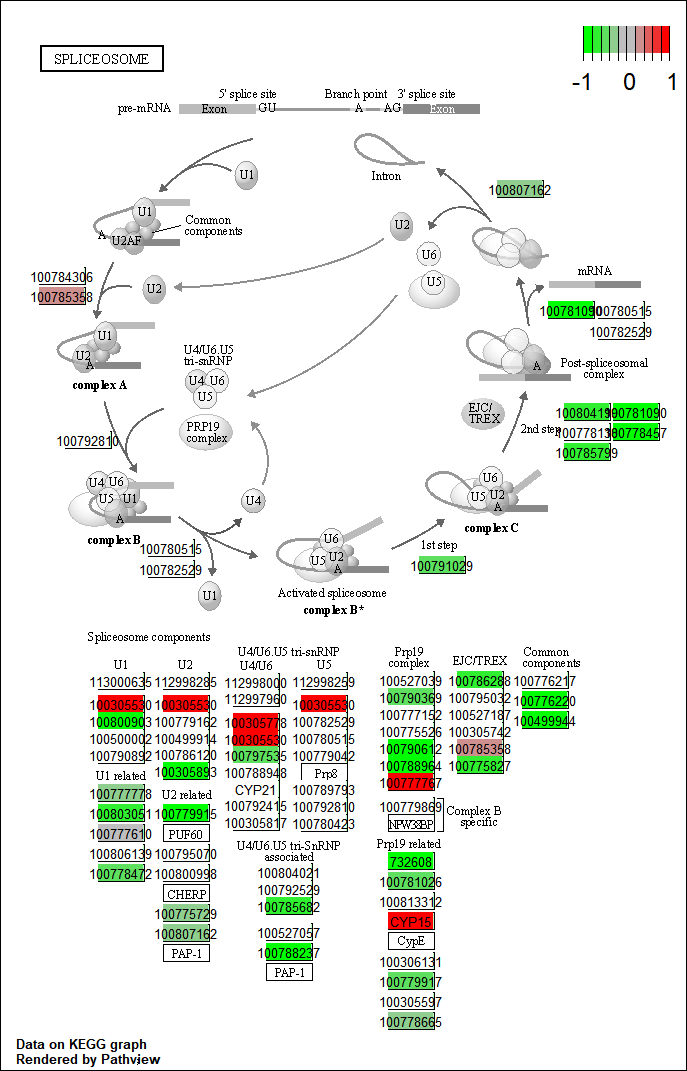

Supplement: Supplementary file 6 — Dataset S6 KEGG pathway maps for all pairwise comparisons; folder names correspond to specific treatment contrasts listed in heading of Dataset S6 in the main .docx document. [file NPH-250-2599-s003.zip › b.uninfected - Bj+Da vs control, uninfected/down/gmx03040.pathview.png]

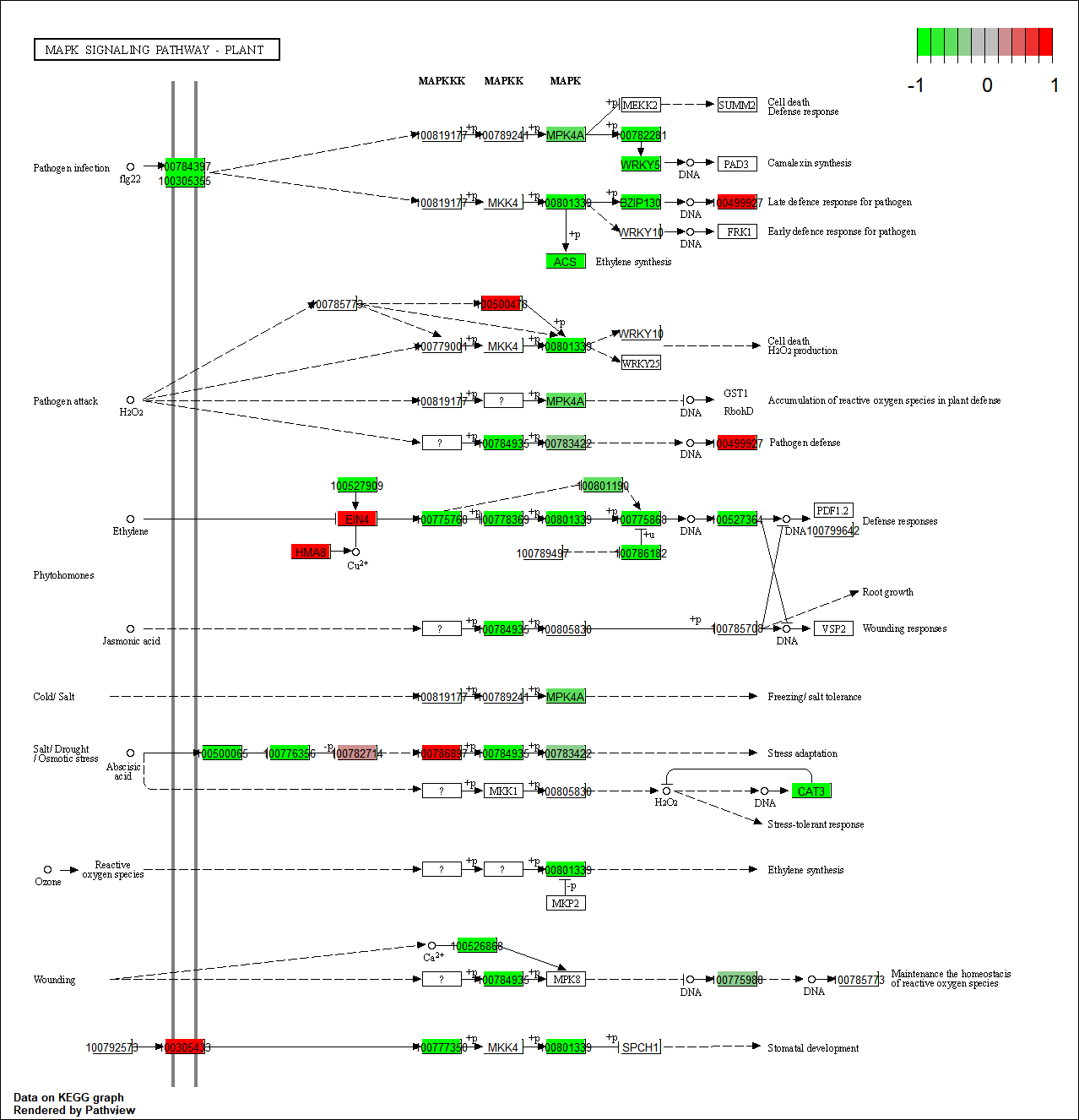

Supplement: Supplementary file 6 — Dataset S6 KEGG pathway maps for all pairwise comparisons; folder names correspond to specific treatment contrasts listed in heading of Dataset S6 in the main .docx document. [file NPH-250-2599-s003.zip › b.uninfected - Bj+Da vs control, uninfected/down/gmx04016.pathview.png]

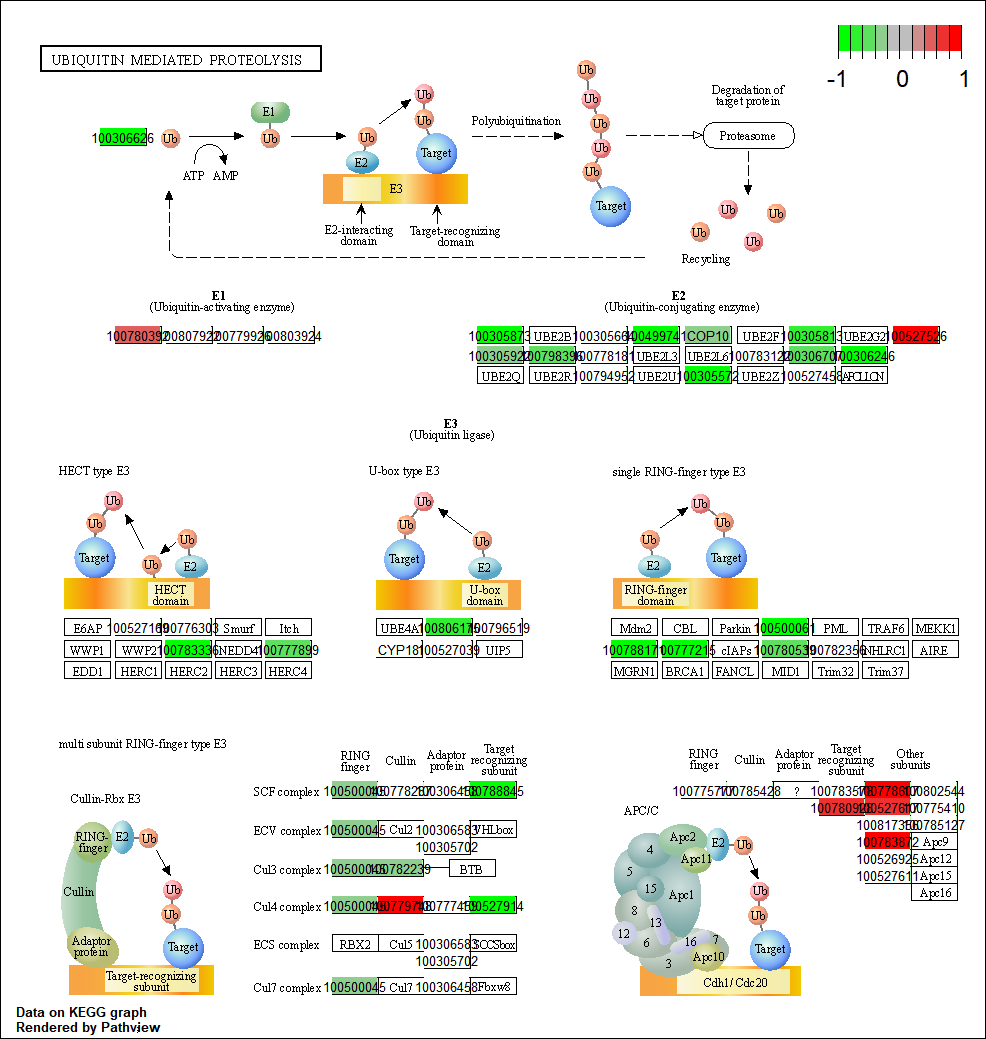

Supplement: Supplementary file 6 — Dataset S6 KEGG pathway maps for all pairwise comparisons; folder names correspond to specific treatment contrasts listed in heading of Dataset S6 in the main .docx document. [file NPH-250-2599-s003.zip › b.uninfected - Bj+Da vs control, uninfected/down/gmx04120.pathview.png]

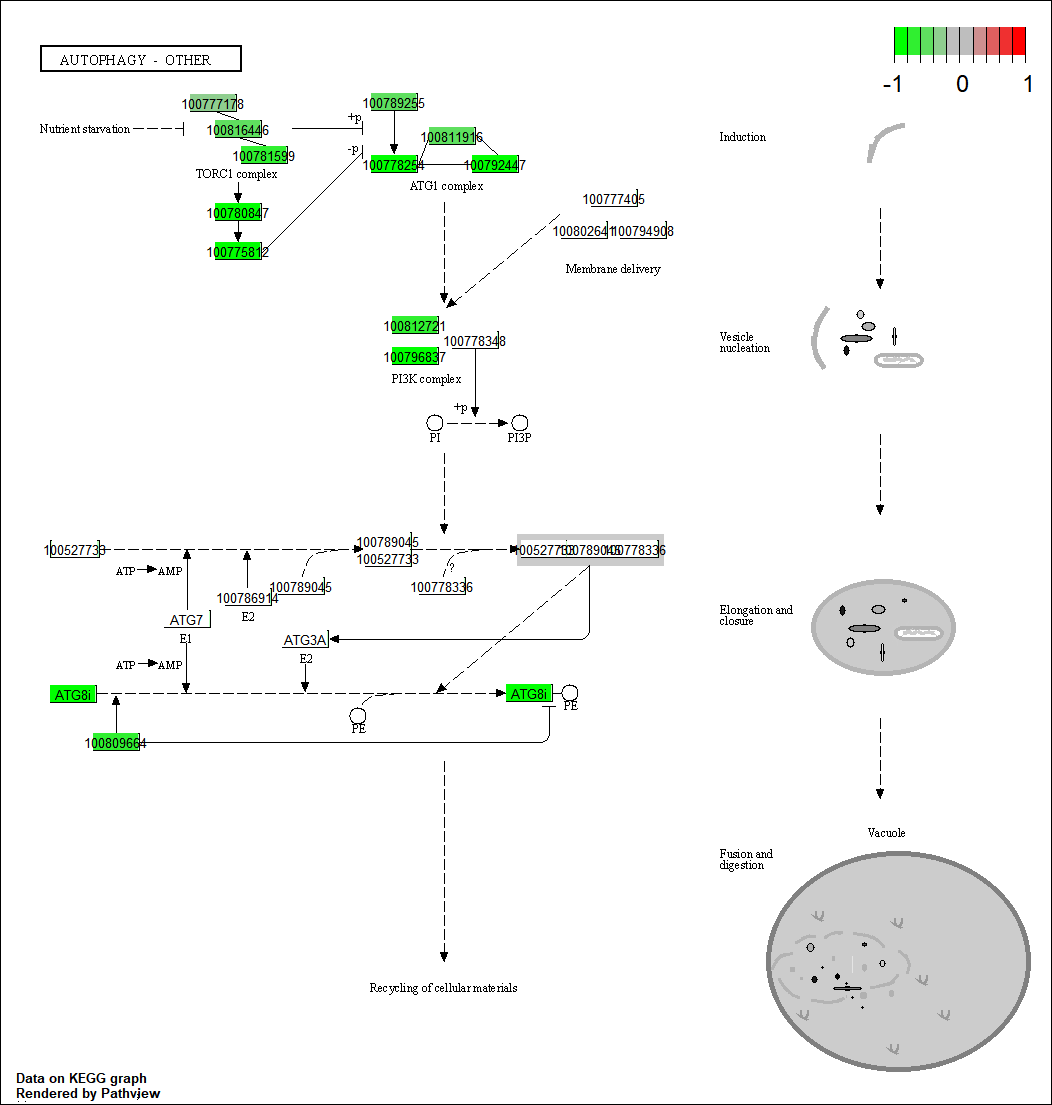

Supplement: Supplementary file 6 — Dataset S6 KEGG pathway maps for all pairwise comparisons; folder names correspond to specific treatment contrasts listed in heading of Dataset S6 in the main .docx document. [file NPH-250-2599-s003.zip › b.uninfected - Bj+Da vs control, uninfected/down/gmx04136.pathview.png]

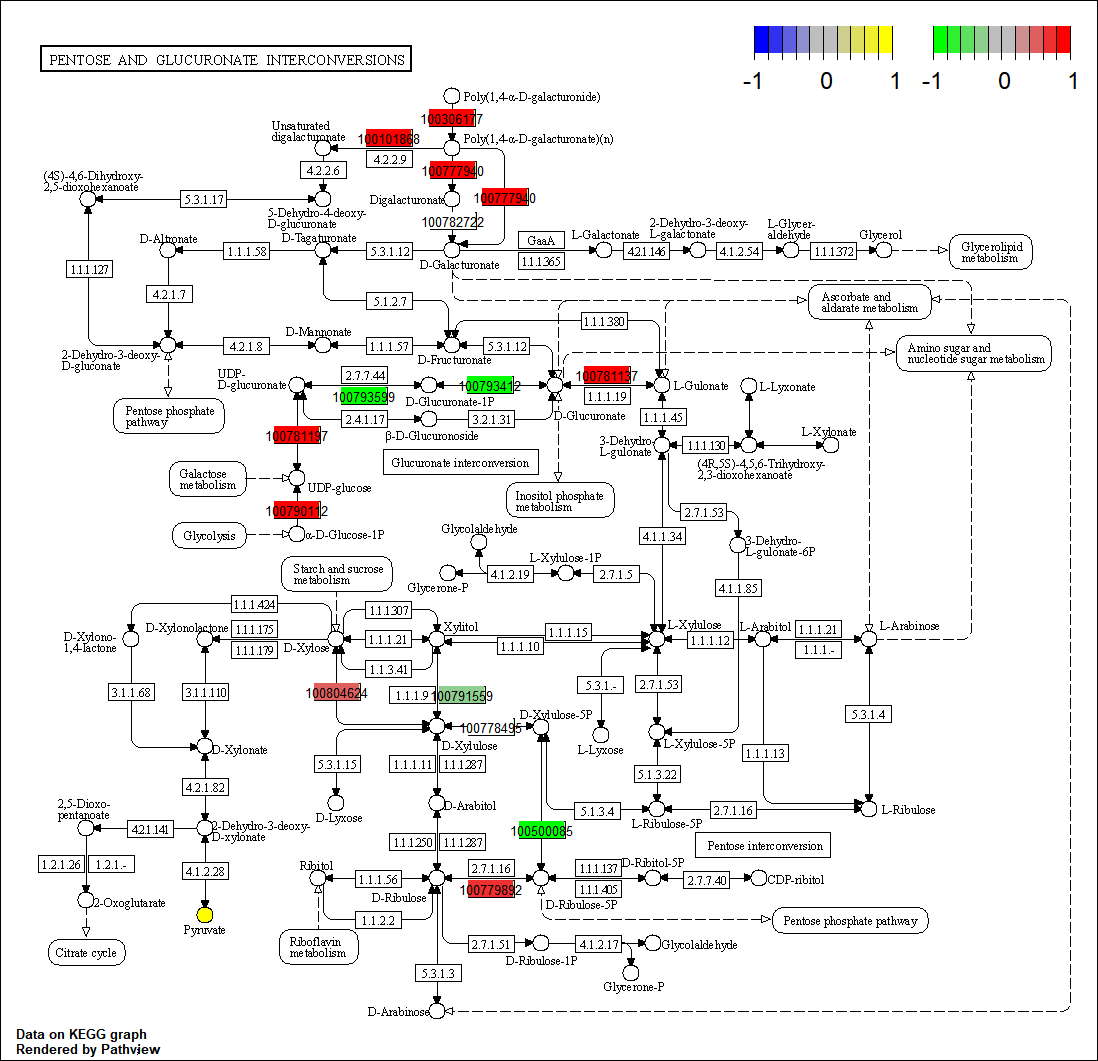

Supplement: Supplementary file 6 — Dataset S6 KEGG pathway maps for all pairwise comparisons; folder names correspond to specific treatment contrasts listed in heading of Dataset S6 in the main .docx document. [file NPH-250-2599-s003.zip › b.uninfected - Bj+Da vs control, uninfected/up/gmx00040.pathview.png]

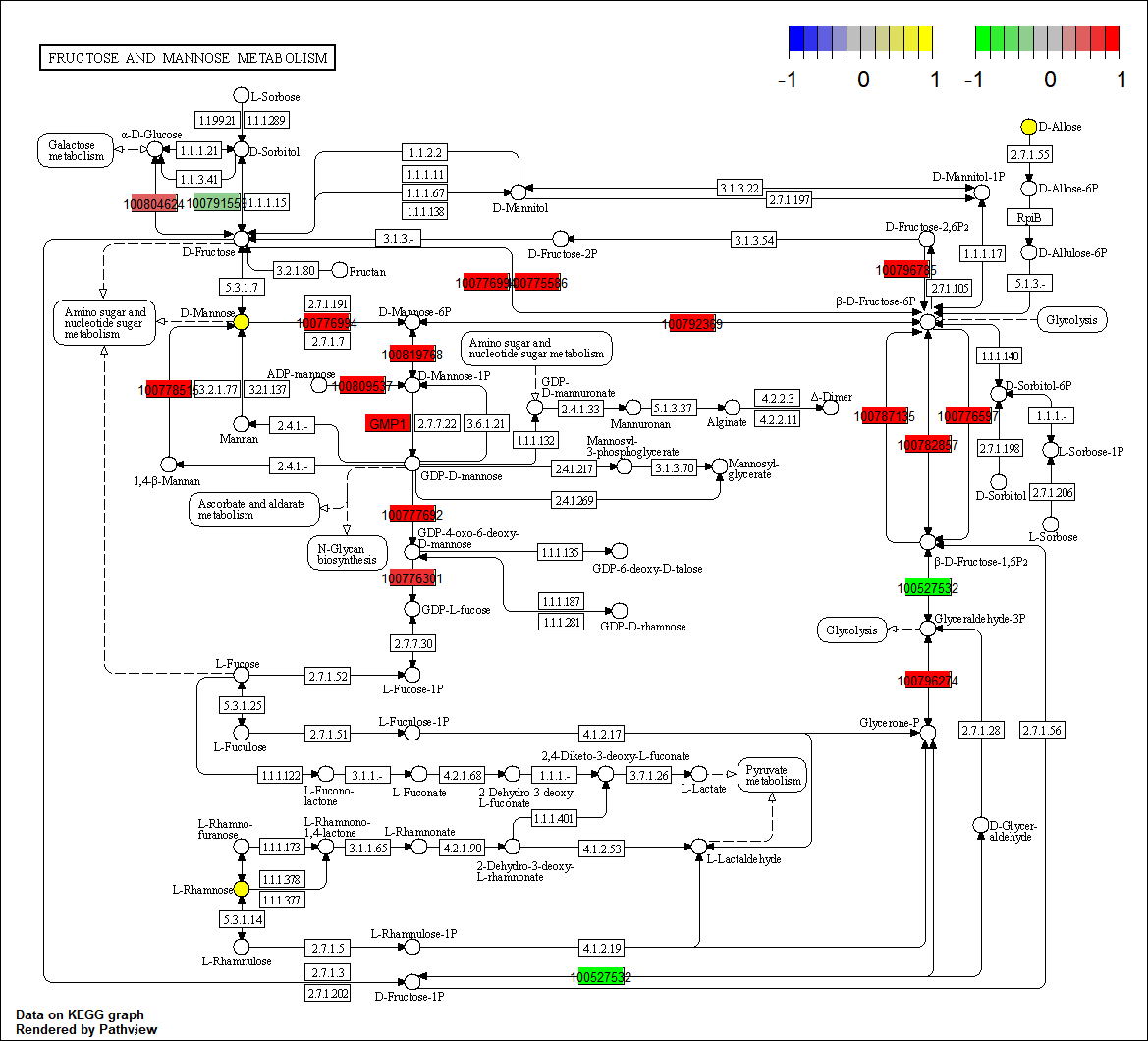

Supplement: Supplementary file 6 — Dataset S6 KEGG pathway maps for all pairwise comparisons; folder names correspond to specific treatment contrasts listed in heading of Dataset S6 in the main .docx document. [file NPH-250-2599-s003.zip › b.uninfected - Bj+Da vs control, uninfected/up/gmx00051.pathview.png]

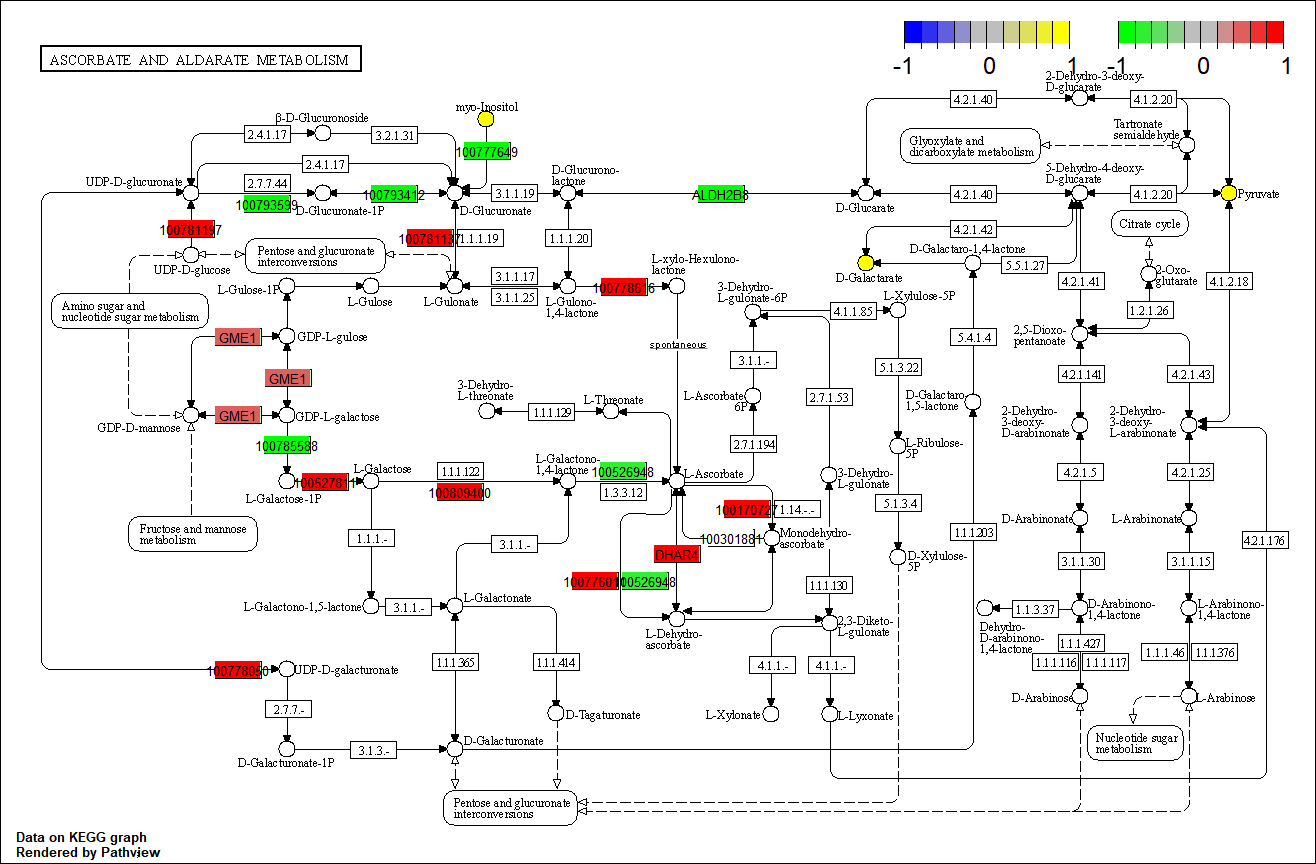

Supplement: Supplementary file 6 — Dataset S6 KEGG pathway maps for all pairwise comparisons; folder names correspond to specific treatment contrasts listed in heading of Dataset S6 in the main .docx document. [file NPH-250-2599-s003.zip › b.uninfected - Bj+Da vs control, uninfected/up/gmx00053.pathview.png]

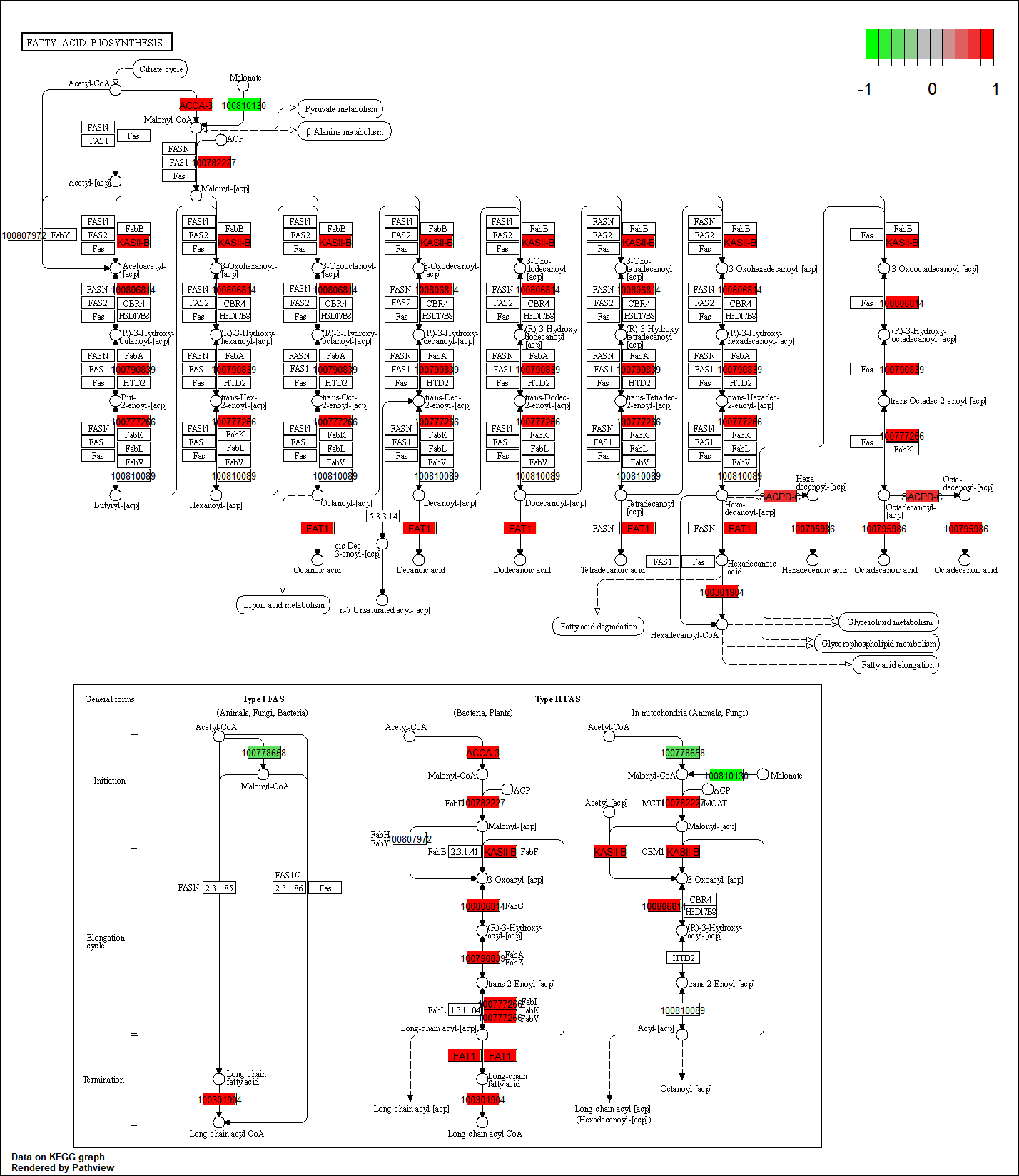

Supplement: Supplementary file 6 — Dataset S6 KEGG pathway maps for all pairwise comparisons; folder names correspond to specific treatment contrasts listed in heading of Dataset S6 in the main .docx document. [file NPH-250-2599-s003.zip › b.uninfected - Bj+Da vs control, uninfected/up/gmx00061.pathview.png]

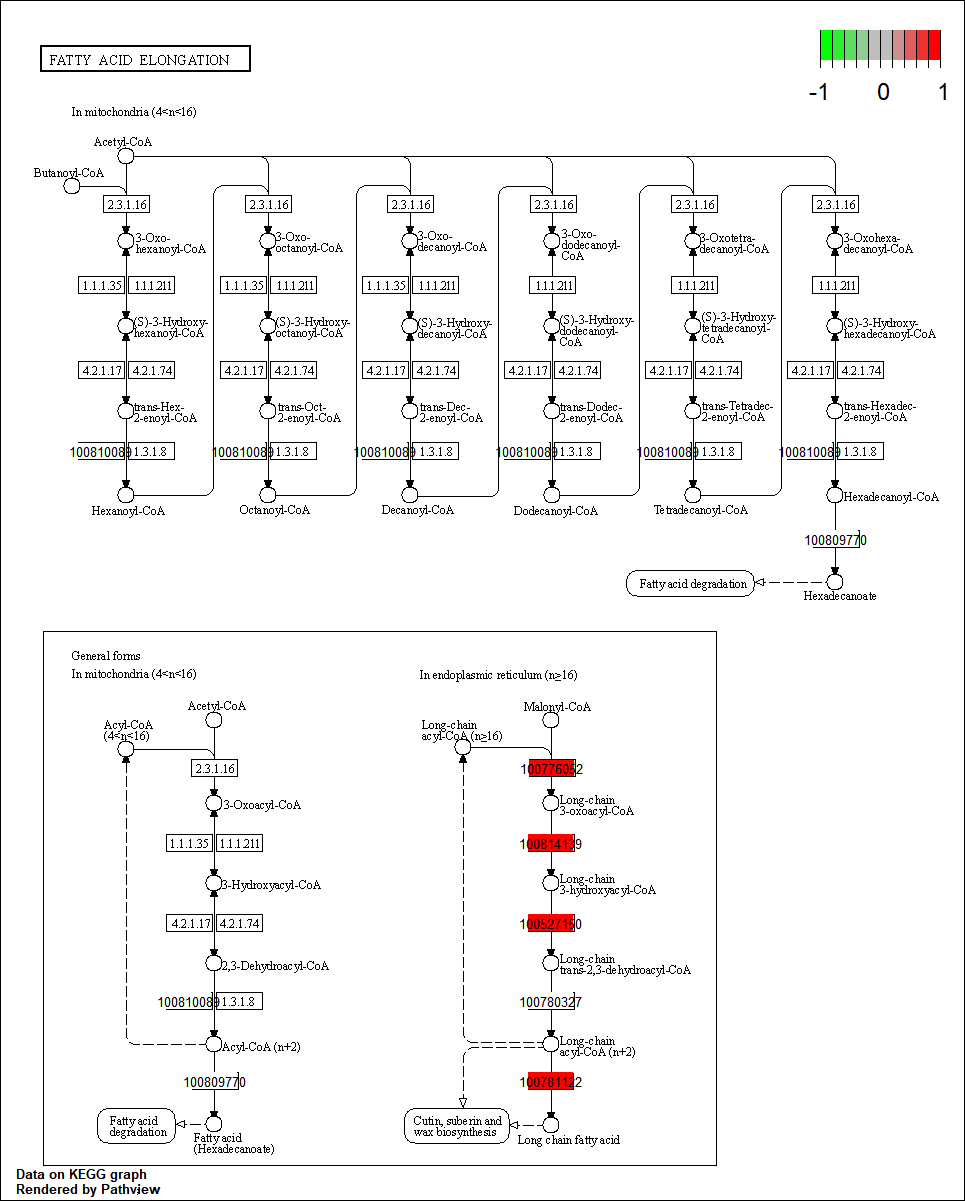

Supplement: Supplementary file 6 — Dataset S6 KEGG pathway maps for all pairwise comparisons; folder names correspond to specific treatment contrasts listed in heading of Dataset S6 in the main .docx document. [file NPH-250-2599-s003.zip › b.uninfected - Bj+Da vs control, uninfected/up/gmx00062.pathview.png]

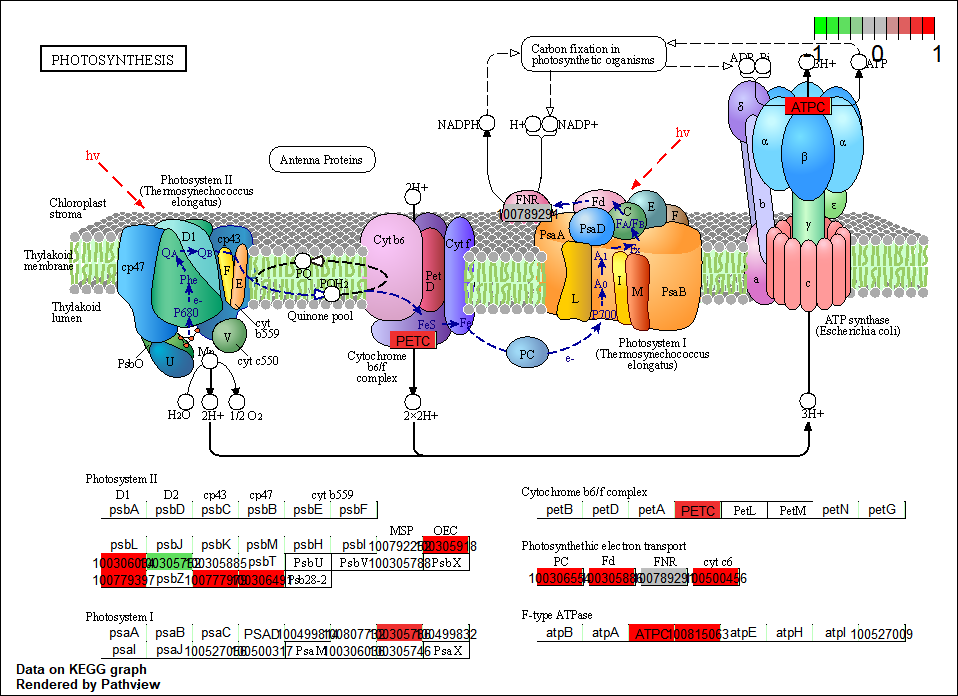

Supplement: Supplementary file 6 — Dataset S6 KEGG pathway maps for all pairwise comparisons; folder names correspond to specific treatment contrasts listed in heading of Dataset S6 in the main .docx document. [file NPH-250-2599-s003.zip › b.uninfected - Bj+Da vs control, uninfected/up/gmx00195.pathview.png]

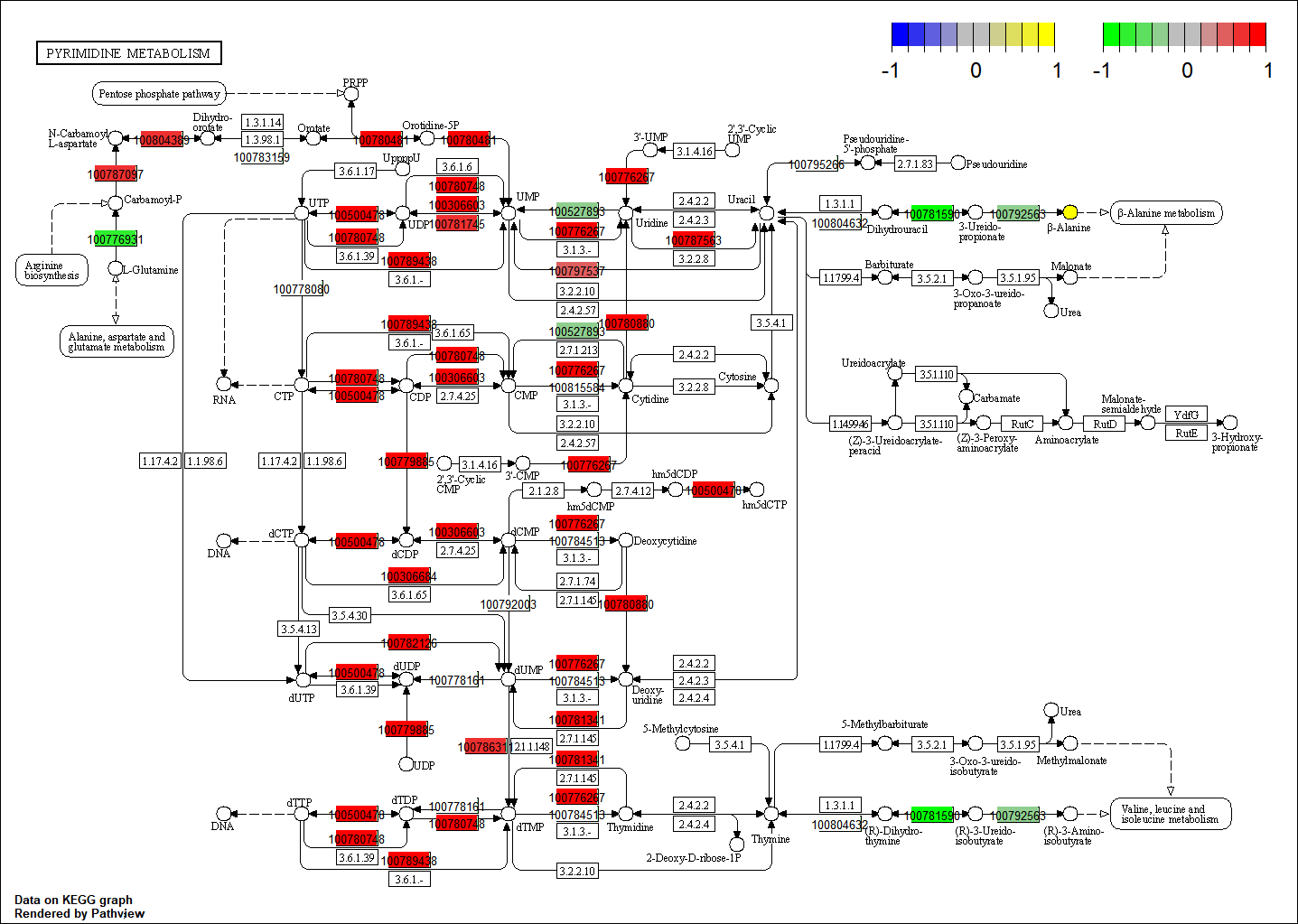

Supplement: Supplementary file 6 — Dataset S6 KEGG pathway maps for all pairwise comparisons; folder names correspond to specific treatment contrasts listed in heading of Dataset S6 in the main .docx document. [file NPH-250-2599-s003.zip › b.uninfected - Bj+Da vs control, uninfected/up/gmx00240.pathview.png]

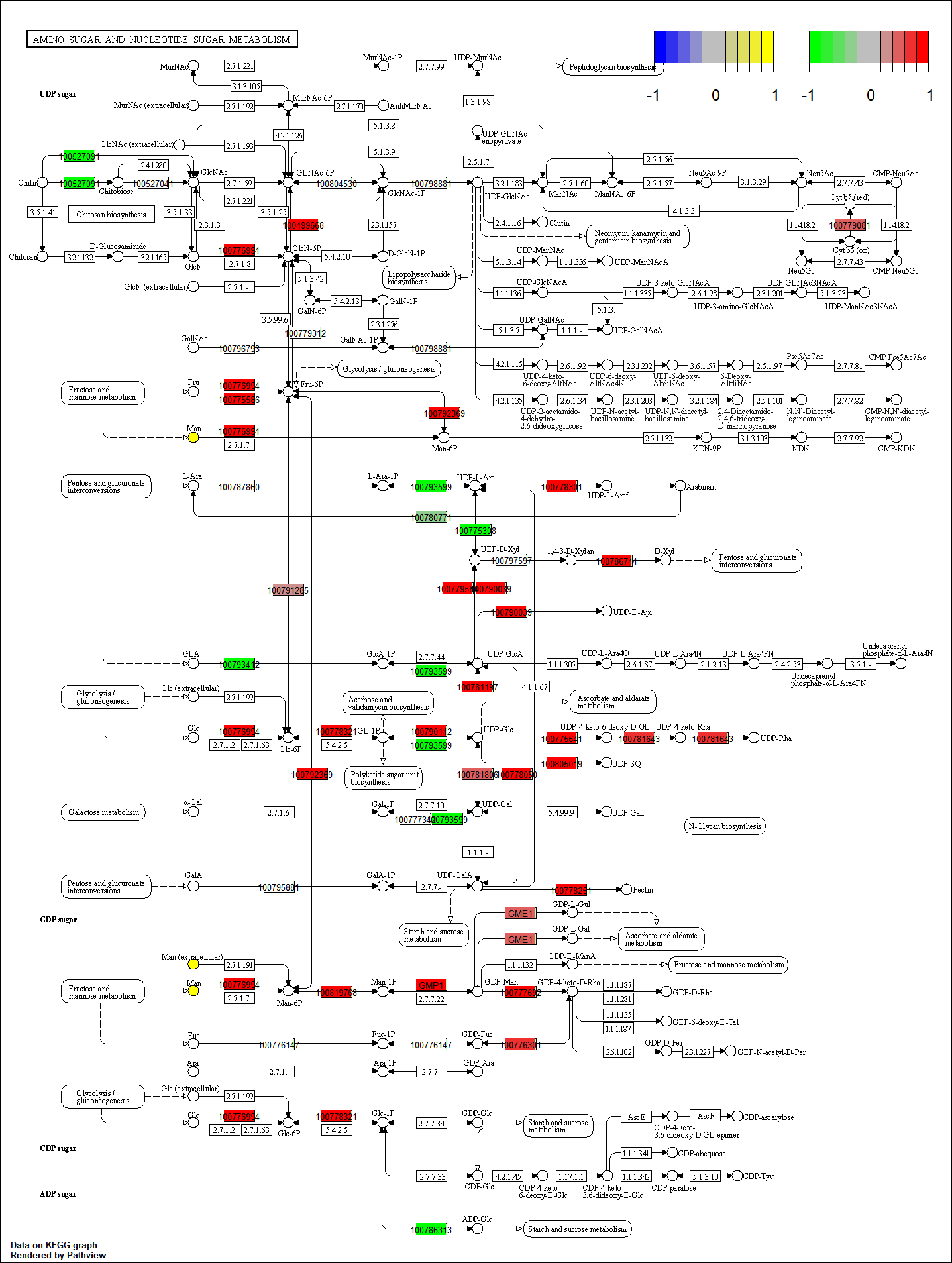

Supplement: Supplementary file 6 — Dataset S6 KEGG pathway maps for all pairwise comparisons; folder names correspond to specific treatment contrasts listed in heading of Dataset S6 in the main .docx document. [file NPH-250-2599-s003.zip › b.uninfected - Bj+Da vs control, uninfected/up/gmx00520.pathview.png]

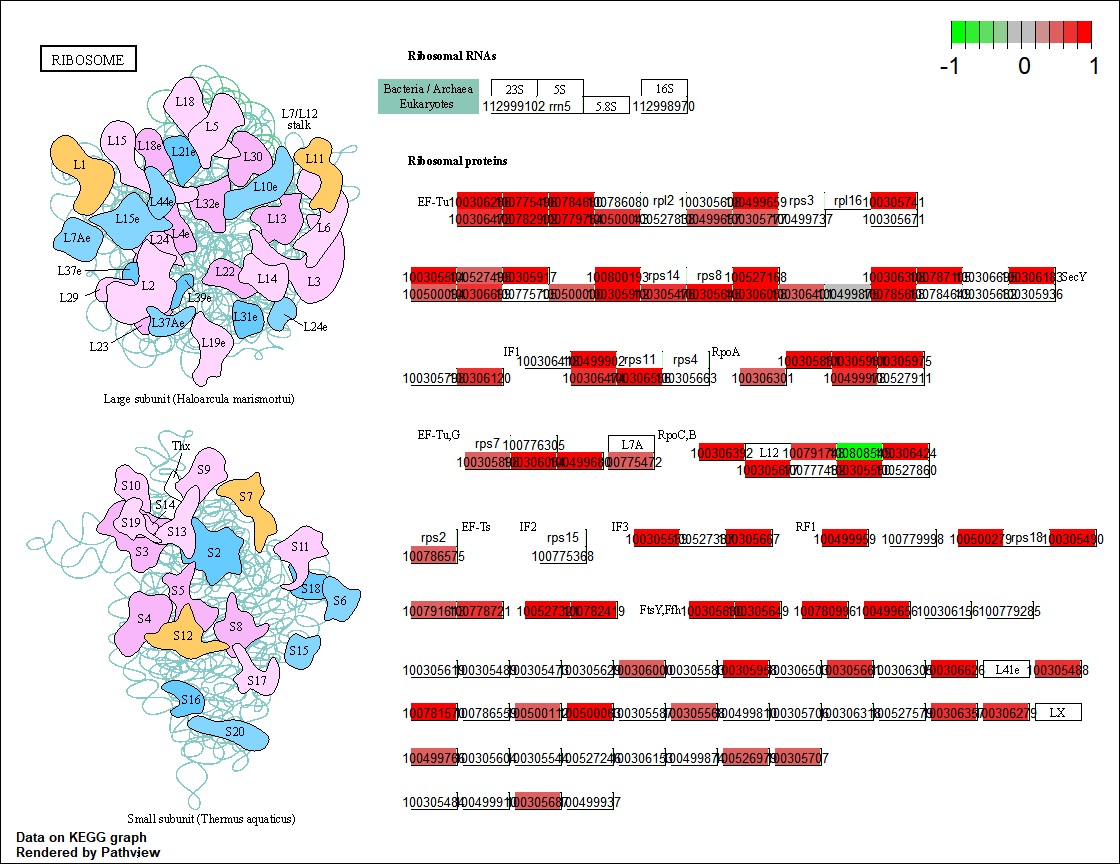

Supplement: Supplementary file 6 — Dataset S6 KEGG pathway maps for all pairwise comparisons; folder names correspond to specific treatment contrasts listed in heading of Dataset S6 in the main .docx document. [file NPH-250-2599-s003.zip › b.uninfected - Bj+Da vs control, uninfected/up/gmx03010.pathview.png]

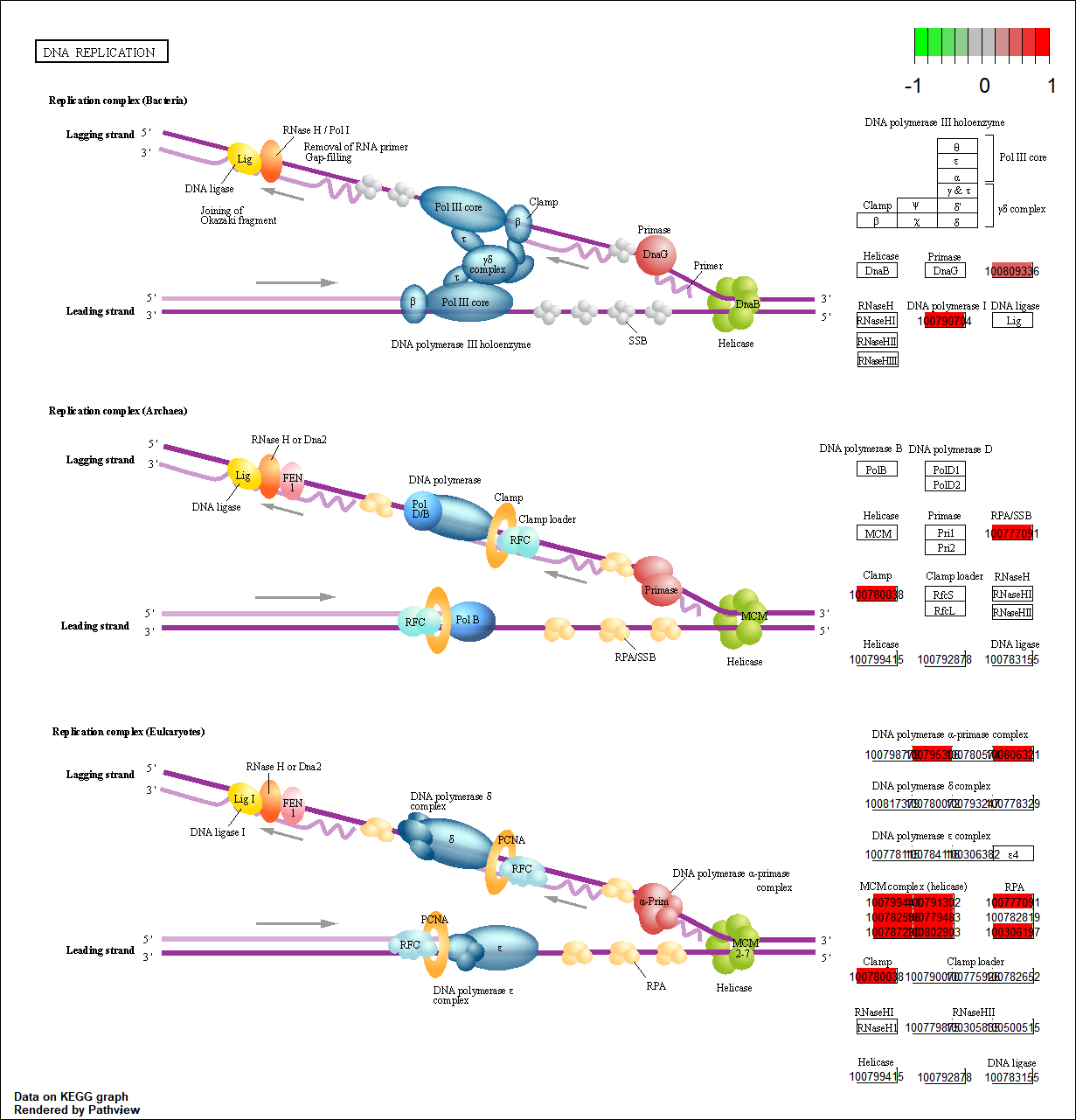

Supplement: Supplementary file 6 — Dataset S6 KEGG pathway maps for all pairwise comparisons; folder names correspond to specific treatment contrasts listed in heading of Dataset S6 in the main .docx document. [file NPH-250-2599-s003.zip › b.uninfected - Bj+Da vs control, uninfected/up/gmx03030.pathview.png]

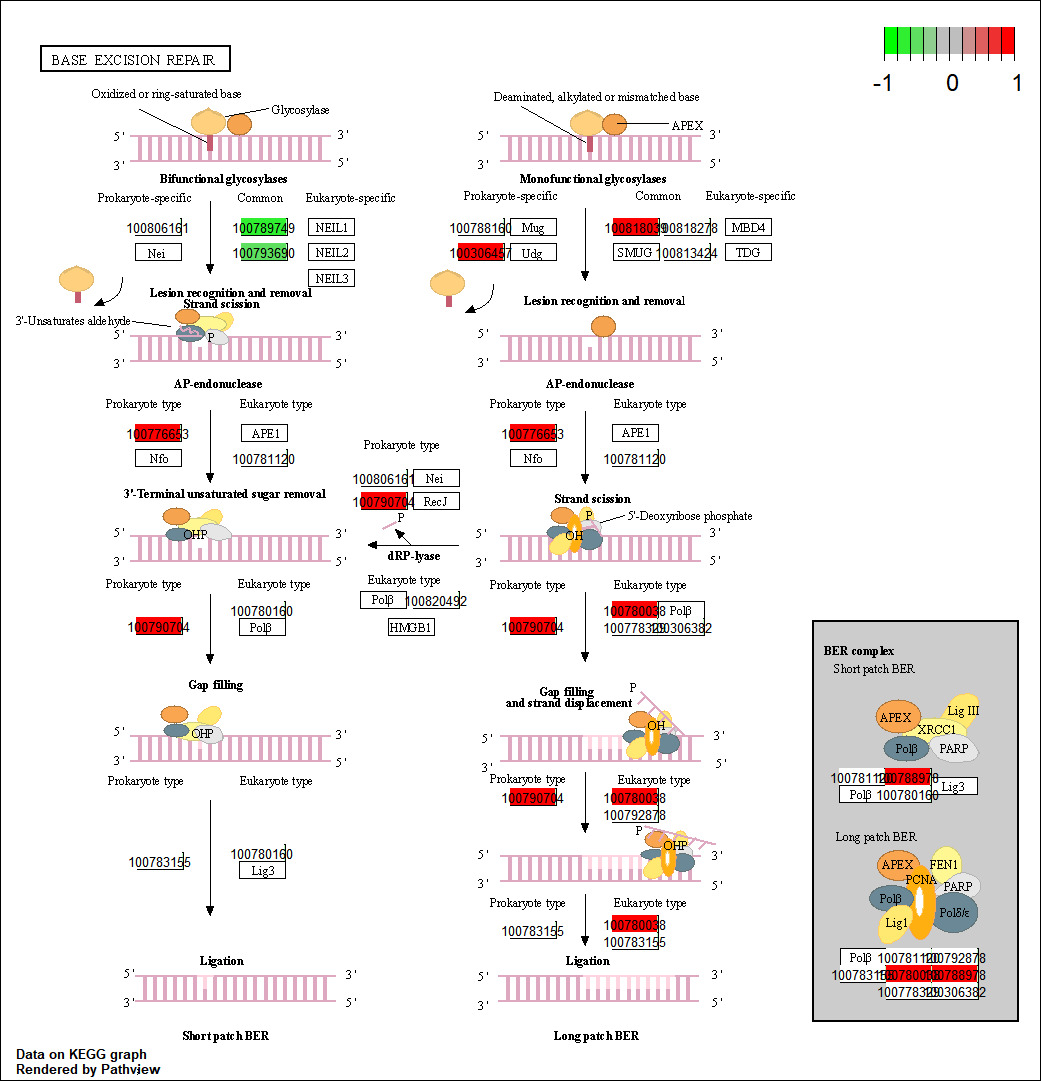

Supplement: Supplementary file 6 — Dataset S6 KEGG pathway maps for all pairwise comparisons; folder names correspond to specific treatment contrasts listed in heading of Dataset S6 in the main .docx document. [file NPH-250-2599-s003.zip › b.uninfected - Bj+Da vs control, uninfected/up/gmx03410.pathview.png]

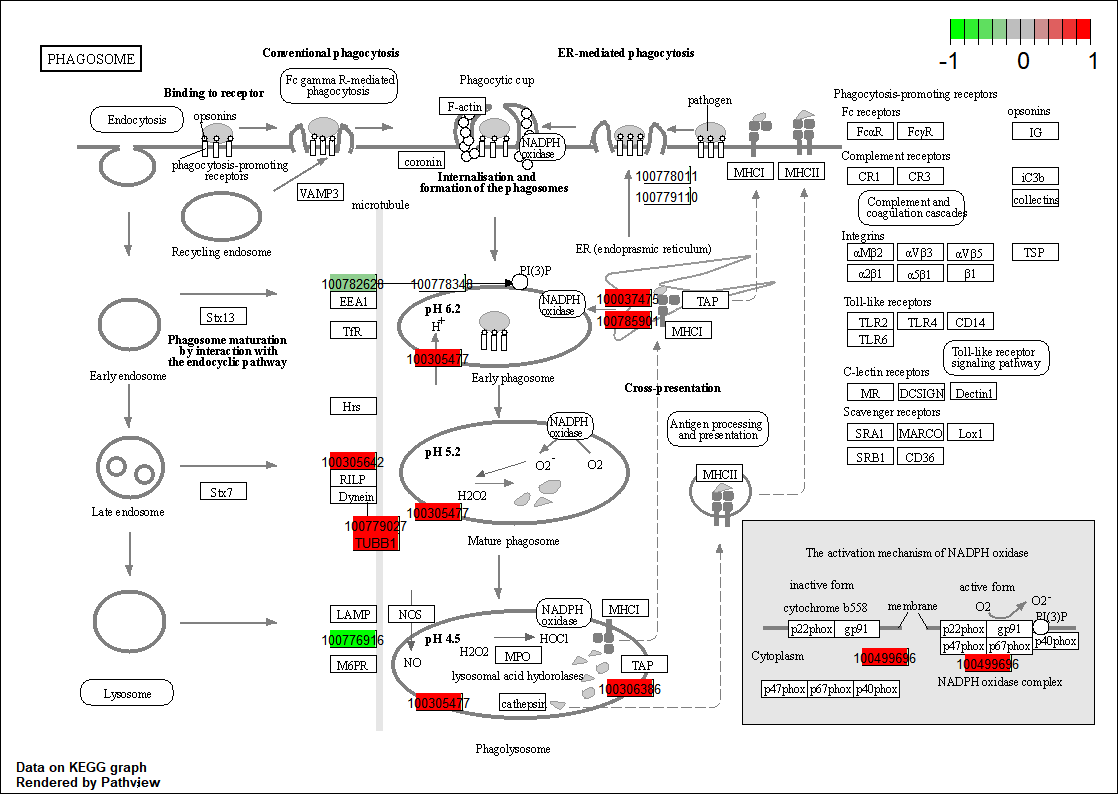

Supplement: Supplementary file 6 — Dataset S6 KEGG pathway maps for all pairwise comparisons; folder names correspond to specific treatment contrasts listed in heading of Dataset S6 in the main .docx document. [file NPH-250-2599-s003.zip › b.uninfected - Bj+Da vs control, uninfected/up/gmx04145.pathview.png]

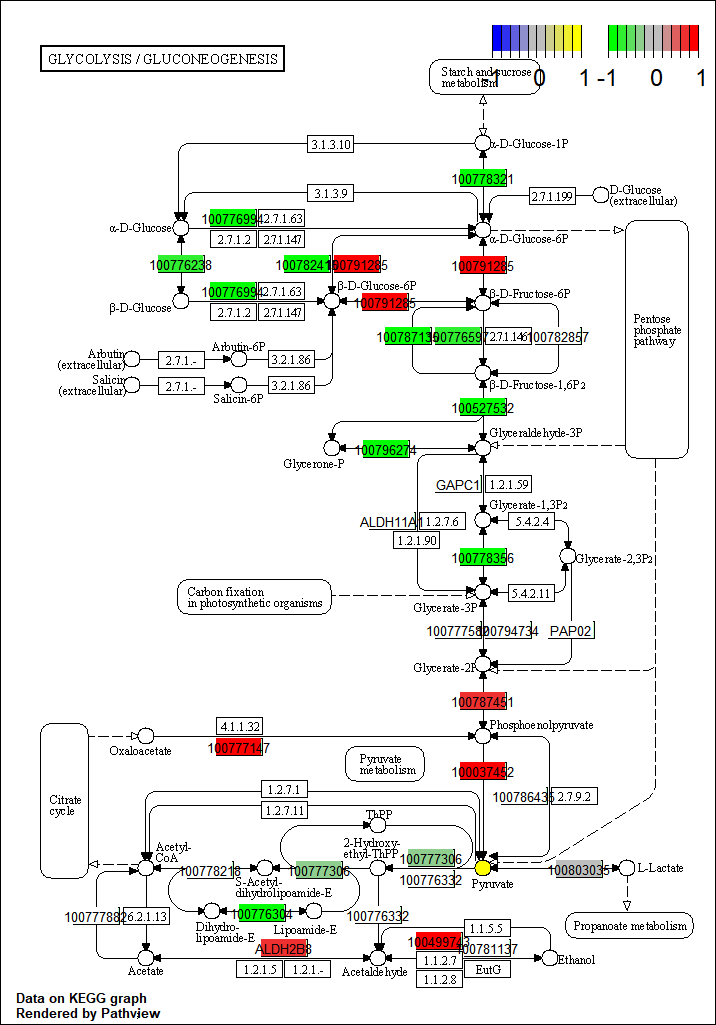

Supplement: Supplementary file 6 — Dataset S6 KEGG pathway maps for all pairwise comparisons; folder names correspond to specific treatment contrasts listed in heading of Dataset S6 in the main .docx document. [file NPH-250-2599-s003.zip › b.mixed - Bj+Da BPMV vs Bj+Da uninfected/down/gmx00010.pathview.png]

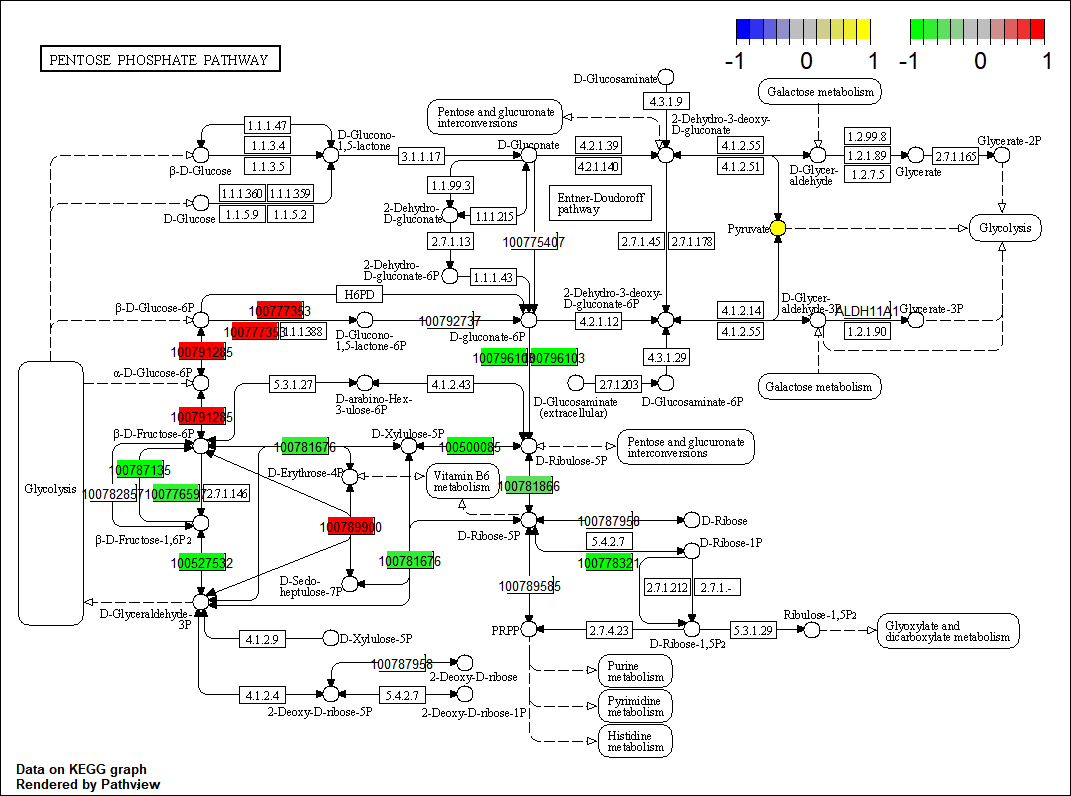

Supplement: Supplementary file 6 — Dataset S6 KEGG pathway maps for all pairwise comparisons; folder names correspond to specific treatment contrasts listed in heading of Dataset S6 in the main .docx document. [file NPH-250-2599-s003.zip › b.mixed - Bj+Da BPMV vs Bj+Da uninfected/down/gmx00030.pathview.png]

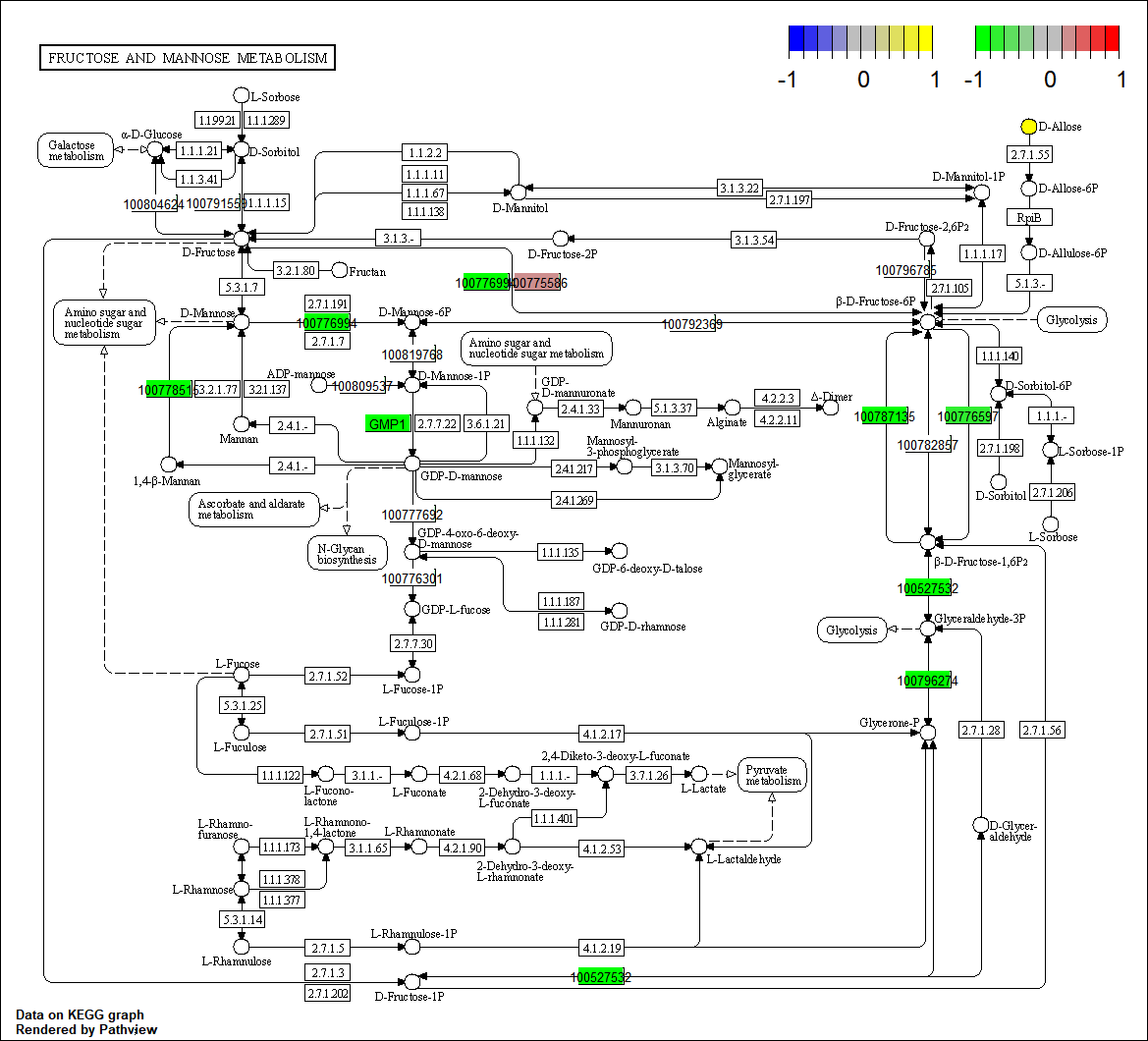

Supplement: Supplementary file 6 — Dataset S6 KEGG pathway maps for all pairwise comparisons; folder names correspond to specific treatment contrasts listed in heading of Dataset S6 in the main .docx document. [file NPH-250-2599-s003.zip › b.mixed - Bj+Da BPMV vs Bj+Da uninfected/down/gmx00051.pathview.png]

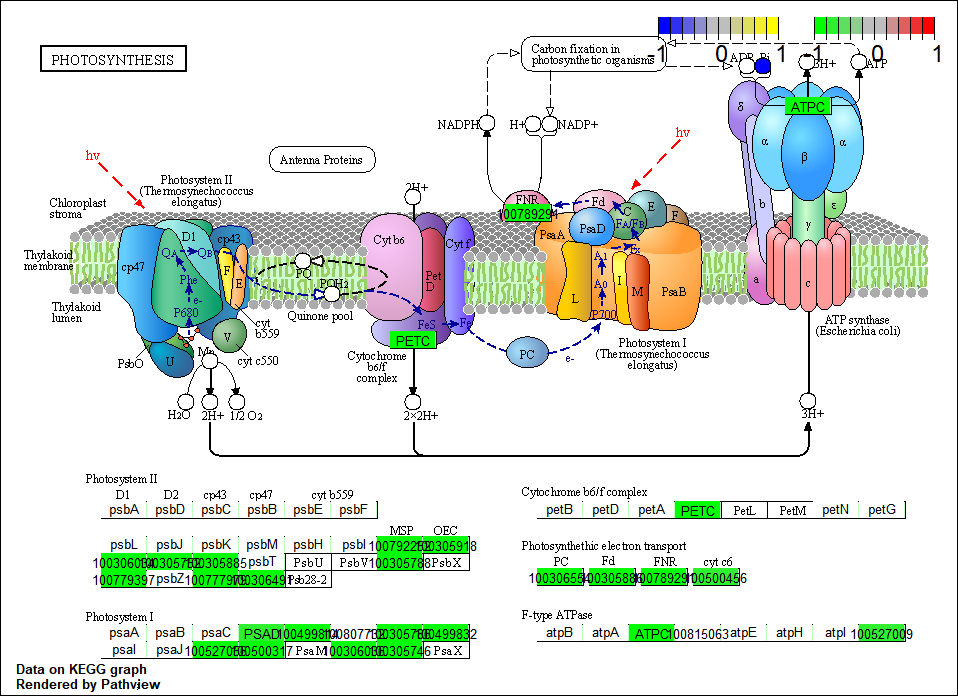

Supplement: Supplementary file 6 — Dataset S6 KEGG pathway maps for all pairwise comparisons; folder names correspond to specific treatment contrasts listed in heading of Dataset S6 in the main .docx document. [file NPH-250-2599-s003.zip › b.mixed - Bj+Da BPMV vs Bj+Da uninfected/down/gmx00195.pathview.png]

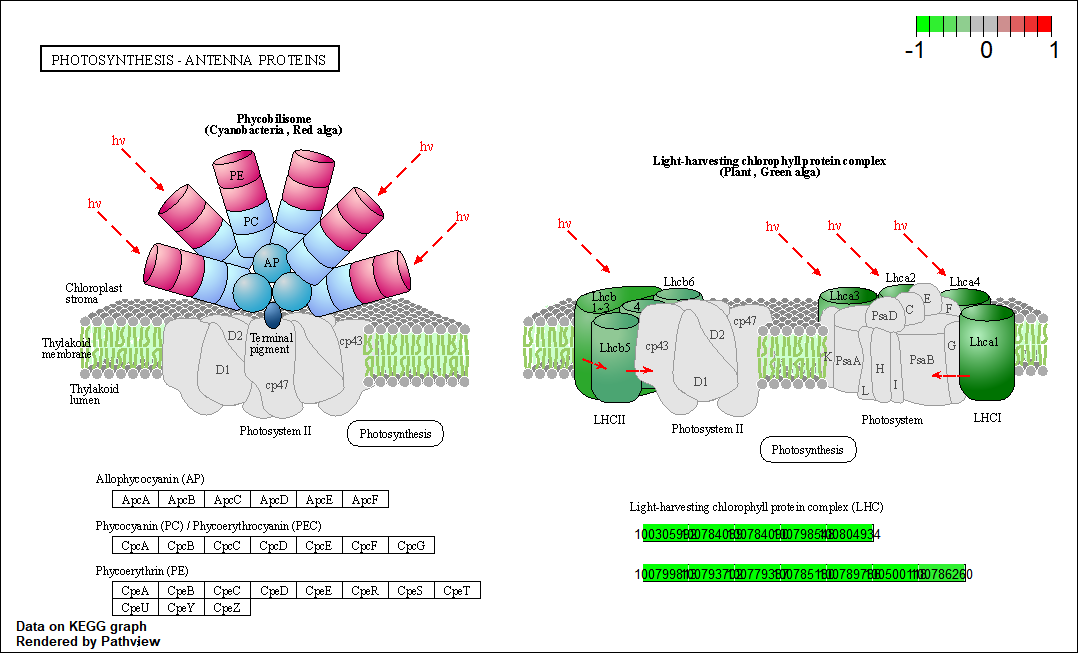

Supplement: Supplementary file 6 — Dataset S6 KEGG pathway maps for all pairwise comparisons; folder names correspond to specific treatment contrasts listed in heading of Dataset S6 in the main .docx document. [file NPH-250-2599-s003.zip › b.mixed - Bj+Da BPMV vs Bj+Da uninfected/down/gmx00196.pathview.png]

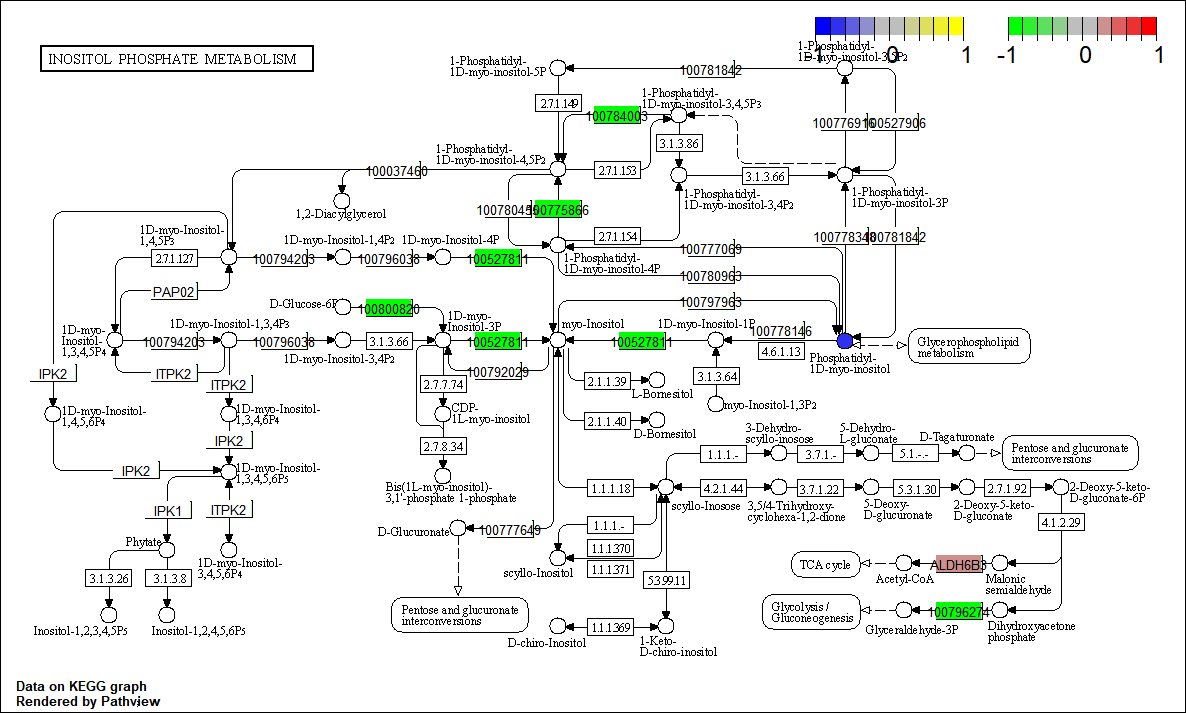

Supplement: Supplementary file 6 — Dataset S6 KEGG pathway maps for all pairwise comparisons; folder names correspond to specific treatment contrasts listed in heading of Dataset S6 in the main .docx document. [file NPH-250-2599-s003.zip › b.mixed - Bj+Da BPMV vs Bj+Da uninfected/down/gmx00562.pathview.png]

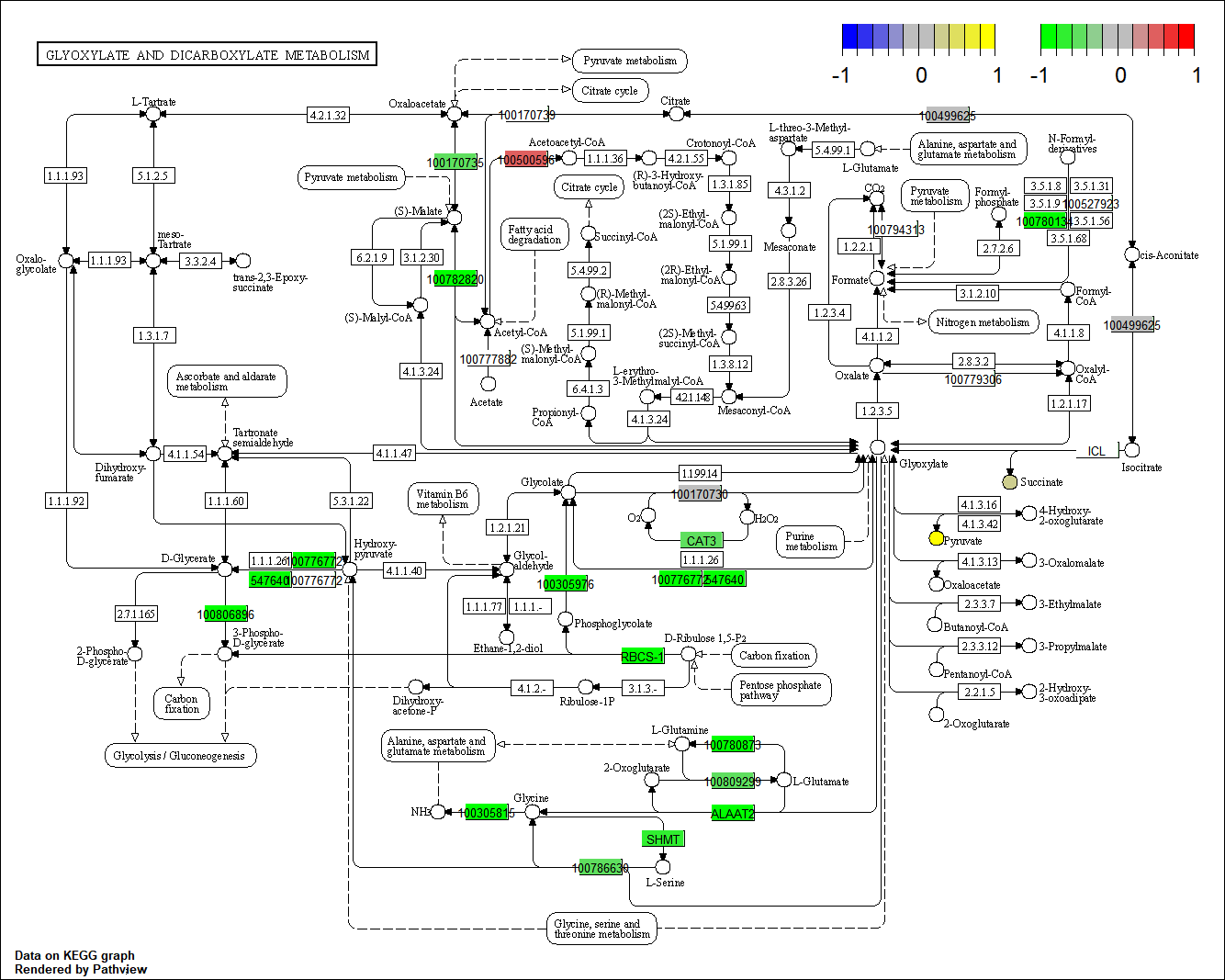

Supplement: Supplementary file 6 — Dataset S6 KEGG pathway maps for all pairwise comparisons; folder names correspond to specific treatment contrasts listed in heading of Dataset S6 in the main .docx document. [file NPH-250-2599-s003.zip › b.mixed - Bj+Da BPMV vs Bj+Da uninfected/down/gmx00630.pathview.png]

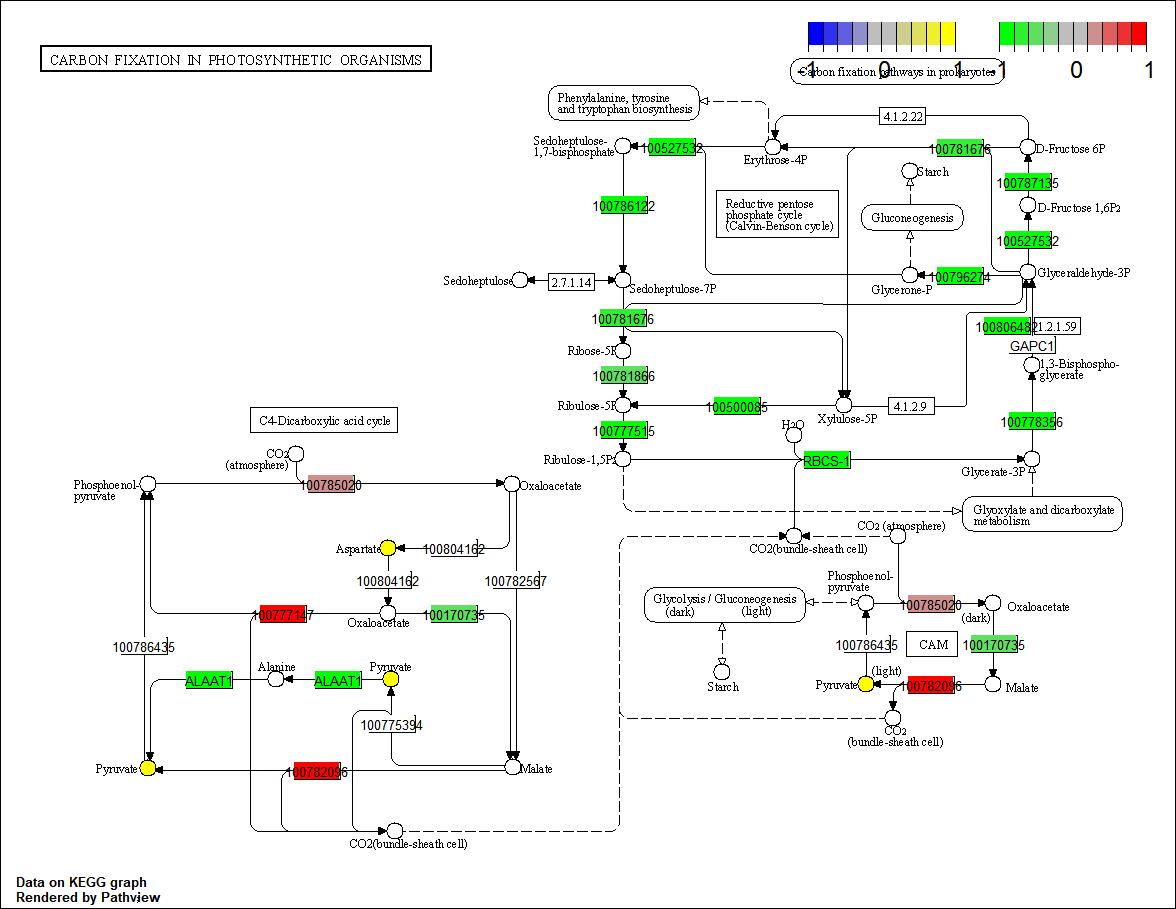

Supplement: Supplementary file 6 — Dataset S6 KEGG pathway maps for all pairwise comparisons; folder names correspond to specific treatment contrasts listed in heading of Dataset S6 in the main .docx document. [file NPH-250-2599-s003.zip › b.mixed - Bj+Da BPMV vs Bj+Da uninfected/down/gmx00710.pathview.png]
