# Supplementary figures and images for: Beneficial rhizobacteria and virus infection modulate the soybean metabolome and influence the feeding preferences of the virus vector Epilachna varivestis (part 3 of 3)
Source: New Phytol. 2026 Mar 24;250(4):2599–618. doi: 10.1111/nph.71104 (PMC13103440; doi:10.1111/nph.71104)

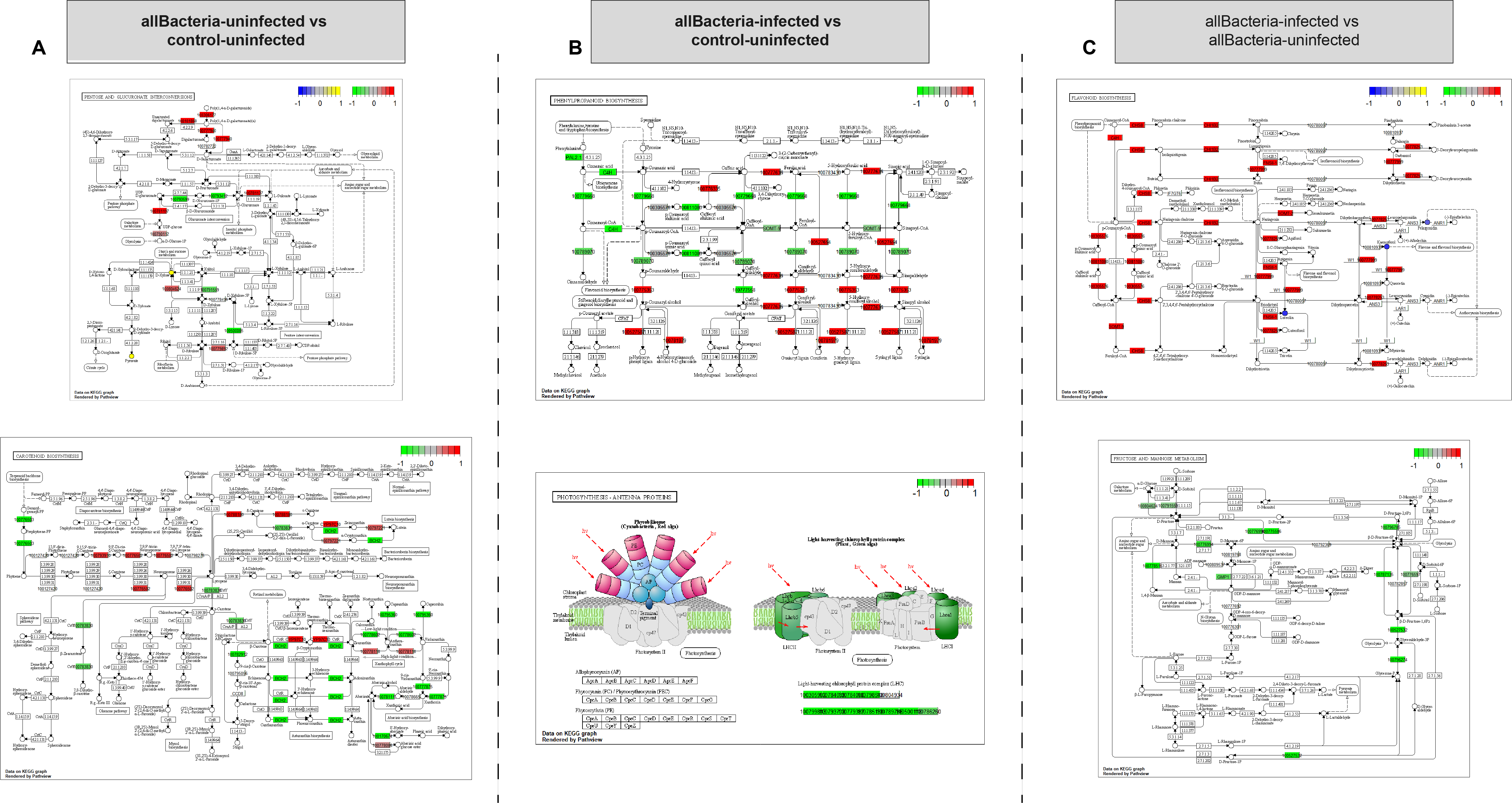

Supplement: Supplementary file 7 — Dataset S7 Summary KEGG pathway diagrams for three major contrasts corresponding to Fig. 9. Fig. S1 Experimental setup illustrating adult beetle dual‐choice feeding and foraging assays. Fig. S2 Volcano plots showing rhizobacteria‐induced metabolite changes in uninfected soybean plants. Fig. S3 Volcano plots showing rhizobacteria‐induced metabolite changes in BPMV‐infected soybean plants. Fig. S4 Volcano plots showing BPMV‐induced metabolite changes under rhizobacteria and mixed treatment conditions. Fig. S5 Number of signature genes identified per pairwise treatment comparison. Fig. S6 Volcano plots of rhizobacteria‐induced signature gene profiles in uninfected soybean plants. Fig. S7 Volcano plots of rhizobacteria‐induced signature gene profiles in BPMV‐infected soybean plants. Fig. S8 Volcano plots summarizing soybean gene expression changes across rhizobacteria inoculation and BPMV infection treatments. [file NPH-250-2599-s007.png]
