# Supplementary material for: Beneficial rhizobacteria and virus infection modulate the soybean metabolome and influence the feeding preferences of the virus vector Epilachna varivestis
Source: New Phytol. 2026 Mar 24;250(4):2599–618. doi: 10.1111/nph.71104 (PMC13103440; doi:10.1111/nph.71104)
Supplement: Supplementary file 8 — Methods S1 Detailed protocols for rhizobacteria culture, BPMV inoculation. Methods S2 Description of plant growth conditions, and experimental design used in soybean assays. Methods S3 Description of metabolite extraction and analysis. Methods S4 Integrative transcriptome–metabolome analyses, feature selection procedures, enrichment analyses, and pathway mapping workflows. Table S1 Full factorial design combining rhizobacteria inoculation treatments and BPMV infection status. Table S2 Exact binomial test results for all pairwise dual‐choice beetle foraging contrasts. Table S3 ANOVA results for adult beetle feeding damage across rhizobacteria and virus treatments. Table S4 ANOVA and Tukey HSD post‐hoc comparisons for larval weight across treatments. Table S5 ANOVA summary for leaf toughness across rhizobacteria and virus treatments. Table S6 ANOVA summary for leaf biomass across rhizobacteria and virus treatments. Table S7 ANOVA summary for nodule biomass across virus treatments. Table S8 Regression analyses of nodule weight vs shoot biomass across virus treatments. Table S9 Identified metabolites with compound IDs and corresponding KEGG and HMDB annotations when available. Table S10 Behavioral contrasts and statistical approaches used for signature gene and metabolite selection. Table S11 Top six hub genes for each co‐expression module highlighted in the network analysis. [file NPH-250-2599-s001.pdf]

New Phytologist Supporting Information

Article title: Beneficial rhizobacteria and virus infection modulate the soybean metabolome and influence the feeding preferences of the virus vector *Epilachna varivestis*

Authors: Hannier Pulido, Kerry E. Mauck, Consuelo M. De Moraes, Mark C. Mescher

Article acceptance date: 9 February 2026

Supplementary Information includes:

- Methods S1-S4
- Tables S1-S4
- Figures S1-S9
- Video S1
- Datasets S1-S7
- References

METHODS

Method S1. Rhizobacteria and BPMV culture conditions

To prepare inoculum sources for all experiments, cultures of *B. japonicum* and *D. acidovorans* were grown in yeast-mannitol broth and stored in 30% glycerol at -80°C. For broth culture inoculations, 50 µl of the bacterial glycerol stock was used to inoculate flasks containing broth media. The flasks were shaken at 25°C for 40 hours (*D. acidovorans*) or 80 hours (*B. japonicum*). Before seedling inoculation, the liquid inoculum was adjusted to a cell density of 1×10^9 cfu/ml. Three-day-old seedlings were inoculated with 1 ml of the bacterial suspension applied to the soil using a sterile pipette. Control plants received the same volume of rhizobacteria-free broth.

BPMV, vectored semi-persistently by *Epilachna varivestis* (Coccinellidae) and *Ceratoma trifurcata* (Chrysomelidae), causes symptoms like stunted growth, leaf mottling and distortion, and mottling on pods (Giesler *et al.*, 2002). The BPMV strain used was collected from Ohio soybeans, purified to isolate BPMV virions, and then mechanically re-inoculated to generate infected tissue (courtesy of Dr. Peg Redinbaugh). To infect soybean plants, one-week-old seedlings at the V1 stage (post-bacterial inoculation) were dusted with carborundum powder and mechanically inoculated by rubbing leaves with a buffer solution (0.1 M potassium phosphate buffer) containing ground virus-infected tissue. Mock-inoculated plants received the same mechanical treatment with virus-free buffer. Plants were visually monitored for symptom development, and infection was confirmed by ELISA at the conclusion of each experiment.

Method S2. Plant growth and experimental design

Starting from the V1 stage until harvest, plants received 50 mL of a diluted, modified Hoagland's nutrient solution (Dean *et al.*, 2014; Pulido *et al.*, 2019) three times per week. Control and Da plants received a nutrient solution containing the following nitrogen concentrations (in µM): 5001.008 NO_3^- and 1675.977 NH_4^+. For Bj and Bj+Da plants, NO_3^- and NH_4^+ were replaced with K_2SO_4 at equivalent concentrations. This design was necessary because supplemental nitrogen, even at relatively modest levels (10–15 mM nitrate), strongly inhibits nodulation and suppresses biological nitrogen fixation (Jiang *et al.*, 2020), thereby defeating the purpose of testing rhizobial symbiosis. Conversely, a treatment with neither rhizobia nor supplemental nitrogen would subject plants to severe nitrogen stress, producing physiological effects unrelated to our questions and obscuring the symbiotic interactions of interest. Thus, our design necessarily couples rhizobial inoculation with the absence of exogenous nitrogen and non-inoculated controls with nitrogen supplementation, reflecting conditions under which nodulation naturally occurs when other nitrogen sources are limited.

This approach has been employed in numerous previous studies of legume–rhizobium interactions. For example, Kontopoulou et al (2015), grew common bean plants in N-free solution after inoculation, while fertilized, uninoculated controls received full-strength nutrient solution. Similar protocols have been used in soybean (Barros De Carvalho *et al.*, 2013; Win *et al.*, 2023), common bean (Martín-Rodríguez *et al.*, 2018), faba bean (Allito *et al.*, 2021), and the invasive legume *Lupinus polyphyllus* (Ramula *et al.*, 2023) consistently applying inorganic N only to uninoculated controls to ensure successful nodulation in inoculated plants. This practice is grounded in the well-established principle that exogenous nitrogen can inhibit nodulation and nitrogen fixation (Streeter & Wong, 1988; Ohyama *et al.*, 2011).

In a previous study, we employed this nitrogen regime in combination with inoculation by commercially available rhizobial strains using the same soybean cultivar (Williams 82) and the same environmental chamber setup (Dean *et al.*, 2014). Results indicated that soybean plants maintained similar foliar nitrogen levels, with shoot biomass data suggesting that rhizobia inoculation compensated for reduced nitrogen input. Moreover, the presence of a second bacterial colonizer, *D. acidovorans*, further enhanced plant growth (Dean *et al.*, 2014; Pulido *et al.*, 2019). Thus, although this design necessarily imposes some degree of confounding between nitrogen supply and rhizobial inoculation, it represents the most appropriate and widely accepted approach for maintaining effective symbiosis while enabling meaningful comparisons between inoculated and uninoculated plants.

All plants were grown in an insect-free chamber maintained at 25°C (day) and 23°C (night) under a 16:8 light/dark cycle with 70% relative humidity. At approximately one week of age, half of the plants from each rhizobacteria treatment were manually rub-inoculated with BPMV using a 0.1 M potassium phosphate buffer solution and carborundum powder. The remaining plants received a mock inoculation with virus-free buffer. Virus presence was confirmed using a commercial immunological test (Agdia).

Method S3. Metabolite extraction and analysis

Leaf samples were extracted with 1.0 mL of 80% methanol containing 18 µl/mL umbelliferone as the internal standard for LC-MS analysis. The mixture was vortexed and incubated for 2 hours. After incubation, the samples were centrifuged at 4°C, and 0.5 mL of the supernatant was transferred to a new vial and stored at -20°C for subsequent LC-MS analysis. To the remaining mixture, 1.5 mL of chloroform containing 10 µg/mL docosanol (internal standard for the non-aqueous phase) was added. The samples were sonicated and vortexed between two incubation steps at 50°C. Afterward, the samples were allowed to sit at room temperature, followed by the addition of 1.5 mL of HPLC-grade water containing 25 µg/mL ribitol (internal standard for the aqueous phase). The samples were then vortexed and incubated at 50°C for 45 minutes.

Phase separation was achieved by centrifuging at 2900xg for 30 minutes at 4°C. One mL from each phase was collected into separate 2.0 mL autosampler vials. The aqueous phase was dried in a speed vacuum, while the non-aqueous phase was dried under nitrogen.

For further analysis, the non-aqueous phase was resuspended in chloroform and hydrolyzed with 1.25 M HCl in methanol, followed by a 4-hour incubation at 50°C. After incubation, the samples were dried under nitrogen, resuspended in 70 µL of pyridine, and derivatized with 30 µL of MSTFA + 1% TMCS (Sigma-Aldrich). Following 1-hour incubation at 50°C, the samples were transferred to glass inserts for GC-MS analysis. Dried aqueous extracts were resuspended in pyridine containing 15 mg/mL methoxyamine-HCl, vortexed, and sonicated between two incubation steps at 50°C. Aqueous metabolites were then derivatized with 50 µL of MSTFA + 1% TMCS for 1 hour at 50°C before transfer to glass inserts.

Both aqueous and non-aqueous metabolites were analyzed using an Agilent 7890 GC coupled to a 5975 MSD. Compounds were injected at 230°C and separated on an HP-5MS capillary column (30 m × 0.25 mm ID × 0.25 µm film thickness; Agilent) under the following temperature program: initial hold at 70°C for 5 minutes, followed by a ramp of 5°C/min to a final temperature of 315°C, held for 12 minutes. One microliter of each derivatized aqueous sample was injected using a 2:1 split ratio, while

0.3 µL of each derivatized non-aqueous sample was injected in splitless mode. Helium was used as the carrier gas at a constant flow of 1.0 mL/min. The MS was operated in electron impact mode (70 eV) with the following settings: Transfer line: 250°C (polar phase), 230°C (non-polar phase); Source: 230°C; Quadrupole: 150°C; Mass scan range: 50–650 amu. Deconvolution algorithms were applied to the total ion chromatograms (TICs) using MassHunter Workstation software (B.06.00; Agilent Technologies), and compounds were identified by comparison with the NIST14 spectral library. Quantification was performed relative to internal standards (25 µg/mL ribitol for the aqueous phase and 10 µg/mL docosanol for the non-aqueous phase).

For secondary metabolite analysis, 5 µL of methanol extract was separated by reverse-phase HPLC using a Prominence 20 UFLCXR system (Shimadzu, Columbia, MD) with a Waters BEH C18 column (100 mm x 2.1 mm, 1.7 µm particle size) maintained at 55°C. Separation was achieved with a 20-minute aqueous acetonitrile gradient at a flow rate of 250 µL/min. Solvent A was HPLC-grade water with 0.1% formic acid, and Solvent B was HPLC-grade acetonitrile with 0.1% formic acid. The initial condition of 97% A and 3% B was increased to 45% B at 10 minutes, 75% B at 12 minutes, held at 75% B until 17.5 minutes, and then returned to initial conditions.

The eluate was analyzed on a 5600 TripleTOF using a Duospray™ ion source (AB Sciex, Framingham, MA). The capillary voltage was set at 5.5 kV in positive ion mode and 4.5 kV in negative ion mode, with a declustering potential of 80 V. The mass spectrometer operated in Information Dependent Acquisition (IDA) mode with a 100 ms survey scan from 100 to 1200 m/z, and up to 20 MS/MS product ion scans (100 ms each) per duty cycle with a collision energy of 50 V and a 20 V spread. LC-MS raw files were processed using XCMS online for retention time alignment, automatic integration, and feature detection. Peak intensities were exported for multivariate analysis, and the METLIN database was used to identify key LC-MS features based on MS signatures and tandem mass spectrometry (MS/MS) spectra.

Data integration was performed after normalizing individual databases from each phase, including secondary metabolites (LC-MS phase) and phytohormones, by their respective phase-specific internal standards and initial sample weights. Compound names were coded according to the IDs listed in Supplementary Table S9.

Method S4 Transcriptomics and metabolomics data integration

We used Discriminant Analysis of Principal Components (DAPC) (Jombart *et al.*, 2010) to explore data structure and assess group separation in both transcriptomics and metabolomics datasets, particularly between the rhizobacteria and virus treatments.

To identify the important genes and metabolites that change according to the treatment, we used various feature selection techniques. For metabolites, we employed a recursive feature elimination approach (RFE) and multiple linear models (limma), while for the gene matrix, we used RFE, differential expression analysis (DESeq2), and Weighted correlation network analysis (WGCNA). The end result of these analyses is a list of signature genes and metabolites that were combined to obtain an Integrated Transcriptome-Metabolome Signature (**ITMS**).

We implemented RFE in the caret R package (Kuhn, 2008) using a 5-fold cross-validation resampling method and three different machine learning algorithms: random forest (rf, ntrees = 5000), bagged adaptive boosting (adabag, ntrees = 5000), and support vector machines (svm). This technique helped us identify the most important features in the gene and metabolite sets by

recursively removing features from the dataset and retraining the model on the remaining features until we obtained a model with the highest possible accuracy. At each step, we evaluated the importance of the remaining features and eliminated the least important feature.

Next, we used the limma R package (Ritchie *et al.*, 2015) to identify significant differences (p-value < 0.05) in metabolite abundance between two given treatments. We fitted a linear model to the metabolite abundance data and used empirical Bayes statistics to calculate the significance of the differences in metabolite abundance between the groups.

The differential gene expression analysis between two given treatments was achieved with DESeq2 (Love *et al.*, 2014) using a threshold of an adjusted p-value < 0.01. DESeq2 identified the genes that were differentially expressed between two or more experimental conditions by fitting a negative binomial model to the read count data and using empirical Bayes shrinkage to estimate the variance of the gene expression changes.

In addition, we used weighted gene co-expression network analysis (WGCNA) (Langfelder & Horvath, 2008) to identify co-expressed gene modules and important genes by detecting significant differences of genes across treatment groups within each module. We used a soft threshold (power = 12) based on the approximate scale-free topology and constructed an unsigned gene network to identify modules of strongly correlated genes using Pearson correlation. We did not pre-filter the gene expression data by differential expression, which allowed us to reveal gene sets that may be highly correlated with specific treatments but do not pass the differential expression threshold (Sánchez-Baizán *et al.*, 2022).

Following the feature selection techniques described above (RFE, DESeq2, limma, and WGCNA), we combined the list of signature genes and signature metabolites into an Integrated Transcriptome-Metabolome Signature (**ITMS**) for downstream network and integrative analysis. To identify metabolic processes affected by the treatments, we performed gene/metabolite set enrichment analysis using the gage Bioconductor package (Luo *et al.*, 2009) and selected significantly up- and downregulated pathways to map the genes and metabolites in the Metabolism and Environmental Information Processing sections of the KEGG PATHWAY database (http://www.genome.jp/kegg/pathway.html). We generated pathway maps showing differentially expressed genes and metabolites for each Metabolism pathway using the “pathview” function (Luo & Brouwer, 2013).

To simplify the comparison of treatments and account for the similarities in gene expression and metabolite abundance patterns observed among the rhizobacteria treatments, we created two new categories: one for all uninfected rhizobacteria treatments (Bj, Bj+Da, and Da) and another for all BPMV-infected rhizobacteria treatments. These categories are referred to as "all Bacteria-uninfected" and "all Bacteria-infected", respectively. This allowed us to compare each combined group against the control-uninfected treatment and identify significant differences between the two groups in the various analyses performed.

TABLES

Table S1. Factorial design used to evaluate the main effect of rhizobacterial inoculation and BPMV-infection in soybean plants.

		**Rhizobacteria treatments**			
		**Control**	***B. japonicum***	***D. acidovorans***	***B. japonicum* + *D. acidovorans***
**BPMV treatments**	**infected**	Control-BPMV	Bj-BPMV	Da-BPMV	Bj+Da-BPMV
	**uninfected**	Control-uninfected	Bj-uninfected	Da-uninfected	Bj+Da-uninfected

Table S2. Results of the exact binomial test for all pairwise dual-choice foraging contrasts shown in Figure 2.

**Contrast**	**Proportion (95% CI)**	**p_value**
Bj vs Bj+Da, uninfected	0.479 [0.466, 0.491]	<0.001
Bj vs Bj+Da, infected	0.481 [0.475, 0.487]	<0.001
control vs Bj, uninfected	0.327 [0.319, 0.335]	<0.001
control vs Bj, infected	0.942 [0.938, 0.945]	<0.001
control vs Da, uninfected	0.996 [0.986, 1.000]	<0.001
control vs Da, infected	0.484 [0.479, 0.489]	<0.001
control vs Bj+Da, uninfected	0.387 [0.380, 0.394]	<0.001
control vs Bj+Da, infected	0.44 [0.436, 0.444]	<0.001
Bj+Da uninfected vs Bj+Da infected	0.912 [0.910, 0.913]	<0.001
control uninfected vs control infected	0.226 [0.217, 0.236]	<0.001

Table S3. Results of the analysis of variance (ANOVA) performed on adult feeding damage across rhizobacterial and virus treatments (Figure 3). The model tested whether feeding percentages differed among treatments, and the table reports the degrees of freedom, sums of squares, mean squares, F-values, and corresponding p-values for each comparison. All statistical assumptions were evaluated prior to interpreting the ANOVA results (see Methods).

Contrast	Degrees of Freedom	Sum of Squares	Mean Square	F statistic	p_value
Bj vs Bj+Da, uninfected	1 22	115.85	115.85	1.90	0.18
**control vs Bj, uninfected**	**1 22**	**333.76**	**333.76**	**7.15**	**0.01**
control vs Da, uninfected	1 22	14.88	14.88	0.25	0.63
**control vs Bj+Da, uninfected**	**1 22**	**604.81**	**604.81**	**7.22**	**0.01**
Bj vs Bj+Da, infected	1 16	65.47	65.47	1.19	0.29
**control vs Bj, infected**	**1 12**	**204.14**	**204.14**	**6.42**	**0.03**
control vs Da, infected	1 22	0.35	0.35	0.00	0.95
**control vs Bj+Da, infected**	**1 22**	**3,212.29**	**3,212.29**	**20.27**	**0.00**
**Bj+Da uninfected vs Bj+Da infected**	**1 38**	**438.18**	**438.18**	**5.17**	**0.03**
**control uninfected vs control infected**	**1 22**	**917.98**	**917.98**	**13.60**	**0.00**

Table S4. Results of the analysis of variance (ANOVA) (Section A) and post-hoc comparisons (Section B) performed on larvae weight across rhizobacterial and virus treatments (Figure 4). All statistical assumptions were evaluated prior to interpreting the ANOVA results (see Methods).

Section A. ANOVA summary

Term	Degrees of Freedom	Sum of Squares	Mean Square	F statistic	p_value
**rhi**	**3**	**0.000081**	**0.000027**	**5.354644**	**0.003012**
**BPMV**	**1**	**0.000068**	**0.000068**	**13.487345**	**<0.001**
rhi:BPMV	3	0.000002	0.000001	0.120152	0.947792
Residuals	46	0.000231	0.000005		

Section B. Tukey HSD pairwise comparisons (rhizobacteria)

Contrast	Difference	Lower CI	Upper CI	Adjusted p-value
Da vs control	0.00005	-0.00279	0.00288	1.00
Bj vs control	0.00227	-0.00005	0.00458	0.06
**Bj+Da vs control**	**0.00291**	**0.00051**	**0.00532**	**0.01**

Section A. ANOVA summary					
**Term**	**Degrees of Freedom**	**Sum of Squares**	**Mean Square**	**F statistic**	**p_value**
Bj vs Da	0.00222	-0.00028	0.00472	0.10	
**Bj+Da vs Da**	**0.00287**	**0.00028**	**0.00545**	**0.02**	
Bj+Da vs Bj	0.00065	-0.00136	0.00265	0.83	

Table S5. Summary of ANOVA test for leaf toughness across rhizobacteria treatments (Figure 5A).

Term	Degrees of Freedom	Sum of Squares	Mean Square	F statistic	p_value
rhi	3	0.450280	0.150093	1.283566	0.293141
virus	1	0.173816	0.173816	1.486436	0.229911
rhi:virus	3	0.050478	0.016826	0.143894	0.932990
Residuals	40	4.677382	0.116935		

Table S6. Summary of ANOVA test for leaf biomass across rhizobacteria treatments (Figure 5B).

Term	Degrees of Freedom	Sum of Squares	Mean Square	F statistic	p_value
trt	3	0.643929	0.214643	1.788566	0.149795
**BPMV**	**1**	**19.955780**	**19.955780**	**166.286413**	**<0.001**
trt:BPMV	3	0.775903	0.258634	2.155132	0.093814
Residuals	254	30.482154	0.120008		

Table S7. Summary of ANOVA test for nodule biomass across virus treatments (Figure 5C).

Term	Degrees of Freedom	Sum of Squares	Mean Square	F statistic	p_value
trt	1	0.000003	0.000003	0.020167	0.887343
**virus**	**1**	**0.003934**	**0.003934**	**28.784898**	**<0.001**
trt:virus	1	0.000024	0.000024	0.174807	0.676728
Residuals	105	0.014351	0.000137		

Table S8. Summary of regression tests between Nodule weight ~ shoot biomass across virus treatments (Figure 5D).

Model	Slope	F_statistic	DF	R_squared	p_value
Bj-uninfected	-0.019	0.97	1, 29	0.03	0.33400
**Bj-infected**	**0.024**	**8.56**	**1, 20**	**0.30**	**0.00837**
**Bj+Da-uninfected**	**0.085**	**9.39**	**1, 21**	**0.31**	**0.00588**
Bj+Da-infected	0.004	0.69	1, 6	0.10	0.43700

Table S9. Compound IDs with KEGG and HMDB codes when available.

ID	Compound Name	KEGG	HMDB	Class
N3	glycolic acid	C00160	HMDB00115	carboxylic acid
N6	benzoic Acid	C00180	HMDB01870	carboxylic acid
N11	glycerol	C00116	HMDB00131	sugar alcohol
N13	maltol	C11918	HMDB30776	pyranone
N22	beta-L-Galactopyranoside			carbohydrate
N23	L-rhamnose	C00507	HMDB00849	monosaccharide
N26	methyl xylopyranoside			carbohydrate
N29	threonolactone		HMDB00940	carboxylic acid
N31	phosphoric acid	C00009	HMDB02142	inorganic acid
N33	phosphoric acid	C00009	HMDB02142	inorganic acid
N35	phosphoric acid	C00009	HMDB02142	inorganic acid
N37	beta-D-Galactofuranoside			carbohydrate
N39	methyl galactoside		HMDB29965	carbohydrate
N42	3,7,11,15-Tetramethyl-2-hexadecen-1-ol			alcohol
N43	citronellyl valerate		HMDB37229	fatty acid
N45	D-Mannose	C00159	HMDB00169	monosaccharide
N47	hexadecanoic acid, methyl ester	C16995	HMDB61859	fatty acid
N49	iduronic acid	C06472	HMDB02704	carbohydrate

N52	11,14-Octadecadienoic acid, methyl ester	C01595	HMDB00673	fatty acid
N57	methyl stearate		HMDB34154	fatty acid
N59	myo-Inositol	C00137	HMDB00211	carbohydrate
N61	methyl 2-hydroxyhexadecanoate			fatty acid
N65	eicosanoic acid	C06425	HMDB02212	fatty acid
N68	glyceryl-glycoside			carbohydrate
N70	D-Galactopyranoside	C03619		glucosinolate
N75	docosanoic acid	C08281	HMDB00944	fatty acid
N79	methyl 2-hydroxydocosanoate			fatty acid
N80	tetracosanoic acid, methyl ester	C08320	HMDB02003	fatty acid
N81	maltose	C00208	HMDB00163	carbohydrate
N90	1-hexacosanol	C08381		fatty alcohol
N95	alpha-D-lactose	C00243	HMDB00186	carbohydrate
N103	beta-sitosterol	C01753	HMDB00852	lipid
N104	beta-amyrin	C08616	HMDB36658	terpenoid
P2	propanoic acid	C00163	HMDB00237	carboxylic acid
P3	pinacol			alcohol
P7	2-pentanol	C16834	HMDB31599	alcohol
P8	acetoin	C00466	HMDB03243	Acyloins
P9	pentenoic acid	C00803	HMDB00892	carboxylic acid

P12	3-furoic acid	C01546	HMDB00444	carboxylic acid
P13	oxalic acid	C00209	HMDB02329	carboxylic acid
P18	1-octanol	C00756	HMDB01183	fatty alcohol
P19	propanedioic acid	C00383	HMDB00691	carboxylic acid
P22	urea	C00086	HMDB00294	organic acid
P26	1-phenyl-1,2-ethanediol			alcohol
P27	dopamine	C03758	HMDB00073	amine
P32	niacin	C00253	HMDB01488	carboxylic acid
P34	2-butenoic acid	C01771	HMDB10720	fatty acid
P35	glycine	C00037	HMDB00123	amino acid
P36	butanedioic acid	C00042	HMDB00254	carboxylic acid
P38	glyceric acid	C00258	HMDB00139	carbohydrate
P39	2-butenedioic acid	C01384	HMDB00176	carboxylic acid
P41	serine	C00065	HMDB00187	carboxylic acid
P42	erythrono-1,4-lactone		HMDB00349	carbohydrate
P46	L-threonine	C00188	HMDB00167	amino acid
P47	hexanoic acid	C01585	HMDB00535	fatty acid
P49	beta-alanine	C00099	HMDB00056	amino acid
P51	aspartic acid	C00049	HMDB00191	amino acid
P52	pyruvic acid	C00022	HMDB00243	carboxylic acid

P53	malic acid	C00711	HMDB00744	carboxylic acid
P55	arabino-hexos-2-ulose		HMDB29932	aldehyde
P57	L-5-oxoproline	C01879	HMDB00267	amino acid
P58	4-aminobutanoic acid	C00334	HMDB00112	amino acid
P60	propanetriol, 2-methyl-			alcohol
P61	L-threonic acid	C01620	HMDB00943	amino acid
P63	pentanedioic acid	C00489	HMDB00661	carboxylic acid
P64	xylose	C00181	HMDB00098	monosaccharide
P66	3-hydroxybenzoic acid	C00587	HMDB02466	carboxylic acid
P73	hexanedioic acid	C06104	HMDB00448	carboxylic acid
P75	d-ribose	C00121	HMDB00283	monosaccharide
P78	putrescine	C00134	HMDB01414	amine
P90	ribonic acid	C01685	HMDB00867	carbohydrate
P95	shikimic acid	C00493	HMDB03070	alcohol
P98	cadaverine	C01672	HMDB02322	amine
P100	citric acid	C00158	HMDB00094	carboxylic acid
P106	indolin-2-one	C12312	HMDB61918	indol
P110	D-pinitol	C03844	HMDB34219	alcohol
P113	D-fructose	C02336	HMDB00660	monosaccharide
P118	D-fructose	C02336	HMDB00660	monosaccharide

P120	levoglucosan		HMDB00640	monosaccharide
P123	D-talose	C06467		carbohydrate
P125	4-coumaric acid	C00811	HMDB02035	carboxylic acid
P127	D-allose	C01487	HMDB01151	monosaccharide
P129	D-mannitol	C00392	HMDB00765	carbohydrate
P130	D-glucitol	C00794	HMDB00247	carbohydrate
P138	D-gluconic acid	C00257	HMDB00625	carbohydrate
P140	palmitic acid	C00249	HMDB00220	fatty acid
P144	D-glucopyranoside	C00738	HMDB62170	monosaccharide
P145	galactaric acid	C00879	HMDB00639	carbohydrate
P148	ferulic acid	C01494	HMDB00954	carboxylic acid
P151	caffeic acid	C01481	HMDB01964	carboxylic acid
P154	sedoheptulose	C02076	HMDB03219	monosaccharide
P155	galactose	C00984	HMDB00143	monosaccharide
P171	D-glucuronic acid	C00191	HMDB00127	carboxylic acid
P172	2-hydroxymandelic acid, ethyl ester, di-TMS	C11527	HMDB00822	carboxylic acid
P180	mannobiose	C20861	HMDB29933	carbohydrate
P192	sucrose	C00089	HMDB00258	carbohydrate
P198	D-xylopyranose	C00181	HMDB00098	monosaccharide
P203	D-trehalose	C01083	HMDB00975	carbohydrate

P207	D-myo-Inisitol	C00137	HMDB00211	alcohol
P227	dihydroxymalonic acid	C00830	HMDB31522	carboxylic acid
P235	cellobiose	C06422	HMDB00055	carbohydrate
P237	D-glucose	C00031	HMDB00122	monosaccharide
S2	isoquercitrin	C05623	HMDB37362	flavonoid
S3	L-beta-aspartyl-L-phenylalanine		HMDB11167	amino acid
S5	eriocitrin	C09732	HMDB05811	flavonoid
S6	kaempferol	C05903	HMDB05801	flavonoid
S7	formononetin 7-O-rutinoside	C00858	HMDB05808	flavonoid
S8	pelargonidin 3-gentiotrioside	C05904	HMDB03263	flavonoid
S9	rubrofusarin	C09047	HMDB34569	glycoside
S10	rutin	C05625	HMDB03249	flavonoid
S12	cyanidin 3-rhamnoside 5-glucoside		HMDB37993	flavonoid
S13	cyanidin 3-glucogalactoside		HMDB31468	flavonoid
S14	pelargonidin 3-galactoside-5-glucoside		HMDB41170	flavonoid
S15	biotin-XX hydrazide	C00120	HMDB00030	amine
S17	caffeic acid 3-O-glucuronide	C01481	HMDB41705	carboxylic acid
S18	pelargonidin 3-sophoroside 5-glucoside		HMDB33687	flavonoid
S20	luteolin	C01514	HMDB05800	flavonoid
S22	soyasaponin II	C12081	HMDB34650	lipid

S23	pratensin A			flavonoid
S29	alpha-calendic acid		HMDB30962	lipid
S32	peonidin 3-lathyroside		HMDB41171	flavonoid
S33	ribose-1-arsenate		HMDB12285	monosaccharide
S35	carnosifloside III	C08791		terpenoid
S36	unkown33			peptide
S38	1,6-digalloyl-beta-D-glucopyranose		HMDB39179	flavonoid
S39	petunidin 3-rhamnoside 5-glucoside		HMDB38099	flavonoid
S40	chlortetracycline	C06571	HMDB14401	tetracycline
S43	kuwanon L		HMDB30121	flavonoid
S45	peonidin 3-galactoside-5-glucoside		HMDB29210	flavonoid
S46	petunidin 3-galactoside		HMDB38093	flavonoid
S50	soyasaponin A3		HMDB38608	flavonoid
S52	spinacetin 3-gentiobioside		HMDB37469	flavonoid
S53	Lys Lys Thr Trp			peptide
S59	soyasaponin I	C08983	HMDB34649	flavonoid
S65	PG(16:1(9Z)/0:0)		HMDB10571	lipid
S73	irilone 4'-O-glucoside		HMDB33818	flavonoid
S78	2-O-caffeoylhydroxycitric acid		HMDB40572	carboxylic acid
S82	soyasaponin bg		HMDB38721	flavonoid

S87	2-O-caffeoylglucarate	C03062	HMDB40572	carboxylic acid
S89	sulindac sulfone		HMDB60620	carboxylic acid
S90	D-ribulose	C00309	HMDB00621	monosaccharide
S91	Lys Leu Glu Ala Thr			peptide
S94	peonidin 3-rutinoside-5-glucoside			flavonoid
S95	malvidin 3-sophoroside 5-glucoside		HMDB38032	flavonoid
S97	phosphatidylinositol	C01194	HMDB09807	lipid
S99	Met Arg Arg Val			peptide
S100	araliasaponin III		HMDB36477	glycoside
S120	indomethacin N-octyl amide			amide
cisOPDA	12-oxo-phytodienoic acid	C01226		phytohormone
ABA	abscisic acid	C15970	HMDB36093	phytohormone
S86	gamma-linolenic acid	C06426	HMDB03073	phytohormone
cisJA	jasmonic acid	C08491	HMDB32797	phytohormone
LA	linoleic acid	C01595	HMDB00673	phytohormone
N54	linolenic acid	C06427	HMDB01388	phytohormone
OA	oleic acid	C00712	HMDB00207	phytohormone
SA	salicylic acid	C00805	HMDB01895	phytohormone
S19	tuberonic acid glucoside	C08558		phytohormone
N5	compound_4			unknown

N16	compound_7			unknown
N24	compound_24			unknown
N25	compound_27			unknown
N27	compound_32			unknown
N32	Unknown N32			unknown
N40	Unknown N40			unknown
N41	compound_41			unknown
N44	compound_43			unknown
N50	compound_61			unknown
N53	compound_58			unknown
N56	compound_46			unknown
N58	compound_48			unknown
N62	compound_74			unknown
N63	compound_76			unknown
N64	compound_77			unknown
N66	compound_83			unknown
N67	compound_85			unknown
N69	compound_81			unknown
N71	compound_87			unknown
N72	compound_53			unknown

N74	compound_89			unknown
N76	compound_93			unknown
N77	compound_94			unknown
N78	compound_95			unknown
N83	compound_100			unknown
N84	compound_102			unknown
N85	Unknown N85			unknown
N86	compound_106			unknown
N87	compound_91			unknown
N89	compound_109			unknown
N93	compound_103			unknown
N96	compound_118			unknown
N98	compound_119			unknown
N99	compound_108			unknown
N105	compound_125			unknown
P1	compound_1			unknown
P10	compound_6			unknown
P14	compound_7			unknown
P20	compound_16			unknown
P23	compound_18			unknown

P37	compound_32			unknown
P40	compound_35			unknown
P44	compound_30			unknown
P56	compound_39			unknown
P59	compound_42			unknown
P65	compound_49			unknown
P67	compound_46			unknown
P69	compound_47			unknown
P70	compound_52			unknown
P71	compound_53			unknown
P76	compound_71			unknown
P77	compound_60			unknown
P79	compound_57			unknown
P88	unknown P88			unknown
P93	compound_75			unknown
P102	compound_69			unknown
P107	etoxeridine			unknown
P111	compound_84			unknown
P112	compound_85			unknown
P131	compound_101			unknown

P135	compound_116			unknown
P136	compound_108			unknown
P139	compound_120			unknown
P142	compound_114			unknown
P147	compound_119			unknown
P149	compound_129			unknown
P152	compound_123			unknown
P153	compound_103			unknown
P156	compound_128			unknown
P157	compound_137			unknown
P161	compound_141			unknown
P162	compound_133			unknown
P163	compound_110			unknown
P174	compound_144			unknown
P175	compound_117			unknown
P176	unknown N176			unknown
P177	compound_122			unknown
P178	compound_150			unknown
P181	compound_153			unknown
P182	compound_125			unknown

P183	compound_126			unknown
P184	compound_154			unknown
P185	unknown P185			unknown
P186	unknown P186			unknown
P187	compound_159			unknown
P193	compound_165			unknown
P194	compound_166			unknown
P196	compound_168			unknown
P197	compound_135			unknown
P199	compound_170			unknown
P204	compound_176			unknown
P205	compound_178			unknown
P206	compound_179			unknown
P211	compound_187			unknown
P219	compound_152			unknown
P226	compound_209			unknown
P228	unknown P228			unknown
P236	compound_221			unknown
P238	unknown P238			unknown
P239	compound_225			unknown

P240	compound_174			unknown
P241	compound_175			unknown
P243	compound_227			unknown
P244	compound_228			unknown
P246	compound_230			unknown
S24	unknown S24			unknown
S25	unknown 74			unknown
S26	unknown 97			unknown
S28	unknown 96			unknown
S34	unknown 71			unknown
S42	unknown 77			unknown
S48	unknown 78			unknown
S56	unknown S56			unknown
S57	unknown 84			unknown
S62	unknown 76			unknown
S63	unknown 93			unknown
S69	unknown 80			unknown
S72	unknown 46			unknown
S74	unknown 70			unknown
S76	unknown 85			unknown

S77	unknown 64			unknown
S79	unknown 48			unknown
S88	unknown 79			unknown
S96	unknown 67			unknown
S98	unknown 60			unknown
S101	unknown 55			unknown
S102	unknown 69			unknown
S103	alhagidin			unknown
S105	unknown 81			unknown
S106	unknown 57			unknown
S108	unknown 95			unknown
S109	unknown 61			unknown
S110	unknown 66			unknown
S112	unknown S112			unknown
S117	unknown 62			unknown
S124	unknown 68			unknown
S126	unknown 63			unknown
S127	unknown S127			unknown

Table S10. Comparisons tested in the behavioral assays, and statistical analysis for signature gene and compound selection. DEG = differentially expressed genes by deseq2; DAM = differentially accumulated metabolites by limma. Bj = *Bradyrhizobium japonicum*, Da = *Delftia acidovorans*.

id	Virus treatment	Choice 1	Choice 2	Adult beetles dual-choice test	DEG	DAM	WGCNA
a.uninfected	Uninfected	Bj	Control	x	x	x	
b.uninfected	Uninfected	Bj+Da	Control	x	x	x	
c.uninfected	Uninfected	Da	Control	x	x	x	
d.uninfected	Uninfected	Bj+Da	Bj	x	x	x	
a.BPMV	Infected	Bj	Control	x	x	x	
b.BPMV	Infected	Bj+Da	Control	x	x	x	
c.BPMV	Infected	Da	Control	x	x	x	
d.BPMV	Infected	Bj+Da	Bj	x	x	x	
a.mixed	Mixed	Control infected	control uninfected		x	x	
b.mixed	Mixed	Bj+Da infected	Bj+Da uninfected		x	x	
c.mixed	Mixed	Bj infected	Bj uninfected		x	x	
d.mixed	Mixed	Bj infected	control uninfected		x	x	
e.mixed	Mixed	Bj+Da infected	control uninfected	x	x	x	
f.mixed	Mixed	Da infected	control uninfected		x	x	
g.mixed	Mixed	(Bj, Bj+Da, Da) uninfected	control uninfected		x	x	x
h.mixed	Mixed	(Bj, Bj+Da, Da) infected	control uninfected		x	x	x
i.mixed	Mixed	(Bj, Bj+Da, Da) infected	(Bj, Bj+Da, Da) BPMV uninfected		x	x	x

Table S11. Top six genes for the modules of co-expression (MEs) shown in Fig. 9

Module	Top six genes
6	uncharacterized LOC100777483<br>uncharacterized LOC100805057<br>chlorophyllide a oxygenase, chloroplastic<br>geraniol 8-hydroxylase<br>chlorophyllide a oxygenase, chloroplastic<br>uncharacterized LOC100812363
1	glutamate decarboxylase<br>uncharacterized LOC100500099<br>2-methyl-6-phytyl-1,4-hydroquinone methyltransferase, chloroplastic<br>protein CELLULOSE SYNTHASE INTERACTIVE 1<br>uncharacterized LOC100798854<br>nucleobase-ascorbate transporter 6
7	serine carboxypeptidase-like<br>resistance protein MG13<br>sodium/calcium exchanger NCL2<br>LEAF RUST 10 DISEASE-RESISTANCE LOCUS RECEPTOR-LIKE PROTEIN KINASE-like 2.1<br>WRKY DNA -binding domain-containing protein<br>WRKY transcription factor 17

FIGURES

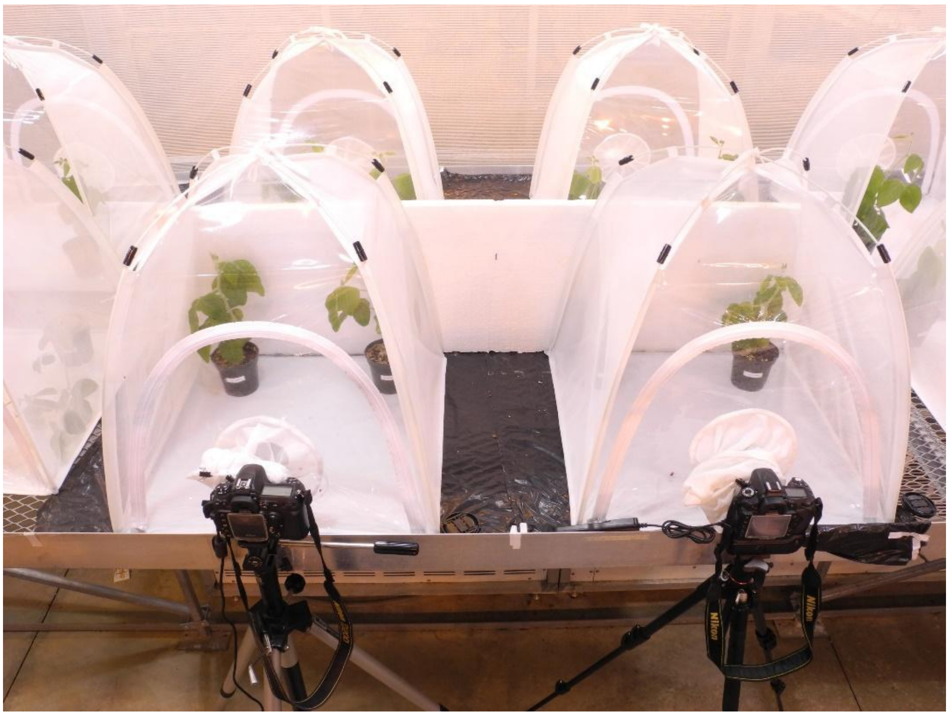

Figure S1. Feeding and foraging preferences of adult beetles. The activity of five adult beetles was recorded inside the tent. Two plants from different treatments were placed inside a fine-mesh cage.

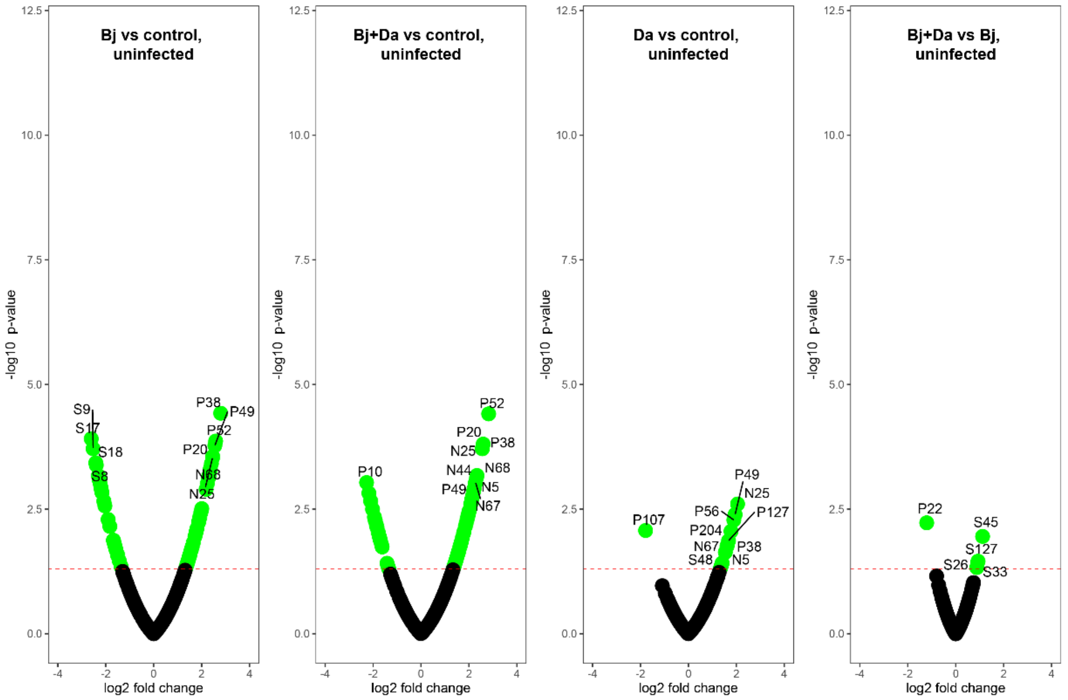

Figure S2. Rhizobacteria-induced metabolite changes in uninfected soybean plants. The volcano plot displays the log2 fold change on the x-axis and the negative log10 of the p-value on the y-axis. Significant metabolites (p-value < 0.05) are highlighted as green dots, while non-significant metabolites (p-value > 0.05) are shown as black dots. The top 10 most significant metabolites are labeled in the plot, with compound IDs listed in Table S9.

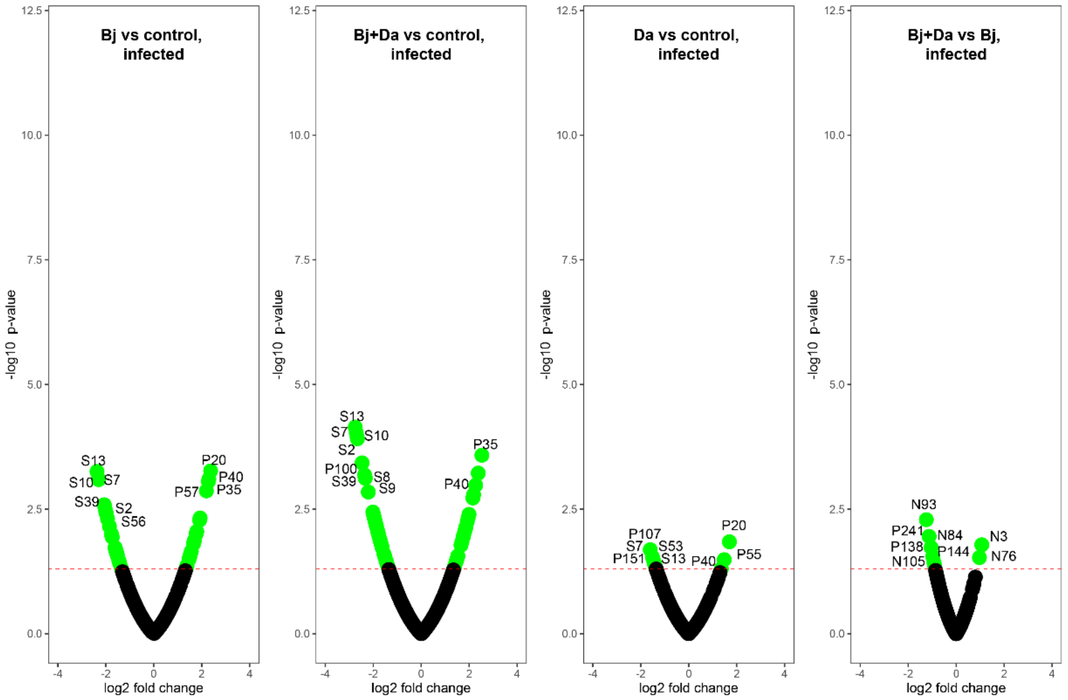

Figure S3.

Rhizobacteria-induced metabolite changes in BPMV-infected soybean plants. The volcano plot displays the log2 fold change on the x-axis and the negative log10 of the p-value on the y-axis. Significant metabolites (p-value < 0.05) are highlighted as green dots, while non-significant metabolites (p-value > 0.05) are shown as black dots. The top 10 most significant metabolites are labeled in the plot, with compound IDs listed in Table S9.

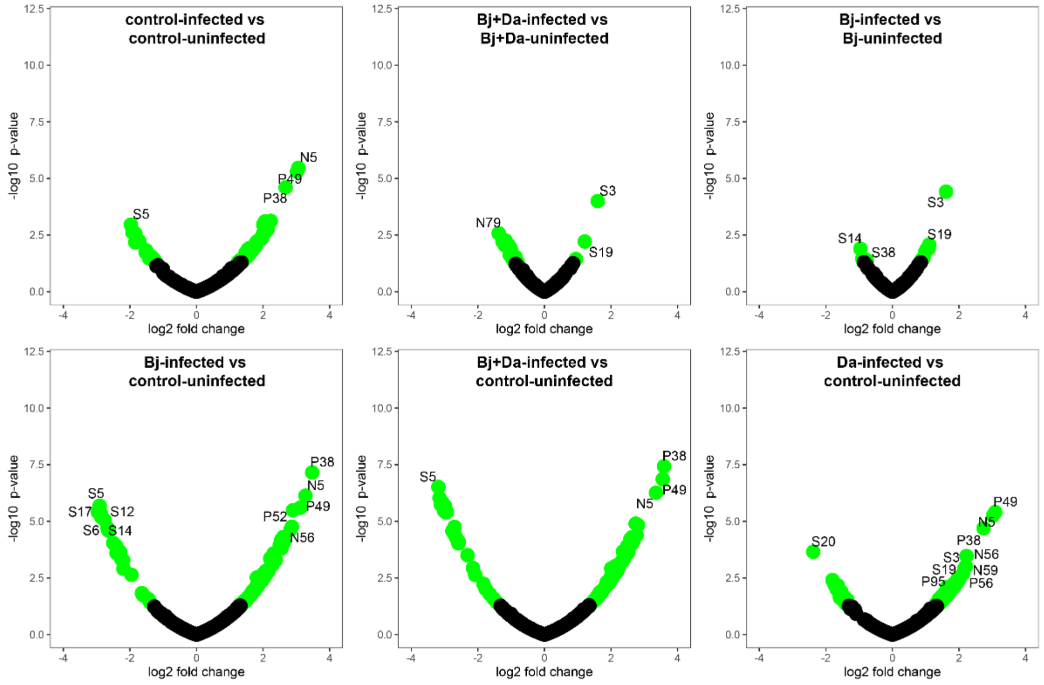

Figure S4. BPMV-induced metabolite changes. The volcano plots display the log2 fold change on the x-axis and the negative log10 of the p-value on the y-axis. The top panels show BPMV-induced metabolite changes in rhizobacteria-free and rhizobacteria-inoculated plants, while the bottom panels depict metabolite changes upon mixed treatments. Significant metabolites (p-value < 0.05) are highlighted as green dots, and non-significant metabolites (p-value > 0.05) are shown as black dots. The top 10 most significant metabolites are labeled in the plot, with compound IDs listed in Table S9.

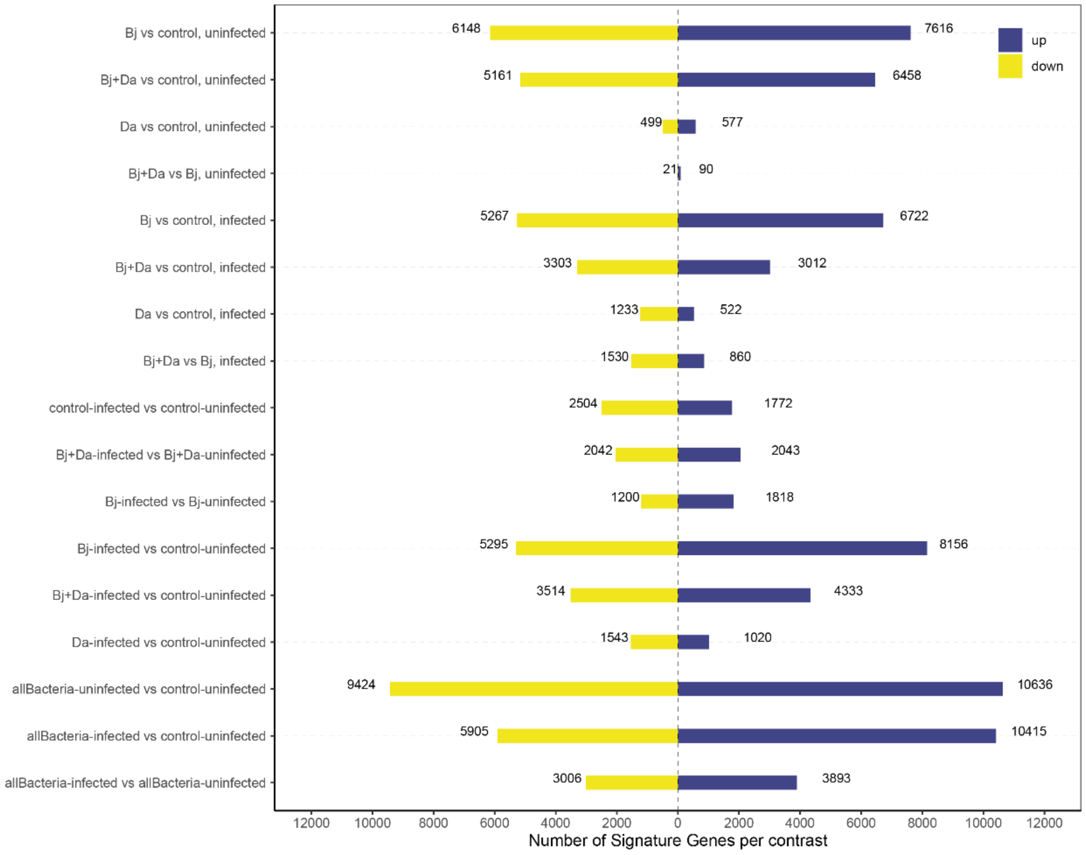

Figure S5. Number of Signature Genes per treatment. The horizontal bar graph displays the number of signature genes identified in various pairwise comparisons of rhizobacteria and BPMV treatments. The x-axis represents the number of signature genes, while each row on the y-axis represents a different pairwise comparison. Bars indicate the number of upregulated genes (in yellow) and downregulated genes (in blue). The **integrated Gene Signature (IGS)** was identified through deseq analysis, recursive feature elimination, and weighted gene co-expression network analysis.

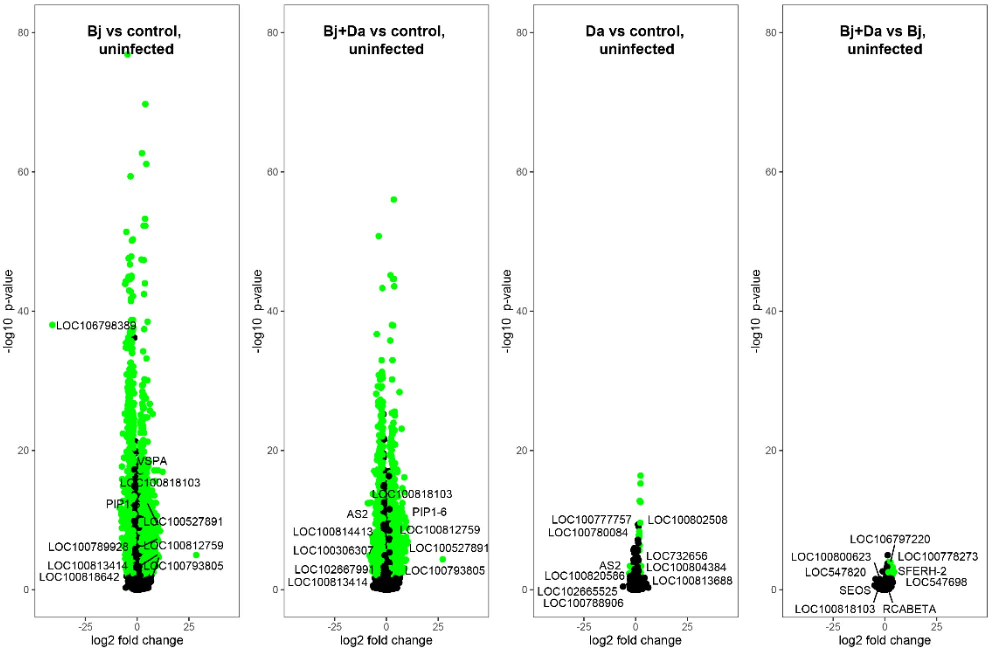

Figure S6. Rhizobacteria-induced signature gene profiles of uninfected soybean plants. The volcano plots display the log2 fold change on the x-axis and the negative log10 of the p-value on the y-axis. Names of Signature Genes identified through deseq analysis, recursive feature elimination, and WGCNA are shown. Significant genes (log2 fold change > 1.5 and adjusted p-value < 0.01) are highlighted as green dots, while non-significant genes are shown as black dots. A. Bj vs control. B. Bj+Da vs control. C. Da vs control. D. Bj+Da vs Bj.

Figure S7.

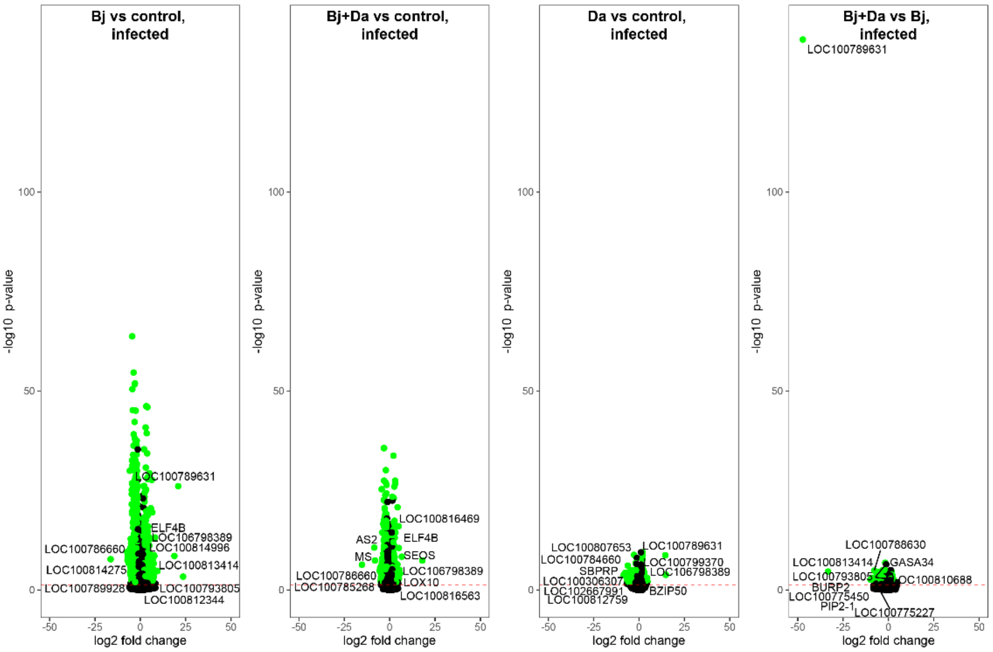

Rhizobacteria-induced gene profiles in BPMV-infected soybean plants. The volcano plots display the log2 fold change on the x-axis and the negative log10 of the p-value on the y-axis. Names of Signature Genes identified through deseq analysis, recursive feature elimination, and WGCNA are shown. Significant genes (log2 fold change > 1.5 and adjusted p-value < 0.01) are highlighted as green dots, while non-significant genes are shown as black dots. A. Bj vs control. B. Bj+Da vs control. C. Da vs control. D. Bj+Da vs Bj.

Figure S8.

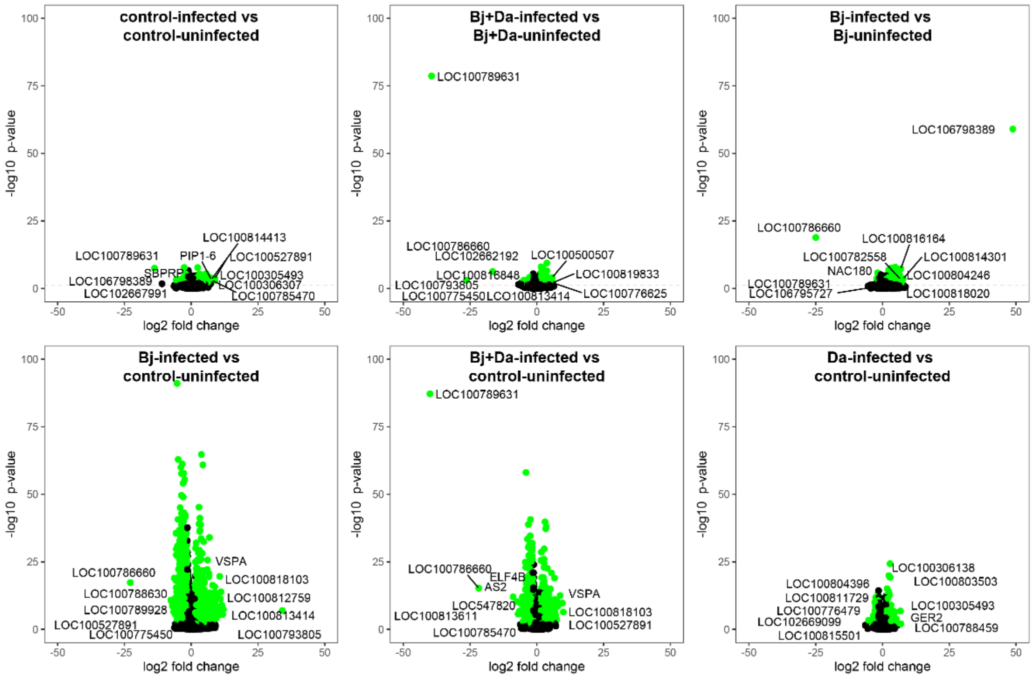

Soybean gene profiles upon rhizobacteria inoculation and BPMV infection. The volcano plots display the log2 fold change on the x-axis and the negative log10 of the p-value on the y-axis. Names of Signature Genes identified through deseq analysis, recursive feature elimination, and WGCNA are shown. Significant genes (log2 fold change > 1.5 and adjusted p-value < 0.01) are highlighted as green dots, while non-significant genes are shown as black dots. A. Bj vs control. B. Bj+Da vs control. C. Da vs control. D. Bj+Da vs Bj.

VIDEO S1

Example of a dual-choice assay demonstrating the foraging behavior of adult beetles. Two plants from different treatments were placed inside a fine-mesh cage, and five beetles were released to forage freely for 24 hours. This setup illustrates beetle preference and feeding behavior under varying experimental conditions.

DATASETS

Dataset S1. "*Supplementary_file_S5.1_results_limma_20230413.xlsx*"

This Excel file contains ranked compounds resulting from the linear model fit using the R function *limma::topTable()* for multiple contrasts. The file includes several sheets, with each sheet representing the results of the *limma* analysis for the specific pair-wise comparisons shown in Figure 7 and Figures S2–S4.

Key information in the file includes:

Compound Identification: The compound ID, classification, and additional identifiers, such as KEGG and Human Metabolome Database (HMDB) IDs, where available.

Statistical Results: The log fold change (logFC) and p-values from the t-test for each comparison.

Dataset S2. *"Supplementary_file_S5.2_results_deseq3_20230413.xlsx"*

Summary of differential expression analysis results from DESeq2.

This file contains multiple sheets, with each sheet presenting results for the specific pair-wise comparisons shown in Figure 8 and Figures S6–S8. Each sheet includes a table with the following columns:

- **id**: The identifier for each gene or feature in the dataset.
- **Entrez_id**: the entrez id for each gene.
- **symbol**: the symbol for each gene.
- **genename**: the corresponding gene name when available.
- **baseMean**: The mean of normalized counts across all samples for each gene or feature.
- **log2FoldChange**: The log2 fold change in expression levels between two conditions (treatment1 vs treatment2). Positive values indicate upregulation in treatment1, while negative values indicate downregulation.
- **pvalue**: The p-value associated with the Wald statistic, indicating the likelihood of the observed data under the null hypothesis of no differential expression.
- **padj**: The adjusted p-value, accounting for multiple testing correction (e.g., Benjamini-Hochberg). This value represents the probability of false positives, with smaller values indicating more significant results.

Dataset S3. ***"****Supplementary_file_S5.3_wgcna_gene_to_module_membership_20230413.xlsx"*

Module membership and position of hub genes identified from the weighted gene co-expression network analysis (WGCNA). The table lists gene ID, Entrez ID, gene symbol, gene name, module membership, and position for each hub gene. The position of the gene indicates its relative importance within the module.

Dataset S4. *"Supplementary_file_S5.4_results_limma_wgcna_20230413.xlsx"*

Differential gene expression analysis was performed within each module identified by Weighted Gene Co-Expression Network Analysis (WGCNA).

The analysis was conducted across three contrasts:

1) All rhizobacteria uninfected treatments vs. control uninfected.
2) All rhizobacteria and BPMV-infected treatments vs. control uninfected.
3) All rhizobacteria and BPMV-infected treatments vs. all rhizobacteria uninfected treatments.

The table includes the following information for each differentially expressed gene:

- Gene ID
- Treatment group names
- Module ID and module color (assigned by WGCNA)
- Number of genes in the module (n)
- Log2 fold change (logFC)
- T-statistic (t)
- P-value (P.Value)
- Adjusted p-value (adj.P.Val)

Dataset S5. *"Supplementary_file_S5.5_gage_pathways_result_20230413.xlsx"*

Significant enriched pathways from gene/metabolite enrichment analysis were identified across multiple contrasts, including comparisons between rhizobacteria for uninfected or BPMV-infected plants and for mixed virus treatments. Some of these comparisons are depicted in Figure 9.

Table Columns:

- **Regulation**: Indicates whether the pathway is upregulated or downregulated.
- **gmx_id**: The KEGG pathway identifier.
- **Path**: Name of the KEGG pathway.
- **Category1** and **Category2**: Classification categories of the pathway.
- **p.geomean**: Geometric mean of the p-values of the genes in the gene set. This provides an overall measure of the pathway's significance, reflecting the combined effect of all genes in the set.
- **stat.mean**: Mean log2 fold change of the genes in the gene set, indicating the overall direction and magnitude of gene expression changes (upregulated or downregulated).
- **p.val**: The unadjusted p-value for the gene set, calculated using a hypergeometric test, which measures the likelihood of observing the given number of genes in the set by chance.
- **q.val**: Adjusted p-value (or False Discovery Rate, FDR) of the gene set, corrected for multiple testing using the Benjamini-Hochberg method.
- **set.size**: The number of genes in the gene set.
- **exp1**: Enrichment score for each gene/metabolite set, representing the degree of over-representation of the genes/metabolites in the ranked list, relative to what would be expected by chance. Scores range from 0 to 1, with higher scores indicating greater over-representation. A score of 1 means all genes/metabolites in the set appear at the top of the ranked list, while a score of 0 means the set is randomly distributed in the list.

Dataset S6. *"Supplementary_file_S5.6_KEGG_pathways.zip"*

This zip file contains KEGG pathway graphs for each of the pair-wise comparisons in the table below. Each graph depicts a significantly perturbed pathway, where the majority of the signature genes and compounds were either upregulated or downregulated. The KEGG pathways maps are based on the

list of signature genes and metabolites used to derive an Integrated Transcriptome-Metabolome Signature (**ITMS**). The folder names are coded based on the specific pairwise comparison between treatment 1 and treatment 2.

folder name	virus	treatment1	treatment2
res.a.uninfected	uninfected	Bj	control
res.b.healthy	uninfected	Bj+Da	control
res.a.BPMV	BPMV	Bj	control
res.b.BPMV	BPMV	Bj+Da	control
res.c.BPMV	BPMV	Da	control
res.d.BPMV	BPMV	Bj+Da	Bj
res.a.mixed	mixed	control-BPMV	control-uninfected
res.b.mixed	mixed	Bj+Da-BPMV	Bj+Da-uninfected
res.c.mixed	mixed	Bj-BPMV	Bj-uninfected
res.d.mixed	mixed	Bj-BPMV	control-uninfected
res.e.mixed	mixed	Bj+Da-BPMV	control-uninfected
res.f.mixed	mixed	Da-BPMV	control-uninfected
res.g.mixed	mixed	allBacteria-uninfected	control-uninfected
res.h.mixed	mixed	allBacteria-BPMV	control-uninfected
res.i.mixed	mixed	allBacteria-uninfected	allBacteria-BPMV

Dataset S7. KEGG pathways identified through the analysis of fold changes in signature genes and metabolites in soybean plants induced by rhizobacteria and BPMV infection. Pathway perturbations were analyzed across three contrasts: A) all uninfected rhizobacteria treatments compared to control uninfected, B) all rhizobacteria and BPMV-infected treatments compared to control uninfected, and C) all rhizobacteria and BPMV-infected treatments compared to all uninfected rhizobacteria treatments. Each graph represents a significantly perturbed pathway, where most of the signature genes and compounds were either upregulated (top) or downregulated (bottom). Note that a KEGG node may represent multiple genes with similar functions. Additional significant pathways can be found in the Dataset S6 deposited in the ETHZ repository https://doi.org/10.3929/ethz-b-000692860. all Bacteria = *B. japonicum, B. japonicum + D. acidovorans,* and *D. acidovorans.*

REFERENCES

Allito BB, Ewusi-Mensah N, Logah V, Hunegnaw DK. **2021**. Legume-rhizobium specificity effect on nodulation, biomass production and partitioning of faba bean (*Vicia faba* L.). *Scientific Reports* **11**: 3678.

Barros De Carvalho GA, Batista JSS, Marcelino-Guimarães FC, Costa Do Nascimento L, Hungria M. **2013**. Transcriptional analysis of genes involved in nodulation in soybean roots inoculated with *Bradyrhizobium japonicum* strain CPAC 15. *BMC Genomics* **14**: 153.

Dean J, Mescher M, De Moraes C. **2014**. Plant Dependence on Rhizobia for Nitrogen Influences Induced Plant Defenses and Herbivore Performance. *International Journal of Molecular Sciences* **15**: 1466–1480.

Giesler LJ, Ghabrial SA, Hunt TE, Hill JH. **2002**. Bean pod mottle virus: a threat to US soybean production. *Plant Disease* **86**: 1280–1289.

Jiang Y, MacLean DE, Perry GE, Marsolais F, Hill B, Pauls KP. **2020**. Evaluation of beneficial and inhibitory effects of nitrate on nodulation and nitrogen fixation in common bean (*Phaseolus vulgaris*). *Legume Science* **2**: e45.

Jombart T, Devillard S, Balloux F. **2010**. Discriminant analysis of principal components: a new method for the analysis of genetically structured populations. *BMC genetics* **11**: 1–15.

Kontopoulou CK, Giagkou S, Stathi E, Savvas D, Iannetta PPM. **2015**. Responses of Hydroponically Grown Common Bean Fed with Nitrogen-free Nutrient Solution to Root Inoculation with N-2-fixing Bacteria. *Hortscience* **50**: 597–602.

Kuhn M. **2008**. Building predictive models in R using the caret package. *Journal of statistical software* **28**: 1–26.

Langfelder P, Horvath S. **2008**. WGCNA: an R package for weighted correlation network analysis. *BMC bioinformatics* **9**: 1–13.

Love MI, Huber W, Anders S. **2014**. Moderated estimation of fold change and dispersion for RNA-seq data with DESeq2. *Genome biology* **15**: 1–21.

Luo W, Brouwer C. **2013**. Pathview: an R/Bioconductor package for pathway-based data integration and visualization. *Bioinformatics* **29**: 1830–1831.

Luo W, Friedman MS, Shedden K, Hankenson KD, Woolf PJ. **2009**. GAGE: generally applicable gene set enrichment for pathway analysis. *BMC bioinformatics* **10**: 1–17.

Martín-Rodríguez JÁ, Leija A, Formey D, Hernández G. **2018**. The MicroRNA319d/TCP10 node regulates the common bean–rhizobia nitrogen-fixing symbiosis. *Frontiers in plant science* **9**: 1175.

Ohyama T, Fujikake H, Yashima H, Tanabata S, Ishikawa S, Sato T, Nishiwaki T, Ohtake N, Sueyoshi K, Ishii S. **2011**. Effect of nitrate on nodulation and nitrogen fixation of soybean. *Soybean physiology and biochemistry* **10**: 333–364.

Pulido H, Mauck KE, De Moraes CM, Mescher MC. **2019**. Combined effects of mutualistic rhizobacteria counteract virus-induced suppression of indirect plant defences in soya bean. *Proceedings of the Royal Society B: Biological Sciences* **286**: 20190211.

Ramula S, Mousavi SA, Kalske A. **2023**. Rhizobial benefits to an herbaceous invader depend on context and symbiotic strain. *Plant and Soil* **490**: 603–616.

Ritchie ME, Phipson B, Wu D, Hu Y, Law CW, Shi W, Smyth GK. **2015**. limma powers differential expression analyses for RNA-sequencing and microarray studies. *Nucleic acids research* **43**: 47–47.

Sánchez-Baizán N, Ribas L, Piferrer F. **2022**. Improved biomarker discovery through a plot twist in transcriptomic data analysis. *BMC biology* **20**: 1–26.

Streeter J, Wong PP. **1988**. Inhibition of legume nodule formation and N_2 fixation by nitrate. *Critical Reviews in Plant Sciences* **7**: 1–23.

Win KT, Wasai-Hara S, Tanaka F, Oo AZ, Minamisawa K, Shimoda Y, Imaizumi-Anraku H. **2023**. Synergistic N2-fixation and salt stress mitigation in soybean through dual inoculation of ACC deaminase-producing *Pseudomonas* and *Bradyrhizobium*. *Scientific Reports* **13**: 17050.
